# Interaction Structure and Affinity of Zwitterionic Amino Acids with Important Metal Cations (Cd^2+^, Cu^2+^, Fe^3+^, Hg^2+^, Mn^2+^, Ni^2+^ and Zn^2+^) in Aqueous Solution: A Theoretical Study

**DOI:** 10.3390/molecules27082407

**Published:** 2022-04-08

**Authors:** Xinning Liu, Menghan Wu, Chenchen Li, Peng Yu, Shanshan Feng, Yanwei Li, Qingzhu Zhang

**Affiliations:** 1 Environment Research Institute, Shandong University, Qingdao 266237, China; 201912787@mail.sdu.edu.cn (X.L.); zqz@sdu.edu.cn (Q.Z.); 2 State Key Joint Laboratory of Environmental Simulation and Pollution Control, School of Environment, Tsinghua University, Beijing 100084, China; wmh19@mails.tsinghua.edu.cn (M.W.); 15629126162@163.com (P.Y.); 3 School of Management, Ocean University of China, Qingdao 266101, China; lcc@ouc.edu.cn; 4 School of Chemistry and Chemical Engineering, Shandong University, Jinan 250100, China; 202120332@mail.sdu.edu.cn; 5 Shenzhen Research Institute, Shandong University, Shenzhen 518057, China

**Keywords:** amino acids, heavy metal cations, DFT, binding affinity, heavy metal and amino acid binary complexes

## Abstract

Heavy metals are non-biodegradable and carcinogenic pollutants with great bio-accumulation potential. Their ubiquitous occurrence in water and soils has caused serious environmental concerns. Effective strategies that can eliminate the heavy metal pollution are urgently needed. Here the adsorption potential of seven heavy metal cations (Cd^2+^, Cu^2+^, Fe^3+^, Hg^2+^, Mn^2+^, Ni^2+^ and Zn^2+^) with 20 amino acids was systematically investigated with Density Functional Theory method. The binding energies calculated at B3LYP-D3/def2TZVP level showed that the contribution order of amino acid side chains to the binding affinity was carboxyl > benzene ring > hydroxyl > sulfhydryl > amino group. The affinity order was inversely proportional to the radius and charge transfer of heavy metal cations, approximately following the order of: Ni^2+^ > Fe^3+^ > Cu^2+^ > Hg^2+^ > Zn^2+^ > Cd^2+^ > Mn^2+^. Compared to the gas-phase in other researches, the water environment has a significant influence on structures and binding energies of the heavy metal and amino acid binary complexes. Collectively, the present results will provide a basis for the design of a chelating agent (e.g., adding carboxyl or a benzene ring) to effectively remove heavy metals from the environment.

## 1. Introduction

With the rapid development of the global metal and mining industries, heavy metals are large accumulated in the upper crust and aquatic environment. Due to its particularities, non-biodegradability, bio-accumulation potential, and carcinogenicity, heavy metal pollution is a serious threat to human health and environmental sustainability [1]. Efficient and economical treatment of heavy metal pollution in the environment has become a subject that is now under the spotlight [2].

In general, heavy metals exist in the environment as cations, such as Cd^2+^, Hg^2+^, Mn^2+^, Ni^2+^, Zn^2+^, and so on [3]. They can remain on soil particles, suspended solids in water, and sediment particles in the environment by electrostatic attraction or chemical bonds with inorganic and organic ligand [2]. This has generated potential hazards to humans and ecosystems along the groundwater cycle and food chains [4]. At present, the treatment methods of heavy metal pollution in the environment include strong chelating agent cleaning, cement-based solidification and stabilization, and electric removal methods. In comparison, strong chelating agent adsorption is one of the preferred methods for the efficient and economical removal of toxic heavy metals [5]. However, the residues of non-degradable chelating agents (EDTA) has an effect on the natural biochemical processes, such as excessive interaction of EDTA with Ca^2+^, which can change soil structure [6]. In addition, non-selective complexation of EDTA with essential soil elements (potassium, calcium and magnesium) reduces the adsorption efficiency of EDTA [7].

In biology, a large number of metalloproteins need the cooperation of amino acids containing special groups to carry out biochemical reactions. The reason is that the functional groups and benzene rings on amino acids (AAs) side chains, easily complexing with corresponding metals cations, are able to play an important role in metal-catalyzed biochemical reactions [8]. Natural AAs offer distinct advantages in metal residue removal and complexing selectively with metal cations in the environment, so they can become more effective heavy metal chelators after modification [9]. For instance, Dolev et al. [10] found that amino acids (AAs) hardly interacted with essential elements in soil compared to EDTA through the control experiments of different soil samples. The better selectivity, competitiveness, and the ability of improving soil fertility of AAs were also observed in experiments.

Recently, different methods have been used, such as X-ray crystallography [7], infrared spectroscopy [11] and quantum chemical methods [9], to accurately model and quantitatively analyze the interaction systems between amino acids and metal cations. In the regioselectivity of metal cation complexation studies, the nitrogen ring structure in the side chain of histidine has a higher affinity for metal cations (as well as protons) than the phenyl ring of phenylalanine [12]. Through calculating the natural bond orbital (NBO), Umadevi et al. [13] explored orbital interactions and charge delocalizations during the complexation of phenylalanine with I A and II A groups metal cations. In research combining theory and experiment, Clark et al. [9] discovered the phenomenon that the radius of the Group 1 metal cation was inversely proportional to the affinity of lysine. Armentrout et al. [14] reported the results combining theoretical calculation and experiments during the complexion of Cs^+^ with different amino acids. Both theoretical calculations and experiments have observed that the order of affinity of Cs^+^ for various amino acids (Gly, Pro, Ser, Thr, and Cys) followed Cs^+^ (Gly) < Cs^+^ (Cys) < Cs^+^ (Ser) < Cs^+^ (Thr) < Cs^+^ (Pro).

The above theoretical studies are mainly focused on using quantum chemical methods to accurately search the potential energy surface (PES) and obtain stable complex configurations. Finally, the steric and thermodynamic properties were obtained by a theoretical calculation and model, almost agreeing with the experiment results. However, most researchers paid attention to the natural or neutral AAs experiments in the gas phase instead of those in the aqueous phase. Previous research [8] has reported that AAs were mainly zwitterionic in the aqueous phase. The large hydrogen bonds have a significant influence on the molecular form, steric and thermodynamic properties of amino acids in water solutions, which are different from those in the gas phase. The reported metal cations contain a variety of alkali metal groups or common metal cations, however, they are deficient in heavy metal cations in contaminated water and soil. Therefore, the interactions of zwitterionic AAs complexing with heavy metal cations in the environment must be investigated and discussed.

In this work, the complexation of seven heavy metal cations in the environment with the zwitterions of 20 normal amino acids in an aqueous solution was investigated with the Density Functional Theory (DFT) method. The metal cations studied included Cd^2+^, Cu^2+^, Fe^3+^, Hg^2+^, Mn^2+^, Ni^2+^ and Zn^2+^. The quantum chemical information of the above complexes was accurately calculated at B3LYP-D3/def2TZVP level, including stable conformer geometric optimization, the relative Gibbs free energy, binding energy and the charge transfer of the atoms. In addition, the affinities between 20 amino acids and seven heavy metal cations were systematically shown and discussed. The results will be helpful to guide the removal methods of heavy metal cations and reduce the heavy metal pollution in water and soil environments.

## 2. Computational Details

The Gaussian 09 [15] and Gauss View 5 [16] program packages were used to calculate, visualize and analyze the geometry optimization and frequency calculations of metal cations (Cd^2+^, Cu^2+^, Fe^3+^, Hg^2+^, Mn^2+^, Ni^2+^ and Zn^2+^), 20 AAs and their complexes (AA-metal cation). First of all, some conformations reported earlier were surveyed, including the conformations in the gas phase, in aqueous solutions with alkali metals [8,9,17]. Second, about 5–8 initial conformations were accordingly generated for each of the amino acid-metal cation binding complexes for initial screening. Finally, 2–5 of the most stable conformations with the lowest energy barriers were obtained. These conformations were used to calculate the complexation process.

B3LYP/6–311++G (d, p) level was applied to obtain relatively stable conformations of the above amino acids-metal cation complexes. Then, the def2TZVP basis set was used to more accurately calculate the quantum chemical information, including geometric optimization and frequency, enthalpy and Gibbs free energy, and the charge transfer of the atoms. The natural population analysis (NPA) was performed to observe the charge transfer in complexes [8]. All complexes optimization took place in aqueous phase with a universal solvation model (SMD), which was able to simulate the water environment around the molecule [18]. Basis set superposition error (BSSE) and the dispersion energy error in the amino acids-metal cations systems were impossible to ignore. So, DFT-D3 was used to compensate the calculation errors of standard DFT, which is currently the generic method used to calculate such interactions [19].

The binding energies (*E*_bind_) of amino acids interacting with metal cations are calculated from the energy difference between the individual molecules (amino acid molecule and the metal cation) and the amino acids-metal cations complexes (Equation (1)). The relative stability of the complexes is determined by the change of the relative Gibbs free energy (∆*G*) at 298 K, which is calculated by Equation (2), respectively. Furthermore, ∆*G*_min_ represents the most stable structure of Gibbs free energy in different conformers, respectively. Here *E*_AA_ and *E*_metal−cation_ are energies of amino acids and metal cations, respectively. It should be emphasized that the AA here retained the geometries and energies in the complex whose energy was thus different from the fully optimized AA. However, the relative Gibbs free energy (∆*G*) is calculated by the differences in various conformations of fully optimized AA. This is just an indicator of which binding conformation is more stable. *E*_bind_ (indicator of the binding potential) and ∆*G* (indicator of the stability of AA) are different from each other.

(1) $$E_{\mathrm{bind}}=-[E_{\mathrm{complex}}-\left(E_{\mathrm{AA}}+E_{\mathrm{metal}-\mathrm{cation}}\right)]$$

(2) $$∆G=∆G_{\mathrm{complex}}-∆G_{\min}$$

## 3. Results and Discussion

### 3.1. Determining the Most Stable Structure of 20 Amino Acids

**Figure 1:**
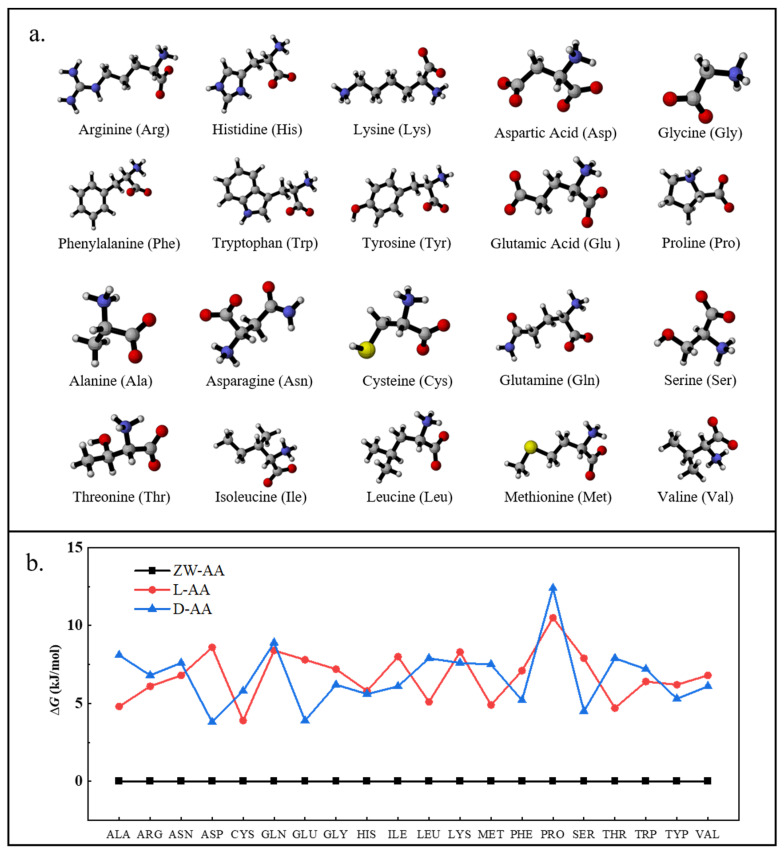
The stable configurations of 20 amino acids in a zwitterionic state (**a**) and the relative free energies (∆*G*) of 20 amino acids (**b**), including zwitterionic states (ZW-AA), left-handed natural AA (L-AA) and right -handed natural AA (D-AA).

Due to the ionization of amino acids in the aqueous solution, the H^+^ on the carboxyl group of the amino acid backbone easily escapes to the amino group, resulting in the protonated amino group and the zwitterionic AA. Here, the ∆*G* and geometrical configuration of 20 AAs in natural and zwitterionic forms were explored in Figure 1. Obviously, the ∆*G* of 20 AAs in zwitterionic state was lower than that of natural AAs, which indicated that zwitterionic AAs were more stable in water. This was consistent with previous studies^9^ that showed AAs were mainly zwitterionic in the aqueous phase, while AAs were naturally or neutrally present in the gas phase. It was speculated that the abundant presence of amphoteric amino acids (ZW-AAs) in an aqueous solution might be caused by hydrogen bonding. Conversely, ZW-AAs were not stable in the gas phase. What’s more, Gochhayat et al. [20] used the LMOEDA method to calculate the polarization, electrostatic and dispersion energies for each of the hydrated clusters (AAs-water molecules) at MP2/6-311++G (d, p) level. The results indicated that polarization and electrostatic interactions were more positive for the stability of zwitterionic AAs in aqueous solution.

In practice, amino acids were affected by pH to become double cations, double anions, zwitterionic ions, single cations, and single anions. Since an aqueous solution model was simulated in the optimized process, the calculated results were close to the actual water environment of pH 7. The results of geometric optimization (Figure 1a) found that the carboxyl group on the side chain of the acidic AAs lost a H^+^ due to ionization, while the amino group of the basic AAs seized the H^+^ in the aqueous solution, forming anionic and cations AAs (Figure 1a), respectively. According to reported studies [21,22,23,24,25,26], most basic amino acids (Arginine, Histidine and Lysine) existed as the form of single cations in water solution at pH 7, as the isoelectric point (pI) of basic amino acids was bigger than seven; most of the acidic amino acids (Aspartic acid and Glutamic acid) existed as the form of single anions in the solution, whose pI was smaller than seven; and the pI of neutral amino acids was close to seven, and most neutral amino acids existed as the form of amphoteric ions in the solution. Therefore, in this study, the geometric structures of AAs in the water environment with pH = seven were optimized and accepted to participate in the complexation process with heavy metal cations at B3LYP-D3/def2TZVP level.

### 3.2. Structural Characterization of Binary Complexes

#### 3.2.1. Acidic Amino Acids and Metal Cation

**Figure 2:**
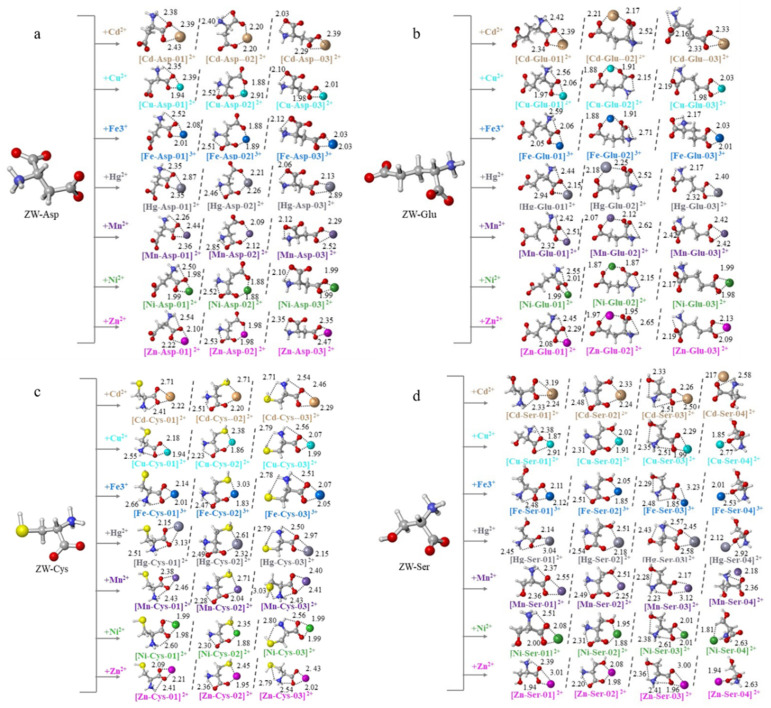
The optimized structures of [acidic/sulfhydryl/hydroxy AA-metal cation] complexes. AA = Asp (**a**), Glu (**b**), Cys (**c**), Ser (**d**); the numbers in the figure indicated the hydrogen bond length in a complex molecule, and the distance between the metal cation and the interacting atom; the length in Å.

For 20 normal AAs, the amino acids with a carboxyl group on the side chain are named as acidic amino acids, such as aspartic acid (Asp) and glutamic acid (Glu). The DFT (B3LYP/def2TZVP/ SMD) predicted the conformations of Asp and Glu in the water solvation model (Figure 2), which were consistent with previous research [27,28].

The calculated results indicated that the complexes of [acidic AAs-metal cation] had two geometric models: salt bridge (SB) mode and charge solvation (CS) mode [9,29]. In the first one, the metal cation bonded to a carboxyl group with negative charge (COO^−^), liking the Structure [M*^n+^*-AA-01] and [M*^n+^*-AA-03] in Figure 2 (M = Cd, Cu, Fe, Hg, Mn, Ni, Zn; *n* = 2,3). In the later mode, the metal cation interacted with two carbonyls (C=O), liking the structure [M*^n+^*-AA-02]. In the acidic AA-metal cations complexes, it was observed that the structure [M*^n+^*-AA-02] was the most stable by comparing the ∆*G* of three configurations (Table S1). Two negatively charged carboxyl groups provided a strong binding force between metal cations and amino acids to form CS mode, which broke through barriers from hydrogen bonds and the molecular chain elongation (Glu had one more carbon molecule than Asp in chain length). The metal cation interacting with two carbonyls (C=O) and amino group (-NH_2_), a tridentate binding [N, O, O], was the lowest energy structure in the gas-phase in previous studies [30,31,32]. The solvation model made the amino group electropositive and repulsive to metal cations, so that the most stable conformation in this study was the bidentate structure [M*^n+^*-AA-02].

#### 3.2.2. Alkaline Amino Acids and Metal Cation

In the water solution, the zwitterion of alkaline AAs, Arginine (Arg) Histidine (His) and Lysine (Lys), was equipped with an electronegative amino group on the side chain, which took a hydrogen ion from aqueous solution to form a positive amino group (-NH_3_^+^).

Two stable structures were obtained by the optimization results (Figure S1) and the ∆*G* (Table S1). One was the SB mode, in which metal cations symmetrically bonded with two O atoms on the carboxyl group, and the other one was the CS mode, where metal cations only bonded with an O atom. The SB mode was more stable by the ∆*G* calculation analysis. However, the difference in ∆*G* between the two structures of alkaline AAs was lower, compared to that of acidic AAs. For instance, the relative energy difference between [Cd^2+^-Arg-01] and [Cd^2+^-Arg-01] was about 8 kJ/mol, however, the value of [Cd^2+^-Asp-01] (SB structure) was about 44 kJ/mol higher than [Cd^2+^-Asp-02] (CS structure). Similar results were found in other studies. The relative energy of the SB structure was 40 kJ/mol higher than the CS structure in normal Aspartic acid interacting with copper cation [27]. Clark et al. [9] reported that the chelates of the Group 1 metal cations with Lysine generated an increase of 0–8 kJ/mol relative energy from the SB structure to the CS structure.

#### 3.2.3. Hydroxy/Sulfhydryl Amino Acids and Metal Cation Complexes

The hydroxyl group in serine (Ser) and the sulfhydryl group in cysteine (Cys) have more functional groups than glycine, making up more intermolecular hydrogen bonds, which results in the less deformation of complexes and the more stable molecules [28].

In calculated results, the Cys-metal cation and Ser-metal cation complexes had three similar optimized structures (Figure 1). Both [M*^n+^*-AA-01] and [M*^n+^*-AA-03] were SB modes. The difference was that [M*^n+^*-AA-03] had one more hydrogen bond, NH⋯S/O than [M*^n+^*-AA-01]. [M*^n+^*-AA-02] was CS mode, whose metal cations bound with an O atom on the carbonyl and an O/S atom of hydroxyl/sulfhydryl whose metal cations bound with an O atom on the carbonyl and an O/S atom of hydroxyl/sulfhydryl group. In addition, Ser-metal cation complexes had the [M*^n+^*-AA-04] structure, in which the metal cations bound to an O atom on the carbonyl. The ∆*G* of optimized structures (Table S1) showed that [M*^n+^*-AA-03] and [M*^n+^*-AA-02] were the most stable structures of cysteine and serine, respectively. Khodabandeh et al. [28] reported that the lowest relative energy was obtained at the [M*^n+^*-Cys-03] structure during the interaction of cysteine with Mn^2+^. In the complexes of [M*^n+^*-Ser-02], the hydroxyl group of the side chain provides more electronegative attraction for the metal cations than sulfhydryl group, so more [M*^n+^*-Ser-02] were obtained in water environment. For the Ser-metal cations complexes, Li^+^ [33], Na^+^ [12], K^+^ [34] and Rb^+^ [14] preferred the tridentate [N, CO, OH] structure. The zwitterionic AAs made the amino group more electropositive to the resistant metal cation, so the tridentate mode was hardly obtained in the water solvent model in this work. However, an interesting result was found; the [M*^n+^*-Ser-02] became more popular as the radius of the alkali metal increased [14].

#### 3.2.4. Aromatic Amino Acids and Metal Cation Complexes

To explore the influence of the side chain phenyl during the complexation process, phenylalanine (Phe), Tryptophan (Try), and Tyrosine (Tyr) were paid attention to herein. The stable conformers containing both the SB and CS modes were obtained in Figure S1. For the SB mode, metal cations symmetrically bound with the aromatic AAs carboxyl as AA-metal cations complexes, as mentioned earlier. For the CS mode, metal cations interacted with both phenyl and carbonyl groups. In addition, the complexes of Tryptophan with metal cations had another CS mode, [M*^n+^*-Trp-03]. As shown in Figure S1e, the metal cations interacted with the pyrrole in the indole ring and carbonyl.

According to the relative energy results of the conformers (Table S1), the aromatic AA- metal cation conformers preferred the [M*^n+^*-AA-02] structure. In the HOMO calculated results [35], a large number of the highest electron-occupied orbitals appeared at the benzene ring position. The electronegativity of the phenyl group and the carboxyl group together stabilized the metal cations, which was more effective than the carboxyl group alone. However, the phenyl group in the side chain was sometime unfriendly to metal cations. The cations radii were so large that the phenyl group in the CS mode were not able to stop their active trend, resulting in similar energy to the SB mode. For instance, the relative difference in energy was less than 5–10 kJ/mol between [M*^n+^*-AA-01] and [M*^n+^*-AA-02], M=Hg, Cd; AA=Phe, Trp, Tyr. Similarly, the [CO, O^−^] structures (SB mode) became isoenergetic, especially for K^+^ and Rb^+^ in the Group 1 alkali metals [36,37].

#### 3.2.5. Other Amino Acids and Metal Cation Complexes

In total, 10 amino acids were considered and discussed here, including Asparagine (Asn), Glutamine (Gln), Threonine (Thr), Methionine (Met), Proline (Pro), Glycine (Gly), Alanine (Ala), Valine (Val), Leucine (Leu), and Isoleucine (Ile).

Asn and Gln were the amino acids carrying an acyl group, derived from acidic amino acids. Similar to Asp-metal cation complexes, the Asn-metal cation complexes generated four conformers (Figure S2a) and the most stable complex was [M*^n+^*-Asn-02]. The results indicated that the metal cations preferred to simultaneously combine with [C=O] from the amide and carboxyl groups. In addition, an interesting conformer was found. In the structures [M*^n+^*-Asn-04], a CS mode, the metal cations interacted with O and N of the amide group on the amino acid side chains, which made structure stabilities close to SB conformers, [M*^n+^*-Asn-01]. However, the complexes of the Gln-metal cations were different to those of the Glu-metal cations. In Figure S2b, a SB complex and four CS complexes were obtained by being optimized. Due to the extension of the carbon chain, it was difficult for metal cations to interact with the O on the amide group, so that the SB structure (M*^n+^*-Gln-01) and a new CS structure, [M*^n+^*-Gln-03], became more stable (Table S1). Infrared Multiple Photon Dissociation Spectroscopy (IRMPD) and theoretical studies [38] also found that zwitterionic [O,O^−^] structures (SB mode) were favored for larger cations (K^+^, Rb^+^, and Cs^+^) and that the [N,CO,CO] structures contributed more to smaller cations (Li^+^, Na^+^) in the gas-phase.

Two types of conformations, SB structures and CS structures, were found by optimizing the conformers of metal cations with Thr, Met and Pro. However, the [S] CS structure was obtained in the conformer instead of the predicted [SO] CS structure. As described above for hydroxyl and sulfhydryl AAs complexing with metal cations, the stable structures of Thr/Met-metal cation complexes were the CS and SB structures, respectively (Figure S2). The stable structures of the Pro-metal cations complexes were the SB structure. The results indicated that in the zwitterionic Pro the pyridine ring binding to hydrogen ions was less electronegative than the benzene ring, which hindered it from interacting with metal cations. The same low-energy structures were found in gas-phase experiments and theoretical studies. When alkali metals complexed with Thr, Met and Pro, the difference in relative energy values were 0–10 kJ [14,36]. The values were lower than those in a water environment, which showed the more stable and unified structures in solvation models.

The optimized structures of five amino acids without a special functional group, Glycine (Gly), Alanine (Ala), Valine (Val), Leucine (Leu) and Isoleucine (Ile), were shown in Figure S3. The SB structures and the CS structures were obtained by five optimized AAs. Unlike other AAs, Ala, Val and Leu interacted with metal cations generating two CS structures, [NO] structure and [O] structure. Though the most stable structures were SB structures (Table S1), all conformers except the AA-Ni^2+^ conformers had similar energy values with differences of 6–15 kJ/mol. The reason was most likely that the smallest radius of nickel cation leads to a preference for SB structures over CS structures. The previous studies [14,28] reported that the difference in ∆*G* was about 5.4 kJ/mol between SB and CS structures in the Lys-Cs^+^ conformers and Gly, Ala, Val, Leu and Ile were the favorites to bind with alkali metal cations by the SB structures.

As reported earlier [28,36], in the 20 AAs-metal cations complexes structures, the distance of M*^n+^*--O lengthened as the metal cations radii increased. The M*^n+^*--O distance of [M*^n+^*-AA] conformers lengthened in the order Hg^2+^ > Cd^2+^> Mn^2+^> Zn^2+^> Cu^2+^> Fe^3+^>Ni^2+^. The order of M*^n+^*--O from Hg^2+^ to Ni^2+^ was approximately similar to the ionic radii for Hg^2+^ (1.19 Å), Cd^2+^ (0.95 Å), Zn^2+^ (0.74 Å), Cu^2+^ (0.73 Å), Mn^2+^ (0.67 Å), Fe^3+^ (0.64 Å), Ni^2+^ (0.56 Å), respectively [39]. Obviously, the metal cation radii played an important role in the geometric conformation of complexes.

### 3.3. The Binding Affinity of 20 Amino Acids with Seven Metal Cations

The quantitative information of affinities between AAs and metal cations can provide a better understanding of the AAs-metal cations systems. The Mulliken charge and *E_bind_* results of 20 AAs-metal cations complexes were obtained by DFT calculation and analysis (Figure S4). It was investigated that the stabilities were closely related to affinities (*E_bind_*) and metal charge transfer (∆Q_M_) in the complexes. For instance, [M*^n+^*-Asp-02], [M*^n+^*-Ala-01] and [M*^n+^*-Tyr-02] structures were not only the most stable structure, but also had the highest *E_bind_* and ∆Q_M_ (Figure S4c,d,s).

**Figure 3:**
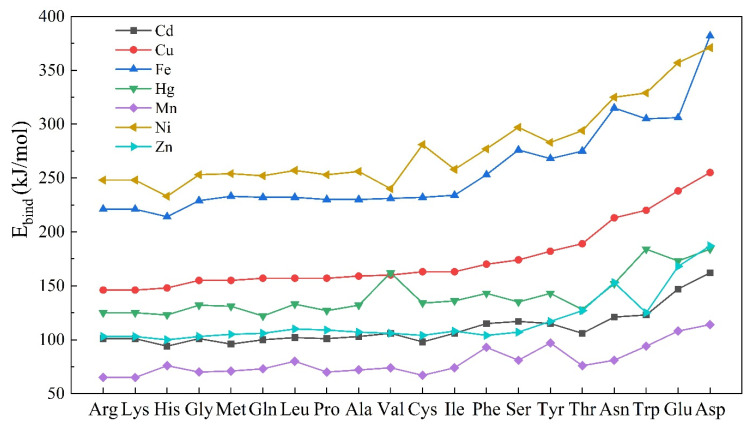
The comparison of the most stable complex binding energies during 20 AAs and seven kinds of metal cation interactions.

As for the same AAs, the affinity and charge transfer were positively correlated in AA-metal cations complexes. For example, when Glu interacted with metal cations in [M*^n+^*-Glu-02] mode, the increasing order of ∆Q_M_ was as follows: Fe^3+^ (0.97) > Ni^2+^ (0.87) > Cu^2+^ (0.77) > Zn^2+^ (0.65) > Hg^2+^ (0.54) > Cd^2+^ (0.47) > Mn^2+^ (0.39), which approximately correlated with the *E_bind_* order: Ni^2+^ (357.6) > Fe^3+^ (306.9) > Cu^2+^ (238.7) > Hg^2+^ (173.8) > Zn^2+^ (168.5) > Cd^2+^ (147.3) > Mn^2+^ (108.7). Small metal cations had better spatial structure and more charge transfer, which was consistent with Lewis-acids nature and led to higher affinity for AAs. Among the *E_bind_* comparisons of 20 amino acids (Figure 3), iron and nickel cations with small radii had obvious advantages in affinities to all AAs. The bond distances necessarily lengthened, resulting in a weakening of electrostatic interactions. Due to the cation radii having increased from 0.70 to 1.67 Å as the Group 1 alkali metals from Li^+^ to Cs^+^, the bond energies became weaker between the AA and alkali ions [10]. Similar phenomena [40] have been reported that when the amino acids without aromatic rings or functional groups in the side chain (Gly, Ala, Ile, Leu, and Val) interacted with alkali metals, the interaction energies trended to Li^+^ > Na^+^ > K^+^. However, Mn^2+^ showed weaker Lewis-acids nature and lower *E_bind_* than other small metal cations in the complexation (Figure 3). The previous studies [41] found that Fe^3+^, Ni^2+^ and Cu^2+^ interacted more easily with the common amino acids than Mn^2+^.

As shown in Figure 3, the trend of affinity with metal cations was Asp > Glu >Trp > Asn > Thr > Tyr > Ser > Phe > Ile > Cys > Val > Ala > Pro > Leu > Gln > Met > Gly > His > Lys > Arg. Negatively charged carboxyl and benzene rings helped the corresponding amino acid to be more stably complex with metal cations. However, through the comparison of theoretical and experimental binding energies in the gas-phase, Armentrout et al. [42] found that the energy values followed the order Asp < Glu < Asn < Gln, associated with the polarizability of AAs molecular. In this work, the water environment might have an influence on the polarizability of four AAs, leading to the changes in their binding energies.

## 4. Conclusions

To explain the mechanism of complexation between amino acids and heavy metals, and help effectively remove heavy metals from the environment, the adsorption potential of 20 amino acids with seven heavy metal cations (Cd^2+^, Cu^2+^, Fe^3+^, Hg^2+^, Mn^2+^, Ni^2+^ and Zn^2+^) was systematically investigated with the DFT method. At the B3LYP-D3/def2TZVP level, the optimized geometry results indicated that the zwitterionic amino acids (ZW-AAs) were abundant in aqueous solutions due to the effects of hydrogen bonds and amino protonation. For AAs with different side chains, the contribution trend of the side chains to heavy metal cation (HMC) affinity was: carboxyl group > benzene ring > hydroxyl group > sulfhydryl group > amino group. AAs were more attractive to metal cations with a smaller radius following the order: Ni^2+^ >Fe^3+^ > Cu^2+^ > Hg^2+^ > Zn^2+^ > Cd^2+^ > Mn^2+^. The AAs-affinity of Mn^2+^ was lower than that of other metals due to weak Lewis acid properties. In addition, the order of affinity of all AAs for HMC followed Asp > Glu >Trp > Asn > Thr > Tyr > Ser > Phe > Ile > Cys > Val > Ala > Pro > Leu > Gln > Met > Gly > His > Lys > Arg. Collectively, the present results will provide the basis of guidance for the removal methods of heavy metal cations and reduce the heavy metal pollution in water and soil environments.

## Data Availability

No new data were created or analyzed in this study. Data sharing is not applicable to this article.

## Supplementary Materials

The following supporting information can be downloaded at: https://www.mdpi.com/article/10.3390/molecules27082407/s1, Figure S1: The optimized structures of [alkaline/aromatic AA-metal cation] complexes; Figure S2: The optimized structures of [AA-metal cation] complexes, AA=Asn (a), Gln (b), Thr (c), Met (d), Pro (e); Figure S3: The optimized structures of [AA-metal cation] complexes, AA= Gly (a), Ala (b), Val (c), Leu (d), and Ile (e); Figure S4: The 20 types of [AA-metal cation] complexes binding energy and Mullikan charge of metal cations; Table S1: The relative Gibbs free energy (∆*G*) of AAs-metal cations structures; Text S1. The cartesian coordinates information of converged conformations.

## Sample Availability

Samples of the compounds are not available from the authors.

## References

[1] Osuna F., Pavón E., Alba M. (2020). Pb^2+^, Cd^2+^ and Hg^2+^ removal by designed functionalized swelling high-charged micas. Sci. Total Environ..

[2] Xu J., Liu C., Hsu P.-C., Zhao J., Wu T., Tang J., Liu K., Cui Y. (2019). Remediation of heavy metal contaminated soil by asymmetrical alternating current electrochemistry. Nat. Commun..

[3] Leštan D., Luo C.-L., Li X. (2008). The use of chelating agents in the remediation of metal-contaminated soils: A review. Environ. Pollut..

[4] Wuana R.A., Okieimen F.E., Imborvungu J.A. (2010). Removal of heavy metals from a contaminated soil using organic chelating acids. Int. J. Environ. Sci. Technol..

[5] Begum Z.A., Rahman I.M., Tate Y., Sawai H., Maki T., Hasegawa H. (2012). Remediation of toxic metal contaminated soil by washing with biodegradable aminopolycarboxylate chelants. Chemosphere.

[6] Jelusic M., Lestan D. (2014). Effect of EDTA washing of metal polluted garden soils. Part I: Toxicity hazards and impact on soil properties. Sci. Total Environ..

[7] Gluhar S., Kaurin A., Finžgar N., Gerl M., Kastelec D., Lestan D. (2021). Demonstrational gardens with EDTA-washed soil. Part I: Remediation efficiency, effect on soil properties and toxicity hazards. Sci. Total Environ..

[8] Umadevi P., Senthilkumar L. (2014). Influence of metal ions (Zn^2+^, Cu^2+^, Ca^2+^, Mg^2+^ and Na^+^) on the water coordinated neutral and zwitterionicl-histidine dimer. RSC Adv..

[9] Clark A.A., Yang B., Rodgers M.T., Armentrout P.B. (2019). Experimental and Computational Study of the Group 1 Metal Cation Chelates with Lysine: Bond Dissociation Energies, Structures, and Structural Trends. J. Phys. Chem. B.

[10] Dolev N., Katz Z., Ludmer Z., Ullmann A., Brauner N., Goikhman R. (2020). Natural amino acids as potential chelators for soil remediation. Environ. Res..

[11] Rodgers M.T., Armentrout P.B., Oomens J., Steill J.D. (2008). Infrared Multiphoton Dissociation Spectroscopy of Cationized Threonine: Effects of Alkali-Metal Cation Size on Gas-Phase Conformation. J. Phys. Chem. A.

[12] Liikanen M., Havukainen J., Hupponen M., Horttanainen M. (2017). Influence of different factors in the life cycle assessment of mixed municipal solid waste management systems—A comparison of case studies in Finland and China. J. Clean. Prod..

[13] Alirezapour F., Khanmohammadi A. (2021). Theoretical study on the interaction of phenylalaninal with group IA (Li^+^, Na^+^, K^+^) and IIA (Be^2+^, Mg^2+^, Ca^2+^) metal cations. J. Chin. Chem. Soc..

[14] Armentrout P.B., Chen Y., Rodgers M.T. (2012). Metal Cation Dependence of Interactions with Amino Acids: Bond Energies of Cs^+^ to Gly, Pro, Ser, Thr, and Cys. J. Phys. Chem. A.

[15] Frisch M.J., Trucks G.W., Schlegel H.B., Scuseria G.E., Robb M.A., Cheeseman J.R., Scalmani G., Barone V., Petersson G.A., Nakatsuji H. (2010). Gaussian 09.

[16] Dennington R., Keith T., Millam J. (2009). GAUSSVIEW.

[17] Shankar R., Kolandaivel P., Senthilkumar L. (2011). Interaction studies of cysteine with Li^+^, Na^+^, K^+^, Be^2+^, Mg^2+^, and Ca^2+^ metal cation complexes. J. Phys. Org. Chem..

[18] Marenich A.V., Cramer C.J., Truhlar D.G. (2009). Universal Solvation Model Based on Solute Electron Density and on a Continuum Model of the Solvent Defined by the Bulk Dielectric Constant and Atomic Surface Tensions. J. Phys. Chem. B.

[19] Grimme S. (2011). Density functional theory with London dispersion corrections. WIREs Comput. Mol. Sci..

[20] Gochhayat J.K., Dey A., Pathak A.K. (2019). An ab iniio study on the micro-solvation of amino acids: On the number of water molecules necessary to stabilize the zwitter ion. Chem. Phys. Lett..

[21] Ustunol I.B., Gonzalez-Pech N., Grassian V.H. (2019). pH-dependent adsorption of α-amino acids, lysine, glutamic acid, serine and glycine, on TiO_2_ nanoparticle surfaces. J. Colloid Interface Sci..

[22] Yang Y., Wang S., Liu J., Xu Y., Zhou X. (2016). Adsorption of Lysine on Na-Montmorillonite and Competition with Ca^2+^: A Combined XRD and ATR-FTIR Study. Langmuir.

[23] Kawamura I., Sato H. (2019). Solid-state vibrational circular dichroism studies of L- and D-serine. Anal. Biochem..

[24] Quesada-Moreno M.M., Avilés-Moreno J.R., Márquez-García A.A., López-González J.J. (2014). Deducing the molecular properties of zwitterionic, protonated, deprotonated, and double-deprotonated forms of L-cysteine from vibrational spectroscopy (IR, Raman, VCD) and quantum chemical calculations. J. Mol. Model..

[25] Quesada-Moreno M.M., Márquez-García A., Avilés-Moreno J.R., López-González J.J. (2013). Conformational landscape of l-threonine in neutral, acid and basic solutions from vibrational circular dichroism spectroscopy and quantum chemical calculations. Tetrahedron Asymmetry.

[26] Stückenschneider K., Merz J., Schembecker G. (2014). Molecular Interaction of Amino Acids with Acidic Zeolite BEA: The Effect of Water. J. Phys. Chem. C.

[27] Meng L., Hu A., Pang R., Lin Z. (2012). Extensive Computational Study on Coordination of Transition Metal Cations and Water Molecules to Glutamic Acid. J. Phys. Chem. A.

[28] Khodabandeh M.H., Reisi H., Davari M.D., Zare K., Zahedi M., Ohanessian G. (2013). Interaction Modes and Absolute Affinities of α-Amino Acids for Mn^2+^: A Comprehensive Picture. ChemPhysChem.

[29] Meng L., Lin Z. (2014). Complexations of alkali/alkaline earth metal cations with gaseous glutamic acid. Comput. Theor. Chem..

[30] Xiang F., Bu Y., Ai H., Li P. (2004). The Coupling Character of Ca^2+^ with Glutamic Acid: Implication for the Conformational Behavior and Transformation of Ca^2+^-ATPase in Transmembrane Ca^2+^ Channel. J. Phys. Chem. B.

[31] Heaton A.L., Armentrout P.B. (2008). Experimental and Theoretical Studies of Potassium Cation Interactions with the Acidic Amino Acids and Their Amide Derivatives. J. Phys. Chem. B.

[32] Heaton A.L., Ye S.J., Armentrout P.B. (2008). Experimental and Theoretical Studies of Sodium Cation Complexes of the Deamidation and Dehydration Products of Asparagine, Glutamine, Aspartic Acid, and Glutamic Acid. J. Phys. Chem. A.

[33] Bowman V.N., Heaton A.L., Armentrout P.B. (2010). Metal Cation Dependence of Interactions with Amino Acids: Bond Energies of Rb^+^ to Gly, Ser, Thr, and Pro. J. Phys. Chem. B.

[34] Talley J.M., Cerda B.A., Ohanessian G., Wesdemiotis C. (2002). Alkali Metal Ion Binding to Amino Acids Versus Their Methyl Esters: Affinity Trends and Structural Changes in the Gas Phase. Chem.—A Eur. J..

[35] Hossain M.E., Hasan M.M., Halim M.E., Ehsan M.Q., Halim M.A. (2015). Interaction between transition metals and phenylalanine: A combined experimental and computational study. Spectrochim. Acta Part A Mol. Biomol. Spectrosc..

[36] Armentrout P.B., Yang B., Rodgers M.T. (2013). Metal Cation Dependence of Interactions with Amino Acids: Bond Energies of Rb^+^ and Cs^+^ to Met, Phe, Tyr, and Trp. J. Phys. Chem. B.

[37] Ruan C., Rodgers M.T. (2004). Cation−π Interactions: Structures and Energetics of Complexation of Na^+^ and K^+^ with the Aromatic Amino Acids, Phenylalanine, Tyrosine, and Tryptophan. J. Am. Chem. Soc..

[38] Bush M.F., Oomens J., Saykally R.J., Williams E.R. (2008). Alkali Metal Ion Binding to Glutamine and Glutamine Derivatives Investigated by Infrared Action Spectroscopy and Theory. J. Phys. Chem. A.

[39] Harvey K.B., Porter G.B., Porter G.B. (1963). Introduction to Physical Inorganic Chemistry.

[40] Jover J., Bosque R., Sales J. (2008). A comparison of the binding affinity of the common amino acids with different metal cations. Dalton Trans..

[41] Marino T., Toscano M., Russo N., Grand A. (2006). Structural and Electronic Characterization of the Complexes Obtained by the Interaction between Bare and Hydrated First-Row Transition-Metal Ions (Mn^2+^, Fe^2+^, Co^2+^, Ni^2+^, Cu^2+^, Zn^2+^) and Glycine. J. Phys. Chem. B.

[42] Armentrout P.B., Yang B., Rodgers M.T. (2014). Metal Cation Dependence of Interactions with Amino Acids: Bond Dissociation Energies of Rb^+^ and Cs^+^ to the Acidic Amino Acids and Their Amide Derivatives. J. Phys. Chem. B.

