# Supplementary material for: Interaction Structure and Affinity of Zwitterionic Amino Acids with Important Metal Cations (Cd2+, Cu2+, Fe3+, Hg2+, Mn2+, Ni2+ and Zn2+) in Aqueous Solution: A Theoretical Study
Source: Molecules. 2022 Apr 8;27(8):2407. doi: 10.3390/molecules27082407 (PMC9028192; doi:10.3390/molecules27082407)
Supplement: Supplementary file 1 [file molecules-27-02407-s001.zip › molecules-1642385-supplementary.pdf]

Supporting Information

Xinning Liu[1], *Menghan Wu*[2], *Chenchen Li*[5], *Peng Yu*[2], *Shanshan Feng*[3], *Yanwei Li*[1, 4]*, *Qingzhu Zhang*[1]

[1] Environment Research Institute, Shandong University, Qingdao 266237, P. R. China.

[2] State Key Joint Laboratory of Environmental Simulation and Pollution Control, School of Environment, Tsinghua University, Beijing 100084, P.R. China.

[3] School of Chemistry and Chemical Engineering, Shandong University, Jinan 250100, P. R. China.

[4] Shenzhen Research Institute, Shandong University, Shenzhen 518057, P.R. China.

[5] School of Management, Ocean University of China, Qingdao 266101, P. R. China.

*Corresponding author: Tel: +86-0532-58631990; E-mail: lyw@sdu.edu.cn

Supplementary material contains following:

Figure S1. The optimized structures of [alkaline/aromatic AA-metal cation] complexes.

Figure S2. The optimized structures of [AA-metal cation] complexes, AA=Asn (a), Gln (b), Thr (c), Met (d), Pro (e).

Figure S3. The optimized structures of [AA-metal cation] complexes, AA= Gly (a), Ala (b), Val (c), Leu (d), and Ile (e).

Figure S4. The 20 types of [AA-metal cation] complexes binding energy and Mullikan charge of metal cations.

Table S1. The relative Gibbs free energy (Δ*G*) of AAs-metal cations structures.

Text S1. The cartesian coordinates information of converged conformations.

FIGURES.

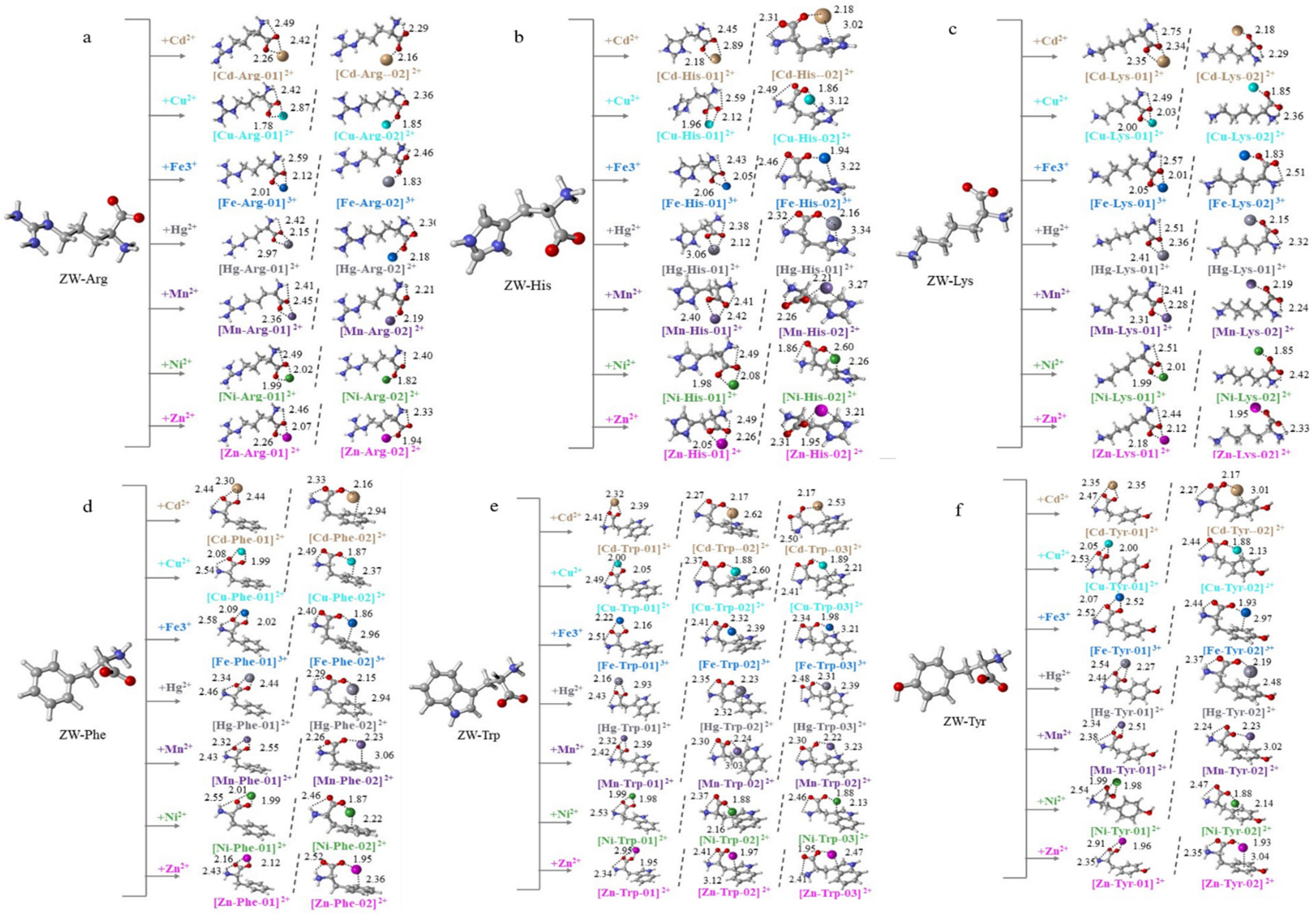

Figure S1. The optimized structures of [alkaline/aromatic AA-metal cation] complexes. AA=Arg (a), His (b), Lys (c), Phe (d), Trp (e), Tyr (f); the numbers in the figure indicated the hydrogen bond length in complex molecule, and the distance between the metal cation and the interacting atom; the length in Å

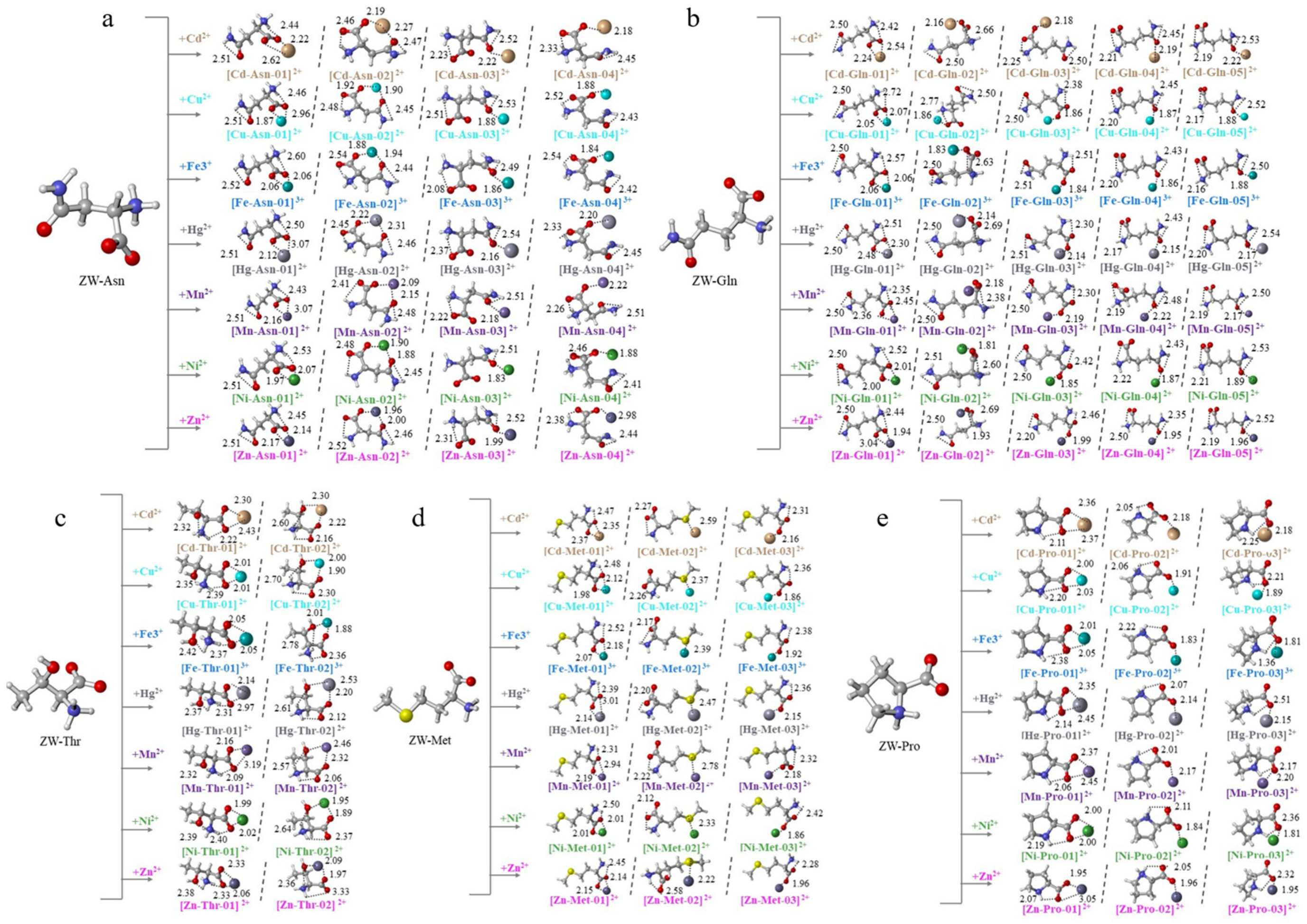

Figure S2. The optimized structures of [AA-metal cation] complexes, AA=Asn (a), Gln (b), Thr (c), Met (d), Pro (e). The numbers in the figure indicated the hydrogen bond length in complex molecule, and the distance between the metal cation and the interacting atom; the length in Å.

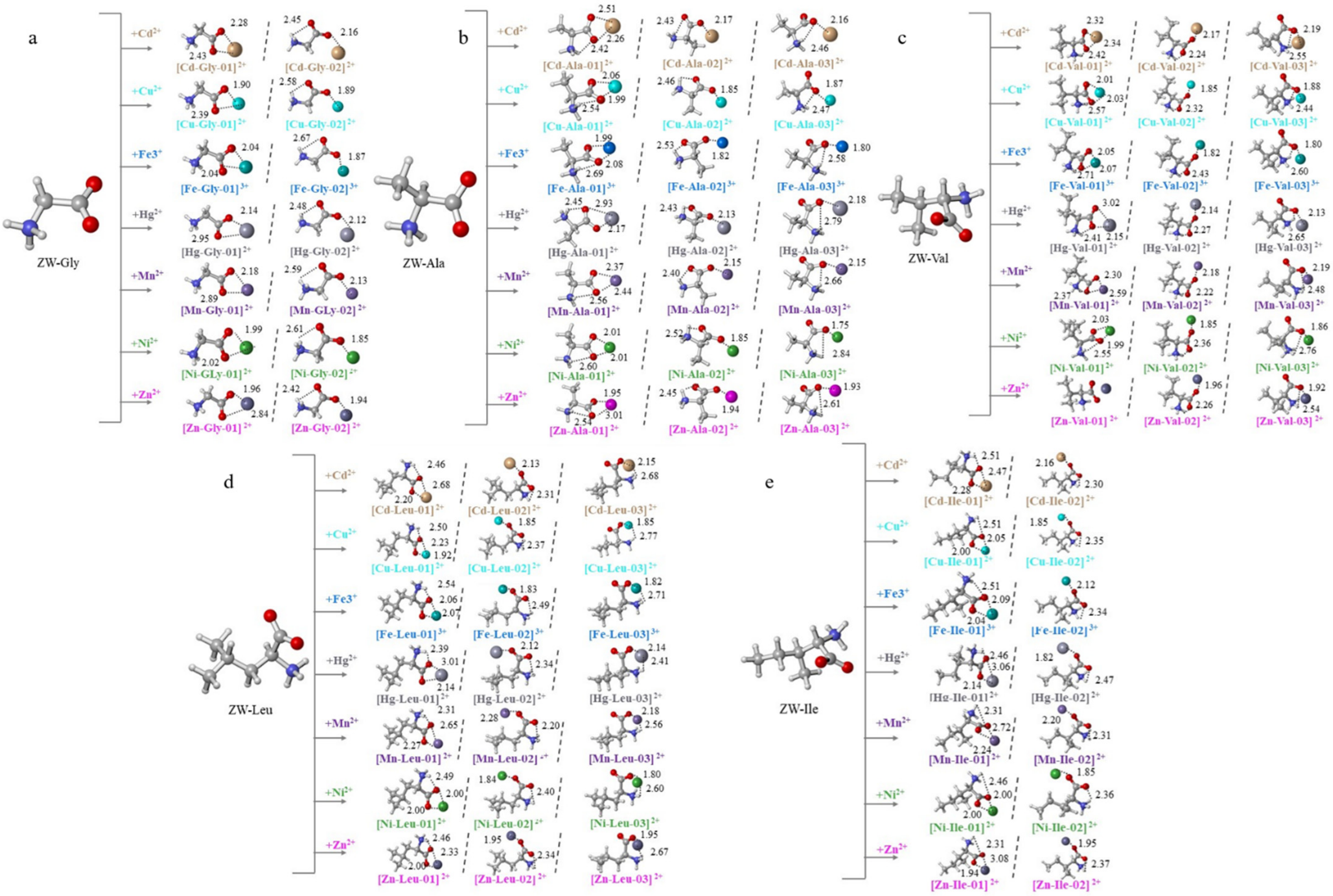

Figure S3. The optimized structures of [AA-metal cation] complexes, AA= Gly (a), Ala (b), Val (c), Leu (d), and Ile (e). The length in Å.

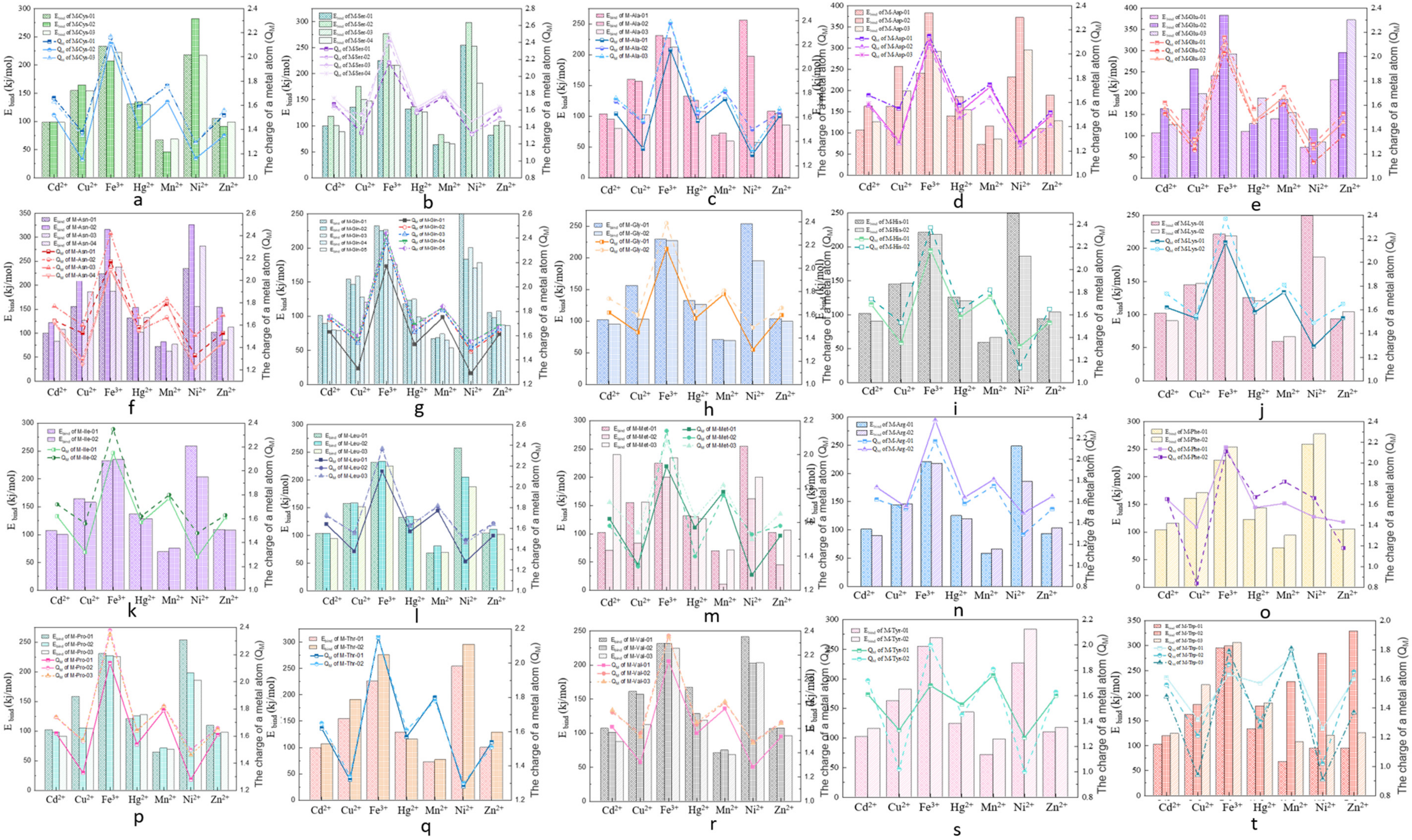

Figure S4. The 20 types of [AA-metal cation] complexes binding energy and Mulliken charge of metal cations.

TABLES

Table S1. The relative Gibbs free energy (Δ*G*) of AAs-metal cations structures.

Complexes	Δ*G* (kJ/mol)	Complexes	Δ*G* (kJ/mol)	Complexes	Δ*G* (kJ/mol)
Cd-Asp-01	44.78	Fe-Glu-02	59.91	Cd-His-01	0
Cd-Asp-02	0	Fe-Glu-03	0	Cd-His-02	1.47
Cd-Asp-03	24.04	Hg-Glu-01	60.01	Cu-His-01	0
Cu-Asp-01	77.33	Hg-Glu-02	0	Cu-His-02	10.63
Cu-Asp-02	0	Hg-Glu-03	35.5	Fe-His-01	0
Cu-Asp-03	49.21	Mn-Glu-01	50.78	Fe-His-02	13.17
Fe-Asp-01	127.56	Mn-Glu-02	0	Mn-His-01	0
Fe-Asp-02	0	Mn-Glu-03	31.83	Mn-His-02	17.45
Fe-Asp-03	81.74	Ni-Glu-01	135.48	Ni-His-01	0
Mn-Asp-01	39.8	Ni-Glu-02	0	Ni-His-02	0.63
Mn-Asp-02	0	Ni-Glu-03	87.12	Zn-His-01	0
Mn-Asp-03	26.54	Zn-Glu-01	85.69	Zn-His-02	11.45
Ni-Asp-01	132.83	Zn-Glu-02	0	Hg-His-01	0
Ni-Asp-02	0	Zn-Glu-03	55.18	Hg-His-02	2.19
Asp-Ni-03	68.42	Cd-Arg-01	0	Cd-Lys-01	0
Zn-Asp-01	64.76	Cd-Arg-02	2.87	Cd-Lys-02	3.81
Zn-Asp-02	0	Cu-Arg-01	0	Cu-Lys-01	0
Zn-Asp-03	44.9	Cu-Arg-02	0.19	Cu-Lys-02	2.8
Hg-Asp-01	32.8	Fe-Arg-01	0	Fe-Lys-01	0
Hg-Asp-02	0	Fe-Arg-02	2.19	Fe-Lys-02	4.77
Cd-Asp-03	15.68	Mn-Arg-01	0	Mn-Lys-01	0
Cd-Glu-01	57.1	Mn-Arg-02	8.95	Mn-Lys-02	11.31
Cd-Glu-02	0	Ni-Arg-01	0	Ni-Lys-01	0
Cd-Glu-03	28.96	Ni-Arg-02	63.13	Ni-Lys-02	52.44
Cu-Glu-01	102.2	Zn-Arg-01	0	Zn-Lys-01	0
Cu-Glu-02	0	Zn-Arg-02	8.02	Zn-Lys-02	1.7
Cu-Glu-03	57.54	Hg-Arg-01	0	Hg-Lys-01	0
Fe-Glu-01	59.91	Hg-Arg-02	6.77	Hg-Lys-02	1.53
Cd-Cys-01	10.2	Mn-Ser-01	22.75	Hg-Trp-01	23.84
Cd-Cys-02	17.03	Mn-Ser-02	0	Hg-Trp-02	0
Cd-Cys-03	0	Mn-Ser-03	7.5	Hg-Trp-03	4.43
Cu-Cys-01	9.11	Mn-Ser-04	149.77	Cd-Phe-01	1.96
Cu-Cys-02	3.6	Ni-Ser-01	44.17	Cd-Phe-02	0
Cu-Cys-03	0	Ni-Ser-02	0	Cu-Phe-01	3.12
Fe-Cys-01	0.94	Ni-Ser-03	32.82	Cu-Phe-02	0
Fe-Cys-02	32.49	Ni-Ser-04	117.11	Cu-Phe-01	3.12

Table S1. continuous: The relative Gibbs free energy (Δ*G*) of AAs-metal cations structures.

Fe-Cys-03	0	Zn-Ser-01	12.41	Cu-Phe-02	0
Mn-Cys-01	13.97	Zn-Ser-02	0	Fe-Phe-01	19.89
Mn-Cys-02	42.32	Zn-Ser-03	7.63	Fe-Phe-02	0
Mn-Cys-03	0	Zn-Ser-04	37.22	Mn-Phe-01	16.85
Ni-Cys-01	64.19	Hg-Ser-01	11.18	Mn-Phe-02	0
Ni-Cys-02	0	Hg-Ser-02	0	Ni-Phe-01	12.96
Ni-Cys-03	54.02	Hg-Ser-03	6.38	Ni-Phe-02	0
Zn-Cys-01	6.48	Hg-Ser-04	38.38	Zn-Phe-01	0
Zn-Cys-02	28.76	Cd-Trp-01	7.63	Zn-Phe-02	10.05
Zn-Cys-03	0	Cd-Trp-02	0	Hg-Phe-01	5.8
Hg-Cys-01	10.99	Cd-Trp-03	6	Hg-Phe-02	0
Hg-Cys-02	12.17	Cu-Trp-01	32.57	Cd-Tyr-01	2.68
Hg-Cys-03	0	Cu-Trp-02	19.73	Cd-Tyr-02	0
Cd-Ser-01	12.63	Cu-Trp-03	0	Cu-Tyr-01	11.14
Cd-Ser-02	0	Fe-Trp-01	15.79	Cu-Tyr-02	0
Cd-Ser-03	3.15	Fe-Trp-02	10	Fe-Tyr-01	14.94
Cd-Ser-04	33.96	Fe-Trp-03	0	Fe-Tyr-02	0
Cu-Ser-01	36.73	Mn-Trp-01	24.28	Mn-Tyr-01	21.48
Cu-Ser-02	0	Mn-Trp-02	0	Mn-Tyr-02	0
Cu-Ser-03	25.66	Mn-Trp-03	1.92	Ni-Tyr-01	55.15
Cu-Ser-04	48.99	Ni-Trp-01	74.58	Ni-Tyr-02	0
Fe-Ser-01	42.17	Ni-Trp-02	29.86	Zn-Tyr-01	0
Fe-Ser-02	0	Ni-Trp-03	0	Zn-Tyr-02	0.1
Fe-Ser-03	33.65	Zn-Trp-01	1.68	Hg-Tyr-01	1.51
Fe-Ser-04	54.65	Zn-Trp-02	0	Hg-Tyr-02	0
Fe-Ser-04	54.65	Zn-Trp-03	12.94	Cd-Asn-01	14.68
Cd-Asn-02	0	Cu-Gln-04	27	Fe-Thr-01	32.44
Cd-Asn-03	33.53	Cu-Gln-05	31.04	Cd-Thr-01	0
Cd-Asn-04	15.39	Cd-Gln-02	15.88	Cd-Thr-02	2.82
Cu-Asn-01	49.45	Cd-Gln-03	4.03	Cu-Thr-01	21.14
Cu-Asn-02	0	Cd-Gln-04	21.66	Cu-Thr-02	0
Cu-Asn-03	79.6	Cd-Gln-05	24.46	Fe-Thr-02	0
Cu-Asn-04	43.62	Cu-Gln-01	4.88	Hg-Thr-01	0
Fe-Asn-01	86.86	Cu-Gln-02	15.97	Hg-Thr-02	19.32
Fe-Asn-02	0	Cu-Gln-03	0	Mn-Thr-01	0.25
Fe-Asn-03	117.58	Cu-Gln-04	27	Mn-Thr-02	0
Fe-Asn-04	91.08	Cu-Gln-05	31.04	Ni-Thr-01	27.36
Hg-Asn-01	13.7	Fe-Gln-01	2.08	Ni-Thr-02	0
Hg-Asn-02	0	Fe-Gln-02	7.08	Zn-Thr-01	9.49
Hg-Asn-03	44.1	Fe-Gln-03	0	Zn-Thr-02	0
Hg-Asn-04	22.69	Fe-Gln-04	39.44	Cd-Met-01	0

Table S1. continuous: The relative Gibbs free energy (Δ*G*) of AAs-metal cations structures.

Mn-Asn-01	6.28	Fe-Gln-05	42.75	Cd-Met-02	7.47
Mn-Asn-02	0	Hg-Gln-01	1.43	Cd-Met-03	24.71
Mn-Asn-03	19.32	Hg-Gln-02	12.3	Cu-Met-01	0
Mn-Asn-04	3.2	Hg-Gln-03	0	Cu-Met-02	3.48
Ni-Asn-01	89.07	Hg-Gln-04	21.93	Cu-Met-03	53.55
Ni-Asn-02	0	Hg-Gln-05	22.93	Fe-Met-01	0.48
Ni-Asn-03	164.21	Mn-Gln-01	8.06	Fe-Met-02	0
Ni-Asn-04	60.91	Mn-Gln-02	8.59	Fe-Met-03	14.82
Zn-Asn-01	47.01	Mn-Gln-03	0	Hg-Met-01	0
Zn-Asn-02	0	Mn-Gln-04	13.87	Hg-Met-02	10.1
Zn-Asn-03	59.14	Mn-Gln-05	19.59	Hg-Met-03	38.38
Zn-Asn-04	47.69	Ni-Gln-01	0	Mn-Met-01	4.11
Cd-Gln-01	0	Ni-Gln-02	70.24	Mn-Met-02	0
Cd-Gln-02	15.88	Ni-Gln-03	52.12	Mn-Met-03	14.19
Cd-Gln-03	4.03	Ni-Gln-04	78.53	Ni-Met-01	0
Cd-Gln-04	21.66	Ni-Gln-05	66.6	Ni-Met-02	81.78
Cd-Gln-05	24.46	Zn-Gln-01	0.76	Ni-Met-03	52.19
Cu-Gln-01	4.88	Zn-Gln-02	13.71	Zn-Met-01	0
Cu-Gln-02	15.97	Zn-Gln-03	0	Zn-Met-02	5.41
Cu-Gln-03	0	Zn-Gln-04	18.35	Zn-Met-03	25.96
Cd-Gln-01	0	Zn-Gln-05	21.53		
Cd-Val-01	0	Cu-Ala-01	0	Fe-Leu-01	5.86
Cd-Val-02	6.13	Cu-Ala-02	3.48	Fe-Leu-02	0
Cd-Val-03	18.58	Cu-Ala-03	53.55	Fe-Leu-03	15.01
Cu-Val-01	1.39	Fe-Ala-01	0.48	Hg-Leu-01	0
Cu-Val-02	0	Fe-Ala-02	0	Hg-Leu-02	3.67
Cu-Val-03	44.53	Fe-Ala-03	14.82	Hg-Leu-03	17.09
Fe-Val-01	4.14	Hg-Ala-01	0	Mn-Leu-01	11.2
Fe-Val-02	0	Hg-Ala-02	10.1	Mn-Leu-02	0
Fe-Val-03	10.09	Hg-Ala-03	38.38	Mn-Leu-03	12.51
Hg-Val-01	0	Mn-Ala-01	4.11	Ni-Leu-01	0
Hg-Val-02	10.72	Mn-Ala-02	0	Ni-Leu-02	52.14
Hg-Val-03	23.89	Mn-Ala-03	14.19	Ni-Leu-03	73.19
Mn-Val-01	8.3	Ni-Ala-01	0	Zn-Leu-01	5.9
Mn-Val-02	0	Ni-Ala-02	56.55	Zn-Leu-02	0
Mn-Val-03	8.45	Ni-Ala-03	218.72	Zn-Leu-03	12.55
Ni-Val-01	0	Zn-Ala-01	0	Cd-Pro-01	0
Ni-Val-02	37.15	Zn-Ala-02	5.41	Cd-Pro-02	5.48
Ni-Val-03	38.5	Zn-Ala-03	25.96	Cd-Pro-03	15.41
Zn-Val-01	2.97	Cd-Gly-01	0	Cu-Pro-01	0
Zn-Val-02	0	Cd-Gly-02	11.73	Cu-Pro-02	44.59

Table S1. continuous: The relative Gibbs free energy (Δ*G*) of AAs-metal cations structures.

Zn-Val-03	12.45	Cu-Gly-01	0	Cu-Pro-03	51
Cd-Ile-01	0	Cu-Gly-02	51.2	Fe-Pro-01	4
Cd-Ile-02	4.61	Fe-Gly-01	1.66	Fe-Pro-02	0
Cu-Ile-01	0	Fe-Gly-02	0	Fe-Pro-03	12.48
Cu-Ile-02	0.44	Hg-Gly-01	0	Hg-Pro-01	0.84
Fe-Ile-01	6.12	Hg-Gly-02	12.22	Hg-Pro-02	0
Fe-Ile-02	0	Mn-Gly-01	1.11	Hg-Pro-03	10.51
Hg-Ile-01	0	Mn-Gly-02	0	Mn-Pro-01	7.48
Hg-Ile-02	13.77	Ni-Gly-01	0	Mn-Pro-02	0
Mn-Ile-01	6.87	Ni-Gly-02	56.87	Mn-Pro-03	6.56
Mn-Ile-02	0	Zn-Gly-01	0	Ni-Pro-01	0
Ni-Ile-01	0	Zn-Gly-02	5.25	Ni-Pro-02	53.08
Ni-Ile-02	55.89	Cd-Leu-01	0	Ni-Pro-03	74.43
Zn-Ile-01	0	Cd-Leu-02	3.89	Zn-Pro-01	0
Zn-Ile-02	2.34	Cd-Leu-03	15.82	Zn-Pro-02	15.13
Cd-Ala-01	0	Cu-Leu-01	5.97	Zn-Pro-03	19.37
Cd-Ala-02	7.47	Cu-Leu-02	0		
Cd-Ala-03	24.71	Cu-Leu-03	15.44		

Text S1. The cartesian coordinates information of converged conformations.

Molecule: Cd-ASP-01

N	-1.39007700	2.52559900	-0.09583400
H	-1.04251200	2.59623700	-1.05494900
H	-0.70411200	2.97219500	0.51554200
H	-2.27329500	3.03624200	-0.03171000
C	-1.59575000	1.09106100	0.29054900
H	-1.97964300	1.09673300	1.30846500
C	-2.60710500	0.44278500	-0.64604700
C	-0.25630000	0.35963800	0.28592200
H	-3.44555000	1.13292500	-0.77788900
H	-2.16039900	0.27043800	-1.62477100
C	-3.20483900	-0.86306500	-0.09690100
O	0.76765100	1.04857400	-0.05549400
O	-3.50616200	-0.89462000	1.12435100
O	-3.39779600	-1.78812700	-0.92798000
O	-0.23443600	-0.83735500	0.59643600
Cd	2.46429100	-0.31654700	-0.05340600

Molecule: Cd-ASP-02

N	-3.07254100	0.08442500	-0.67982400
H	-3.63720200	-0.19992700	0.12473400
H	-3.30382200	-0.54537400	-1.45092600
H	-3.32365500	1.03874700	-0.94606000
C	-1.61983100	-0.00930200	-0.34020100
H	-1.07810900	0.14371700	-1.27135300
C	-1.24674800	1.07805500	0.69149200
C	-1.33172900	-1.43245700	0.15886100
H	-2.13930000	1.62237300	0.99226400
H	-0.83638600	0.60642600	1.58576000
C	-0.20662200	2.07746800	0.18519400
O	-2.29084400	-2.17168300	0.41653400
O	-0.48470400	3.28680700	0.20151700
O	0.93025400	1.63447700	-0.22476900
O	-0.10697600	-1.76441000	0.28827700
Cd	1.62237500	-0.44627200	-0.08125400

Molecule: Cd-ASP-03

N	3.72365600	-1.23728800	-0.35361500
H	3.92370700	-1.63021100	0.56845600
H	4.46678800	-0.55941100	-0.57146900
H	3.71580200	-1.99751000	-1.03373300
C	2.43691000	-0.47091700	-0.35165300
H	2.06963400	-0.48652000	-1.37646600
C	1.42835400	-1.12808300	0.57710600
C	2.75297300	0.99994600	0.00139000
H	1.40028700	-2.20298700	0.37569500
H	1.71290700	-0.98592900	1.61819200
C	0.01858000	-0.62683800	0.36167500
O	3.95518500	1.35221400	-0.09952600
O	-0.41434500	-0.51155200	-0.82660100
O	-0.71587200	-0.39374900	1.36010800
O	1.77493900	1.70978900	0.32149900
Cd	-2.49947700	0.13802800	-0.13917300

Molecule: Cu-ASP-01

N	-1.73196800	2.44169200	-0.00112700
H	-1.60680200	2.65768400	-0.99334000
H	-1.18380000	3.12035300	0.53198600
H	-2.72078200	2.55551200	0.23205100
C	-1.28938900	1.04390400	0.29792400
H	-1.49136200	0.87065700	1.35350100
C	-2.06615500	0.04970800	-0.55394900
C	0.21561900	0.96933500	0.07309000
H	-3.12399500	0.32562800	-0.52706500
H	-1.73262500	0.09944500	-1.58990400
C	-1.97974900	-1.39606500	-0.03495900
O	0.86631800	1.96083600	-0.24858000
O	-2.10376200	-1.57232500	1.20470700
O	-1.84135600	-2.30009500	-0.89890900
O	0.69912900	-0.21437700	0.25438600
Cu	2.54270900	-0.47292200	-0.00637300

Molecule: Cu-ASP-02

N	2.57519700	1.17858300	0.57226500
H	3.08129800	1.27253200	-0.31200900
H	3.15200400	0.61020500	1.19624100
H	2.45970400	2.10736800	0.98367000
C	1.24504500	0.53441600	0.35660600
H	0.77334800	0.47368000	1.33565400
C	0.40480800	1.39768900	-0.60147900
C	1.47850200	-0.88671400	-0.15898300
H	0.86491300	2.37787600	-0.70866300
H	0.37925700	0.94229000	-1.59362000
C	-1.03050400	1.62069700	-0.15567100
O	2.62594600	-1.25505500	-0.41356200
O	-1.71600300	0.61165000	0.27344400
O	-1.51994100	2.75115600	-0.21945900
O	0.44268500	-1.63001000	-0.30969800
Cu	-1.37881000	-1.23629300	0.13118100

Molecule: Cu-ASP-03

N	3.16173100	-1.26708800	-0.20577600
H	3.32362200	-1.53876200	0.76690400
H	3.92112300	-0.63064200	-0.47731200
H	3.17274500	-2.10869700	-0.78374000
C	1.88414300	-0.50290000	-0.34161700
H	1.60610900	-0.55162500	-1.39356000
C	0.80566900	-1.13370600	0.52566100
C	2.16243400	0.97948500	-0.00448000
H	0.73629100	-2.20318200	0.30412100
H	1.04192600	-1.01919300	1.58281300
C	-0.55107700	-0.56796900	0.26207900
O	3.35987600	1.35017100	-0.06677300
O	-1.00160400	-0.43487900	-0.92343200
O	-1.34368300	-0.27392400	1.21124200
O	1.15730900	1.67193600	0.26600600
Cu	-2.72814200	0.19882100	-0.17603400

Molecule: Fe-ASP-01

N	-0.66886600	2.54640500	0.11688700
H	-0.48495300	2.76277100	-0.86717900
H	0.10740900	2.91597500	0.67271800
H	-1.52522300	3.03122500	0.39697300
C	-0.83959300	1.07496100	0.33005200
H	-1.07803400	0.92475900	1.38292800
C	-1.97253600	0.52657400	-0.52947000
C	0.47886400	0.41013100	0.07933600
H	-2.79232900	1.24899400	-0.48871900
H	-1.66413400	0.43934400	-1.57020600
C	-2.54438600	-0.81074000	-0.02659100
O	1.49247900	1.05642800	-0.31651400
O	-2.36894000	-1.10871500	1.18109100
O	-3.18863500	-1.47891000	-0.87341700
O	0.66775800	-0.83103400	0.30341600
Fe	2.63707400	-0.67137000	-0.07004900

Molecule: Fe-ASP-02

N	-2.61532500	1.15236100	-0.41043500
H	-3.07387400	1.13609800	0.50513100
H	-3.20947200	0.63259800	-1.06064900
H	-2.55018300	2.12311400	-0.72545500
C	-1.25876100	0.53549100	-0.32940300
H	-0.84915500	0.55381800	-1.33855700
C	-0.37385000	1.34864000	0.63712800
C	-1.43991800	-0.92439500	0.07743000
H	-0.83272000	2.31675300	0.82413400
H	-0.28853200	0.83216900	1.59516300
C	1.03034900	1.60785500	0.12122600
O	-2.55132600	-1.38095800	0.28284900
O	1.48390800	2.73827100	0.06514200
O	1.76373700	0.57353100	-0.24871400
O	-0.35654100	-1.63922200	0.18361100
Fe	1.48638700	-1.28460000	-0.08569700

Molecule: Fe-ASP-03

N	3.18045400	-1.09559300	-0.19659600
H	3.35269200	-1.36089800	0.77635700
H	3.86617800	-0.37098600	-0.44314100
H	3.30592300	-1.91895500	-0.78730100
C	1.82846300	-0.48629700	-0.35875300
H	1.58928400	-0.54231000	-1.41990200
C	0.79278500	-1.25080800	0.45897300
C	1.90714700	1.01111800	0.01734800
H	0.76866800	-2.29910200	0.14667500
H	1.02184100	-1.20659400	1.52295300
C	-0.56809400	-0.70201700	0.23028900
O	3.04327400	1.53729000	-0.00700500
O	-1.05286600	-0.54565400	-0.94389600
O	-1.37138200	-0.39790300	1.17515600
O	0.80987000	1.55389700	0.27483000
Fe	-2.74464400	0.25980700	-0.17305000

Molecule: Hg-ASP-01

N	-2.91002100	2.35707000	0.01971500
H	-2.86920300	2.58013800	-0.97813700
H	-2.36833300	3.06822800	0.51581500
H	-3.88543200	2.40846600	0.32123200
C	-2.36092600	0.98971700	0.28493700
H	-2.50274100	0.79901300	1.34769900
C	-3.11109300	-0.05079500	-0.53148800
C	-0.85365400	1.02283400	0.02390600
H	-4.18194600	0.15753700	-0.45104000
H	-2.83411700	0.01737400	-1.58269300
C	-2.91094300	-1.48649100	-0.01663200
O	-0.27974800	2.07938600	-0.22024100
O	-2.95568500	-1.66366700	1.22848100
O	-2.76892000	-2.38542000	-0.88522200
O	-0.31570600	-0.14405000	0.12380900
Hg	1.81240100	-0.14339800	0.00187700

Molecule: Hg-ASP-02

N	3.28485100	-0.07449800	0.76685500
H	3.90372900	-0.37110300	0.00813100
H	3.43518300	-0.71149000	1.55247000
H	3.54273400	0.87127300	1.05831400
C	1.86054800	-0.11664700	0.32110800
H	1.26314900	0.05222900	1.21573100
C	1.59437200	0.97922700	-0.73096300
C	1.53853600	-1.52482200	-0.19690700
H	2.53906100	1.39518200	-1.07378800
H	1.09971200	0.53587600	-1.59565700
C	0.71015500	2.11949500	-0.23122600
O	2.46240800	-2.32534200	-0.36993100
O	-0.51203500	1.86137700	0.12077900
O	1.16143600	3.26920200	-0.19531500
O	0.30625500	-1.78836900	-0.43429800
Hg	-1.25429600	-0.22661200	0.06906100

Molecule: Hg-ASP-031

N	4.45091800	-1.20055000	0.03125300
H	4.52760700	-1.43556000	1.02321700
H	5.20974900	-0.54245800	-0.18905300
H	4.54653900	-2.05910700	-0.51215300
C	3.16725200	-0.48958600	-0.26446500
H	2.97126600	-0.64171600	-1.32479000
C	2.03065500	-1.06810100	0.56394900
C	3.38936900	1.02396400	-0.04944200
H	2.06248000	-2.16000000	0.51372400
H	2.12112800	-0.77168900	1.60683100
C	0.67439400	-0.67306400	0.00788700
O	4.58289500	1.41482100	-0.00979100
O	0.43181100	-0.75710200	-1.19571300
O	-0.18612900	-0.30799900	0.90948300
O	2.35501600	1.72154300	0.03048200
Hg	-2.07042400	0.08356200	-0.00949800

Molecule: Mn-ASP-11

N	-0.88417300	2.55897100	-0.05689700
H	-0.83612200	2.71561000	-1.06654200
H	-0.04017700	2.96264200	0.35740100
H	-1.70837800	3.04189600	0.30499600
C	-0.95175700	1.09404700	0.25227200
H	-1.17397600	1.01705700	1.31624900
C	-2.05633400	0.43142100	-0.54999000
C	0.43322500	0.49281100	0.04616300
H	-2.94069800	1.07521100	-0.51790900
H	-1.76098100	0.32490500	-1.59298200
C	-2.49628600	-0.92716200	0.01683800
O	1.42216200	1.27151600	-0.01074400
O	-2.54001600	-1.05151800	1.26891200
O	-2.83517800	-1.80470400	-0.81940800
O	0.53523200	-0.76114200	-0.00417700
Mn	2.89675500	-0.67320100	-0.01885200

Molecule: Mn-ASP-21

N	2.64870500	0.30369400	1.04422000
H	3.26496200	0.38444900	0.22961400
H	2.94236000	-0.52351400	1.56729600
H	2.76157100	1.12553800	1.64125200
C	1.23549300	0.16134900	0.57274400
H	0.64465600	-0.16331900	1.42569200
C	0.75291100	1.51449400	0.05684400
C	1.21537400	-0.91442100	-0.52650400
H	1.00917300	2.27636700	0.79940500
H	1.28202700	1.77470700	-0.86024300
C	-0.74880200	1.68143600	-0.18440200
O	2.27141900	-1.13856700	-1.13756800
O	-1.57021600	0.92442900	0.44174600
O	-1.09550000	2.61069000	-0.93139600
O	0.10016100	-1.48171300	-0.75323900
Mn	-1.71289800	-1.15903800	0.29676000

Molecule: Mn-ASP-31

N	3.13510900	-1.29435200	-0.21069500
H	3.30904100	-1.54029600	0.76663300
H	3.91739400	-0.70946200	-0.52369900
H	3.08589600	-2.15573500	-0.75624300
C	1.88414700	-0.47884000	-0.33492900
H	1.61843100	-0.48895700	-1.39119500
C	0.77510700	-1.10135400	0.49163800
C	2.22866700	0.98104800	0.03766100
H	0.73352500	-2.17602900	0.28733400
H	0.97245800	-0.97604900	1.55554900
C	-0.60309400	-0.56501400	0.16328400
O	3.43792900	1.30834500	-0.06916500
O	-0.90112700	-0.27222500	-1.02316400
O	-1.44475400	-0.49208900	1.11385200
O	1.27189600	1.71138200	0.37611900
Mn	-3.20812100	0.24194600	-0.15162300

Molecule: Ni-ASP-01

N	-0.69855700	2.57684300	-0.02415500
H	-0.44670700	2.71480600	-1.00706100
H	0.03781100	2.99203300	0.55208700
H	-1.57270000	3.07714900	0.15380400
C	-0.87635600	1.12444300	0.28547700
H	-1.15851200	1.05314000	1.33572600
C	-1.96850700	0.50834900	-0.57835800
C	0.44913300	0.43309100	0.12802500
H	-2.80916400	1.20694700	-0.60814200
H	-1.61707500	0.36677100	-1.59916300
C	-2.52290700	-0.81350500	-0.01580700
O	1.51607300	1.08189300	-0.10882600
O	-2.54737300	-0.95213900	1.23333900
O	-2.95733000	-1.63473300	-0.86234100
O	0.53901100	-0.82873400	0.24234300
Ni	2.48446500	-0.65333300	-0.05751100

Molecule: Ni-ASP-02

N	-2.42586700	1.42478400	-0.47571000
H	-2.91577600	1.48485300	0.42088400
H	-3.05850000	0.97335700	-1.13949800
H	-2.22252500	2.37183100	-0.80285400
C	-1.16355000	0.63735500	-0.33141200
H	-0.72665900	0.57866100	-1.32759400
C	-0.21217900	1.36549500	0.64094700
C	-1.55119600	-0.77816300	0.10499700
H	-0.58528400	2.36971100	0.82848500
H	-0.18240700	0.84365000	1.59965600
C	1.21662800	1.50058400	0.13563900
O	-2.72763700	-1.04206700	0.33540400
O	1.76380000	2.60133800	0.12143800
O	1.83451100	0.42687600	-0.26070700
O	-0.59758500	-1.63882800	0.20385500
Ni	1.24104600	-1.34734800	-0.09821600

Molecule: Ni-ASP-03

N	3.14613400	-1.24548600	-0.19311500
H	3.30381700	-1.51195600	0.78180200
H	3.89730300	-0.59691000	-0.45898000
H	3.17752600	-2.08788600	-0.76928900
C	1.85807100	-0.50337400	-0.34396000
H	1.59577200	-0.55205300	-1.39996500
C	0.77654900	-1.15380200	0.50483400
C	2.10559400	0.98235900	0.00384100
H	0.70891300	-2.21912700	0.26382200
H	1.00388200	-1.05709600	1.56541400
C	-0.57589300	-0.58146800	0.23880700
O	3.29536300	1.37706300	-0.05275000
O	-1.01478500	-0.41838400	-0.94820100
O	-1.37873200	-0.30453100	1.18968100
O	1.08586600	1.65241700	0.27620500
Ni	-2.73563500	0.20816500	-0.17041400

Molecule: Zn-ASP-01

N	-0.88555400	2.57300900	-0.02885300
H	-0.55846200	2.67838300	-0.99249900
H	-0.20409800	3.02862700	0.58170200
H	-1.78335700	3.05334100	0.06319700
C	-1.03639800	1.12411500	0.32187800
H	-1.36928800	1.08217500	1.35615800
C	-2.06964000	0.47334700	-0.59153300
C	0.31830200	0.44980200	0.21229600
H	-2.94956900	1.12181500	-0.62903100
H	-1.67384200	0.38532400	-1.60277200
C	-2.54991000	-0.89778600	-0.08645900
O	1.29123100	1.09620500	-0.28066800
O	-2.81141400	-1.00306100	1.13942600
O	-2.68910600	-1.79976800	-0.95225500
O	0.44346600	-0.74023600	0.58951300
Zn	2.56299800	-0.55609100	-0.05600000

Molecule: Zn-ASP-02

N	-2.50686200	1.28826800	-0.54690300
H	-3.04040600	1.34143900	0.32426500
H	-3.08645200	0.79710300	-1.23051700
H	-2.33067700	2.23726100	-0.88354600
C	-1.22049100	0.56051500	-0.33132700
H	-0.73954000	0.50184100	-1.30623500
C	-0.33691000	1.34331200	0.66387800
C	-1.54596600	-0.86933500	0.12779000
H	-0.81122700	2.29325400	0.90130700
H	-0.24443200	0.78276900	1.59564700
C	1.07302800	1.63432700	0.15270100
O	-2.72341800	-1.15960800	0.36134300
O	1.80867000	0.65120000	-0.24467400
O	1.47792400	2.80394500	0.15227100
O	-0.56793900	-1.68760100	0.24394600
Zn	1.33403000	-1.26159800	-0.11179800

Molecule: Zn-ASP-03

N	3.23357700	-1.16955100	-0.32514700
H	3.42793200	-1.54978500	0.60348300
H	3.94385100	-0.45109400	-0.52197200
H	3.28769700	-1.92926800	-1.00377200
C	1.90970000	-0.47247800	-0.35972200
H	1.58181000	-0.48601400	-1.39784300
C	0.90415500	-1.20531200	0.51762400
C	2.14013500	1.00752800	0.02801200
H	0.89616900	-2.26460200	0.24627800
H	1.18016500	-1.12381900	1.56773700
C	-0.50090100	-0.69443900	0.32396100
O	3.32629700	1.41941400	-0.03054400
O	-0.94801900	-0.55270800	-0.86312600
O	-1.23528400	-0.43905600	1.31497800
O	1.12031400	1.66538800	0.32616800
Zn	-2.72592200	0.24784400	-0.20856500

Molecule:Cd-GLU-01

N	-0.75231400	2.98471200	-0.02076600
H	-0.50858700	2.92445800	-1.01301800
H	-0.00822700	3.50188400	0.45269500
H	-1.62516500	3.50905000	0.07199400
C	-0.91203100	1.60914100	0.55255100
H	-1.08196300	1.72673600	1.61986700
C	-2.10928400	0.93613000	-0.12292700
C	0.37929700	0.83962600	0.32297700
H	-2.95766100	1.61637200	-0.02546500
H	-1.89591200	0.83291300	-1.18870400
C	-2.46589500	-0.41194900	0.48344100
O	1.10219300	1.15198600	-0.66005500
H	-1.63805600	-1.11575200	0.37854100
H	-2.64254500	-0.30936600	1.55696500
C	-3.70075600	-1.08128500	-0.12886200
O	-4.06242500	-2.16779900	0.40551500
O	-4.27016100	-0.53456100	-1.11084900
O	0.65467100	-0.11067700	1.10412500
Cd	2.56421100	-0.65918500	-0.13042700

Molecule:Cd-GLU-02

N	-4.04942900	1.66677800	-0.31108600
H	-4.19527900	1.73893100	0.70235600
H	-4.96976400	1.62738600	-0.75160000
H	-3.55811300	2.49289100	-0.65642400
C	-3.27604900	0.40765300	-0.56782300
H	-3.43170600	0.13905200	-1.60949800
C	-1.80040000	0.68462800	-0.28635800
C	-3.84899400	-0.68703200	0.35701000
H	-1.50556900	1.57077700	-0.85250800
H	-1.68104700	0.92071500	0.77277700
C	-0.89945600	-0.47961000	-0.66988700
O	-4.34535500	-0.29065100	1.44235600
H	-1.17427300	-1.38560900	-0.12621100
H	-1.00128300	-0.71996100	-1.73024600
C	0.56629300	-0.24459600	-0.39327900
O	1.39730300	-1.12778800	-0.76733500
O	0.94702400	0.79824600	0.21527100
O	-3.73742600	-1.86894400	-0.03880800
Cd	3.15254800	0.07865000	0.18706500

Molecule:Cd-GLU-03

N	2.88733800	0.83575400	-1.26547300
H	2.47032500	0.11200600	-1.85760900
H	3.83058900	0.53603300	-1.00820000
H	2.96468700	1.69656600	-1.81304000
C	2.03298700	1.05632400	-0.05032100
H	2.57730400	1.73832800	0.59665100
C	0.70379400	1.65294700	-0.50766300
C	1.86674800	-0.29848200	0.66996800
H	0.90056000	2.60631900	-0.99958100
H	0.28259600	0.98394900	-1.26128300
C	-0.30206500	1.84968800	0.64067500
O	1.37386400	-1.25222500	-0.03837800
H	-0.10714600	1.13333600	1.44218400
H	-0.21089500	2.84878800	1.05951500
C	-1.74034600	1.62321900	0.18792200
O	-2.52714200	2.58089000	0.12547900
O	-2.11076900	0.42116700	-0.11527600
O	2.20168200	-0.37851800	1.85296800
Cd	-0.82889900	-1.32871500	-0.15712800

Molecule:Cu-GLU-01

N	0.15095600	2.85017100	0.01241200
H	0.30188800	2.82034600	-1.00001000
H	0.99097000	3.23738300	0.45049300
H	-0.63021100	3.48368100	0.20225700
C	-0.16995800	1.48590600	0.54239300
H	-0.26040400	1.57414200	1.62222200
C	-1.48257400	1.00527500	-0.08097300
C	0.97617100	0.55718000	0.22988800
H	-2.24819500	1.73286000	0.19085400
H	-1.37930300	1.02370500	-1.16778100
C	-1.89532100	-0.38101400	0.38997900
O	1.61016600	0.62120300	-0.86063300
H	-1.17912300	-1.13542200	0.05847500
H	-1.89942800	-0.42480000	1.48239500
C	-3.27992200	-0.83347400	-0.09047300
O	-3.61089200	-2.01184300	0.22079200
O	-3.99365700	-0.02944200	-0.74654200
O	1.30019600	-0.34376500	1.06421900
Cu	2.68656000	-1.00536400	-0.18253000

Molecule:Cu-GLU-02

N	-3.43178600	1.66785300	-0.23174900
H	-3.62037900	1.66270400	0.77680300
H	-4.33249600	1.63488000	-0.71235300
H	-2.95306700	2.53065400	-0.49545700
C	-2.61309700	0.45424300	-0.55067800
H	-2.70319300	0.27630000	-1.61918700
C	-1.16115900	0.73550600	-0.16839400
C	-3.20666600	-0.73401500	0.23628700
H	-0.84441900	1.64830900	-0.67667100
H	-1.10313000	0.91991200	0.90639500
C	-0.23792800	-0.41023500	-0.55683600
O	-3.76536300	-0.45513800	1.32682900
H	-0.51693700	-1.33994500	-0.05866700
H	-0.29673200	-0.61372700	-1.63001200
C	1.20075200	-0.17265100	-0.24638100
O	2.05705000	-1.10385700	-0.43843600
O	1.65086700	0.92522400	0.20924400
O	-3.05149100	-1.86813500	-0.27030600
Cu	3.49561800	0.08221000	0.21478500

Molecule:Cu-GLU-03

N	2.81876800	1.54507600	-0.56619200
H	2.68787300	1.39580400	-1.56994300
H	3.67596300	1.05827000	-0.28598900
H	2.91500200	2.54773600	-0.39844700
C	1.67067600	0.97694400	0.21118100
H	1.79090900	1.35341500	1.22831600
C	0.34332700	1.44072200	-0.36724900
C	1.90297500	-0.54390000	0.28891100
H	0.46163500	2.44716200	-0.76790100
H	0.07493800	0.78946100	-1.20023100
C	-0.76709800	1.45372700	0.70231200
O	0.89970200	-1.31863100	0.44538700
H	-0.52200800	0.75118600	1.50033400
H	-0.85959400	2.44517900	1.13949900
C	-2.10473400	1.03298000	0.13813000
O	-3.04504900	1.82433600	0.03096500
O	-2.22231400	-0.20932600	-0.22861300
O	3.07330800	-0.93910200	0.26124200
Cu	-0.89214300	-1.53878600	-0.19289200

Molecule:Fe-GLU-01

N	0.26994100	2.76556000	0.06718500
H	0.43836500	2.77627800	-0.94336400
H	1.10862400	3.12136200	0.53397900
H	-0.50483400	3.40391800	0.26766600
C	-0.08856700	1.39234300	0.53936400
H	-0.24018900	1.44983800	1.61702200
C	-1.37379400	0.92438100	-0.16022800
C	1.06693600	0.47591000	0.27958500
H	-2.08568500	1.74646000	-0.07522200
H	-1.16620700	0.77933400	-1.22166300
C	-1.97005900	-0.33090500	0.45767800
O	2.05737700	0.80426200	-0.44650300
H	-1.30482400	-1.18565900	0.33192700
H	-2.09495200	-0.19498900	1.53509400
C	-3.33477400	-0.73143600	-0.11618200
O	-3.84077900	-1.78800800	0.35410300
O	-3.86152100	-0.00378400	-0.99852100
O	1.10087200	-0.70254800	0.75585800
Fe	2.86592600	-1.08140700	-0.22448200

Molecule:Fe-GLU-02

N	-3.28664900	1.67356200	-0.34655600
H	-3.48635700	1.72974300	0.65854200
H	-4.18382600	1.64189200	-0.83377000
H	-2.77869800	2.50509100	-0.65304800
C	-2.50573200	0.41777300	-0.58633500
H	-2.59882800	0.17481600	-1.64171500
C	-1.04869200	0.68425700	-0.21517300
C	-3.13176400	-0.69971600	0.27503100
H	-0.71282300	1.57080300	-0.75693700
H	-0.98701000	0.90669700	0.85171800
C	-0.13923200	-0.48765400	-0.56109400
O	-3.74724300	-0.32956400	1.30578800
H	-0.42054400	-1.39544100	-0.01947400
H	-0.18825100	-0.74275200	-1.62249600
C	1.28229600	-0.23867600	-0.23573600
O	2.21827900	-1.03527100	-0.60959100
O	1.68745400	0.76330200	0.44516000
O	-2.93937300	-1.87292000	-0.11596500
Fe	3.61033500	0.13976700	0.23768400

Molecule:Fe-GLU-03

N	2.60011000	0.99896900	-1.20479700
H	2.28072700	0.29052700	-1.87191900
H	3.55225900	0.76291800	-0.91258200
H	2.63133000	1.90055200	-1.68933700
C	1.66665300	1.08277700	-0.03163500
H	2.08785700	1.81730800	0.64914700
C	0.29214200	1.49812800	-0.53236900
C	1.67359100	-0.27652700	0.68599300
H	0.37834800	2.47330900	-1.01041800
H	-0.01432300	0.78542700	-1.30114600
C	-0.76018800	1.55085100	0.59180100
O	1.12872000	-1.27332100	0.02857300
H	-0.40937200	1.01787400	1.47978200
H	-0.96012900	2.57626200	0.88991400
C	-2.06823800	0.90497900	0.20500800
O	-3.13098600	1.50490400	0.21334000
O	-2.03532800	-0.37597800	-0.13047200
O	2.18655100	-0.39764500	1.78057900
Fe	-0.68318800	-1.64776500	-0.32495400

Molecule:Hg-GLU-01

N	-1.31719700	2.91676700	-0.25975500
H	-0.98494900	2.70285300	-1.20371500
H	-0.59865100	3.47121300	0.21121800
H	-2.16528900	3.48355700	-0.33682600
C	-1.61258600	1.65264100	0.48968100
H	-1.93009000	1.94525200	1.48681600
C	-2.72090200	0.89422700	-0.23849100
C	-0.33312200	0.82520200	0.62159900
H	-3.57972500	1.56442400	-0.30751300
H	-2.39005500	0.68107100	-1.25692800
C	-3.12830900	-0.39042600	0.46618200
O	0.43829600	0.87688700	-0.41210000
H	-2.30049300	-1.10258500	0.48399400
H	-3.37655200	-0.18838100	1.51130600
C	-4.32664700	-1.11010400	-0.16263100
O	-4.68536300	-2.18178400	0.40230300
O	-4.87322300	-0.61196800	-1.18235700
O	-0.13730800	0.16726500	1.63814400
Hg	2.16670500	-0.37759000	-0.11745000

Molecule:Hg-GLU-02

N	-4.70358200	1.69690500	-0.23192600
H	-4.85571000	1.71717000	0.78280600
H	-5.62082500	1.69024600	-0.68070900
H	-4.20192300	2.53541900	-0.52903800
C	-3.94141100	0.44536700	-0.55025100
H	-4.08671300	0.23731600	-1.60708600
C	-2.46716500	0.69381400	-0.23872300
C	-4.53597200	-0.69335900	0.30512600
H	-2.14793400	1.58087200	-0.78896100
H	-2.36329100	0.91417100	0.82571700
C	-1.57732800	-0.48071500	-0.61763400
O	-5.01813500	-0.35660500	1.41622300
H	-1.85540700	-1.38639500	-0.07815200
H	-1.67160800	-0.71634500	-1.68040500
C	-0.10883800	-0.24038200	-0.34779000
O	0.69828300	-1.19776100	-0.57188400
O	0.29225400	0.87060100	0.08736600
O	-4.45634600	-1.84833000	-0.17052800
Hg	2.54230400	0.04322000	0.09981900

Molecule:Hg-GLU-05

N	-3.22957400	0.11704500	1.28966100
H	-2.59727300	-0.43712800	1.87433900
H	-4.03615200	-0.46342300	1.04897000
H	-3.56129300	0.91476300	1.83736900
C	-2.50529100	0.58583200	0.06109300
H	-3.24078300	1.07661600	-0.56997200
C	-1.40352100	1.54913400	0.50382900
C	-1.95947100	-0.65408600	-0.67393000
H	-1.86785000	2.40388200	0.99688100
H	-0.79786200	1.03313100	1.24988600
C	-0.50734200	2.03169700	-0.65027800
O	-1.20775100	-1.43916100	0.02740100
H	-0.46959100	1.28243700	-1.44384900
H	-0.89715900	2.95133000	-1.07912800
C	0.92581900	2.27221600	-0.19673000
O	1.37193300	3.42278000	-0.12049400
O	1.68483700	1.25570200	0.10543600
O	-2.25793000	-0.81862100	-1.85552900
Hg	0.95056400	-0.79569100	0.09424300

Molecule:Mn-GLU-01

N	0.08575500	2.81756200	0.09225800
H	0.29942600	2.81562200	-0.90852300
H	0.89131800	3.21383400	0.58072500
H	-0.72494800	3.41752600	0.25935200
C	-0.19354900	1.41845400	0.55381800
H	-0.29780500	1.45847600	1.63519000
C	-1.49010200	0.94063500	-0.09739200
C	1.00817600	0.55107300	0.19879600
H	-2.26630200	1.66529200	0.15463900
H	-1.36764000	0.95920700	-1.18266900
C	-1.91697200	-0.44348400	0.36670400
O	1.69959600	0.85319200	-0.80508700
H	-1.20359500	-1.20035000	0.03548200
H	-1.92055500	-0.49041900	1.45896900
C	-3.30427800	-0.88603100	-0.11240500
O	-3.67113300	-2.03895800	0.25223700
O	-3.98913300	-0.10204200	-0.82244900
O	1.24339600	-0.44328600	0.94038500
Mn	3.16433400	-1.08789000	-0.18627100

Molecule:Mn-GLU-02

N	-3.25044900	1.74737400	-0.17561300
H	-3.45071700	1.70773100	0.83014100
H	-4.14555400	1.79427900	-0.66516200
H	-2.71393800	2.58869500	-0.39358500
C	-2.50478200	0.49832100	-0.53822400
H	-2.59601400	0.36621700	-1.61327800
C	-1.04399300	0.68212800	-0.13148300
C	-3.17781100	-0.67558400	0.20120500
H	-0.66723600	1.57973500	-0.62496500
H	-0.99790400	0.85827800	0.94609300
C	-0.16908500	-0.50671400	-0.50271600
O	-3.71600300	-0.40121300	1.30420600
H	-0.48809800	-1.41407400	0.00861800
H	-0.24735100	-0.71577000	-1.57440800
C	1.29934800	-0.31000100	-0.21178200
O	2.06027900	-1.31901600	-0.19049600
O	1.76656300	0.85605800	-0.01106900
O	-3.10305300	-1.79843600	-0.34683600
Mn	3.82302400	0.16700900	0.21469600

Molecule:Mn-GLU-031

N	-2.54920800	-1.18510400	-1.19995000
H	-2.28748600	-0.46158600	-1.87549900
H	-3.52603500	-1.04026400	-0.93466400
H	-2.47469200	-2.09549700	-1.66029400
C	-1.64130800	-1.12229900	-0.00358600
H	-2.01279900	-1.85416800	0.70813200
C	-0.22388000	-1.43648600	-0.45930100
C	-1.77347500	0.29010800	0.60591900
H	-0.20775800	-2.43007100	-0.90943900
H	0.03936400	-0.71756200	-1.23799200
C	0.79806800	-1.36053800	0.68847900
O	-1.35349100	1.25025300	-0.12717500
H	0.45907000	-0.65119400	1.44804400
H	0.91056700	-2.33058600	1.16714400
C	2.15324100	-0.86393300	0.20057900
O	3.13183600	-1.62981100	0.19914100
O	2.22090600	0.35956200	-0.19605300
O	-2.27059700	0.40057100	1.73148000
Mn	0.68956500	1.75163800	-0.29429900

Molecule:Ni-GLU-01

N	0.22521400	2.75199000	0.10877200
H	0.40015300	2.77815100	-0.89982200
H	1.06356400	3.09563300	0.58384200
H	-0.54844900	3.38824900	0.31716000
C	-0.13043200	1.36776300	0.55330200
H	-0.27417600	1.40628000	1.63188800
C	-1.42266900	0.92643000	-0.14499600
C	1.03105500	0.45673000	0.26181900
H	-2.13609400	1.74327700	-0.02539200
H	-1.22857400	0.81712900	-1.21353800
C	-2.01265500	-0.34981900	0.43524300
O	1.96328900	0.80228700	-0.52840800
H	-1.35328500	-1.20079200	0.26005300
H	-2.11131200	-0.25824500	1.52000700
C	-3.39167100	-0.72243500	-0.12063000
O	-3.90876700	-1.77524900	0.34779200
O	-3.92062100	0.01973500	-0.98995300
O	1.09569200	-0.69443300	0.78951000
Ni	2.79761300	-0.99729800	-0.20698400

Molecule:Ni-GLU-02

N	-3.33995700	1.67177700	-0.36480500
H	-3.54331400	1.73660900	0.63899200
H	-4.23472000	1.63703600	-0.85617400
H	-2.82921600	2.49993500	-0.67549700
C	-2.55773200	0.41322000	-0.58915100
H	-2.64364400	0.16230500	-1.64326300
C	-1.10297000	0.68027100	-0.20938200
C	-3.19356000	-0.69519400	0.27624400
H	-0.76248800	1.56001300	-0.75974900
H	-1.05028500	0.91816900	0.85469400
C	-0.19261600	-0.49651600	-0.53264600
O	-3.79083700	-0.31536700	1.31477000
H	-0.47110800	-1.38753000	0.03549600
H	-0.25969600	-0.77547700	-1.58621700
C	1.24396400	-0.23778200	-0.23242600
O	2.15443800	-1.03794000	-0.63980600
O	1.63707900	0.77142700	0.44114500
O	-3.02780600	-1.87141100	-0.11769100
Ni	3.50745200	-0.12817200	0.22436300

Molecule:Ni-GLU-032

N	2.78456000	1.64257000	-0.39719400
H	2.67918800	1.63459200	-1.41492100
H	3.65106800	1.14653900	-0.16698900
H	2.84386500	2.61426500	-0.08838400
C	1.63377900	0.94424300	0.26045600
H	1.70062600	1.19759000	1.31963400
C	0.31313500	1.44225400	-0.31911300
C	1.92553900	-0.56479700	0.16438800
H	0.44597900	2.47148400	-0.65093000
H	0.07296300	0.85614700	-1.20737400
C	-0.83987700	1.40584400	0.70634400
O	0.95215500	-1.39656800	0.26191000
H	-0.61491800	0.69209200	1.50202700
H	-0.96595500	2.38295500	1.16602900
C	-2.14663700	0.97881400	0.07239800
O	-3.09039900	1.75371000	-0.06971500
O	-2.21776200	-0.26361500	-0.32985700
O	3.09955400	-0.91827300	0.06263700
Ni	-0.87745500	-1.54049400	-0.08519200

Molecule:Zn-GLU-01

N	0.00071300	2.85926500	0.07331100
H	0.19415400	2.85021400	-0.93170800
H	0.81155600	3.26990700	0.54157700
H	-0.81284700	3.45383600	0.24657300
C	-0.26559200	1.46634200	0.55881000
H	-0.39908900	1.52503600	1.63656400
C	-1.53646200	0.94764300	-0.11972200
C	0.94947100	0.61367500	0.24455500
H	-2.30734700	1.70767300	0.01980200
H	-1.35269400	0.86495100	-1.19310900
C	-2.02455600	-0.37733400	0.44449900
O	1.73429500	0.96223300	-0.66963100
H	-1.29681900	-1.16945900	0.26325500
H	-2.13204500	-0.30624500	1.53023600
C	-3.36817900	-0.85714500	-0.11570200
O	-3.77947800	-1.97103700	0.31613700
O	-3.97772100	-0.13726600	-0.95107700
O	1.11457800	-0.45719500	0.90368700
Zn	2.80095500	-1.00479100	-0.18313100

Molecule:Zn-GLU-02

N	-3.46850300	1.68073700	-0.24086700
H	-3.64702700	1.68671700	0.76950100
H	-4.37387400	1.66035000	-0.71302900
H	-2.97679400	2.53267000	-0.51561800
C	-2.67211800	0.45028400	-0.55559300
H	-2.77697100	0.26212600	-1.62099800
C	-1.21340400	0.71618100	-0.19007500
C	-3.27578400	-0.71794100	0.25205100
H	-0.88983900	1.61630200	-0.71644200
H	-1.14764500	0.92227700	0.88046900
C	-0.29812600	-0.44210000	-0.55776800
O	-3.80600000	-0.41735900	1.35141000
H	-0.58199300	-1.35733100	-0.03700100
H	-0.36827900	-0.66886200	-1.62508400
C	1.15566700	-0.19980300	-0.25777700
O	1.98696000	-1.14340200	-0.45611200
O	1.56894400	0.91299400	0.18311100
O	-3.15636100	-1.85985500	-0.24685900
Zn	3.53720600	0.09339500	0.21556100

Molecule:Zn-GLU-03

N	2.52325400	1.12825800	-1.26010100
H	2.23915300	0.36466500	-1.88020400
H	3.50642300	0.99484300	-1.01242700
H	2.44000200	2.00753000	-1.77676800
C	1.64740000	1.15679800	-0.03780700
H	2.06559700	1.91174400	0.62190100
C	0.23579900	1.51971200	-0.48282400
C	1.77897600	-0.21958700	0.65729800
H	0.25734900	2.51570500	-0.92536000
H	-0.05161000	0.82160800	-1.27244400
C	-0.80188600	1.47082800	0.65594200
O	1.32880500	-1.24362800	0.01645700
H	-0.43947300	0.84215100	1.47419300
H	-0.97783000	2.46380100	1.06074500
C	-2.13201900	0.87284200	0.20961100
O	-3.16286500	1.55700900	0.24937200
O	-2.15125200	-0.35999900	-0.19661100
O	2.33474700	-0.26532900	1.75350100
Zn	-0.59558300	-1.53759400	-0.26880000

Molecule: Cd-ARG-01

N	1.21580800	3.36335400	-0.00085000
H	2.09945000	3.56126200	0.47319600
H	0.58473100	4.15509700	0.14204100
H	1.41497700	3.27718600	-1.00094500
C	0.59888600	2.10072100	0.51730400
H	0.47974500	2.22409200	1.59152600
C	-0.76124200	1.90379400	-0.15521900
C	1.55928000	0.95135000	0.24708300
H	-1.31011800	2.84342800	-0.05433300
H	-0.60736500	1.72774800	-1.22318100
C	-1.58303900	0.77576600	0.45551200
O	2.46799800	1.09255000	-0.60236400
H	-1.06249600	-0.17572500	0.34952600
H	-1.72000400	0.95861500	1.52464200
C	-2.93964400	0.67378800	-0.21961600
H	-3.47997600	1.62029400	-0.14015600
H	-2.80248100	0.45052600	-1.27984400
N	-3.71827800	-0.39336600	0.40196100
H	-3.37320200	-0.78788000	1.26511400
C	-4.88761000	-0.83541300	-0.04419100
N	-5.42984700	-0.33219400	-1.14976000
N	-5.53942000	-1.78257000	0.63530600
H	-4.96436400	0.38388300	-1.68185300
H	-6.28911600	-0.71704100	-1.50884300
H	-5.09077900	-2.23896000	1.41359500
H	-6.36750700	-2.20206000	0.24378200
O	1.36456900	-0.12382200	0.88797400
Cd	3.00438200	-1.29660200	-0.13356200

Molecule: Cd-ARG-02

N	-2.35051800	2.82506400	-0.89268100
H	-3.33331900	2.63216900	-1.09753100
H	-1.92732000	3.27016800	-1.71025800
H	-2.32083500	3.47399500	-0.09987000
C	-1.62038100	1.57335100	-0.52198200
H	-1.84919600	0.82945900	-1.28284800
C	-0.12341000	1.87428700	-0.48834900
C	-2.14833300	1.11806700	0.84403500
H	0.15268600	2.28833300	-1.46157700
H	0.06158000	2.64780600	0.26170600
C	0.73809900	0.65317100	-0.19665500
O	-2.53644800	1.98592800	1.63426600
H	0.50732700	0.25123800	0.79094300
H	0.52660800	-0.13292900	-0.93026100
C	2.21152600	1.01646800	-0.25258400
H	2.45826400	1.43262400	-1.23218300
H	2.43071600	1.77551400	0.50165100
N	3.02988800	-0.16596600	0.00103400
H	2.56754900	-1.06099700	0.06597800
C	4.35144200	-0.14883400	0.13132500
N	5.03114000	0.98852700	0.01378000
N	5.00794800	-1.28044400	0.39032800
H	4.56155000	1.86032000	-0.16234100
H	6.02647300	0.99690400	0.16961000
H	4.51124400	-2.15425900	0.45436600
H	6.01398600	-1.28671100	0.42872600
O	-2.12892200	-0.13288800	1.11374700
Cd	-1.63640000	-1.76536000	-0.21587900

Molecule: Cu-ARG-01

N	2.13793900	2.76891800	-0.24612900
H	3.03856100	2.89606000	0.22080400
H	1.67236500	3.67836900	-0.30299000
H	2.31621400	2.43870500	-1.19831800
C	1.27228300	1.79545800	0.49359400
H	1.15629300	2.17723100	1.50451200
C	-0.07791400	1.70206700	-0.21834900
C	1.96090300	0.44037200	0.56980800
H	-0.51932800	2.70132500	-0.21246200
H	0.09539200	1.42240500	-1.26076300
C	-1.03032800	0.70998100	0.43660600
O	2.65376800	0.10994100	-0.47343000
H	-0.60841300	-0.29579800	0.40088700
H	-1.17175200	0.96653800	1.48933100
C	-2.37461100	0.69837700	-0.26893100
H	-2.84337400	1.68357500	-0.21262900
H	-2.23887700	0.44946300	-1.32412100
N	-3.24357300	-0.29546000	0.35705800
H	-2.89563800	-0.77803200	1.17274000
C	-4.47015400	-0.59625600	-0.05052700
N	-5.00108800	-0.00751000	-1.11834100
N	-5.19308600	-1.48968000	0.63281200
H	-4.50501700	0.70457500	-1.62742800
H	-5.94015300	-0.23765500	-1.40139400
H	-4.74927200	-2.03719200	1.35334600
H	-6.05530900	-1.83421900	0.24036400
O	1.79891600	-0.28238800	1.54925700
Cu	3.27739400	-1.65136800	-0.36550000

Molecule: Cu-ARG-02

N	2.87272700	-2.25851600	-0.87088700
H	3.80836200	-1.96429300	-1.15992000
H	2.45929600	-2.81620500	-1.62140300
H	2.97734600	-2.84543500	-0.03812500
C	2.00610300	-1.08199700	-0.55148700
H	2.03410500	-0.41535900	-1.41210300
C	0.58194100	-1.57906900	-0.29646400
C	2.61878400	-0.38994000	0.66689100
H	0.31577600	-2.24223200	-1.12306400
H	0.57214800	-2.17521000	0.61917700
C	-0.43671300	-0.45272700	-0.21286300
O	3.25016300	-1.05479300	1.48355000
H	-0.18938100	0.22045300	0.61323200
H	-0.40271400	0.13545100	-1.13549800
C	-1.84251700	-0.98750100	-0.01444300
H	-2.09470700	-1.68129900	-0.81952400
H	-1.90338500	-1.53033600	0.93169000
N	-2.79366200	0.12108100	-0.00883300
H	-2.43406400	1.05178700	-0.16264200
C	-4.10951400	-0.01735600	0.10684400
N	-4.64916800	-1.21676900	0.30895100
N	-4.90257700	1.05071200	-0.00908500
H	-4.07250200	-2.02488700	0.47321700
H	-5.64442700	-1.30428300	0.44006100
H	-4.49751700	1.96902200	-0.09737600
H	-5.88088800	0.97756100	0.22039700
O	2.43528800	0.88149800	0.80393800
Cu	1.47824900	2.04164200	-0.28137600

Molecule: Fe-ARG-02

N	2.91157300	-2.24730000	-0.83833100
H	3.84490800	-1.94821900	-1.13222100
H	2.50405500	-2.81796500	-1.58324400
H	3.01765600	-2.83107900	-0.00315600
C	2.02647900	-1.07851500	-0.54553000
H	2.02182400	-0.44245900	-1.42918800
C	0.62189900	-1.59475700	-0.22924500
C	2.65185100	-0.32631800	0.62432500
H	0.31618100	-2.21483600	-1.07521100
H	0.67241300	-2.23795700	0.65222700
C	-0.38867900	-0.47963600	-0.02201000
O	3.30790200	-0.88307700	1.47395700
H	-0.14684300	0.09128200	0.87757900
H	-0.34798600	0.21333800	-0.86986400
C	-1.80465900	-1.01528500	0.09329100
H	-2.04556400	-1.61643900	-0.78555500
H	-1.89930200	-1.65071200	0.97677800
N	-2.73454800	0.10691800	0.18412600
H	-2.34452300	1.03633300	0.23897600
C	-4.05595500	0.00350300	0.09628800
N	-4.63892200	-1.19024000	0.02409500
N	-4.80606300	1.10694800	0.05792300
H	-4.10568400	-2.03653200	0.13288300
H	-5.64336100	-1.25691400	-0.02080800
H	-4.37646500	2.01343400	0.14895200
H	-5.80862500	1.03731700	0.12783000
O	2.42859900	0.98094800	0.67211600
Fe	1.50074600	2.16885400	-0.36622900

Molecule: Fe-ARG-011

N	2.22273200	2.82067100	-0.03229000
H	3.14906800	2.87581300	0.39995900
H	1.72623700	3.69527600	0.15870800
H	2.34624900	2.73860600	-1.04618800
C	1.43404600	1.67274700	0.50676700
H	1.38056500	1.78863600	1.58776000
C	0.02849000	1.68666000	-0.10792600
C	2.16151700	0.39711300	0.20882600
H	-0.40346200	2.65880600	0.14032800
H	0.12195600	1.62862200	-1.19455200
C	-0.86831000	0.56783000	0.40835300
O	2.98873400	0.26736200	-0.73729300
H	-0.48877300	-0.40046700	0.07772500
H	-0.87184600	0.56351400	1.50038800
C	-2.28564700	0.74251600	-0.10895700
H	-2.72688000	1.64603600	0.31663100
H	-2.27548800	0.85277600	-1.19618000
N	-3.09591800	-0.41634100	0.25846400
H	-2.62623200	-1.21697700	0.65610100
C	-4.38215800	-0.54900200	-0.04880000
N	-5.04018300	0.44730700	-0.63627900
N	-5.01812300	-1.68906000	0.22066100
H	-4.60129500	1.34003600	-0.78535000
H	-6.02627200	0.35861000	-0.82305800
H	-4.53274000	-2.45504500	0.65911000
H	-6.00563100	-1.77399300	0.04119400
O	1.94271300	-0.65192800	0.90519000
Fe	3.16830000	-1.78726700	-0.21766200

Molecule: Hg-ARG-01

N	0.31721600	3.37474500	-0.18092200
H	1.13676400	3.69977200	0.33686800
H	-0.34212800	4.15265900	-0.26212800
H	0.62233400	3.10864500	-1.12097600
C	-0.33503100	2.21682400	0.50907700
H	-0.57593600	2.54658400	1.51636000
C	-1.60237700	1.84124200	-0.25639800
C	0.64619500	1.04923400	0.61366600
H	-2.23051700	2.73509300	-0.29058100
H	-1.33499100	1.58867800	-1.28535400
C	-2.37756500	0.69435800	0.38160500
O	1.51567600	0.99671000	-0.34046300
H	-1.80881500	-0.23396200	0.30420400
H	-2.53346900	0.89564700	1.44395700
C	-3.72179700	0.51554300	-0.30184900
H	-4.32810500	1.41398600	-0.16802900
H	-3.58018800	0.36016200	-1.37438800
N	-4.42230100	-0.63235500	0.26777100
H	-3.94615800	-1.18351100	0.96714900
C	-5.65104700	-1.00305500	-0.07006600
N	-6.33370600	-0.33014900	-0.99189400
N	-6.21835500	-2.05098000	0.53462700
H	-5.94533200	0.48321500	-1.43930700
H	-7.27609000	-0.60320600	-1.22052600
H	-5.66362800	-2.63735500	1.13811300
H	-7.08613200	-2.42125800	0.18024300
O	0.53648400	0.24847500	1.53591800
Hg	2.79156100	-0.72850400	-0.13690700

Molecule: Hg-ARG-02

N	-1.85609400	3.25659100	-0.95001000
H	-2.84303100	3.10726200	-1.17147700
H	-1.39552500	3.66060400	-1.76856100
H	-1.80978800	3.92593100	-0.17497700
C	-1.19321100	1.98008100	-0.53978300
H	-1.44084800	1.23018300	-1.28882600
C	0.31688100	2.21014800	-0.47426200
C	-1.77151100	1.59121900	0.82229900
H	0.63476600	2.59257400	-1.44705600
H	0.52295600	2.98475100	0.26902200
C	1.09070700	0.94303100	-0.14562300
O	-2.13328800	2.48358400	1.58743600
H	0.81441700	0.57512700	0.84325600
H	0.82807000	0.16180100	-0.86801000
C	2.59189300	1.16790500	-0.18680800
H	2.88740000	1.58900500	-1.15068700
H	2.88273500	1.87313300	0.59434500
N	3.27185800	-0.10766600	0.02601200
H	2.70537400	-0.94363900	0.02789400
C	4.58623200	-0.26972000	0.12413700
N	5.40935200	0.77405300	0.08843200
N	5.09078600	-1.50249800	0.23704400
H	5.06242300	1.71353900	-0.00745600
H	6.40267100	0.63515400	0.18276700
H	4.47113200	-2.28266300	0.38843500
H	6.06733400	-1.62299200	0.45488500
O	-1.82889000	0.34451600	1.15787000
Hg	-1.39037400	-1.30629900	-0.12820100

Molecule: Mn-ARG-01

N	2.03068900	2.86656800	0.01773400
H	2.91319700	2.99413200	0.51704800
H	1.47070100	3.71550500	0.12510500
H	2.25172100	2.73829600	-0.97321200
C	1.28768500	1.67283400	0.53616000
H	1.17244100	1.81365800	1.60813900
C	-0.07713400	1.61075400	-0.14670800
C	2.13240700	0.42998700	0.28125400
H	-0.57433500	2.56843000	0.03056700
H	0.06925000	1.51297200	-1.22534700
C	-0.96047400	0.48013200	0.36759100
O	2.95270300	0.44959600	-0.67929600
H	-0.54717700	-0.48636600	0.07468800
H	-0.99816900	0.50318700	1.45910200
C	-2.36600800	0.61650400	-0.19091800
H	-2.81292500	1.54903400	0.16035300
H	-2.33363200	0.64918700	-1.28303000
N	-3.19419700	-0.50693100	0.23982700
H	-2.74958500	-1.26916900	0.73022200
C	-4.48330200	-0.63104000	-0.05219600
N	-5.11179400	0.31526200	-0.74465300
N	-5.16224100	-1.70228000	0.36501700
H	-4.63672400	1.15800700	-1.02060500
H	-6.09782700	0.23278800	-0.93394600
H	-4.67358100	-2.47599700	0.78656000
H	-6.11343300	-1.84061000	0.06313700
O	1.96111400	-0.56366900	1.02918800
Mn	3.64853200	-1.77335300	-0.27337900

Molecule: Mn-ARG-24

N	2.95076700	-2.16835600	-0.81723500
H	3.89188600	-1.87184500	-1.08308400
H	2.56203400	-2.73808700	-1.57111200
H	3.03310500	-2.73315100	0.03517800
C	2.07888700	-0.99106500	-0.51210900
H	2.24815600	-0.25152100	-1.29228700
C	0.62441700	-1.45807200	-0.50105600
C	2.52078500	-0.43564700	0.85230300
H	0.41028200	-1.89390400	-1.48072500
H	0.52218100	-2.25439800	0.24115000
C	-0.37897900	-0.35048800	-0.20630500
O	2.96542400	-1.24601900	1.68140600
H	-0.20799700	0.06162700	0.78951200
H	-0.25850200	0.46792100	-0.92449200
C	-1.79864300	-0.88166700	-0.28337600
H	-2.00182300	-1.27675700	-1.28158000
H	-1.92297700	-1.69681800	0.43300900
N	-2.74565600	0.18497200	0.02852900
H	-2.38367600	1.11933000	0.15099500
C	-4.05680100	0.01139100	0.14237500
N	-4.59910900	-1.18628500	-0.06115800
N	-4.83973900	1.04120000	0.47506200
H	-4.02266000	-1.99111200	-0.24124300
H	-5.58342400	-1.32775000	0.10126700
H	-4.45499800	1.97170700	0.51038800
H	-5.84133200	0.93949500	0.43317700
O	2.37195800	0.81059800	1.05127900
Mn	1.68035400	2.25964800	-0.44036100

Molecule: Ni-ARG-01

N	2.14446500	2.78806400	0.08118500
H	3.04642300	2.83871500	0.56195500
H	1.63546700	3.65796900	0.25718000
H	2.32635100	2.71814000	-0.92436700
C	1.33804700	1.62376000	0.56014900
H	1.22305000	1.72889000	1.63682200
C	-0.02730400	1.63336500	-0.13639100
C	2.10419900	0.36089400	0.26970100
H	-0.44063200	2.63675100	-0.01025600
H	0.12005300	1.46847800	-1.20628700
C	-0.99196100	0.60539100	0.43824500
O	2.95906400	0.31885700	-0.66901800
H	-0.58299400	-0.40065800	0.33328500
H	-1.13185200	0.79267400	1.50584900
C	-2.33673900	0.66605100	-0.26522400
H	-2.76598200	1.66741800	-0.18129200
H	-2.21405300	0.44021400	-1.32693400
N	-3.24069300	-0.30770400	0.34121500
H	-2.91213500	-0.81483300	1.14974000
C	-4.49222600	-0.52808000	-0.04385500
N	-5.00786600	0.10582300	-1.09378300
N	-5.24241000	-1.40097200	0.62990200
H	-4.47930500	0.79410000	-1.60258400
H	-5.96769300	-0.05534600	-1.35439000
H	-4.85429900	-1.90937400	1.40800200
H	-6.18137300	-1.60645100	0.32929200
O	1.88239800	-0.70043400	0.92461400
Ni	3.22531200	-1.62031500	-0.25950600

Molecule: Ni-ARG-02

N	2.81362000	-2.34055600	-0.77759000
H	3.75497700	-2.05929300	-1.06244000
H	2.40859900	-2.91631000	-1.51956600
H	2.90032800	-2.90832000	0.07068100
C	1.94802400	-1.15143300	-0.50986500
H	1.99952400	-0.51413700	-1.39103600
C	0.51332400	-1.62458900	-0.27585100
C	2.53060500	-0.43190500	0.70468400
H	0.22076300	-2.20766100	-1.15252200
H	0.49879200	-2.29393100	0.58757400
C	-0.46676900	-0.47808300	-0.07515300
O	3.07819500	-1.06280400	1.59122000
H	-0.25065900	0.04564000	0.85873700
H	-0.35923500	0.24559000	-0.89098200
C	-1.89985900	-0.97875300	-0.04563900
H	-2.14191600	-1.46413600	-0.99331200
H	-2.02635900	-1.71170900	0.75483100
N	-2.80251300	0.14788400	0.16782300
H	-2.39322300	1.05285900	0.34778900
C	-4.12723400	0.07497700	0.11263800
N	-4.73275700	-1.08835400	-0.10747100
N	-4.85893800	1.18346700	0.25680300
H	-4.20526100	-1.93637700	-0.22857400
H	-5.73777700	-1.13370000	-0.15614500
H	-4.41163300	2.04682300	0.52016400
H	-5.85998000	1.11447700	0.34775400
O	2.39896300	0.86963900	0.76375600
Ni	1.70933400	2.08596900	-0.39909300

Molecule: Zn-ARG-01

N	2.09240700	2.88305500	-0.06140100
H	2.99501200	2.98040100	0.40918100
H	1.58767600	3.76917700	0.02032500
H	2.27099500	2.69976000	-1.05258300
C	1.28580300	1.77224300	0.53572800
H	1.19134400	1.98181500	1.59812700
C	-0.08788600	1.74272300	-0.13881800
C	2.02771500	0.46183700	0.34577700
H	-0.52407200	2.73852900	-0.02866200
H	0.05152700	1.55902900	-1.20726700
C	-1.02465100	0.70123300	0.45855800
O	2.77527200	0.32616900	-0.67453500
H	-0.59097800	-0.29522200	0.36295500
H	-1.16227000	0.89616700	1.52510500
C	-2.37360100	0.71647300	-0.23855900
H	-2.83936900	1.70087800	-0.15023400
H	-2.24524200	0.49851500	-1.30142800
N	-3.24207900	-0.29355800	0.36245000
H	-2.89597900	-0.79196000	1.16935800
C	-4.47258800	-0.57761800	-0.04493600
N	-5.00602400	0.03658600	-1.09710100
N	-5.19750200	-1.48140500	0.62332200
H	-4.51207000	0.76243400	-1.58844900
H	-5.95073600	-0.17707200	-1.37435900
H	-4.74942600	-2.05285500	1.32249500
H	-6.05948300	-1.81729500	0.22262400
O	1.84179800	-0.46945900	1.16065500
Zn	3.12800500	-1.67400200	-0.27045100

Molecule: Zn-ARG-021

N	2.83431100	-2.32008300	-0.80016900
H	3.78808000	-2.04598400	-1.04579600
H	2.44498000	-2.86953000	-1.56942900
H	2.88743400	-2.90702200	0.03806100
C	1.98335500	-1.12401400	-0.51313700
H	2.10524200	-0.43227200	-1.34566500
C	0.52865500	-1.57977700	-0.39244500
C	2.49363200	-0.48499700	0.78171500
H	0.26525600	-2.08868100	-1.32316400
H	0.45993000	-2.31348100	0.41513000
C	-0.45179200	-0.44100900	-0.14182200
O	3.02364000	-1.20744000	1.62869500
H	-0.27268100	0.00490800	0.83760100
H	-0.30835000	0.34737700	-0.89006700
C	-1.88288200	-0.94365700	-0.20437300
H	-2.08445700	-1.39107100	-1.18045200
H	-2.03412900	-1.71120300	0.55814300
N	-2.80862600	0.16057600	0.02999700
H	-2.42869000	1.09416300	0.08699300
C	-4.12472600	0.02122600	0.13038300
N	-4.68727800	-1.17906300	0.01465200
N	-4.89358400	1.08925200	0.36537900
H	-4.12451600	-2.00851500	-0.07411000
H	-5.67750000	-1.28629600	0.16782100
H	-4.49418000	2.01231000	0.30085000
H	-5.89521100	1.00117300	0.29578300
O	2.30667100	0.77537700	0.93635400
Zn	1.61117000	2.03697400	-0.36743400

Molecule: Cd-HIS-02

N	-3.28940400	0.40244600	-1.03140700
H	-3.83754600	0.78295000	-0.25372200
H	-3.67232300	-0.51872800	-1.25433500
H	-3.39392600	1.00760100	-1.84929400
C	-1.85778400	0.29528600	-0.61746400
H	-1.33473900	-0.26187900	-1.38982900
C	-1.29500000	1.72343100	-0.50768600
C	-1.81716900	-0.46178100	0.71891700
H	-1.45354800	2.22162700	-1.46439200
H	-1.85663900	2.26874100	0.25355800
C	0.15367100	1.77649700	-0.19299800
O	-2.80710000	-0.38401500	1.45419500
N	0.65074900	1.78658100	1.09887900
C	1.24314400	1.80541600	-1.01086400
H	0.08889000	1.74743800	1.94028200
C	1.97806100	1.80956400	1.07912100
N	2.35463200	1.82170200	-0.19422900
H	1.31790000	1.81725300	-2.08247500
H	2.62741100	1.82166800	1.93626700
H	3.31730900	1.85284800	-0.50355200
O	-0.75215400	-1.10184200	1.01616100
Cd	1.00478900	-1.47121400	-0.22962900

Molecule: Cd-HIS-011

N	0.83249000	3.23829600	-0.23719400
H	0.58319100	3.50152200	0.72030400
H	0.10374000	3.60475800	-0.85330200
H	1.72310900	3.68111300	-0.47394800
C	0.93536100	1.75335100	-0.36956500
H	1.27853600	1.55429200	-1.38355600
C	1.95354900	1.23263700	0.66200000
C	-0.46817600	1.16343500	-0.20189000
H	2.73890900	1.97933600	0.78046200
H	1.46062900	1.12638600	1.63124600
C	2.62765600	-0.03907900	0.28541300
O	-1.42330200	1.90465400	0.03793500
N	1.95718300	-1.18197500	-0.10679500
C	3.94942700	-0.36167700	0.28948900
H	0.94492100	-1.19838200	-0.24751300
C	2.82256900	-2.16021700	-0.33817600
N	4.03843800	-1.68265900	-0.09520300
H	4.81640800	0.22295800	0.53623500
H	2.58621900	-3.15581300	-0.66817500
H	4.89261200	-2.21548000	-0.19208400
O	-0.53999200	-0.10895700	-0.32908800
Cd	-2.58627100	-0.74192900	0.07479700

Molecule: Cu-HIS-01

N	0.78346300	3.04204400	0.00895800
H	1.05241100	3.03476900	-0.97953800
H	1.63458500	3.15379700	0.56646200
H	0.18212000	3.85434800	0.17307100
C	0.06343200	1.78905600	0.37690100
H	-0.18341200	1.85473200	1.43471600
C	-1.22607700	1.68803100	-0.45969500
C	0.98476200	0.60859400	0.18577500
H	-1.83748200	2.55890000	-0.22238100
H	-0.97110600	1.73409700	-1.51922900
C	-1.99165900	0.44378400	-0.18879800
O	2.05779900	0.68885900	-0.46398500
N	-2.08924600	-0.58279000	-1.10951400
C	-2.66062300	0.01813000	0.91652900
H	-1.69105600	-0.57282900	-2.04019100
C	-2.77949300	-1.59430400	-0.59556900
N	-3.13361000	-1.24407400	0.63537000
H	-2.82494000	0.49247300	1.86635100
H	-3.01812600	-2.51896900	-1.08992800
H	-3.67238900	-1.82791500	1.26216400
O	0.66063300	-0.50474500	0.70890900
Cu	2.28674600	-1.36077000	0.01498500

Molecule: Cu-HIS-022

N	2.95302900	-1.09916800	-0.88786700
H	3.22534300	-1.75042100	-0.14166700
H	3.66576300	-0.36340500	-0.93462900
H	2.95459600	-1.60498100	-1.78106700
C	1.60008200	-0.52268800	-0.61474600
H	1.38137100	0.18087300	-1.41851800
C	0.57854000	-1.68391700	-0.62492100
C	1.65361400	0.22669900	0.72309600
H	0.58090100	-2.11505100	-1.62916000
H	0.91354000	-2.45415300	0.07719600
C	-0.81540400	-1.28383200	-0.28076100
O	2.43576900	-0.14854200	1.60221000
N	-1.32729600	-1.38211400	1.00675100
C	-1.84263300	-0.80704000	-1.05333400
H	-0.80924800	-1.70541700	1.81897900
C	-2.59841500	-0.97935300	1.03060400
N	-2.92515600	-0.62325700	-0.21264300
H	-1.88997800	-0.58972000	-2.10806100
H	-3.24173500	-0.94783100	1.89578300
H	-3.83942100	-0.27701800	-0.48750200
O	0.84577900	1.23546600	0.88527100
Cu	-0.39835300	1.89532000	-0.33153500

Molecule: Fe-HIS-01

N	-0.50533200	2.77873800	-0.18965100
H	-0.86040500	2.98769700	0.74892200
H	-1.26666400	2.92965200	-0.85811500
H	0.24696000	3.43918400	-0.40472200
C	0.01648400	1.38191600	-0.27255300
H	0.39283800	1.24697100	-1.28819100
C	1.14670900	1.17355300	0.75305800
C	-1.14225900	0.43712400	-0.11592000
H	1.64470600	2.13316900	0.88923600
H	0.72407600	0.88523000	1.71602200
C	2.19077100	0.21057400	0.31884300
O	-2.33999600	0.85806300	-0.16017300
N	2.02412500	-1.16214400	0.24556700
C	3.47327400	0.45153000	-0.06226600
H	1.16427300	-1.65866500	0.44527000
C	3.15073300	-1.73431300	-0.16398900
N	4.04178300	-0.76845200	-0.35219700
H	4.01792200	1.37397100	-0.14517800
H	3.31309200	-2.78708500	-0.31107700
H	4.99518100	-0.91931500	-0.65514700
O	-0.99270700	-0.81458600	0.02149900
Fe	-3.06341000	-1.05530500	0.01166500

Molecule: Fe-HIS-021

N	2.91891700	-1.23326200	-0.81084700
H	3.33382100	-1.62192900	0.04196800
H	3.56246600	-0.52565700	-1.17452900
H	2.83406900	-1.98102600	-1.50451400
C	1.58297500	-0.63598800	-0.52427700
H	1.21804400	-0.19022800	-1.44699800
C	0.63148400	-1.75648400	-0.05384100
C	1.77865600	0.44615600	0.52590100
H	0.70673000	-2.56881100	-0.77827100
H	0.96936600	-2.13557700	0.91142500
C	-0.78924200	-1.33402700	0.03085300
O	2.74938600	0.47733100	1.24710600
N	-1.41061700	-0.96440000	1.21043200
C	-1.73020100	-1.17430500	-0.94759500
H	-0.96937100	-0.94959700	2.12249400
C	-2.66479400	-0.59796500	0.97376000
N	-2.87382200	-0.72059400	-0.33250300
H	-1.67970400	-1.35993600	-2.00474500
H	-3.38025700	-0.26839400	1.70584300
H	-3.75514000	-0.52809500	-0.79118300
O	0.80571100	1.33281300	0.62543000
Fe	-0.56059300	1.86121400	-0.48308300

Molecule: Hg-HIS-01

N	1.02742200	3.15810300	0.10768900
H	0.46943600	3.06106600	0.96075500
H	0.43262400	3.57970300	-0.60945200
H	1.80688300	3.79394300	0.29691800
C	1.53760500	1.82539500	-0.33958800
H	2.06165900	1.97847800	-1.27847200
C	2.50054900	1.29442400	0.73801800
C	0.37211900	0.86058300	-0.57121800
H	3.26503200	2.05413500	0.90261900
H	1.95122100	1.16233100	1.67223100
C	3.18143700	0.02716400	0.37039000
O	-0.66554200	1.12298200	0.14796600
N	2.65744900	-1.22762200	0.63016600
C	4.38210200	-0.19024100	-0.23002600
H	1.75735800	-1.40865800	1.05597300
C	3.48987100	-2.16870400	0.20175600
N	4.54406800	-1.55489200	-0.32270400
H	5.12277300	0.50158200	-0.58637900
H	3.33720200	-3.23097300	0.26851500
H	5.34391300	-2.03145200	-0.71856900
O	0.51963300	-0.07958800	-1.34080200
Hg	-2.18456900	-0.37910200	0.04570600

Molecule: Hg-HIS-021

N	-3.41428100	-0.70918700	-1.05811400
H	-4.09103800	-0.49400700	-0.31793500
H	-3.43365100	-1.71984800	-1.21382400
H	-3.70417200	-0.24314500	-1.92135500
C	-2.05381700	-0.26039000	-0.63857400
H	-1.33938600	-0.67187100	-1.34667400
C	-2.03805800	1.27741000	-0.67856800
C	-1.79944400	-0.82307800	0.76668900
H	-2.27413600	1.58210600	-1.69816700
H	-2.82591200	1.65501300	-0.02379300
C	-0.74252100	1.89574500	-0.29888300
O	-2.75467100	-0.95468000	1.52850600
N	-0.43607400	2.28736600	0.99278100
C	0.34735100	2.22141200	-1.05236800
H	-1.03942400	2.16965700	1.79767800
C	0.77784000	2.82466300	1.03378100
N	1.26636200	2.79450200	-0.20003200
H	0.53230600	2.10751600	-2.10467500
H	1.27289800	3.21331200	1.90559700
H	2.17839600	3.14548000	-0.46254800
O	-0.59303800	-1.12407400	1.11776600
Hg	1.15807000	-0.84421900	-0.10899200

Molecule: Mn-HIS-11

N	0.58975600	3.13118500	-0.04921200
H	1.06830900	3.01556200	-0.94713100
H	1.28815500	3.42662100	0.63658000
H	-0.11018200	3.87178100	-0.13853100
C	-0.04428900	1.84258800	0.36198400
H	-0.43660600	1.97737500	1.36609900
C	-1.18583300	1.53303200	-0.62358400
C	1.00336300	0.73584500	0.35508300
H	-1.88977100	2.36469400	-0.59547400
H	-0.77621000	1.47241800	-1.63384000
C	-1.91373200	0.27940300	-0.30496700
O	2.02809200	0.87721100	-0.35969600
N	-1.57359800	-0.94969400	-0.84147700
C	-2.97407500	0.04602900	0.51357500
H	-0.79309200	-1.11849100	-1.46343900
C	-2.37559100	-1.89510700	-0.36683900
N	-3.23467300	-1.30606600	0.45736300
H	-3.55523900	0.71689600	1.11879300
H	-2.33896500	-2.94245600	-0.60741800
H	-3.97327400	-1.78721500	0.95379600
O	0.73906500	-0.29943700	1.01853400
Mn	2.55400600	-1.47992400	-0.02153400

Molecule: Mn-HIS-21

N	2.94021300	-0.88011500	-1.01994800
H	3.40289500	-1.30104600	-0.20715300
H	3.51728400	-0.09651300	-1.33209600
H	2.88843200	-1.56627700	-1.77634800
C	1.58335900	-0.40721600	-0.60969500
H	1.21485100	0.25448500	-1.38953900
C	0.67498700	-1.64139400	-0.48189300
C	1.73654700	0.35912500	0.71636100
H	0.70432700	-2.17751200	-1.43063600
H	1.07353300	-2.30186300	0.29026700
C	-0.73959900	-1.30984900	-0.18027700
O	2.63995100	-0.00532400	1.48470100
N	-1.24274900	-1.19464000	1.10475700
C	-1.78512500	-1.04229200	-1.01166000
H	-0.70578500	-1.31948000	1.95400500
C	-2.52643400	-0.85628100	1.06673700
N	-2.87083600	-0.75745100	-0.21193500
H	-1.84198500	-1.02211500	-2.08431800
H	-3.16849600	-0.69778500	1.91446200
H	-3.79990700	-0.52356400	-0.53720000
O	0.90806000	1.29417800	0.93802600
Mn	-0.68472200	1.98614900	-0.43563300

Molecule: Ni-HIS-01

N	-0.49551800	2.86056200	-0.21457500
H	-0.85538600	3.02570900	0.73030600
H	-1.26049200	3.02756400	-0.87406000
H	0.24913900	3.53775800	-0.39997000
C	0.03286700	1.47130300	-0.35101300
H	0.42346500	1.37878500	-1.36447800
C	1.14769100	1.23707800	0.68664600
C	-1.12148400	0.50910500	-0.20851000
H	1.67289800	2.18063300	0.83125600
H	0.69793700	0.96476200	1.64354900
C	2.17311500	0.23569100	0.28976500
O	-2.31216300	0.91652300	-0.12061900
N	1.91340800	-1.09922900	0.03159700
C	3.51785300	0.38633700	0.15418800
H	0.98278300	-1.50472300	0.01064100
C	3.04272200	-1.73555800	-0.25296200
N	4.02886100	-0.84939200	-0.17951200
H	4.14246500	1.25198400	0.27555600
H	3.13973100	-2.77642900	-0.50472000
H	5.00384500	-1.06267500	-0.34502900
O	-0.90272800	-0.74368700	-0.16063900
Ni	-2.83432500	-1.08619200	0.10270500

Molecule: Ni-HIS-02

N	-2.79163500	1.65461700	-0.63970800
H	-3.13186500	1.98470000	0.26860700
H	-3.55990600	1.14270200	-1.08036900
H	-2.55724400	2.46609900	-1.21677300
C	-1.60885000	0.76242400	-0.47369700
H	-1.29500300	0.47809000	-1.47733200
C	-0.50950200	1.53667300	0.25868300
C	-2.07226500	-0.48945700	0.27825000
H	-0.48640000	2.56011900	-0.12942400
H	-0.73907700	1.60479700	1.32357200
C	0.86985900	1.01329300	0.08821000
O	-3.23351400	-0.58336500	0.64783100
N	1.85839200	1.32909200	0.98155900
C	1.49812200	0.30526000	-0.92850000
H	1.71588600	1.81415300	1.86200200
C	3.04499400	0.90310200	0.53819200
N	2.85780800	0.30252400	-0.61581400
H	1.14885800	0.10064100	-1.93033400
H	3.98389600	1.02032900	1.05271600
H	3.59099800	-0.12045100	-1.17466200
O	-1.18253000	-1.39996300	0.49382700
Ni	0.56614700	-1.58485500	-0.11715600

Molecule: Zn-HIS-01

N	-0.43024200	3.05061400	-0.06801700
H	-0.81251700	3.09931800	0.88050700
H	-1.19033800	3.26581400	-0.71779200
H	0.29482400	3.76539500	-0.16688900
C	0.13145900	1.69345400	-0.33912500
H	0.47632300	1.68791900	-1.37021800
C	1.31326500	1.45552400	0.61655600
C	-0.97888900	0.67047100	-0.17958900
H	1.95681200	2.33409400	0.56608000
H	0.94108800	1.37371800	1.63896000
C	2.13772700	0.26863200	0.27441800
O	-2.04856600	0.99076700	0.38492200
N	1.91498400	-0.99816200	0.78401900
C	3.22927400	0.15864400	-0.52971000
H	1.14913300	-1.25666000	1.39281300
C	2.81889600	-1.84721500	0.31051200
N	3.62596600	-1.15996100	-0.48988600
H	3.74914900	0.89801000	-1.11067100
H	2.88633500	-2.89648900	0.53546100
H	4.41878800	-1.55505800	-0.97863800
O	-0.77161900	-0.49709000	-0.62637000
Zn	-2.63311500	-1.17699900	-0.04130700

Molecule: Zn-HIS-03

N	-2.95966400	1.04439400	-0.93595100
H	-3.37599000	1.47359900	-0.10311500
H	-3.57208800	0.28001900	-1.22896400
H	-2.92139300	1.73576700	-1.68877100
C	-1.59817900	0.53196000	-0.60122400
H	-1.27808200	-0.10300400	-1.42256900
C	-0.65372600	1.73591300	-0.45623300
C	-1.70342800	-0.28220700	0.69571600
H	-0.68849800	2.30249500	-1.38682100
H	-1.01937300	2.38096900	0.34458100
C	0.75652300	1.35708600	-0.18569500
O	-2.59501500	0.00440400	1.49590600
N	1.29648700	1.30517600	1.08717000
C	1.76690100	1.01386500	-1.03169800
H	0.79022300	1.49632900	1.94296200
C	2.57380800	0.94637500	1.02963200
N	2.87585600	0.76587800	-0.25081400
H	1.79184700	0.94045100	-2.10323000
H	3.23978100	0.82770500	1.86545300
H	3.79064700	0.49851100	-0.59060600
O	-0.82674200	-1.19834700	0.89334000
Zn	0.50922800	-1.86358000	-0.35829100

Molecule: Cd-LYS-01

N	0.05668700	3.15198500	-0.03756400
H	0.29440500	3.10919700	-1.03201700
H	0.88505200	3.47389500	0.46729100
H	-0.69024400	3.83800500	0.09278200
C	-0.38208800	1.80564900	0.45169300
H	-0.55032800	1.90049800	1.52211100
C	-1.67838600	1.41982600	-0.26096900
C	0.75254400	0.81921100	0.22054600
H	-2.37214700	2.25440800	-0.12931200
H	-1.48207600	1.32475800	-1.33209500
C	-2.32087100	0.14741000	0.27947100
O	1.61330200	1.07821000	-0.66130900
H	-1.70395400	-0.71871700	0.03362600
H	-2.37446200	0.20197500	1.37008800
C	-3.72483200	-0.04526200	-0.28537700
H	-4.34446200	0.82004800	-0.03277500
H	-3.68463000	-0.10923000	-1.37661000
C	-4.37038400	-1.29809400	0.26863800
H	-3.82337400	-2.19566600	-0.01158800
H	-4.45748000	-1.26064700	1.35230400
N	-5.76114000	-1.45577300	-0.26900600
H	-5.74800900	-1.55149100	-1.28647000
H	-6.21574500	-2.28237200	0.12367800
H	-6.33184300	-0.63913100	-0.03930500
O	0.78249000	-0.22415700	0.92596000
Cd	2.78558800	-0.91591400	-0.07785300

Molecule: Cd-LYS-02

N	-1.52318200	2.89633300	-1.02230700
H	-1.49314200	3.56684600	-0.24760400
H	-2.50307900	2.77030000	-1.28434700
H	-1.01898500	3.28317700	-1.82259800
C	-0.91065800	1.60668000	-0.57305300
H	-1.16044400	0.85498800	-1.31969000
C	0.60321200	1.79178700	-0.47876100
C	-1.53407100	1.24630900	0.78049800
H	0.95628500	2.11152500	-1.46254800
H	0.81334400	2.60273000	0.22393400
C	1.34974300	0.53308400	-0.05425700
O	-1.87360400	2.17161000	1.52765300
H	1.07642400	0.26217700	0.96717600
H	1.05474800	-0.30417300	-0.69748500
C	2.86093500	0.71860200	-0.14257200
H	3.14469600	0.95561700	-1.17180100
H	3.16895600	1.56054700	0.48390800
C	3.58725500	-0.53234200	0.30515700
H	3.38180700	-0.76777500	1.34713400
H	3.32737100	-1.39199800	-0.30907700
N	5.07123000	-0.35470100	0.18859500
H	5.38745900	0.43727800	0.75250000
H	5.56791000	-1.18903600	0.50596100
H	5.33980200	-0.17487000	-0.78143400
O	-1.63800600	0.00858800	1.08779900
Cd	-1.24010600	-1.70823000	-0.19361900

Molecule: Cu-LYS-01

N	1.15464700	2.87771500	-0.16505100
H	1.33906200	2.72212000	-1.16029700
H	2.04601900	3.07997200	0.29420200
H	0.55074600	3.69866900	-0.07432400
C	0.48682700	1.68415900	0.44405000
H	0.35973800	1.89060700	1.50451600
C	-0.86696900	1.45626300	-0.23834000
C	1.38760900	0.48959700	0.27210100
H	-1.40464600	2.40668400	-0.21066900
H	-0.69173000	1.20706600	-1.28812400
C	-1.71125000	0.37627300	0.42803900
O	2.20750400	0.42862500	-0.69583700
H	-1.16831600	-0.57003400	0.44404600
H	-1.90087800	0.65427500	1.46804200
C	-3.03438800	0.17826500	-0.30431100
H	-3.59054000	1.11925100	-0.34034100
H	-2.84121300	-0.12574900	-1.33714500
C	-3.87900300	-0.87788100	0.37813700
H	-3.37342400	-1.84048600	0.41292400
H	-4.15214800	-0.58641900	1.38986500
N	-5.15967100	-1.09285300	-0.37011300
H	-4.97126800	-1.40549200	-1.32523400
H	-5.73970100	-1.79895500	0.08640200
H	-5.69848200	-0.22567300	-0.42712200
O	1.29801200	-0.50784200	1.04330600
Cu	2.65278900	-1.44573000	-0.14994500

Molecule: Cu-LYS-02

N	2.17726600	-2.32460300	-0.85175900
H	2.26101700	-2.90353300	-0.01098100
H	3.12250200	-2.06816200	-1.14531800
H	1.74302600	-2.87745800	-1.59417700
C	1.35369400	-1.11469900	-0.54691300
H	1.40751600	-0.45730500	-1.41365200
C	-0.08871200	-1.55761500	-0.29140300
C	1.98874300	-0.43338800	0.66500300
H	-0.39506500	-2.16463800	-1.14732500
H	-0.10686900	-2.20301400	0.59068500
C	-1.06506900	-0.40170700	-0.11886300
O	2.58145300	-1.11276600	1.49854200
H	-0.79995400	0.17988100	0.76795900
H	-0.98398000	0.27215000	-0.98025000
C	-2.50651300	-0.87962500	0.00945400
H	-2.77842600	-1.48124500	-0.86230300
H	-2.60675900	-1.51683000	0.89252400
C	-3.45565100	0.29489700	0.12393300
H	-3.23654500	0.90898100	0.99470600
H	-3.43086100	0.92139700	-0.76519300
N	-4.86829900	-0.18009400	0.27602600
H	-4.97064400	-0.74477400	1.12232000
H	-5.51642800	0.60709400	0.33914500
H	-5.14476300	-0.75713700	-0.52123400
O	1.86786200	0.84845400	0.77629400
Cu	0.94206800	2.01671700	-0.32699100

Molecule: Fe-LYS-01

N	1.26935700	2.86350900	-0.14896900
H	1.42525500	2.73983000	-1.15417800
H	2.17208700	3.04856500	0.29675200
H	0.67352700	3.68617100	-0.01832300
C	0.59955200	1.66622300	0.44546000
H	0.49696200	1.84382700	1.51402400
C	-0.77401100	1.46650100	-0.21060800
C	1.46880400	0.46320700	0.23928400
H	-1.31594400	2.40586400	-0.08204200
H	-0.62839100	1.30931100	-1.28196300
C	-1.58349300	0.32315300	0.38907000
O	2.23713400	0.33065600	-0.76231200
H	-1.05643900	-0.62377900	0.25913500
H	-1.69922500	0.48129800	1.46418800
C	-2.95609800	0.22350000	-0.26887700
H	-3.50388000	1.16006100	-0.13258800
H	-2.83956600	0.06640100	-1.34492200
C	-3.75715500	-0.91787900	0.32143900
H	-3.26992400	-1.87794100	0.16484400
H	-3.93095800	-0.78032800	1.38649100
N	-5.10488800	-1.00804000	-0.32654700
H	-5.01295100	-1.17563500	-1.33088700
H	-5.65472800	-1.76961600	0.07457600
H	-5.62612600	-0.13776000	-0.19985400
O	1.42161400	-0.53092600	1.03267600
Fe	2.66782400	-1.58117800	-0.15169800

Molecule: Fe-LYS-02

N	2.22390100	-2.31229400	-0.81760000
H	2.22546600	-2.93784600	-0.00612000
H	3.19439600	-2.06156200	-1.02289300
H	1.84351000	-2.82388000	-1.61798700
C	1.38787500	-1.10450700	-0.54525700
H	1.46107000	-0.45439900	-1.41655700
C	-0.06062100	-1.54847900	-0.31631100
C	1.98027400	-0.39799500	0.66626100
H	-0.35658300	-2.12275500	-1.19755800
H	-0.08538500	-2.22424600	0.54230800
C	-1.03380800	-0.39623000	-0.10871700
O	2.55768400	-0.99254600	1.54679200
H	-0.78161100	0.14439000	0.80708000
H	-0.93924300	0.31551400	-0.93731900
C	-2.48061400	-0.86792100	-0.02082400
H	-2.74850200	-1.42404300	-0.92323200
H	-2.59670300	-1.54483300	0.83008000
C	-3.41477400	0.31318900	0.14045100
H	-3.19198700	0.88417500	1.03933100
H	-3.37381900	0.97936400	-0.71871800
N	-4.83613600	-0.14245000	0.26304500
H	-4.95482400	-0.74380200	1.08142400
H	-5.46763900	0.65469100	0.36096100
H	-5.12094900	-0.67507400	-0.56170100
O	1.81740000	0.91897300	0.71369500
Fe	0.99645400	2.14683700	-0.35979700

Molecule: Hg-LYS-01

N	-0.68546000	3.34507100	-0.12794500
H	-0.45672000	3.26719500	-1.12266500
H	0.12875600	3.73755500	0.35055700
H	-1.46742900	3.99628800	-0.02319300
C	-1.05325000	2.00644900	0.43475900
H	-1.24902100	2.15298300	1.49420600
C	-2.30256000	1.48909400	-0.27961100
C	0.13398500	1.06679500	0.28085900
H	-3.04683400	2.28852000	-0.23707400
H	-2.06504500	1.31948500	-1.33282400
C	-2.88204300	0.22739500	0.34780100
O	0.96981600	1.29980900	-0.63489500
H	-2.17029100	-0.59566800	0.26292200
H	-3.04780600	0.39730900	1.41512000
C	-4.19741500	-0.17124700	-0.31550300
H	-4.91572900	0.65011100	-0.24051400
H	-4.03313500	-0.36528500	-1.37946700
C	-4.78002100	-1.40709800	0.33609700
H	-4.11409600	-2.26287500	0.24962600
H	-5.00704000	-1.24086800	1.38689500
N	-6.06797600	-1.79655300	-0.32536000
H	-5.92203000	-1.99523400	-1.31768600
H	-6.46979100	-2.62939000	0.10945900
H	-6.75451200	-1.04189700	-0.25696700
O	0.21719400	0.08945800	1.06250000
Hg	2.23570600	-0.61125400	-0.05540600

Molecule: Hg-LYS-02

N	-0.82317600	3.37923400	-1.02720600
H	-0.67176700	4.05241200	-0.26961100
H	-1.81456200	3.40607800	-1.27512500
H	-0.27504500	3.66459400	-1.84175000
C	-0.41658300	2.01448800	-0.56840200
H	-0.76347400	1.30517800	-1.31719800
C	1.10660900	1.97627000	-0.44574700
C	-1.12101600	1.75868300	0.76676000
H	1.51519700	2.29696400	-1.40708600
H	1.41594400	2.71008200	0.30331600
C	1.66189200	0.60381700	-0.09313700
O	-1.34599900	2.71425800	1.50834200
H	1.30817600	0.29903100	0.89397500
H	1.28091300	-0.13479900	-0.80976900
C	3.18624400	0.58184000	-0.10419700
H	3.55882900	0.89245600	-1.08422600
H	3.57041200	1.29298200	0.63285900
C	3.70623400	-0.80346600	0.21543600
H	3.36147900	-1.15082600	1.18702100
H	3.41574400	-1.52845200	-0.54149900
N	5.20379000	-0.80907400	0.26563700
H	5.54448600	-0.18059100	0.99632500
H	5.56126100	-1.74584100	0.46066700
H	5.59997000	-0.49686600	-0.62380800
O	-1.44425300	0.55114600	1.09975100
Hg	-1.12112700	-1.19483200	-0.11827700

Molecule: Mn-LYS-11

N	1.11770300	2.87582800	-0.11928300
H	1.34882800	2.72568800	-1.10494800
H	1.98326900	3.10896800	0.37208900
H	0.47903000	3.67094300	-0.04733700
C	0.48706600	1.64259900	0.45298300
H	0.36622200	1.81395200	1.51988800
C	-0.86905400	1.42602300	-0.21881300
C	1.43502500	0.47455100	0.23115700
H	-1.44168000	2.34966300	-0.10163000
H	-0.71113500	1.27192600	-1.28978600
C	-1.66134100	0.26351300	0.36810400
O	2.21021400	0.49851100	-0.76364900
H	-1.11390200	-0.67101800	0.23294200
H	-1.77886100	0.41050500	1.44513100
C	-3.03489700	0.14089000	-0.28371100
H	-3.59750800	1.06853700	-0.14528400
H	-2.92224100	-0.01472600	-1.36051600
C	-3.81553600	-1.01218200	0.31135300
H	-3.31619900	-1.96527800	0.15062800
H	-3.98248900	-0.87771700	1.37796200
N	-5.16811100	-1.12172500	-0.32440700
H	-5.08295100	-1.28520600	-1.32992500
H	-5.70197800	-1.89281700	0.08011400
H	-5.70117000	-0.25990900	-0.18996500
O	1.39442600	-0.49495600	1.03441300
Mn	3.01763700	-1.57492100	-0.15344300

Molecule: Mn-LYS-21

N	2.25913000	-2.17004800	-0.95457100
H	2.33839300	-2.78442400	-0.13765600
H	3.20400700	-1.88218100	-1.21648700
H	1.84608700	-2.68582900	-1.73417700
C	1.42013900	-0.99063400	-0.57258900
H	1.56916800	-0.22800100	-1.33521300
C	-0.04227700	-1.43146200	-0.51253600
C	1.92571100	-0.48991700	0.78925800
H	-0.30438300	-1.84816500	-1.48879700
H	-0.12975800	-2.23821800	0.22061400
C	-1.01561700	-0.31217400	-0.16238600
O	2.40526500	-1.33051500	1.56773700
H	-0.77663100	0.09598300	0.82140500
H	-0.90955900	0.50856700	-0.88111100
C	-2.45784700	-0.80603800	-0.15716500
H	-2.72065800	-1.21225400	-1.13769600
H	-2.56172400	-1.61645100	0.57030100
C	-3.41552400	0.31060500	0.20129500
H	-3.17955000	0.74711800	1.16939500
H	-3.42225000	1.09782100	-0.54950600
N	-4.81821500	-0.20962600	0.29584700
H	-4.88428400	-0.93786300	1.01020000
H	-5.47305100	0.53525700	0.54052800
H	-5.11665600	-0.61861400	-0.59257000
O	1.78759200	0.74763600	1.04101300
Mn	1.05616300	2.26822900	-0.36133700

Molecule: Ni-LYS-01

N	1.13208800	2.84739800	-0.09741600
H	1.31076400	2.74645000	-1.10066200
H	2.02449100	3.03743800	0.36543700
H	0.51936400	3.65524000	0.04091600
C	0.48238200	1.61724800	0.45460200
H	0.36193800	1.76830400	1.52520000
C	-0.87744100	1.41636000	-0.22088200
C	1.40381200	0.44814200	0.23088800
H	-1.42717000	2.35297200	-0.10223100
H	-0.71935500	1.25877100	-1.29073700
C	-1.69260100	0.27270800	0.37098100
O	2.24164600	0.44275500	-0.72289600
H	-1.16769800	-0.67485900	0.23800500
H	-1.80769600	0.42699400	1.44701700
C	-3.06716500	0.18256800	-0.28412500
H	-3.60459300	1.12597600	-0.15250300
H	-2.95532900	0.01757800	-1.35952900
C	-3.88018300	-0.94488400	0.31609300
H	-3.40740900	-1.91252500	0.16193400
H	-4.04596800	-0.79922200	1.38137500
N	-5.23303500	-1.02001100	-0.32343900
H	-5.14958100	-1.20099100	-1.32616100
H	-5.79387600	-1.76718400	0.08940700
H	-5.73861200	-0.13934600	-0.20505600
O	1.33873700	-0.57571700	0.97611700
Ni	2.76612400	-1.41344500	-0.14276900

Molecule: Ni-LYS-022

N	2.10325900	-2.39867700	-0.84803900
H	2.13738400	-3.00607100	-0.02413100
H	3.06526400	-2.16027600	-1.10064000
H	1.68318900	-2.91846900	-1.62235800
C	1.29783200	-1.17526000	-0.54877100
H	1.37731100	-0.52064200	-1.41511300
C	-0.15711400	-1.59024900	-0.32014200
C	1.91943100	-0.50624800	0.67431500
H	-0.47554800	-2.14199200	-1.20825100
H	-0.19650300	-2.27779400	0.52867500
C	-1.09357500	-0.41199700	-0.09108500
O	2.44619400	-1.18439200	1.54144000
H	-0.83256300	0.09600000	0.84027400
H	-0.95966600	0.31504200	-0.90154100
C	-2.55759200	-0.83310100	-0.03859000
H	-2.83729000	-1.32899900	-0.97213600
H	-2.71018400	-1.54987900	0.77301400
C	-3.44723200	0.37353200	0.17757900
H	-3.22815600	0.87229400	1.11917300
H	-3.35159200	1.09343900	-0.63250900
N	-4.89069000	-0.02618200	0.22778900
H	-5.06045700	-0.68139300	0.99419600
H	-5.49065000	0.78891900	0.36765800
H	-5.17159800	-0.48344400	-0.64238600
O	1.85172100	0.79639800	0.76002400
Ni	1.12175900	2.10147000	-0.33191900

Molecule: Zn-LYS-01

N	0.87771900	2.88188600	-0.05905000
H	1.12457000	2.79052500	-1.04813100
H	1.72658900	3.13521200	0.45134900
H	0.20080500	3.64170200	0.03969200
C	0.29635600	1.60033900	0.45471200
H	0.12171600	1.73644600	1.51963900
C	-1.01920500	1.32468500	-0.27664600
C	1.31219900	0.49342200	0.25195900
H	-1.59917300	2.25051400	-0.25071600
H	-0.80528800	1.10449300	-1.32579100
C	-1.84333800	0.20548400	0.34924800
O	2.21276900	0.64081800	-0.62498000
H	-1.31023100	-0.74372200	0.27674800
H	-1.98313900	0.41386400	1.41355100
C	-3.20500000	0.07806800	-0.32623900
H	-3.73601000	1.03277900	-0.27107700
H	-3.07567100	-0.16210300	-1.38545900
C	-4.04272100	-0.99542400	0.33586100
H	-3.58410900	-1.97836000	0.25362200
H	-4.22008200	-0.77606400	1.38662900
N	-5.38859500	-1.09076600	-0.31586200
H	-5.29635500	-1.32989700	-1.30563600
H	-5.96526200	-1.80542400	0.13145600
H	-5.88217600	-0.19744200	-0.25401300
O	1.22075400	-0.55820800	0.93664400
Zn	2.98006700	-1.28502300	-0.14113900

Molecule: Zn-LYS-022

N	-1.80996500	2.50884600	-0.86811700
H	-1.70230500	3.14274400	-0.07041700
H	-2.80988800	2.37267100	-1.03122200
H	-1.40262400	2.94525800	-1.69881500
C	-1.12888100	1.21417900	-0.55844100
H	-1.45126400	0.49612800	-1.30993700
C	0.38258400	1.42763600	-0.61921900
C	-1.58163500	0.75903900	0.83492700
H	0.64160000	1.66378500	-1.65433100
H	0.63162800	2.30130500	-0.01099300
C	1.19966200	0.23448800	-0.13666300
O	-1.78829300	1.61884100	1.69401300
H	0.99662100	0.05342600	0.92108600
H	0.90432600	-0.67036800	-0.67653200
C	2.69344300	0.48524200	-0.32157500
H	2.93726800	0.52338100	-1.38672400
H	2.95503400	1.45717300	0.10731900
C	3.52355100	-0.58650200	0.35218400
H	3.33638000	-0.62310800	1.42327400
H	3.34524600	-1.57253700	-0.07121200
N	4.98511200	-0.29950700	0.18270300
H	5.22011900	0.61018300	0.58588800
H	5.55649400	-1.00968400	0.64378400
H	5.24026100	-0.27927100	-0.80721700
O	-1.67573800	-0.50576700	1.04824000
Zn	-1.64816800	-1.89951800	-0.31371200

Molecule: Cd-CYS-01

N	-1.97086000	2.33129000	-0.02644900
H	-1.50888800	2.44233000	-0.93320400
H	-1.49619000	2.93874900	0.64585900
H	-2.94270200	2.63990700	-0.11040700
C	-1.91212900	0.90179900	0.41515100
H	-2.33061500	0.85842400	1.41657800
C	-2.74234600	0.07688300	-0.56444200
C	-0.45730300	0.43648200	0.44884900
H	-3.76287100	0.45421000	-0.55403500
H	-2.33187300	0.17811800	-1.56855400
O	0.32693400	0.96970200	-0.40333800
O	-0.13830600	-0.45262500	1.25120600
Cd	2.19760500	-0.19604200	-0.10708300
S	-2.77138300	-1.69028800	-0.07998700
H	-3.81209300	-2.00375900	-0.87163400

Molecule: Cd-CYS-02

N	3.18438100	0.39877800	0.62154300
H	3.69152100	0.33110600	-0.26490600
H	3.52287700	-0.35651100	1.22137400
H	3.40115800	1.29602400	1.06169400
C	1.71276000	0.26098300	0.39469200
H	1.24879900	0.34041000	1.37660100
C	1.26441600	1.39610100	-0.53845100
C	1.44687100	-1.14330800	-0.18216100
H	1.93599900	2.24626800	-0.43483100
H	1.25570200	1.07849500	-1.57715200
O	2.42023600	-1.81701900	-0.53149800
O	0.23331900	-1.52675200	-0.26332900
Cd	-1.62047100	-0.39011700	0.11724700
S	-0.40621600	2.02579800	-0.10534300
H	-0.83740400	2.25310400	-1.36180400

Molecule: Cd-CYS-03

N	-2.13796000	1.72901900	0.04686700
H	-1.96202600	1.87436800	-0.95130500
H	-1.64079000	2.46127000	0.55873900
H	-3.14127300	1.83363900	0.22171800
C	-1.67400600	0.37641100	0.47531700
H	-1.88226500	0.29471400	1.54082400
C	-2.40425000	-0.71733400	-0.29764200
C	-0.16604100	0.27471000	0.27119100
H	-2.17044600	-0.66044000	-1.35977000
H	-2.05682000	-1.67729100	0.07461700
O	0.45829100	1.24938300	-0.20636000
O	0.37922400	-0.81850200	0.59422600
Cd	2.47009700	-0.17019100	-0.08174600
S	-4.21718900	-0.70350700	-0.07542000
H	-4.50463900	0.14611200	-1.07849800

Molecule: Cu-CYS-01

N	-1.45852300	2.34445000	0.10509100
H	-1.23266100	2.53025300	-0.87667900
H	-0.84485300	2.92389700	0.68436600
H	-2.42736200	2.62765600	0.27441600
C	-1.28729300	0.89477600	0.42639800
H	-1.52672400	0.76671400	1.47977400
C	-2.24218600	0.08778200	-0.45273300
C	0.15877400	0.51883900	0.19916900
H	-3.25060900	0.45687000	-0.28040800
H	-1.98468200	0.22915700	-1.50170600
O	0.92477200	1.21601300	-0.50321100
O	0.59499100	-0.55937800	0.72345800
Cu	2.35435200	-0.38420000	-0.08315600
S	-2.20511700	-1.69538700	-0.02721200
H	-3.45165000	-1.93916400	-0.46748000

Molecule: Cu-CYS-02

N	-2.88252200	0.72528700	0.03599800
H	-3.03295200	0.71205100	1.04937400
H	-3.45785200	-0.02022600	-0.36726000
H	-3.18932700	1.63109300	-0.32695400
C	-1.44916400	0.47441400	-0.29669200
H	-1.35082400	0.66373200	-1.36786500
C	-0.58932600	1.44010800	0.50869500
C	-1.18130300	-1.02496100	-0.07317800
H	-1.03314600	2.43414100	0.49753900
H	-0.46345000	1.10664900	1.53560300
O	-2.14169100	-1.78359400	-0.00438500
O	0.03656500	-1.43390600	-0.01322900
Cu	1.73507900	-0.65611600	0.05923000
S	1.05349500	1.60561100	-0.27931000
H	1.69175000	2.13576700	0.78682800

Molecule: Cu-CYS-33

N	-1.42893900	1.72636600	0.09550100
H	-1.27544400	1.89345900	-0.90372500
H	-0.87444100	2.41064300	0.61681200
H	-2.41785600	1.89100200	0.30390600
C	-1.05057200	0.33261000	0.47035900
H	-1.23796800	0.22580200	1.53822900
C	-1.85677800	-0.69476900	-0.32219800
C	0.42971000	0.14892000	0.23244700
H	-1.63990500	-0.61531800	-1.38634600
H	-1.55301100	-1.68343900	0.01282900
O	1.13585400	1.07433900	-0.25305700
O	0.98360300	-0.95564400	0.51866700
S	-3.65793400	-0.59027800	-0.05332500
H	-3.91720800	0.33010700	-1.00037600
Cu	2.73639700	-0.23318900	-0.11741900

Molecule: Fe-CYS-01

N	1.28432400	2.36518400	-0.08943500
H	1.08985100	2.55606600	0.89851400
H	0.62550100	2.90883900	-0.65475400
H	2.23293300	2.69071400	-0.29760800
C	1.18173400	0.90565300	-0.39090900
H	1.42452300	0.77974800	-1.44537400
C	2.18547600	0.14425700	0.48584900
C	-0.23665700	0.45688500	-0.18791300
H	3.15472300	0.62738700	0.37814800
H	1.88832600	0.20439000	1.53260000
O	-1.12311100	1.17189400	0.35541300
O	-0.59914900	-0.70280400	-0.57864000
Fe	-2.49032500	-0.44990200	0.07956500
S	2.34326900	-1.62201100	0.10171400
H	2.84477900	-1.46730400	-1.13792000

Molecule: Fe-CYS-02

N	-2.96703800	-0.04574600	0.23315100
H	-2.94359100	-0.28894600	1.22878400
H	-3.40043500	-0.82819900	-0.26526400
H	-3.55254500	0.78549100	0.11412200
C	-1.59415200	0.21021700	-0.28969000
H	-1.70409800	0.49900800	-1.33662300
C	-0.96185800	1.33477400	0.52478200
C	-0.81565200	-1.09950800	-0.24669100
H	-1.66810300	2.15915600	0.61613200
H	-0.68362900	0.98969400	1.51919600
O	-1.33275300	-2.15087200	0.04123700
O	0.45002100	-0.98446400	-0.61696500
Fe	2.03153900	-0.58187300	0.21742600
S	0.51626600	1.98678800	-0.32449000
H	0.93322300	2.75391800	0.70577700

Molecule: Fe-CYS-33

N	-1.32251100	1.70073400	0.07399700
H	-1.19368300	1.83525800	-0.93442100
H	-0.73933400	2.38697400	0.56176700
H	-2.30172300	1.89607700	0.30308700
C	-0.97758000	0.30735200	0.48022700
H	-1.16314200	0.22649100	1.55190100
C	-1.80374600	-0.72715700	-0.28796600
C	0.49321200	0.09485600	0.25772100
H	-1.55903500	-0.70136100	-1.34824800
H	-1.54689400	-1.70972700	0.10020500
O	1.23553600	1.02448800	-0.18458300
O	1.06490300	-1.01044900	0.50087700
S	-3.60300000	-0.53330400	-0.06853500
H	-3.79423200	0.35194300	-1.06449900
Fe	2.86649200	-0.22386800	-0.14697900

Molecule: Hg-CYS-01

N	-3.25705500	-1.98327700	-0.17054800
H	-3.17154200	-2.25040500	0.81422400
H	-2.84545300	-2.73353700	-0.73046600
H	-4.24961500	-1.90738200	-0.40668400
C	-2.55876200	-0.68636300	-0.42452400
H	-2.66773800	-0.46504900	-1.48307600
C	-3.21853400	0.39227600	0.43133800
C	-1.07693700	-0.86865300	-0.08283400
H	-4.28789500	0.37936800	0.23350600
H	-3.05164500	0.18002800	1.48695800
O	-0.70215700	-1.81633000	0.59687000
O	-0.35483300	0.08686000	-0.55970200
Hg	1.72351600	0.07193600	0.01055100
S	-2.59360300	2.06487600	0.01492400
H	-3.72901000	2.69923500	0.35529400

Molecule: Hg-CYS-02

N	3.48559700	0.36867000	0.66749300
H	4.02068500	0.27927600	-0.20078700
H	3.78966400	-0.38391200	1.28964500
H	3.70390500	1.26791500	1.10333400
C	2.02073100	0.25341300	0.39497100
H	1.52691200	0.32854600	1.36236000
C	1.61292500	1.40119400	-0.54410900
C	1.75874100	-1.14256400	-0.20271000
H	2.29883200	2.23836700	-0.42806200
H	1.60999500	1.08713200	-1.58378200
O	2.72882200	-1.82065200	-0.54225800
O	0.54367600	-1.53145300	-0.31507300
Hg	-1.23306100	-0.24078600	0.08041000
S	-0.04874400	2.08192500	-0.14183600
H	-0.45877700	2.29861800	-1.40887000

Molecule: Hg-CYS-03

N	-2.97846900	1.73287600	0.16509000
H	-2.87051100	1.97708300	-0.82366600
H	-2.58029200	2.49648900	0.71636500
H	-3.97631200	1.66604400	0.38317800
C	-2.28792300	0.44569900	0.46840600
H	-2.40821300	0.25893900	1.53261300
C	-2.87790100	-0.69821100	-0.35369800
C	-0.80100200	0.61068800	0.14019700
H	-2.72213900	-0.52769500	-1.41772600
H	-2.35526100	-1.61037600	-0.07607700
O	-0.41703900	1.55728600	-0.53908100
O	-0.07621900	-0.33822900	0.62077000
Hg	1.96297000	-0.15000200	-0.02601200
S	-4.64935100	-1.01054700	-0.04038900
H	-5.13896700	-0.12321900	-0.92605400

Molecule: Mn-CYS-011

N	-1.32929800	2.34548000	0.09016100
H	-0.97097600	2.51231300	-0.85399500
H	-0.75538900	2.88894100	0.73887700
H	-2.29300600	2.68376600	0.14521300
C	-1.25876600	0.88708900	0.42088800
H	-1.55790300	0.78238900	1.46060100
C	-2.22334600	0.14397700	-0.49791600
C	0.18183100	0.41322800	0.25706000
H	-3.21251700	0.58380600	-0.39031000
H	-1.89942500	0.24513500	-1.53333700
O	0.96668300	1.10763900	-0.44417600
O	0.50405200	-0.66173100	0.81609100
S	-2.34173900	-1.62879700	-0.04515000
H	-3.57794200	-1.79064800	-0.54813400
Mn	2.76303500	-0.41985300	-0.11932600

Molecule: Mn-CYS-24

N	-2.86368000	0.57852500	-0.27589000
H	-3.18931600	0.60079100	0.69436800
H	-3.33602300	-0.20618300	-0.73227900
H	-3.12790900	1.45474500	-0.73075500
C	-1.38499500	0.36428300	-0.33470600
H	-1.10893000	0.48320200	-1.38323200
C	-0.69616400	1.41003800	0.54348500
C	-1.10310900	-1.09401400	0.06893000
H	-1.26655300	2.33665900	0.53057800
H	-0.60798700	1.06029000	1.56861400
O	-2.06303300	-1.86201000	0.18298500
O	0.11433300	-1.41124200	0.24866100
S	0.97668300	1.80806500	-0.09878900
H	1.50069100	2.21681500	1.07219900
Mn	2.01000300	-0.75283500	-0.10508300

Molecule: Mn-CYS-31

N	1.76271800	-1.75583700	-0.60905700
H	1.61580900	-1.23744200	-1.47868900
C	1.26310200	-0.97868100	0.56770700
H	1.30482300	-1.64987700	1.42450700
C	-0.18703300	-0.57857900	0.32010800
O	-0.80975200	-0.04302000	1.27297000
C	2.15888100	0.22113100	0.84733900
H	1.83008900	0.67335800	1.78008600
H	3.18951900	-0.10363700	0.97807300
H	2.75843700	-1.96914500	-0.51044700
H	1.24887300	-2.63510700	-0.69725700
O	-0.68213700	-0.78612300	-0.81765300
S	2.08676000	1.55805900	-0.39531200
H	2.81049300	0.94734500	-1.35210200
Mn	-2.71839200	0.31945300	-0.14437000

Molecule: Ni-CYS-01

N	-1.21018300	2.38443700	0.10114900
H	-0.99153800	2.55308000	-0.88556700
H	-0.52994800	2.89831300	0.66840500
H	-2.14231000	2.76090200	0.29397800
C	-1.18033200	0.92367300	0.41731100
H	-1.44528400	0.81333000	1.46696500
C	-2.19315800	0.20774900	-0.47704300
C	0.22186100	0.40980400	0.22367900
H	-3.15802100	0.68899300	-0.33461100
H	-1.89536500	0.30572700	-1.52012000
O	1.09623500	1.07943700	-0.40625600
O	0.55068300	-0.72850400	0.67535500
Ni	2.35593200	-0.43402300	-0.09645900
S	-2.37090000	-1.56096700	-0.02778600
H	-3.66349900	-1.63810600	-0.38813500

Molecule: Ni-CYS-02

N	-2.80068700	0.84667700	-0.12513500
H	-3.05286800	0.84806500	0.86809000
H	-3.39271200	0.15110000	-0.58776200
H	-2.99443000	1.77321200	-0.51274400
C	-1.36522200	0.48732300	-0.31278500
H	-1.15540800	0.62867100	-1.37478900
C	-0.51140900	1.41830100	0.54604800
C	-1.20773800	-1.01303100	-0.00804000
H	-0.93108400	2.42237400	0.54883600
H	-0.42236800	1.05747400	1.56770500
O	-2.21381200	-1.69645100	0.12501700
O	-0.01200600	-1.49057500	0.05774700
Ni	1.69501300	-0.70771200	-0.01468300
S	1.16295900	1.57207600	-0.18536000
H	1.79871000	1.99571800	0.93004100

Molecule: Ni-CYS-03

N	-1.36299900	1.72358700	0.10487500
H	-1.22877200	1.89723800	-0.89630100
H	-0.78796300	2.39498300	0.62128800
H	-2.34473600	1.90226100	0.33526600
C	-1.00452600	0.32077600	0.46123100
H	-1.18539700	0.20385300	1.52956400
C	-1.83052100	-0.68778800	-0.33717800
C	0.46870200	0.11459900	0.22087400
H	-1.62358400	-0.59601900	-1.40221000
H	-1.53387800	-1.68397000	-0.01805600
O	1.19951900	1.03682100	-0.25397200
O	1.01302700	-0.99787800	0.49597300
Ni	2.73740400	-0.22570600	-0.11503700
S	-3.62619200	-0.56463200	-0.04462500
H	-3.88522500	0.37337800	-0.97421800

Molecule: Zn-CYS-02

N	-2.84084800	0.77344000	-0.28649400
H	-3.17410500	0.77327900	0.68167800
H	-3.38324600	0.06804200	-0.79086400
H	-3.01604800	1.69432200	-0.69488900
C	-1.38721700	0.43390700	-0.34792000
H	-1.09726600	0.53933800	-1.39399700
C	-0.63284200	1.42406700	0.54470900
C	-1.22765300	-1.04519000	0.05260600
H	-1.13103100	2.39099700	0.52635600
H	-0.57732300	1.07018700	1.57054800
O	-2.24483500	-1.69461900	0.28909300
O	-0.04103400	-1.52472800	0.09765500
Zn	1.71013800	-0.68672000	-0.07869100
S	1.06913800	1.71848100	-0.07703400
H	1.62783600	2.03372700	1.10954300

Molecule: Zn-CYS-11

N	-1.41306000	2.36439900	0.08505900
H	-1.19764300	2.54527300	-0.89932100
H	-0.75747300	2.90847500	0.65103600
H	-2.36337200	2.69160700	0.27621100
C	-1.29757800	0.90536300	0.39506500
H	-1.56268100	0.78315300	1.44332000
C	-2.27036200	0.14082300	-0.50063600
C	0.14279000	0.46942500	0.20225300
H	-3.25100900	0.60196100	-0.40960700
H	-1.94527500	0.20462400	-1.53835900
O	0.98797800	1.25269200	-0.29767100
O	0.44332400	-0.71017000	0.54090200
S	-2.43292200	-1.61516400	0.00308200
H	-3.73279200	-1.71028600	-0.32637700
Zn	2.42429700	-0.40556100	-0.07891900

Molecule: Zn-CYS-33

N	-1.60871600	1.73173300	0.11476400
H	-1.48625200	1.93597800	-0.88119100
H	-1.07841400	2.43141000	0.63930700
H	-2.59928600	1.83199200	0.35188800
C	-1.12857200	0.35587800	0.44020400
H	-1.28002000	0.21347200	1.50897000
C	-1.90127000	-0.69188900	-0.35388700
C	0.36578400	0.27963000	0.15470500
H	-1.73172100	-0.56959700	-1.42260800
H	-1.53235200	-1.67208100	-0.06250600
O	0.96039700	1.25351700	-0.33955200
O	0.94828600	-0.81279500	0.45639000
S	-3.69613100	-0.70757500	-0.01959100
H	-4.04126000	0.26746900	-0.88071600
Zn	2.82877700	-0.28090200	-0.07079600

Molecule: Cd-SER-01

N	2.40354400	-1.84929000	0.01801800
H	1.91109300	-2.03857100	-0.85953000
H	2.01858000	-2.47105700	0.73220500
H	3.39379900	-2.07713200	-0.10418600
C	2.23144700	-0.41819100	0.41055900
H	2.69334100	-0.28763700	1.38510100
C	2.93453300	0.44172700	-0.63700500
C	0.74165300	-0.08568100	0.49643600
H	3.98143600	0.12785900	-0.69325600
H	2.46652800	0.27846900	-1.61283900
O	2.82504300	1.79689200	-0.23291600
O	0.00511600	-0.67490200	-0.37007300
H	3.23707200	2.34334000	-0.91417700
O	0.34771000	0.72787500	1.33635000
Cd	-2.02907100	0.05507700	-0.11554700

Molecule: Cd-SER-02

N	3.11200200	0.57342700	0.52987000
H	3.58455300	0.51752400	-0.37615200
H	3.49680100	-0.17018000	1.11578000
H	3.32269900	1.47827900	0.95718100
C	1.64062300	0.39314600	0.37171200
H	1.19906500	0.51683100	1.36020700
C	1.10752700	1.48774800	-0.55447000
C	1.36740200	-1.03354000	-0.13201200
H	1.65864300	2.41287300	-0.37524300
H	1.21997600	1.20112200	-1.60044300
O	-0.27719100	1.71746900	-0.24492200
O	2.33688000	-1.74720200	-0.41386400
H	-0.66502900	2.25396800	-0.95058300
O	0.14690300	-1.39603500	-0.23098500
Cd	-1.62389100	-0.12296600	0.10764500

Molecule: Cd-SER-03

N	-2.63859500	1.48283800	-0.01183500
H	-2.33780600	1.68536000	-0.96889800
H	-2.34721400	2.26251900	0.58017700
H	-3.65941400	1.40906700	0.00194400
C	-2.04800500	0.19445600	0.45924400
H	-2.29250800	0.09966500	1.51648200
C	-2.67242000	-0.95221500	-0.32234600
C	-0.53816700	0.22455200	0.29436000
H	-2.41380300	-0.86346200	-1.38081400
H	-2.26947700	-1.88934300	0.06298600
O	-4.08231000	-0.87629000	-0.13705600
O	0.01021500	1.23807200	-0.18979200
H	-4.51130700	-1.29574100	-0.89278400
O	0.09173100	-0.81783400	0.64227100
Cd	2.11867000	-0.10292200	-0.08223300

Molecule: Cd-SER-04

N	1.19948300	0.06144000	1.98625100
H	0.19582900	-0.13355000	1.94055500
H	1.32032800	0.98072300	2.41704500
H	1.63383400	-0.63597600	2.59635600
C	1.81587500	0.02545700	0.62536700
H	2.87265100	0.24421900	0.74997400
C	1.63652300	-1.38168700	0.06671400
C	1.20206300	1.11628400	-0.26357600
H	2.16792200	-2.08484800	0.71466700
H	0.57258300	-1.64876500	0.07730300
O	2.15830800	-1.39572900	-1.25056300
O	-0.06244400	1.32775400	-0.17689400
H	2.11876000	-2.30349500	-1.57706300
O	1.96111600	1.75171900	-0.99539800
Cd	-1.65960200	-0.14330600	-0.08355800

Molecule: Cu-SER-01

N	1.99247700	-1.78058900	0.10240200
H	1.74431000	-2.00064600	-0.86720700
H	1.52196300	-2.45332200	0.71358800
H	3.00299600	-1.90507300	0.21262300
C	1.60829600	-0.38116800	0.44966000
H	1.83863000	-0.22299400	1.50002900
C	2.41346300	0.57467800	-0.42978800
C	0.12482700	-0.20622100	0.22930300
H	3.47624700	0.39243100	-0.24395800
H	2.19571900	0.36783600	-1.48158200
O	2.03827200	1.89323100	-0.07196000
O	-0.45090900	-0.74406700	-0.76096200
H	2.43480500	2.49992400	-0.70987900
O	-0.56599700	0.49746800	1.02139300
Cu	-2.17974000	0.09273000	-0.09804300

Molecule: Cu-SER-02

N	-2.71754600	0.68285500	-0.21284200
H	-2.99507300	0.72863500	0.77209200
H	-3.22285200	-0.10296100	-0.63086400
H	-2.99795500	1.55188400	-0.67301500
C	-1.25221600	0.45581400	-0.34775400
H	-1.00469600	0.57185000	-1.40369800
C	-0.50507800	1.51084400	0.45651700
C	-0.95372700	-0.99784000	0.04886500
H	-0.94927600	2.49114600	0.28528900
H	-0.52024800	1.27978400	1.52176100
O	0.85503800	1.54479300	-0.02511500
O	-1.88745400	-1.77697300	0.21552000
H	1.37106300	2.13866600	0.54246000
O	0.28331900	-1.33780300	0.16072200
Cu	1.77933700	-0.23076000	-0.09237600

Molecule: Cu-SER-03

N	1.83495900	1.53693500	-0.12039700
H	1.52739200	1.77440400	0.82684300
H	1.41406400	2.20519400	-0.76894700
H	2.85422500	1.62886900	-0.16170600
C	1.46677300	0.13220200	-0.46572400
H	1.72148300	-0.01640200	-1.51486800
C	2.27772100	-0.82285700	0.40173700
C	-0.01757200	-0.08341100	-0.29683600
H	2.03239900	-0.66439700	1.45439100
H	2.01348900	-1.84455500	0.12574700
O	3.65300200	-0.55694200	0.15325400
O	-0.72887600	0.87575600	0.16848100
H	4.14320600	-0.68933300	0.97368900
O	-0.54318200	-1.16975300	-0.59727600
Cu	-2.41241600	-0.05863500	0.14747900

Molecule: Cu-SER-04

N	0.73956600	-0.06713500	1.98664800
H	-0.24837900	-0.33244600	1.94499400
H	0.81274100	0.83552800	2.46234500
H	1.22781600	-0.76595200	2.55448200
C	1.34536600	-0.00695300	0.62166700
H	2.38958400	0.25903200	0.75627800
C	1.23625500	-1.38237600	-0.02592400
C	0.70485300	1.11057600	-0.21554500
H	1.86452200	-2.08237000	0.53264900
H	0.20478500	-1.75254800	0.01571300
O	1.67910100	-1.24168200	-1.36601800
O	-0.57713800	1.11283000	-0.39554200
H	1.58057000	-2.09391300	-1.80921600
O	1.42865500	1.97840500	-0.68721900
Cu	-1.82663400	-0.23176900	-0.10533600

Molecule: Fe-SER-01

N	-1.84286000	1.82763900	0.10529700
H	-1.59552100	2.06350900	-0.86130600
H	-1.33515100	2.46496800	0.72521300
H	-2.84604000	1.99394200	0.22692900
C	-1.52652400	0.40386000	0.41520700
H	-1.79688000	0.21704900	1.45335100
C	-2.35051300	-0.50713000	-0.50666500
C	-0.05346000	0.18923700	0.23717300
H	-3.37968500	-0.13522000	-0.49592600
H	-1.96724400	-0.44640200	-1.52758900
O	-2.27023300	-1.82266900	0.00550400
O	0.65738400	1.03141100	-0.39402600
H	-2.40626600	-2.44018200	-0.72385500
O	0.54774700	-0.84050100	0.67502500
Fe	2.32040800	-0.15280200	-0.10385400

Molecule: Fe-SER-02

N	-2.72412800	0.63252700	-0.16692800
H	-2.98095400	0.59302400	0.82480200
H	-3.25334700	-0.09708800	-0.65194800
H	-3.00698100	1.54490800	-0.53706800
C	-1.26376400	0.43119500	-0.35684900
H	-1.05231100	0.55707500	-1.41984700
C	-0.51856400	1.50038600	0.42790600
C	-0.89850200	-1.00513700	0.02169600
H	-0.93042100	2.48423600	0.21215000
H	-0.53930200	1.30504400	1.49969300
O	0.84715600	1.48904000	-0.04457900
O	-1.75298900	-1.83660900	0.23610200
H	1.35540600	2.21004600	0.36583700
O	0.38938700	-1.28459000	0.06312300
Fe	1.91128300	-0.21255100	-0.06610800

Molecule: Fe-SER-03

N	1.44675100	1.50941800	-0.29602400
H	1.02677900	1.77161000	0.59986400
H	0.92943300	1.98121800	-1.04040200
H	2.41954000	1.83251700	-0.30466700
C	1.44658300	0.02803700	-0.47781100
H	1.77667800	-0.16413600	-1.49896700
C	2.43678700	-0.58897400	0.50541400
C	0.05648100	-0.56257200	-0.32291500
H	2.10845400	-0.40488500	1.53040500
H	2.46889500	-1.66420500	0.32793300
O	3.70173400	0.01220500	0.25801500
O	-0.87990000	0.34719400	-0.10066200
H	4.14267800	0.16476800	1.10210700
O	-0.14628400	-1.75120700	-0.40724400
Fe	-2.69397000	0.14587300	0.19718800

Molecule: Hg-SER-01

N	-3.44499200	1.36140500	0.09887200
H	-3.15086500	1.73128900	-0.80963700
H	-3.34404400	2.10892200	0.78748500
H	-4.42887200	1.07994100	0.04941800
C	-2.61575500	0.17551500	0.46149400
H	-2.75011600	-0.00301400	1.52616800
C	-3.09957400	-1.02762200	-0.33856100
C	-1.15133900	0.48157400	0.15886500
H	-2.92500100	-0.85902700	-1.40397300
H	-2.53133400	-1.90217900	-0.01892400
O	-4.48891600	-1.18573500	-0.07148200
O	-0.85110300	1.48585600	-0.47873500
H	-4.90479200	-1.58509700	-0.84529200
O	-0.34547400	-0.42095400	0.60366100
Hg	1.68542400	-0.08638500	-0.02619600

Molecule: Hg-SER-02

N	-3.50091200	0.42025400	-0.62522800
H	-4.01862300	0.30109000	0.24962600
H	-3.79149900	-0.33320900	-1.25193400
H	-3.75822600	1.31772600	-1.04291700
C	-2.03082700	0.35622300	-0.37951700
H	-1.53869300	0.50832600	-1.33889900
C	-1.63417600	1.48990200	0.57308000
C	-1.69685700	-1.03610000	0.16235100
H	-2.29611600	2.34259900	0.40529200
H	-1.72437200	1.17017300	1.61132500
O	-0.28882800	1.88587700	0.28250300
O	-2.62084400	-1.79095600	0.46774400
H	0.07724400	2.31717700	1.06683300
O	-0.45923800	-1.36619900	0.28791900
Hg	1.25848900	-0.06569500	-0.07204400

Molecule: Hg-SER-41

N	-1.49982900	-0.24644300	-1.98353800
H	-0.50999100	-0.50122700	-1.92312200
H	-1.57188800	0.62700100	-2.51147700
H	-1.97654300	-0.97922100	-2.51681400
C	-2.13353900	-0.10653900	-0.63717500
H	-3.18881800	0.07448400	-0.82127800
C	-1.96606100	-1.40401700	0.13666700
C	-1.61394700	1.12029900	0.12811800
H	-2.41465100	-2.21850600	-0.43967400
H	-0.90203200	-1.62596000	0.27952300
O	-2.62395400	-1.22312600	1.37996000
O	-0.35833600	1.26319300	0.40617900
H	-2.55280500	-2.04495900	1.88132700
O	-2.43821500	1.95443200	0.48939200
Hg	1.26576100	-0.06526200	0.04958000

Molecule: Mn-SER-1

N	-1.93856300	1.79493100	0.20931100
H	-1.68507100	2.06585700	-0.74526700
H	-1.42637000	2.40653600	0.84829700
H	-2.94000200	1.95063800	0.34824400
C	-1.57744100	0.36562000	0.44905400
H	-1.73171500	0.16224600	1.50579900
C	-2.50558400	-0.50433400	-0.39335300
C	-0.10207000	0.17915200	0.10183900
H	-3.53887500	-0.24746100	-0.13863800
H	-2.34396400	-0.29204800	-1.45516300
O	-2.22930800	-1.86294200	-0.09184200
O	0.41645100	0.94918000	-0.74073700
H	-2.83894200	-2.40802500	-0.60535300
O	0.52248500	-0.74050800	0.69931400
Mn	2.62033700	-0.12842900	-0.04408900

Molecule: Mn-SER-2

N	-2.64384600	0.63428300	-0.49668200
H	-3.11397400	0.53911600	0.40699000
H	-3.05289400	-0.06245800	-1.12157200
H	-2.82846700	1.56833500	-0.86838900
C	-1.17523000	0.40020300	-0.35229000
H	-0.73963700	0.53045600	-1.34197600
C	-0.58839600	1.45582900	0.58915300
C	-0.95584200	-1.04969200	0.11272800
H	-1.21962800	2.34771700	0.56986100
H	-0.54616300	1.08204400	1.61308100
O	0.72175600	1.79704400	0.12523600
O	-1.95888200	-1.73084400	0.38018200
H	1.15998600	2.31627300	0.81299200
O	0.24548900	-1.44857800	0.19154800
Mn	2.12390400	-0.26162000	-0.17069900

Molecule: Mn-SER-3

N	2.27718300	1.38437300	-0.14772400
H	1.91429500	1.80273400	0.71467700
H	2.19473900	2.08224700	-0.88833100
H	3.26343000	1.13702500	-0.03034900
C	1.48178900	0.16012600	-0.46216100
H	1.60143800	-0.04635600	-1.52328700
C	2.02260100	-0.99793800	0.36209800
C	0.01110900	0.41893900	-0.13298100
H	1.88405700	-0.79228300	1.42671600
H	1.46330400	-1.89712000	0.09933600
O	3.40734300	-1.13951000	0.05248200
O	-0.27701100	1.41057400	0.55569000
H	3.84376300	-1.56852800	0.79877500
O	-0.80087200	-0.45214600	-0.57976400
Mn	-2.87335900	-0.25785700	0.06430100

Molecule: Mn-SER-41

N	0.67272300	0.17829900	1.97909200
H	-0.34376800	0.13749400	1.85797100
H	0.89879800	1.06471600	2.43461000
H	0.96596200	-0.58348800	2.59569700
C	1.33394600	0.08457700	0.64136800
H	2.38201100	0.33514100	0.77671900
C	1.19392200	-1.35312200	0.15015500
C	0.68778100	1.10454200	-0.31286200
H	1.75281200	-2.00850700	0.82485600
H	0.14264200	-1.65835000	0.18240700
O	1.70477000	-1.41620000	-1.17094100
O	-0.57429900	1.27475200	-0.20447700
H	1.63448100	-2.33076800	-1.47166200
O	1.42767300	1.69405500	-1.10630300
Mn	-2.07604200	-0.30564700	-0.16289800

Molecule: Ni-SER-01

N	-1.90180000	1.80854400	0.13547000
H	-1.67652500	2.03865700	-0.83731500
H	-1.38465000	2.45167400	0.74091700
H	-2.90269300	1.96856200	0.28138200
C	-1.56013100	0.38992400	0.44519800
H	-1.78489300	0.21363400	1.49454000
C	-2.41539200	-0.51721100	-0.44153400
C	-0.08486700	0.18022800	0.21704100
H	-3.46476300	-0.26861900	-0.25496100
H	-2.18474500	-0.31843100	-1.49221500
O	-2.12858300	-1.85838000	-0.09034800
O	0.54828800	0.87606000	-0.63498700
H	-2.57265900	-2.43344800	-0.72659200
O	0.55276900	-0.69676000	0.87007000
Ni	2.20950200	-0.11417300	-0.09414800

Molecule: Ni-SER-02

N	-2.67816800	0.71783300	-0.22707200
H	-2.95942000	0.77787900	0.75611700
H	-3.19817300	-0.06071800	-0.64090000
H	-2.93828500	1.58817400	-0.69682400
C	-1.21779000	0.45967500	-0.35338900
H	-0.96123800	0.57030500	-1.40790700
C	-0.45884000	1.50327100	0.45707700
C	-0.94833300	-0.99800000	0.04736900
H	-0.87022000	2.49352400	0.26717700
H	-0.49431400	1.28236300	1.52368200
O	0.91487400	1.50538000	0.00095500
O	-1.89307700	-1.74899100	0.25703100
H	1.42426300	2.14368000	0.52593500
O	0.28681300	-1.36657600	0.11253400
Ni	1.78662400	-0.24029400	-0.09315300

Molecule: Ni-SER-03

N	1.88207500	1.54227100	-0.02787300
H	1.64542800	1.74197000	0.94840800
H	1.46814500	2.27165000	-0.61300300
H	2.90095400	1.57788400	-0.12577000
C	1.40891400	0.18548700	-0.42800400
H	1.61799000	0.07228900	-1.49252200
C	2.17056900	-0.87266900	0.35791700
C	-0.07616100	0.07658900	-0.22647100
H	1.99368100	-0.73482300	1.42792300
H	1.79978700	-1.85445500	0.05988000
O	3.54485200	-0.70918900	0.03752200
O	-0.76056800	1.05992300	0.18765600
H	4.07201800	-1.10606000	0.74207200
O	-0.67374800	-1.01422400	-0.46942000
Ni	-2.37774100	-0.13546000	0.10647900

Molecule: Ni-SER-04

N	0.85874000	-0.04701100	1.99671600
H	-0.08639600	-0.44184500	1.98858000
H	0.82470400	0.84845100	2.49115500
H	1.46348600	-0.68128300	2.52621500
C	1.39095800	0.12641300	0.61058300
H	2.39119600	0.54021700	0.70357100
C	1.44916300	-1.24361400	-0.06051700
C	0.54951700	1.12428000	-0.18573400
H	2.07832600	-1.90010200	0.54844200
H	0.44285900	-1.67742300	-0.10913300
O	1.98908500	-1.05414900	-1.35592900
O	-0.75872800	1.02472400	-0.09678100
H	1.94977800	-1.89765800	-1.82443900
O	1.08856400	1.97845800	-0.85832100
Ni	-1.92729700	-0.36057200	-0.14282700

Molecule:Zn-SER-01

N	-2.51820700	1.49846200	0.28149200
H	-2.42250500	1.81994400	-0.68668700
H	-2.16741900	2.23965900	0.89183300
H	-3.51000100	1.35434400	0.48761700
C	-1.74123700	0.24030800	0.47783600
H	-1.75072500	0.00762300	1.53882500
C	-2.42579200	-0.86665800	-0.32017500
C	-0.31161500	0.47643700	-0.01168700
H	-3.46658500	-0.93767200	0.01044500
H	-2.41228500	-0.60812700	-1.38350400
O	-1.72717900	-2.07709400	-0.07512300
O	-0.11849400	1.24334300	-0.95718800
H	-2.11997700	-2.76293500	-0.62961300
O	0.57708100	-0.19573300	0.62244200
Zn	2.41658500	-0.08222400	0.00679100

Molecule: Zn-SER-02

N	-2.75154400	0.68470200	-0.18822600
H	-2.98645800	0.83334700	0.79736900
H	-3.26095600	-0.15065700	-0.49049100
H	-3.06246700	1.49789000	-0.72310400
C	-1.28792000	0.44811900	-0.36083100
H	-1.06653000	0.56646900	-1.42172300
C	-0.54127800	1.52045100	0.43202700
C	-0.99858600	-1.01765300	0.02122000
H	-1.02010000	2.48564700	0.26579700
H	-0.54601200	1.28904600	1.49796500
O	0.81157300	1.59548600	-0.05650000
O	-1.95910600	-1.74869800	0.27751600
H	1.28511800	2.28054400	0.43689400
O	0.21924800	-1.40981400	0.00883100
Zn	1.81037300	-0.22655000	-0.04794700

Molecule: Zn-SER-03

N	1.76390900	1.54354700	-0.30033300
H	1.27013000	1.84455900	0.54340000
H	1.38265200	2.06869200	-1.08914000
H	2.75848900	1.76515200	-0.20193900
C	1.60064100	0.07276100	-0.50517600
H	1.99283500	-0.15602000	-1.49458600
C	2.40515600	-0.66407800	0.55325200
C	0.13146900	-0.33487700	-0.44732100
H	2.03578200	-0.40165900	1.54856700
H	2.27334600	-1.73573900	0.39787300
O	3.76787200	-0.27885000	0.40026700
O	-0.68089200	0.58222800	-0.06196100
H	4.22710800	-0.44168000	1.23363900
O	-0.16088500	-1.49317500	-0.74365400
Zn	-2.55066900	0.04424700	0.22675900

Molecule: Zn-SER-04

N	0.70179400	-0.14355700	1.99143200
H	-0.29482200	-0.36244200	1.92014200
H	0.80160100	0.73584700	2.50387600
H	1.14539200	-0.88428900	2.54209400
C	1.34956700	-0.05190800	0.64463400
H	2.39734800	0.16837100	0.82694200
C	1.22089300	-1.39742100	-0.05681100
C	0.77698300	1.11648000	-0.18003000
H	1.78628200	-2.14348400	0.50973700
H	0.17576700	-1.72703500	-0.09206400
O	1.75070700	-1.23100500	-1.36283900
O	-0.48776300	1.16743300	-0.40474100
H	1.66243300	-2.06891000	-1.83434800
O	1.57039600	1.95832400	-0.59752900
Zn	-1.84459800	-0.19580300	-0.12807600

Molecule: Cd-PHE-01

N	0.00310600	3.33195000	-0.04440200
H	-0.41077300	3.38261800	0.89031300
H	-0.72329400	3.58152700	-0.71949400
H	0.75701600	4.02008600	-0.10761400
C	0.53013200	1.95521000	-0.30481500
H	0.88054200	1.93909800	-1.33322700
C	1.68850300	1.67819700	0.66371500
C	-0.61330900	0.96322800	-0.15144900
H	2.38349800	2.51655300	0.57486400
H	1.30012200	1.67214300	1.68319100
C	2.40704000	0.38973000	0.36792900
O	-1.61499600	1.28994300	0.53099000
C	2.27124900	-0.71098700	1.21041500
C	3.21335600	0.27733800	-0.76554900
H	1.64621800	-0.63167900	2.09130300
C	2.92787300	-1.90448000	0.92761500
C	3.86675800	-0.91385800	-1.05314900
H	3.32721800	1.12889800	-1.42571700
H	2.81646000	-2.75074000	1.59341600
C	3.72450300	-2.01001800	-0.20674700
H	4.48950300	-0.98691400	-1.93566800
H	4.23587500	-2.93787000	-0.42859200
O	-0.49436500	-0.15961300	-0.71753700
Cd	-2.58220600	-0.86762800	-0.06682000

Molecule: Cd-PHE-02

N	-3.35201000	0.63937900	-1.04150500
H	-3.87528400	1.09410100	-0.28700900
H	-3.80135700	-0.25863900	-1.23192100
H	-3.40472700	1.21574300	-1.88454300
C	-1.93142000	0.44884000	-0.61299000
H	-1.44674300	-0.17005600	-1.36494800
C	-1.25605600	1.82764300	-0.52941300
C	-1.95113400	-0.26812400	0.74488300
H	-1.39285900	2.31937600	-1.49433500
H	-1.76999800	2.41945200	0.22997700
C	0.20844200	1.71813700	-0.21099900
O	-2.88505900	-0.01050500	1.51342700
C	0.66308000	1.81396500	1.10283300
C	1.12743300	1.43270000	-1.22496900
H	-0.04315900	2.03367800	1.89388200
C	2.00475400	1.60881800	1.40525800
C	2.46914300	1.21319300	-0.92267300
H	0.78641100	1.36536100	-2.25080200
H	2.34202700	1.68343100	2.43100800
C	2.90904100	1.29740700	0.39620000
H	3.16863200	0.98281800	-1.71568800
H	3.95122500	1.12672400	0.63240600
O	-0.99854400	-1.07372800	1.02952300
Cd	0.71998000	-1.58685900	-0.18541400

Molecule: Cu-PHE-01

N	-1.23900900	2.96648100	0.01444800
H	-1.60036600	2.90519800	0.97045500
H	-2.03862600	3.02919500	-0.62092100
H	-0.69273700	3.82795500	-0.06900400
C	-0.37904900	1.78600700	-0.30552700
H	-0.05075400	1.89740400	-1.33625900
C	0.82657000	1.76954400	0.64836900
C	-1.21391900	0.53589100	-0.19873000
H	1.32397900	2.73578300	0.53837300
H	0.45798100	1.69483300	1.67243300
C	1.79203200	0.65197500	0.35943700
O	-2.29160400	0.51644600	0.45619800
C	1.91749200	-0.41711100	1.24352900
C	2.56466200	0.66317700	-0.80238100
H	1.31946900	-0.43355200	2.14645800
C	2.80095500	-1.45753400	0.97551300
C	3.44358600	-0.37702500	-1.07527000
H	2.47550700	1.49002100	-1.49685400
H	2.89210900	-2.27948100	1.67405500
C	3.56441800	-1.44120600	-0.18583100
H	4.03672500	-0.35654100	-1.98062600
H	4.25175000	-2.25025300	-0.39690200
O	-0.83182100	-0.52881400	-0.77451200
Cu	-2.43500200	-1.48997600	-0.09001700

Molecule: Cu-PHE-02

N	-2.97762700	1.71138600	-0.22726900
H	-3.40715400	1.70801200	0.70197700
H	-3.67787200	1.36692200	-0.88725600
H	-2.73499900	2.67417900	-0.47187500
C	-1.76058900	0.84451200	-0.24764800
H	-1.35149600	0.91017000	-1.25416800
C	-0.74703900	1.37294400	0.78363500
C	-2.21771100	-0.59569500	0.00839200
H	-0.81880500	2.46219600	0.78332700
H	-1.02395700	1.02258600	1.77741400
C	0.67811500	1.00387800	0.47065000
O	-3.39314100	-0.81513900	0.28723400
C	1.35639700	0.02666900	1.23280900
C	1.36615200	1.63544300	-0.57524000
H	0.85756900	-0.42457100	2.08153000
C	2.70230900	-0.27252000	0.96784600
C	2.68858800	1.32567000	-0.83549500
H	0.85508900	2.38214400	-1.16911700
H	3.21339300	-1.00530800	1.57669600
C	3.36172900	0.37152400	-0.06604600
H	3.20779600	1.82829200	-1.64052500
H	4.39767800	0.14206800	-0.27510200
O	-1.34101700	-1.53657800	-0.09585300
Cu	0.50454200	-1.39680000	-0.39987900

Molecule: Fe-PHE-011

N	-1.32964900	2.94766300	0.01792800
H	-1.72928900	2.84453400	0.95586700
H	-2.09905200	3.03672100	-0.65182200
H	-0.78814400	3.81721800	-0.00108500
C	-0.43617900	1.79894900	-0.31979700
H	-0.05657000	1.95880100	-1.32561500
C	0.71741600	1.72953900	0.70237900
C	-1.22940200	0.52675000	-0.29385900
H	1.22453900	2.69441500	0.65804700
H	0.28436000	1.61434600	1.69717400
C	1.68233300	0.61278800	0.41124500
O	-2.14016000	0.32315400	0.57508100
C	1.53422000	-0.63016600	1.02577600
C	2.72325400	0.80002300	-0.49760600
H	0.72824300	-0.78187400	1.73372900
C	2.40941600	-1.67035300	0.73530000
C	3.60187800	-0.23694000	-0.78608400
H	2.84797300	1.76462500	-0.97447500
H	2.28424800	-2.63034100	1.21940100
C	3.44588200	-1.47535800	-0.17123000
H	4.41084500	-0.07748200	-1.48733800
H	4.13113900	-2.28271800	-0.39483200
O	-0.98783600	-0.42826400	-1.08241100
Fe	-2.44614200	-1.55701600	-0.08971700

Molecule: Fe-PHE-021

N	3.16995200	-1.06600700	-0.74806000
H	3.66168100	-1.21964200	0.13744700
H	3.69965600	-0.37449700	-1.28492700
H	3.15813400	-1.94452900	-1.27278800
C	1.77386600	-0.59549400	-0.49164800
H	1.34443600	-0.32279100	-1.45190900
C	0.97628600	-1.74528400	0.15539300
C	1.87599100	0.63264700	0.41286000
H	1.12004600	-2.62461800	-0.47544900
H	1.39291100	-1.95348400	1.14137500
C	-0.47990800	-1.40228000	0.24189200
O	2.78898800	0.74093300	1.20752900
C	-1.02648300	-0.86877200	1.40867300
C	-1.27838900	-1.47467400	-0.90984300
H	-0.42138300	-0.80725100	2.30391500
C	-2.32463900	-0.36007300	1.40727900
C	-2.58348600	-0.98954700	-0.90391100
H	-0.86345100	-1.89945700	-1.81520000
H	-2.72904600	0.07700800	2.31056100
C	-3.10237500	-0.41222900	0.25014700
H	-3.18565200	-1.05279700	-1.80017500
H	-4.10880800	-0.01607700	0.25691500
O	0.94990900	1.55501600	0.29470600
Fe	-0.69825200	1.71257000	-0.54831800

Molecule: Hg-PHE-01

N	0.92746000	3.52397400	0.05774000
H	0.46973000	3.52020800	0.97404200
H	0.27983300	3.93192100	-0.62042100
H	1.75557500	4.12287200	0.11270400
C	1.32102400	2.13485000	-0.33538300
H	1.71893700	2.18398200	-1.34412600
C	2.38082200	1.62165200	0.65494900
C	0.09262200	1.23583000	-0.31949900
H	3.24860800	2.27685600	0.56240400
H	1.98058000	1.72493200	1.66488200
C	2.76860900	0.19067300	0.39848200
O	-0.76367900	1.42417000	0.59105200
C	2.15843100	-0.84177400	1.11033500
C	3.70925000	-0.12695600	-0.58053400
H	1.42567100	-0.60192000	1.87126500
C	2.47896300	-2.16850300	0.84686900
C	4.03230700	-1.45246300	-0.84534500
H	4.18889700	0.66943800	-1.13666500
H	1.99837200	-2.96009800	1.40744400
C	3.41625500	-2.47704900	-0.13300700
H	4.76612700	-1.68598500	-1.60606300
H	3.66848000	-3.50944200	-0.33819300
O	0.01779700	0.31921400	-1.16629300
Hg	-2.00219600	-0.46231500	-0.02663400

Molecule: Hg-PHE-02

N	-3.55851300	0.25408100	-1.07250000
H	-4.17665800	0.56568000	-0.31665900
H	-3.80902200	-0.71048600	-1.30154200
H	-3.71568500	0.83763800	-1.89733200
C	-2.13703300	0.34472100	-0.61730600
H	-1.52676400	-0.18554800	-1.34434900
C	-1.73440600	1.82808900	-0.54539500
C	-2.05693100	-0.32826200	0.75598300
H	-1.96982500	2.27537400	-1.51339700
H	-2.34890200	2.31610100	0.21315000
C	-0.27384600	2.00136000	-0.23621600
O	-3.04309700	-0.27978900	1.49059400
C	0.15691300	2.27463500	1.05983500
C	0.68087000	1.80993300	-1.24081000
H	-0.57603900	2.42008500	1.84366600
C	1.51462200	2.33647900	1.35740500
C	2.04071800	1.85903200	-0.94162600
H	0.35674800	1.61555400	-2.25575800
H	1.83357500	2.54287200	2.37088500
C	2.45891000	2.11761100	0.36179300
H	2.76925500	1.70693500	-1.72730100
H	3.51511000	2.15550000	0.59416300
O	-0.95840900	-0.89329800	1.12914900
Hg	0.78338700	-1.16744000	-0.09797400

Molecule: Mn-PHE-01

N	-1.19444800	3.10659200	0.01737000
H	-1.59664500	2.96388100	0.94796400
H	-1.96502500	3.23763700	-0.64160800
H	-0.63015200	3.95967600	0.02908900
C	-0.35990000	1.92217800	-0.35895900
H	-0.05884000	2.05548500	-1.39375900
C	0.86373700	1.86957400	0.56789300
C	-1.21075600	0.66421800	-0.22642800
H	1.45420000	2.76918700	0.38540600
H	0.51141200	1.89805500	1.60053800
C	1.69918100	0.63721500	0.34918800
O	-2.02834500	0.58678500	0.72136300
C	1.53362600	-0.47821700	1.16929500
C	2.61838500	0.57492700	-0.69750500
H	0.81826400	-0.43746800	1.98166800
C	2.27293900	-1.63493900	0.94995200
C	3.36048900	-0.57924400	-0.91840300
H	2.75126400	1.43588600	-1.34144400
H	2.13690900	-2.49174400	1.59747000
C	3.18893500	-1.68812700	-0.09507500
H	4.07279500	-0.61395500	-1.73289500
H	3.76841200	-2.58633300	-0.26534500
O	-1.01236600	-0.25606300	-1.06735500
Mn	-2.49502300	-1.77231000	-0.11842000

Molecule: Ni-PHE-01

N	-1.44732200	2.93951800	0.05251000
H	-1.82186400	2.78164700	0.99279700
H	-2.23324000	3.00191400	-0.60008900
H	-0.95911000	3.83968700	0.05057900
C	-0.49938000	1.85004200	-0.33385400
H	-0.19099400	2.03180800	-1.35954000
C	0.70961400	1.87339900	0.61833300
C	-1.20703900	0.52203800	-0.26696200
H	1.21116400	2.83012700	0.46598200
H	0.33727300	1.83950900	1.64317500
C	1.65571600	0.72855800	0.37530700
O	-2.03233300	0.25778900	0.65921500
C	1.57406900	-0.42535400	1.15365300
C	2.59533700	0.78675300	-0.65326200
H	0.84505400	-0.47622100	1.95339500
C	2.41631400	-1.50404400	0.90912500
C	3.43923000	-0.28975300	-0.89871800
H	2.66449700	1.67959200	-1.26285300
H	2.34483100	-2.39244000	1.52342300
C	3.35087100	-1.43875600	-0.11852200
H	4.16710200	-0.23198700	-1.69787500
H	4.00949000	-2.27643400	-0.30820000
O	-0.93911100	-0.38529400	-1.10928000
Ni	-2.16713500	-1.60003900	-0.10280100

Molecule: Zn-PHE-03

N	-2.92396800	1.82862000	-0.08206500
H	-3.38720200	1.69590600	0.82023500
H	-3.61231800	1.62414200	-0.80933600
H	-2.63575600	2.80612900	-0.16194700
C	-1.73920700	0.92229100	-0.20372100
H	-1.31428700	1.11009700	-1.18756800
C	-0.72119500	1.28361400	0.89782200
C	-2.25932100	-0.52274500	-0.15785300
H	-0.82398500	2.35223800	1.09989500
H	-0.96702100	0.75368700	1.81778600
C	0.70835000	1.02439200	0.50094200
O	-3.44612100	-0.70936300	0.11697800
C	1.49031900	0.06796100	1.17529700
C	1.28491700	1.72566600	-0.55939000
H	1.08902900	-0.42446600	2.05484500
C	2.81611800	-0.17744400	0.77875200
C	2.59753400	1.48578900	-0.93891300
H	0.70034100	2.47031200	-1.08468000
H	3.40271000	-0.91126800	1.31470800
C	3.36491300	0.52793000	-0.28015200
H	3.02606200	2.04610400	-1.75953000
H	4.38525900	0.34238500	-0.58677900
O	-1.43981200	-1.46773100	-0.44591500
Zn	0.48126200	-1.57578600	-0.18627900

Molecule: Mn-PHE-21

N	3.23924000	-1.01318900	-0.79492300
H	3.72643500	-1.10714600	0.10187800
H	3.76579600	-0.34770000	-1.36434000
H	3.22763900	-1.91624000	-1.27368300
C	1.84957000	-0.51586600	-0.53379100
H	1.43136500	-0.21141300	-1.48907300
C	1.03668400	-1.66983500	0.07421600
C	1.97381100	0.69056700	0.41258500
H	1.17641100	-2.54115800	-0.56965900
H	1.44846700	-1.90750800	1.05634000
C	-0.42792800	-1.35033300	0.17575300
O	2.89373200	0.65620300	1.24925600
C	-0.98579300	-0.91226200	1.37687800
C	-1.24124600	-1.42697500	-0.95816000
H	-0.36346500	-0.85205600	2.26091900
C	-2.32452800	-0.53547500	1.44167600
C	-2.57550700	-1.04103000	-0.89980600
H	-0.81825500	-1.77132300	-1.89404100
H	-2.74265300	-0.19135700	2.37864300
C	-3.11886500	-0.58726400	0.30029100
H	-3.19048100	-1.09192100	-1.78897100
H	-4.15600400	-0.28170700	0.34492000
O	1.13900200	1.63463900	0.28229900
Mn	-0.94236000	1.80303800	-0.51155000

Molecule: Ni-PHE-02

N	3.23289000	-1.09718600	-0.76171200
H	3.58078600	-1.49272100	0.11643300
H	3.90400500	-0.38807400	-1.06571600
H	3.19495700	-1.83697100	-1.46703900
C	1.88314700	-0.49405300	-0.56342900
H	1.60785100	-0.02505100	-1.50600400
C	0.89856900	-1.61422900	-0.19953300
C	2.00189700	0.58102500	0.51980200
H	0.95653400	-2.36641500	-0.99104800
H	1.22292800	-2.08169000	0.73034000
C	-0.53086500	-1.17124700	-0.06588100
O	3.00085800	0.62295600	1.22781400
C	-1.25029600	-1.44121300	1.09721100
C	-1.18755000	-0.49515500	-1.12272200
H	-0.75755800	-1.94632000	1.91685200
C	-2.58409600	-1.06836700	1.21153700
C	-2.54240000	-0.12143400	-0.99798900
H	-0.68371800	-0.37049500	-2.07497900
H	-3.12140100	-1.29021500	2.12382100
C	-3.23271800	-0.39888400	0.17431500
H	-3.03374700	0.36882600	-1.82714400
H	-4.26820900	-0.10657500	0.27637900
O	1.01889300	1.41253200	0.63429000
Ni	-0.64731400	1.43833700	-0.21845000

Molecule: Zn-PHE-011

N	-1.15850600	3.10384500	0.02888300
H	-1.65422100	2.92454900	0.90703300
H	-1.85431200	3.30212500	-0.69446400
H	-0.57381000	3.93494200	0.14965200
C	-0.31518400	1.92555100	-0.33796400
H	0.07565200	2.10065500	-1.33586400
C	0.82618000	1.80108400	0.68903000
C	-1.16875100	0.67365800	-0.32131600
H	1.39574300	2.73108700	0.65089900
H	0.38032600	1.71487800	1.68169500
C	1.72580900	0.62562000	0.41881000
O	-2.07258900	0.55424200	0.55051000
C	1.49670600	-0.60001900	1.04354400
C	2.77559600	0.73300500	-0.49227500
H	0.68069200	-0.69242700	1.75018700
C	2.29748300	-1.70005100	0.75902900
C	3.57983600	-0.36432300	-0.77672600
H	2.96313000	1.68267200	-0.97867400
H	2.10629300	-2.64626900	1.24905800
C	3.34003600	-1.58539800	-0.15434700
H	4.39426500	-0.26652000	-1.48314100
H	3.96485300	-2.44084000	-0.37673600
O	-0.88632900	-0.26487000	-1.11431200
Zn	-2.24813300	-1.51471700	-0.07260400

Molecule: Cd-TRP-01

N	-0.91788300	3.37200000	-0.14678700
H	-1.44815200	3.53886700	-1.00476400
H	-0.26549200	4.14861300	-0.01353600
H	-1.58011100	3.36126000	0.63416300
C	-0.17910700	2.07210300	-0.21031500
H	0.40345600	2.07860300	-1.12638300
C	0.73525500	1.96733300	1.02225000
C	-1.18332500	0.93253200	-0.25603800
H	1.40750500	2.82913800	0.98739400
H	0.11854900	2.06462200	1.91791000
C	1.50757700	0.69409400	1.07503800
O	-2.28331600	1.07573900	0.35170500
C	1.33644800	-0.31615800	1.98422300
C	2.53670700	0.26051100	0.16862800
H	0.65985900	-0.37666700	2.82085000
N	2.19959500	-1.35028600	1.70541600
C	2.94888700	-1.02885900	0.59905500
C	3.14335700	0.82541400	-0.96106300
H	2.28571400	-2.19716800	2.24345900
C	3.94195200	-1.74898800	-0.06169000
C	4.12947400	0.11048600	-1.62046000
H	2.84367400	1.80441500	-1.31396600
H	4.24388500	-2.72981600	0.28261200
C	4.52477500	-1.16363500	-1.17442700
H	4.60606600	0.53440400	-2.49508200
H	5.29820600	-1.69749800	-1.71152600
O	-0.86908300	-0.11750100	-0.86759900
Cd	-2.97870700	-1.05845200	-0.23740800

Molecule: Cd-TRP-02

N	-3.58261500	-0.30055600	1.41406900
H	-3.98047800	0.63586800	1.50898400
H	-3.56428900	-0.74125100	2.33653100
H	-4.19405600	-0.83974400	0.79201500
C	-2.21464100	-0.24815400	0.81146100
H	-1.66119500	0.52711300	1.33300700
C	-1.55590200	-1.62279000	1.00432100
C	-2.37517500	0.11192200	-0.67106000
H	-1.45667200	-1.78220400	2.08176000
H	-2.23907500	-2.38329400	0.62093600
C	-0.23740600	-1.76788600	0.32195300
O	-3.36592500	-0.33506500	-1.26390900
C	0.03049300	-2.61561500	-0.72331600
C	0.97266000	-1.03862200	0.56885300
H	-0.62170300	-3.32190700	-1.21015900
N	1.33080000	-2.46554400	-1.13512900
C	1.93928400	-1.50486300	-0.36791000
C	1.33501500	0.00881500	1.44765400
H	1.77090300	-2.98873200	-1.87521000
C	3.23556500	-1.00513400	-0.41035200
C	2.63804000	0.51862100	1.38481600
H	0.64715300	0.35208800	2.21124500
H	3.95503000	-1.39071500	-1.12108900
C	3.57303800	0.00991400	0.47274500
H	2.93007500	1.30381700	2.06881900
H	4.57424300	0.41855500	0.45665400
O	-1.47431700	0.82884700	-1.22361700
Cd	0.29747700	1.67803300	-0.29772600

Molecule: Cd-TRP-031

N	-1.99760000	3.02294000	0.27033600
H	-2.50753900	3.37613200	-0.54111100
H	-1.19975700	3.63494200	0.45474000
H	-2.63498300	3.06264200	1.06952900
C	-1.53500200	1.61914200	0.04063400
H	-0.89059400	1.65162300	-0.83402000
C	-0.74654400	1.14247200	1.28440500
C	-2.76729700	0.76286000	-0.28821600
H	-0.38815800	2.03279800	1.80859700
H	-1.41491200	0.61924500	1.96752200
C	0.45154800	0.29691900	0.98216400
O	-3.88539600	1.27670100	-0.16021700
C	0.73838100	-0.96565300	1.50719700
C	1.63323600	0.71139900	0.27703100
H	0.17426400	-1.54171700	2.22487800
N	2.02360100	-1.32117500	1.17015300
C	2.58584700	-0.32992600	0.40873000
C	1.98191900	1.87802400	-0.41914800
H	2.45122000	-2.20737800	1.38797300
C	3.85225300	-0.25290400	-0.17158400
C	3.24250400	1.96420900	-0.97693100
H	1.27818400	2.69520400	-0.51302100
H	4.56172100	-1.06380400	-0.07425100
C	4.16355200	0.90428300	-0.86083100
H	3.53286600	2.85804400	-1.51306100
H	5.14036900	1.00280400	-1.31622200
O	-2.56927000	-0.43186500	-0.69636100
Cd	-0.79687000	-1.69117800	-0.37610500

Molecule: Cu-TRP-01

N	-1.59870900	2.99938100	0.06928600
H	-2.14655800	3.24495800	-0.75904300
H	-0.94367700	3.76245400	0.25983200
H	-2.24100800	2.92370500	0.86348800
C	-0.84836400	1.72145600	-0.12923300
H	-0.22358800	1.83955300	-1.01001700
C	0.01137900	1.43657500	1.12361100
C	-1.82881700	0.60045200	-0.33775300
H	0.59444700	2.34064500	1.31437500
H	-0.66011900	1.28758700	1.97095700
C	0.91470200	0.26548400	0.94881300
O	-2.96151900	0.62393400	0.23792600
C	0.67833700	-1.02631600	1.34356900
C	2.18106400	0.26178300	0.26981700
H	-0.16411000	-1.43853300	1.87422200
N	1.71905700	-1.83288300	0.95029300
C	2.66016100	-1.07430300	0.29426600
C	2.95758600	1.25420800	-0.34142400
H	1.79428500	-2.81831900	1.14393700
C	3.88250600	-1.43296900	-0.26968600
C	4.17451000	0.90052500	-0.90027800
H	2.61330800	2.28057100	-0.37110600
H	4.23282100	-2.45674200	-0.24101300
C	4.63133300	-0.42961900	-0.86483700
H	4.78877400	1.65733000	-1.37111300
H	5.58721700	-0.67389100	-1.31005100
O	-1.51283600	-0.42600700	-0.99918500
Cu	-3.26778500	-1.26075400	-0.35285000

Molecule: Cu-TRP-02

N	3.52214700	-0.09143600	1.42255500
H	3.95742400	-1.01410300	1.35636000
H	3.47199900	0.17319500	2.40949200
H	4.12395300	0.57483400	0.92955100
C	2.16120500	-0.08763000	0.80925100
H	1.60421800	-0.90862100	1.25289000
C	1.49059400	1.26368500	1.12512700
C	2.31806500	-0.31157900	-0.69563300
H	1.39127300	1.32605700	2.21195900
H	2.16069800	2.06342400	0.80476100
C	0.16986900	1.42892400	0.45616900
O	3.35380900	0.05207900	-1.24927600
C	-0.13069600	2.30841900	-0.54486300
C	-1.00042600	0.60258000	0.63408400
H	0.48544400	3.07131200	-0.99097900
N	-1.41806600	2.10052600	-0.99295200
C	-1.98122300	1.06730100	-0.30449500
C	-1.34316300	-0.44478900	1.52209400
H	-1.86741200	2.62460400	-1.72757100
C	-3.24915700	0.49767400	-0.38769300
C	-2.60559700	-1.00857600	1.42204800
H	-0.63502000	-0.78649300	2.26504900
H	-3.97531200	0.85751800	-1.10413200
C	-3.54171000	-0.54309700	0.47606300
H	-2.88108500	-1.81752200	2.08473000
H	-4.51496800	-1.01272100	0.42789100
O	1.34174800	-0.86379500	-1.33402900
Cu	-0.32209700	-1.42616700	-0.66667900

Molecule: Cu-TRP-03

N	2.84255900	-2.20541400	0.63456000
H	3.52320000	-2.45261500	-0.08716500
H	2.28183800	-3.02871600	0.86385200
H	3.36936400	-1.92792100	1.46698900
C	1.96897000	-1.09081000	0.16233800
H	1.39705900	-1.48316000	-0.67673500
C	1.03806700	-0.65951300	1.30054600
C	2.87025800	0.03241000	-0.35642600
H	0.71430100	-1.56323400	1.82408200
H	1.59963100	-0.05523800	2.01375200
C	-0.20332200	0.07778200	0.87746300
O	4.09063600	-0.12499900	-0.37109700
C	-0.68928500	1.21881900	1.56155900
C	-1.37667000	-0.51329100	0.23647900
H	-0.14994800	1.88906600	2.21098000
N	-1.99569200	1.37491100	1.31807500
C	-2.46452200	0.34323200	0.50731800
C	-1.58715100	-1.66274600	-0.52730300
H	-2.55134500	2.15436300	1.64302300
C	-3.74258200	0.10918300	0.02586600
C	-2.86607800	-1.91380100	-1.00300800
H	-0.77360800	-2.34149300	-0.74373000
H	-4.55850400	0.78507400	0.24258200
C	-3.92679100	-1.03736500	-0.73668400
H	-3.05003100	-2.80144700	-1.59339600
H	-4.91040500	-1.25992600	-1.12814300
O	2.28457600	1.09899400	-0.76771700
Cu	0.41563200	1.40287600	-0.78906600

Molecule: Fe-TRP-011

N	-1.70324100	2.96138500	0.08566300
H	-2.26825800	3.12404600	-0.75138800
H	-1.08422100	3.76474600	0.22201600
H	-2.34323400	2.90269000	0.88252500
C	-0.91007400	1.70490600	-0.04888500
H	-0.29940800	1.80487700	-0.94325200
C	-0.01182000	1.53800700	1.19225200
C	-1.85369300	0.52994400	-0.21895000
H	0.56462200	2.45874600	1.30313200
H	-0.63734300	1.40953700	2.07574700
C	0.92150300	0.39725900	1.04794900
O	-3.08842700	0.68061800	-0.03172700
C	0.79847200	-0.86777900	1.69873800
C	2.07274200	0.31091100	0.23081900
H	0.04456600	-1.18866900	2.39871400
N	1.78756500	-1.65833200	1.31733400
C	2.61603000	-0.98544000	0.40259800
C	2.69798900	1.21148800	-0.64403100
H	1.92892000	-2.60871000	1.63747100
C	3.74107400	-1.41167000	-0.24813600
C	3.84267300	0.79197500	-1.31373100
H	2.29860400	2.20598800	-0.78850000
H	4.14447900	-2.40452400	-0.10650800
C	4.35441900	-0.48925800	-1.12030900
H	4.34366000	1.46698000	-1.99321300
H	5.24708400	-0.79193100	-1.65046300
O	-1.35890500	-0.59274700	-0.52179500
Fe	-3.32949900	-1.47500700	-0.52111400

Molecule: Fe-TRP-031

N	-3.09356300	1.96797500	-0.59314500
H	-3.61563400	1.71367400	-1.43481900
H	-2.71927700	2.91166900	-0.71470200
H	-3.75404700	1.96445100	0.19027700
C	-1.99912300	0.98718200	-0.33112000
H	-1.41013000	0.90618700	-1.24049800
C	-1.13566600	1.53102600	0.82670200
C	-2.64321800	-0.36012700	0.02249100
H	-0.85218700	2.55362000	0.57296300
H	-1.73973800	1.55260200	1.73482500
C	0.09113100	0.72992900	1.04092800
O	-3.77263300	-0.35897400	0.51206400
C	0.22870000	-0.36081900	1.94696300
C	1.32923200	0.82793700	0.34784700
H	-0.49375200	-0.74446400	2.64856900
N	1.43892500	-0.88784000	1.82496600
C	2.16563700	-0.20373600	0.83909000
C	1.79901200	1.67144600	-0.66655000
H	1.78561100	-1.67401100	2.36071300
C	3.43257100	-0.42219800	0.36551100
C	3.08586000	1.46188500	-1.15572500
H	1.17639400	2.46531900	-1.05606700
H	4.05742700	-1.21643800	0.74943200
C	3.88571100	0.43937400	-0.65173600
H	3.47187900	2.10125700	-1.93720400
H	4.88240200	0.29770200	-1.04656000
O	-1.94379500	-1.41495300	-0.18286000
Fe	-0.18900600	-1.69278800	-1.06114200

Molecule: Hg-TRP-01

N	-0.04762500	3.44748500	0.10622400
H	-0.54234500	3.74578600	-0.73735800
H	0.63980200	4.16622300	0.34796400
H	-0.72943100	3.38272500	0.86705000
C	0.63611000	2.13235700	-0.09724400
H	1.29850800	2.24550100	-0.95002000
C	1.42640200	1.78502500	1.17745300
C	-0.38571200	1.04342400	-0.41238400
H	2.14590700	2.59363300	1.33344300
H	0.73367700	1.78792600	2.02147200
C	2.11652100	0.46767300	1.08765600
O	-1.51151500	1.18045600	0.20354900
C	1.74033700	-0.68953100	1.71769900
C	3.24435400	0.13629000	0.26023600
H	0.93349800	-0.86094000	2.41124100
N	2.56894200	-1.72012600	1.33986100
C	3.50053300	-1.24818900	0.44598500
C	4.05419100	0.86609500	-0.61968100
H	2.51136600	-2.66638800	1.67907000
C	4.53472600	-1.90592600	-0.21683000
C	5.08254200	0.21416300	-1.27975400
H	3.87723500	1.92210600	-0.78186700
H	4.71471700	-2.96220800	-0.06302900
C	5.31953100	-1.15815200	-1.08034200
H	5.71635100	0.76562400	-1.96239000
H	6.12983400	-1.63936300	-1.61278800
O	-0.07488600	0.11538300	-1.15163800
Hg	-2.75005400	-0.55997800	-0.13734300

Molecule: Hg-TRP-02

N	3.68326900	0.57146200	1.30957100
H	4.13483500	-0.34580700	1.32119100
H	3.67969200	0.94423700	2.26177400
H	4.23815100	1.19022500	0.70981100
C	2.29040200	0.47848600	0.76503200
H	1.79063900	-0.30503400	1.32492200
C	1.61646300	1.84027700	1.00051900
C	2.42261000	0.08307500	-0.71750000
H	1.54904700	1.96689400	2.08502800
H	2.28673800	2.61671300	0.62645600
C	0.27635700	2.03852300	0.36767300
O	3.30145900	0.64512500	-1.37949200
C	-0.01550400	3.04014300	-0.53357300
C	-0.93007400	1.29479600	0.52633200
H	0.62885800	3.81490600	-0.91544000
N	-1.31684300	2.95675400	-0.93644800
C	-1.91089500	1.88817100	-0.31525600
C	-1.30273000	0.13485000	1.27049200
H	-1.76831200	3.58375900	-1.58405300
C	-3.20485500	1.40041500	-0.41672900
C	-2.62785900	-0.35519500	1.14705200
H	-0.69092500	-0.19603700	2.10376800
H	-3.92860000	1.89058900	-1.05494700
C	-3.55086000	0.26505400	0.31021000
H	-2.92130500	-1.20769900	1.74370200
H	-4.55345000	-0.13161600	0.23404100
O	1.64770100	-0.82336200	-1.19422200
Hg	-0.23727500	-1.37180400	-0.14129900

Molecule: Hg-TRP-031

N	-2.01134700	3.18854700	0.14120000
H	-2.59378400	3.45776600	-0.65387000
H	-1.20346000	3.81362200	0.18853500
H	-2.57582000	3.31216600	0.98555300
C	-1.56784300	1.76755600	0.01339400
H	-1.01518400	1.70322000	-0.92002300
C	-0.66659100	1.41488200	1.21678300
C	-2.82149000	0.88348000	-0.08051800
H	-0.22621900	2.34392300	1.58834000
H	-1.27696900	1.00681400	2.02205900
C	0.48482800	0.48467900	0.93349600
O	-3.92049800	1.40045300	0.15880500
C	0.90194200	-0.53966200	1.82912000
C	1.68972300	0.84671100	0.19153500
H	0.32997500	-1.00664100	2.61398000
N	2.20312400	-0.80182800	1.65018500
C	2.72368000	0.00484600	0.64634500
C	1.96588200	1.81056200	-0.77847800
H	2.70600400	-1.54513200	2.11450900
C	4.00995900	0.05316600	0.12665100
C	3.25155600	1.87808500	-1.29244400
H	1.19374700	2.48415700	-1.12536000
H	4.78378200	-0.61505800	0.47935400
C	4.25679300	1.00300100	-0.85380700
H	3.48598800	2.61508100	-2.04890700
H	5.24730200	1.07583500	-1.28291100
O	-2.65762300	-0.34237400	-0.39948500
Hg	-0.53679300	-1.26826600	-0.32860000

Molecule:Mn-TRP-02

N	3.30425600	0.56765700	1.63471500
H	3.71182700	-0.29280700	2.00625800
H	3.08890500	1.18862300	2.41776400
H	4.00587900	1.01004600	1.03300600
C	2.08028400	0.27985700	0.82196400
H	1.44583600	-0.36803900	1.41967400
C	1.37740000	1.61484500	0.51973300
C	2.52296100	-0.43187800	-0.46298300
H	1.21077800	2.12033700	1.47514200
H	2.05661700	2.23587000	-0.06742000
C	0.08686900	1.43289600	-0.20113100
O	3.61627600	-0.09346600	-0.95039400
C	-0.15006300	1.63381000	-1.53532100
C	-1.12812300	0.90011600	0.35469400
H	0.50921800	2.01289000	-2.29862500
N	-1.43620300	1.25311400	-1.84616300
C	-2.05786200	0.78286600	-0.71452500
C	-1.48942200	0.44015100	1.62989200
H	-1.85327400	1.31248200	-2.76081600
C	-3.32668200	0.23545900	-0.53322300
C	-2.74870100	-0.10932900	1.80789800
H	-0.79184200	0.50409400	2.45525900
H	-4.02136800	0.14916800	-1.35867100
C	-3.65601700	-0.21017600	0.73707000
H	-3.04106300	-0.47558400	2.78348400
H	-4.62993700	-0.65025600	0.90803800
O	1.73732200	-1.29557200	-0.95781900
Mn	-0.26642400	-1.99166500	-0.23246600

Molecule:Mn-TRP-03

N	-3.05429400	1.91758600	-0.69227500
H	-3.47642600	1.70026900	-1.59671600
H	-2.67556400	2.86606800	-0.72726000
H	-3.79525600	1.86866800	0.01416800
C	-1.99323500	0.91942900	-0.33681800
H	-1.34935400	0.81395100	-1.20452700
C	-1.20620400	1.45812200	0.87266200
C	-2.70752000	-0.40200200	-0.01119000
H	-0.97137800	2.50557500	0.66695100
H	-1.85604000	1.43427300	1.74927600
C	0.05434600	0.70594000	1.12212200
O	-3.83687900	-0.32109000	0.50372100
C	0.26002400	-0.30267900	2.02709300
C	1.27619900	0.84057500	0.37177400
H	-0.41198200	-0.71073200	2.76387900
N	1.53385700	-0.80530100	1.88763400
C	2.17833900	-0.13755900	0.87327900
C	1.67035300	1.64063500	-0.70891700
H	1.92861100	-1.54741800	2.44196500
C	3.45110400	-0.31538800	0.33465000
C	2.93445700	1.46299800	-1.24603600
H	0.99418000	2.37926800	-1.12110800
H	4.12567700	-1.06432300	0.72911500
C	3.81526800	0.49576500	-0.72845800
H	3.25230800	2.07201400	-2.08258600
H	4.79533800	0.37947100	-1.17285300
O	-2.09700900	-1.48548400	-0.26072500
Mn	-0.03378900	-1.76902000	-1.04771100

Molecule:Mn-TRP-015

N	-1.94181000	2.89109800	-0.40299200
H	-2.56841300	2.82895800	-1.20822100
H	-1.43545700	3.77832900	-0.45396000
H	-2.51806100	2.88726400	0.44329500
C	-0.98513700	1.73881400	-0.36903600
H	-0.47613800	1.71998700	-1.32770000
C	0.00357300	1.97073100	0.78022100
C	-1.78898700	0.45571700	-0.19326000
H	0.53347000	2.90270500	0.56171900
H	-0.56855500	2.12497300	1.69766300
C	0.96495200	0.84838600	0.97840300
O	-2.80590500	0.48309600	0.54215300
C	1.04309600	0.04820000	2.08755100
C	1.97747200	0.38564300	0.06730100
H	0.45915500	0.07564300	2.99290600
N	2.04416800	-0.88188200	1.92796800
C	2.63883400	-0.69969700	0.70253600
C	2.38774100	0.77225600	-1.21575000
H	2.32412300	-1.55763600	2.61991700
C	3.68872100	-1.38609200	0.09616500
C	3.42775600	0.08768100	-1.82246300
H	1.89638800	1.59074800	-1.72676800
H	4.18420800	-2.20664500	0.59905300
C	4.07311700	-0.97920600	-1.17149500
H	3.75200800	0.37422300	-2.81475600
H	4.88413600	-1.49350400	-1.67092800
O	-1.38970400	-0.57323600	-0.80546800
Mn	-3.28821300	-1.83292100	-0.31686300

Molecule:Ni-TRP-01

N	-2.01021700	2.90148300	-0.04733600
H	-2.56980900	2.99858300	-0.89862900
H	-1.51869500	3.78461100	0.11537700
H	-2.64544300	2.74039600	0.74020200
C	-1.01958000	1.78799200	-0.16585900
H	-0.42697100	1.96659700	-1.05853500
C	-0.12901100	1.76057100	1.09319700
C	-1.74856600	0.48233300	-0.31638200
H	0.40361600	2.71415100	1.11827000
H	-0.77927700	1.71389400	1.96852200
C	0.81809400	0.61072200	1.09699500
O	-2.83453100	0.25427300	0.30377300
C	0.72415100	-0.51344100	1.87571700
C	1.95430000	0.42219100	0.23745000
H	0.00443900	-0.75936000	2.63904800
N	1.73685000	-1.38687000	1.55979700
C	2.50996600	-0.84383500	0.56034300
C	2.55015000	1.19169400	-0.76977100
H	1.90066900	-2.27187900	2.01124400
C	3.63695100	-1.34495700	-0.08783700
C	3.66948400	0.69482100	-1.41654600
H	2.14084400	2.15780500	-1.03776800
H	4.04890200	-2.31132400	0.17321700
C	4.20706800	-0.56044100	-1.07815100
H	4.14071800	1.27823200	-2.19717200
H	5.08261800	-0.92080800	-1.60271400
O	-1.26482400	-0.45032100	-1.02824300
Ni	-2.78968700	-1.58038200	-0.46205200

Molecule:Ni-TRP-02

N	3.65636200	0.07272600	1.16989800
H	4.09588800	-0.85065300	1.16803000
H	3.72167700	0.46118500	2.11368200
H	4.18185300	0.67211200	0.52615500
C	2.23205600	-0.01115100	0.72816300
H	1.76307400	-0.78950400	1.32292800
C	1.57914800	1.36261200	0.98139700
C	2.22117400	-0.41973700	-0.74552300
H	1.56675700	1.50828900	2.06460800
H	2.22960500	2.12872500	0.55469400
C	0.21005600	1.53026100	0.40698100
O	3.13883700	-0.06503700	-1.47666500
C	-0.16826600	2.52318000	-0.47492000
C	-0.93559600	0.70594600	0.58450800
H	0.41778000	3.33741700	-0.86864500
N	-1.47210100	2.36393000	-0.84272800
C	-1.97868100	1.24530800	-0.23068500
C	-1.17419500	-0.51839600	1.26939800
H	-1.97233100	2.95519500	-1.48875200
C	-3.21551900	0.64089700	-0.34361600
C	-2.44516300	-1.12014900	1.15015500
H	-0.47250200	-0.88085800	2.01132800
H	-3.98851200	1.06916200	-0.96776800
C	-3.43555800	-0.55449500	0.34717800
H	-2.65015300	-2.02718400	1.70175300
H	-4.39414700	-1.04620100	0.26126600
O	1.22442300	-1.13843800	-1.15124000
Ni	-0.42970100	-1.65257600	-0.41801600

Molecule:Ni-TRP-03

N	-2.90183300	2.10216900	0.78674300
H	-3.54893900	2.44998300	0.07652300
H	-2.35601200	2.88508600	1.15345000
H	-3.46307200	1.71101100	1.54809000
C	-1.99849000	1.06186200	0.21108900
H	-1.44214000	1.54504900	-0.58973000
C	-1.05525000	0.56266200	1.31118900
C	-2.86873800	-0.03375600	-0.40694600
H	-0.72867200	1.43395900	1.88482900
H	-1.61127600	-0.08340300	1.99161900
C	0.18791700	-0.15575300	0.84417700
O	-4.09142500	0.06765000	-0.38192300
C	0.71760800	-1.25756300	1.56207400
C	1.35744700	0.47153700	0.21167800
H	0.19189700	-1.94538400	2.20502900
N	2.03197600	-1.35851400	1.35664400
C	2.47491600	-0.33028800	0.52716700
C	1.54011800	1.60923000	-0.57697400
H	2.60966700	-2.11491100	1.69846300
C	3.75147700	-0.06230100	0.05959800
C	2.81785300	1.89759800	-1.03456200
H	0.70863400	2.25375800	-0.82532200
H	4.58691300	-0.70282300	0.30694200
C	3.90607000	1.06933400	-0.72955500
H	2.97823000	2.77809800	-1.64217300
H	4.88686600	1.31898800	-1.11142700
O	-2.24369900	-1.02215300	-0.95062700
Ni	-0.39384600	-1.36051300	-0.81832900

Molecule:Zn-TRP-03

N	-2.84593000	2.26101800	0.40886400
H	-3.46674400	2.48012900	-0.37244600
H	-2.28192000	3.08568100	0.62590800
H	-3.43761800	2.04458000	1.21502900
C	-1.96845500	1.09728500	0.07791400
H	-1.37984100	1.38992000	-0.78882900
C	-1.05581400	0.81419000	1.28969700
C	-2.87529600	-0.07905400	-0.30751000
H	-0.79258500	1.78594400	1.71758900
H	-1.63115300	0.27948400	2.04679100
C	0.22951700	0.08092900	1.02297000
O	-4.09080500	0.02500400	-0.13206200
C	0.69901900	-0.99230200	1.77760800
C	1.38238800	0.57177500	0.28239000
H	0.16877200	-1.59334800	2.49792300
N	2.01935600	-1.19531500	1.53157800
C	2.47002300	-0.27564300	0.60737200
C	1.59037800	1.63357900	-0.60678800
H	2.56076500	-1.96245700	1.90012700
C	3.73148000	-0.12204800	0.03937000
C	2.84821400	1.80007000	-1.16019600
H	0.78222800	2.30659800	-0.86060100
H	4.54272600	-0.79263000	0.28999600
C	3.90474800	0.92647700	-0.84850600
H	3.02408400	2.61505600	-1.85017300
H	4.87249400	1.07931400	-1.30793900
O	-2.31253100	-1.12284200	-0.80439400
Zn	-0.38952400	-1.47090100	-0.80835800

Molecule:Zn-TRP-011

N	-1.70315500	3.22393600	-0.21179300
H	-2.18186400	3.38333800	-1.10040600
H	-1.08256400	4.01618800	-0.02976200
H	-2.41562600	3.18250400	0.52318400
C	-0.93734000	1.93986700	-0.23468000
H	-0.33293100	1.93499200	-1.13659700
C	-0.05106000	1.87256500	1.02381200
C	-1.93687200	0.78947300	-0.24526200
H	0.55581600	2.78169300	1.03442300
H	-0.69963200	1.89682900	1.90187500
C	0.82245200	0.66663700	1.05859000
O	-3.03968600	0.93999800	0.28864400
C	0.65049900	-0.44927200	1.83443500
C	1.97211700	0.41839400	0.23278400
H	-0.10267900	-0.65941900	2.57588700
N	1.62434400	-1.37745000	1.54401700
C	2.45301600	-0.87485000	0.56895900
C	2.63891300	1.15912600	-0.75172200
H	1.73128800	-2.26829100	2.00107800
C	3.57382900	-1.43029800	-0.04513500
C	3.75221000	0.60831100	-1.36433200
H	2.28913000	2.14631000	-1.02730000
H	3.92930300	-2.41644700	0.22495200
C	4.21453900	-0.67331900	-1.01370800
H	4.27797900	1.16987400	-2.12600200
H	5.08760800	-1.07565200	-1.51120600
O	-1.49621300	-0.29167600	-0.77853100
Zn	-2.57102600	-1.87879000	-0.43730200

Molecule:Zn-TRP-021

N	3.17798500	-1.40390200	-1.31181800
H	3.64422000	-0.78021100	-1.97353900
H	2.88172100	-2.24551900	-1.81082700
H	3.85924700	-1.65995500	-0.59024300
C	2.01166000	-0.72927100	-0.66444700
H	1.41296500	-0.28200400	-1.45395500
C	1.20269400	-1.78816400	0.10335800
C	2.56115700	0.35140700	0.27217400
H	0.98813500	-2.59787800	-0.59890000
H	1.83477000	-2.19681000	0.89418700
C	-0.06227300	-1.25284100	0.67927600
O	3.65602800	0.15808800	0.80840200
C	-0.29113900	-0.93077100	1.99114700
C	-1.26436600	-0.92166700	-0.04010700
H	0.36275500	-1.02457200	2.84247300
N	-1.55808300	-0.41507700	2.13461900
C	-2.17882100	-0.38673100	0.90904900
C	-1.64525900	-0.99417500	-1.38800800
H	-1.96449800	-0.10834200	3.00351400
C	-3.45107500	0.04997500	0.54492500
C	-2.90956300	-0.55984600	-1.74935700
H	-0.96321700	-1.38914500	-2.13050900
H	-4.13696800	0.44901200	1.28110400
C	-3.80251800	-0.04432100	-0.79219100
H	-3.21988200	-0.61472400	-2.78483400
H	-4.78340000	0.28752100	-1.10725600
O	1.84149300	1.39297400	0.48135900
Zn	0.12472300	1.85751400	-0.36146100

Molecule:Cd-TYR-01

N	-0.97551800	3.37025600	-0.08000100
H	-1.35190500	3.33872500	0.87105100
H	-1.76578100	3.43128800	-0.72595700
H	-0.40970800	4.21672900	-0.18071600
C	-0.14405400	2.15550000	-0.35337100
H	0.18055000	2.22160500	-1.38827400
C	1.06220700	2.16478900	0.59532400
C	-1.01546300	0.91994500	-0.19106800
H	1.57844300	3.11529100	0.43876100
H	0.69438400	2.15144500	1.62267800
C	2.00570600	1.01601700	0.36953300
O	-2.02587000	0.98257700	0.56067500
C	2.11738600	-0.00807300	1.30574900
C	2.78138800	0.94236500	-0.78859600
H	1.52464800	0.03044200	2.21145800
C	2.97937800	-1.07916700	1.10152000
C	3.64113100	-0.12215000	-1.01094400
H	2.71219000	1.72834100	-1.53109300
H	3.06404800	-1.86678200	1.84050100
C	3.73911200	-1.13649500	-0.06141300
H	4.24006500	-0.17442500	-1.91075800
O	4.60715600	-2.17203800	-0.31805900
H	4.57496200	-2.80619500	0.41184000
O	-0.69267300	-0.11866800	-0.82241800
Cd	-2.63172800	-1.20061700	-0.04711400

Molecule:Cu-TYR-01

N	-2.21322700	2.74291200	-0.04726000
H	-2.59577400	2.56068500	0.88496500
H	-2.97062400	2.64642300	-0.72809800
H	-1.87866200	3.70975100	-0.07269900
C	-1.08611800	1.80737800	-0.35044800
H	-0.75665900	2.00938900	-1.36627900
C	0.05799800	2.05316200	0.65184200
C	-1.58728800	0.38941200	-0.27116200
H	0.35336300	3.09770600	0.53966800
H	-0.33720300	1.91918100	1.66024100
C	1.23388900	1.14359500	0.42713000
O	-2.51674100	0.05928300	0.52026400
C	1.40066300	-0.00701800	1.19469000
C	2.16080600	1.40907800	-0.58118800
H	0.68853500	-0.23235800	1.97914400
C	2.46137200	-0.87436100	0.96815500
C	3.22550200	0.55338000	-0.82039500
H	2.04906300	2.29912500	-1.18842500
H	2.58585800	-1.76599800	1.57085400
C	3.37438000	-0.59245800	-0.04322300
H	3.94445200	0.76496300	-1.60098600
O	4.44352300	-1.41288300	-0.31285400
H	4.43955300	-2.16021300	0.30139000
O	-1.04365400	-0.51584900	-0.97086600
Cu	-2.22557100	-1.87539600	-0.08943800

Molecule:Fe-TYR-02

N	-3.47554200	1.06500600	-0.69444700
H	-3.95226800	1.17980800	0.20512800
H	-4.01203000	0.38937300	-1.24415500
H	-3.47426800	1.95970000	-1.19032500
C	-2.08096900	0.57625200	-0.46944100
H	-1.67204400	0.30974400	-1.44017800
C	-1.26474100	1.72177300	0.16964700
C	-2.17598800	-0.66101200	0.43379400
H	-1.41783100	2.60824800	-0.44966900
H	-1.65514700	1.91735900	1.16860400
C	0.18909600	1.39177000	0.22119100
O	-3.10693100	-0.73693000	1.23000700
C	0.76795500	0.86452300	1.38896200
C	0.97720300	1.49130200	-0.95390400
H	0.17659300	0.79763700	2.29176500
C	2.05343200	0.37695500	1.37208500
C	2.26824100	1.03194400	-0.98338100
H	0.53687200	1.92298900	-1.84280600
H	2.50271800	-0.06017500	2.25428000
C	2.81450100	0.45073900	0.18061600
H	2.87657100	1.09862100	-1.87408600
O	4.06143600	-0.00506700	0.11411800
H	4.34579700	-0.38630100	0.96189900
O	-1.26854400	-1.56639600	0.31165900
Fe	0.43437600	-1.69963000	-0.59165500

Molecule:Cd-TYR-02

N	3.57447600	-0.92590800	-0.89206800
H	4.05486800	-1.28730300	-0.06189100
H	4.07140600	-0.08657200	-1.19754100
H	3.62215400	-1.62094100	-1.63963100
C	2.15572000	-0.61119000	-0.53191000
H	1.73228600	-0.03798100	-1.35292600
C	1.39449000	-1.93666800	-0.35107400
C	2.20131400	0.22315600	0.75883300
H	1.55270900	-2.52823600	-1.25474000
H	1.83428900	-2.47675700	0.48928400
C	-0.07331400	-1.70463500	-0.13445500
O	3.08596100	-0.05078600	1.57903800
C	-0.58678000	-1.50188100	1.14565300
C	-0.93131400	-1.54451600	-1.22564500
H	0.06119100	-1.62129200	2.00507700
C	-1.90703100	-1.11724900	1.33825100
C	-2.25362900	-1.15455000	-1.05083700
H	-0.55445400	-1.70352200	-2.22849100
H	-2.29568500	-0.95657600	2.33654000
C	-2.73630900	-0.92597800	0.23632400
H	-2.91290900	-1.02577100	-1.89893400
O	-4.04502400	-0.52914200	0.36521800
H	-4.25706400	-0.40671500	1.30130000
O	1.33380300	1.14633100	0.93541400
Cd	-0.38556100	1.61130900	-0.30003700

Molecule:Cu-TYR-02

N	-3.31444800	1.71221000	-0.13017400
H	-3.71225900	1.66298300	0.81137900
H	-4.03692500	1.39650800	-0.78067700
H	-3.08102000	2.68651700	-0.33431000
C	-2.10057000	0.84898500	-0.23930000
H	-1.71930700	0.98420900	-1.25039100
C	-1.06054400	1.29094200	0.79907100
C	-2.54259200	-0.60784200	-0.07361400
H	-1.08712700	2.38141300	0.85566900
H	-1.33812400	0.90398700	1.77902400
C	0.35061100	0.88675500	0.46178800
O	-3.73652900	-0.86241100	0.07250900
C	1.10011700	0.06260300	1.33174100
C	1.01737900	1.46114000	-0.64787200
H	0.62471500	-0.33821000	2.21741600
C	2.44328400	-0.20753500	1.08690000
C	2.34671500	1.20881900	-0.88449600
H	0.46929000	2.11751200	-1.31054300
H	3.00444600	-0.84822600	1.75420000
C	3.06877500	0.36761300	-0.01811800
H	2.85643200	1.65116100	-1.72930900
O	4.36864700	0.15534000	-0.30651300
H	4.77493900	-0.43064100	0.35117400
O	-1.62865700	-1.51284600	-0.09944300
Cu	0.23032200	-1.31938700	-0.33384200

Molecule:Fe-TYR-011

N	-1.92955500	2.79304100	0.00458100
H	-2.31003700	2.67776500	0.94777200
H	-2.70787900	2.72067600	-0.65469700
H	-1.52425800	3.72969100	-0.06810300
C	-0.89888700	1.75449900	-0.28897200
H	-0.55178300	1.94049700	-1.30400300
C	0.25206700	1.88372500	0.71777800
C	-1.52175600	0.36665400	-0.25276400
H	0.62205000	2.91267200	0.66275700
H	-0.12789600	1.70802600	1.72395600
C	1.38528900	0.96332100	0.42001500
O	-2.77836400	0.27118500	-0.06541000
C	1.77074400	-0.02408000	1.36326900
C	2.10973200	1.08991500	-0.79598500
H	1.21701900	-0.10561100	2.28790400
C	2.80797800	-0.86742800	1.10539300
C	3.15121100	0.26051800	-1.07305300
H	1.82359200	1.85761600	-1.50180900
H	3.11001200	-1.63655300	1.80399500
C	3.50732100	-0.74147400	-0.12642700
H	3.71980700	0.33236900	-1.98926300
O	4.50046000	-1.53607500	-0.44730300
H	4.67711800	-2.20217100	0.24213000
O	-0.77771100	-0.61631600	-0.40840800
Fe	-2.97608200	-1.79040500	-0.04728000

Molecule:Hg-TYR-01

N	0.18961700	3.56779400	-0.17026400
H	-0.28211300	3.69726400	0.72864100
H	-0.48721200	3.77965500	-0.90707400
H	0.96374200	4.23276600	-0.23572500
C	0.69783600	2.16498900	-0.29640000
H	1.14309700	2.07809700	-1.28375600
C	1.74377400	1.90359100	0.80240700
C	-0.49491100	1.22663000	-0.18141900
H	2.32827300	2.82031100	0.90524800
H	1.22924400	1.73493500	1.74945900
C	2.67359300	0.76564500	0.48394600
O	-1.55118500	1.65945200	0.31642400
C	2.52784500	-0.48861400	1.07043800
C	3.72372600	0.95884500	-0.41564300
H	1.72089900	-0.66001000	1.77182800
C	3.40478800	-1.52636700	0.77437000
C	4.60671700	-0.06398900	-0.72171300
H	3.85837000	1.92980200	-0.87736300
H	3.28702800	-2.49842800	1.23792900
C	4.44659700	-1.31042400	-0.12207300
H	5.42455400	0.09491400	-1.41238700
O	5.35148800	-2.29312400	-0.44802600
H	5.14651500	-3.09876900	0.04603900
O	-0.33878400	0.03202200	-0.58622300
Hg	-2.41664600	-0.70067100	-0.03939800

Molecule:Hg-TYR-02

N	-3.23490100	2.25427200	-0.52964200
H	-3.76421800	2.35067000	0.34147700
H	-3.88009300	1.90268100	-1.24015400
H	-2.89689700	3.17410600	-0.82057300
C	-2.09211600	1.30892200	-0.33292000
H	-1.62602900	1.17487400	-1.30534000
C	-1.09554800	1.94347400	0.66712700
C	-2.69256500	-0.01825700	0.15523900
H	-1.18878700	3.02612600	0.56334800
H	-1.39685700	1.69091400	1.68375300
C	0.34899900	1.58251200	0.44073500
O	-3.78372700	0.01162000	0.72829700
C	1.00508100	0.63538700	1.25960000
C	1.09204700	2.19883000	-0.56483500
H	0.49129400	0.23839300	2.12719800
C	2.37309200	0.34591700	1.08357600
C	2.43550000	1.91335300	-0.74676200
H	0.61391000	2.92701800	-1.20760600
H	2.87115100	-0.35046800	1.74540100
C	3.07934900	0.98671600	0.07320800
H	3.00030200	2.40565600	-1.52743300
O	4.40660600	0.75403800	-0.15550300
H	4.74560600	0.11430100	0.48702500
O	-2.05586000	-1.11383000	-0.07589800
Hg	0.13019700	-1.21287400	-0.16655700

Molecule:Mn-TYR-02

N	3.47951900	-0.95067000	-0.82025700
H	4.00694200	-1.05969600	0.05218900
H	3.96371500	-0.25265900	-1.38802300
H	3.46953200	-1.83706200	-1.32894200
C	2.09287300	-0.49517200	-0.48348300
H	1.64112900	-0.13244000	-1.40285700
C	1.29665800	-1.69155400	0.06973000
C	2.22244900	0.64216600	0.54241200
H	1.44724400	-2.53176000	-0.61146800
H	1.70881000	-1.96753200	1.04193000
C	-0.16751100	-1.37455100	0.17193800
O	3.19555500	0.59894400	1.31658600
C	-0.71064300	-0.83938600	1.33890200
C	-0.99023900	-1.48356200	-0.95229500
H	-0.09157500	-0.74417100	2.22186000
C	-2.02379100	-0.38813500	1.37800100
C	-2.30578600	-1.03923800	-0.93020000
H	-0.58884100	-1.90324900	-1.86654900
H	-2.43416800	0.04131400	2.28388800
C	-2.81538900	-0.47043200	0.23506800
H	-2.93589000	-1.11913600	-1.80590300
O	-4.11155900	-0.01477500	0.21007000
H	-4.33993700	0.35661700	1.07362100
O	1.32101500	1.53212900	0.55345800
Mn	-0.52141600	1.74853000	-0.69557200

Molecule:Mn-TYR-014

N	-2.16681200	2.82011300	-0.03137100
H	-2.64352400	2.50897600	0.81972900
H	-2.85742200	2.85371400	-0.78407600
H	-1.80227000	3.76376400	0.11736500
C	-1.05645200	1.86947100	-0.35904100
H	-0.65995800	2.15369300	-1.32915700
C	0.02123700	1.98263300	0.73169100
C	-1.61941200	0.45536100	-0.41034500
H	0.35520700	3.02154000	0.75487900
H	-0.44414400	1.75680900	1.69339300
C	1.18684900	1.06464500	0.48834500
O	-2.51876000	0.15872600	0.43067900
C	1.22283900	-0.21131300	1.04819900
C	2.23716000	1.45248900	-0.34324100
H	0.41473200	-0.53456300	1.69327000
C	2.27289200	-1.08145900	0.78554500
C	3.29455200	0.59634200	-0.61462200
H	2.22932700	2.44185900	-0.78436700
H	2.29407900	-2.07201100	1.22402800
C	3.30898800	-0.67589700	-0.05032300
H	4.10904100	0.90464600	-1.25702600
O	4.37385200	-1.49497700	-0.34553500
H	4.26592100	-2.34099500	0.11083400
O	-1.12869200	-0.35972900	-1.22369500
Mn	-2.44465700	-2.13376000	-0.02352500

Molecule:Ni-TYR-01

N	-2.21985800	2.70584000	-0.04873800
H	-2.60192600	2.53258800	0.88559700
H	-2.97839000	2.60933100	-0.72869700
H	-1.88034500	3.67098000	-0.08079900
C	-1.09540100	1.76614100	-0.34815200
H	-0.75980500	1.96611400	-1.36230500
C	0.04485700	2.00275300	0.66167600
C	-1.60281300	0.35316800	-0.27256000
H	0.33373900	3.05023000	0.56233600
H	-0.35427800	1.85428500	1.66644800
C	1.22778900	1.10477700	0.42883400
O	-2.52189700	0.02143100	0.54138500
C	1.38333700	-0.07293200	1.15699000
C	2.17927400	1.41577700	-0.54284200
H	0.65283000	-0.33255700	1.91352900
C	2.45764000	-0.92240500	0.92747200
C	3.25844200	0.57894600	-0.78338000
H	2.07652000	2.32799200	-1.11787500
H	2.57364400	-1.83461900	1.50021600
C	3.39668200	-0.59393700	-0.04545300
H	3.99794600	0.82671400	-1.53346600
O	4.48191500	-1.39290500	-0.31360900
H	4.46857200	-2.16262000	0.27218800
O	-1.09127900	-0.55907000	-0.99054200
Ni	-2.30136600	-1.84995300	-0.09390700

Molecule:Ni-TYR-02

N	-3.49854700	1.23048000	-0.63135000
H	-3.85618500	1.51424100	0.28523100
H	-4.18473400	0.59420000	-1.04266800
H	-3.41841500	2.05841500	-1.22608300
C	-2.17287000	0.55829300	-0.48729800
H	-1.89335000	0.21087700	-1.48037300
C	-1.15873400	1.58432800	0.03690100
C	-2.36276800	-0.65544800	0.43069200
H	-1.22734100	2.46843500	-0.60281000
H	-1.44293700	1.88237100	1.04612000
C	0.26983400	1.11000800	0.04041800
O	-3.41499600	-0.79133200	1.04503900
C	1.04509000	1.19238500	1.21210300
C	0.90241800	0.64107100	-1.13446300
H	0.58389500	1.55451100	2.12065400
C	2.37258200	0.81788600	1.22554400
C	2.23747400	0.21037600	-1.11760200
H	0.36300000	0.64333000	-2.07420400
H	2.95450400	0.89082500	2.13524100
C	2.97935700	0.31529300	0.06229200
H	2.70949100	-0.14685000	-2.02160600
O	4.26651900	-0.07564400	0.03037800
H	4.68165000	0.03289300	0.89971000
O	-1.38495700	-1.49618500	0.50211900
Ni	0.31476500	-1.28773100	-0.28041300

Molecule:Zn-TYR-01

N	-1.70275500	2.87133700	0.06159800
H	-2.24931700	2.51213300	0.84882700
H	-2.34335500	3.03556700	-0.71799400
H	-1.28351900	3.76480900	0.32998500
C	-0.63826300	1.88406300	-0.30951100
H	-0.17696800	2.23854700	-1.22656000
C	0.39039300	1.82481500	0.83000000
C	-1.27611000	0.51965200	-0.56174300
H	0.76798300	2.83923600	0.97585300
H	-0.12599800	1.52347700	1.74361600
C	1.52611200	0.88322500	0.53953300
O	-2.29101300	0.26195700	0.18616900
C	1.53736100	-0.40685600	1.06462800
C	2.57769700	1.26859500	-0.29259100
H	0.72820800	-0.72703400	1.70967100
C	2.56711700	-1.29274600	0.77325600
C	3.61298800	0.39635400	-0.59458200
H	2.58897400	2.26853100	-0.70927400
H	2.57024300	-2.29390500	1.18754100
C	3.60537400	-0.88912200	-0.05982900
H	4.42842500	0.70201400	-1.23704800
O	4.65145600	-1.72264400	-0.38066700
H	4.53190700	-2.57399200	0.06284400
O	-0.77825700	-0.23172300	-1.39850900
Zn	-3.11969300	-1.49924500	0.03368200

Molecule:Zn-TYR-02

N	-3.41838100	1.14810600	-0.78441600
H	-3.85538600	1.46873700	0.08475600
H	-4.01766200	0.42178400	-1.18213400
H	-3.36814900	1.92714700	-1.44426100
C	-2.05077800	0.61489200	-0.48864900
H	-1.68233600	0.15277000	-1.40100300
C	-1.14550100	1.78678400	-0.07249100
C	-2.19983800	-0.42981600	0.62202500
H	-1.25820500	2.56779500	-0.82780600
H	-1.50637400	2.18521300	0.87719300
C	0.29825300	1.38404800	0.03463300
O	-3.08378300	-0.26077000	1.46556800
C	0.86224900	1.03268800	1.26018900
C	1.08502800	1.26384500	-1.11329400
H	0.26480100	1.10336700	2.16072900
C	2.17281700	0.58027600	1.34743600
C	2.39302900	0.80479100	-1.04462800
H	0.66701500	1.53029100	-2.07639000
H	2.60291700	0.31193200	2.30488300
C	2.93779700	0.46336700	0.19078800
H	2.99937000	0.72043900	-1.93676800
O	4.23825100	0.01924400	0.21328800
H	4.50163100	-0.16810100	1.12512900
O	-1.39147400	-1.42550300	0.63109500
Zn	0.14529200	-1.73090500	-0.50296900

Molecule:Zn-TYR-011

N	-2.05267200	2.99785400	-0.22660100
H	-2.51037500	2.91030900	0.68554100
H	-2.77702200	2.94294800	-0.94573300
H	-1.60222500	3.91361900	-0.28800000
C	-1.04924500	1.90046300	-0.38982900
H	-0.71658400	1.91434000	-1.42346900
C	0.12489600	2.16397500	0.56628800
C	-1.74491300	0.58081500	-0.06737100
H	0.46622300	3.18421500	0.38030500
H	-0.24816000	2.11869500	1.59118000
C	1.26253200	1.19951900	0.37689900
O	-2.65131100	0.56733500	0.77134100
C	1.39449800	0.07270000	1.18529900
C	2.19916300	1.39957400	-0.63784700
H	0.67762700	-0.10248900	1.97826000
C	2.42866300	-0.83453500	0.99058900
C	3.23743900	0.50399500	-0.84612300
H	2.11769100	2.27251000	-1.27426100
H	2.52615000	-1.70752600	1.62482900
C	3.34942200	-0.61789300	-0.02969400
H	3.96325300	0.66744400	-1.63202900
O	4.39188900	-1.48223500	-0.26989400
H	4.35737100	-2.21112300	0.36491600
O	-1.28351300	-0.43758200	-0.69563200
Zn	-2.09188300	-2.14265800	-0.16037100

Molecule: Cd-LEU-01

N	-1.59517600	2.68616600	0.14959800
H	-1.37952000	2.87716300	-0.83221900
H	-0.94717100	3.23133800	0.72225300
H	-2.54812100	3.00153800	0.34384300
C	-1.46682000	1.21960600	0.43050800
H	-1.64707300	1.09218000	1.49415900
C	-2.51137800	0.47909200	-0.40740800
C	-0.03777700	0.79810600	0.10882000
H	-3.48081000	0.93514200	-0.18448100
H	-2.29794500	0.66909600	-1.46278700
C	-2.61358700	-1.03056500	-0.16864700
O	0.64024100	1.46970700	-0.68838900
H	-1.65093600	-1.48492400	-0.41694000
C	-2.94525200	-1.36625700	1.28425400
C	-3.66648400	-1.61116700	-1.11161300
H	-3.87311400	-0.87550500	1.59133200
H	-3.08038100	-2.44286800	1.40537400
H	-2.15538900	-1.05493500	1.96914100
H	-3.43352500	-1.38536100	-2.15458900
H	-3.72532100	-2.69588500	-1.00499200
H	-4.65447900	-1.19754500	-0.89129500
O	0.37268500	-0.25970000	0.68762400
Cd	2.44550900	-0.41844900	-0.05073600

Molecule: Cd-LEU-02

N	-2.32653400	-1.69149300	-1.24470200
H	-2.94070500	-2.08905200	-0.52797200
H	-1.86992900	-2.47002700	-1.72460500
H	-2.89001200	-1.17581400	-1.92378100
C	-1.32160300	-0.79374300	-0.59076000
H	-0.59151100	-0.53074700	-1.35165200
C	-2.04464200	0.44420400	-0.05199800
C	-0.66039000	-1.59913700	0.53326500
H	-2.75767700	0.77295100	-0.81399800
H	-2.62657700	0.14254900	0.82243400
C	-1.14638500	1.63500200	0.30301100
O	-1.32524400	-2.48161400	1.08716400
H	-0.33517900	1.28668900	0.94904300
C	-0.53480000	2.27851500	-0.94160200
C	-1.96219000	2.65673000	1.09292200
H	-1.31998300	2.64280500	-1.60992400
H	0.09396900	3.12694000	-0.66589500
H	0.08651100	1.58100000	-1.51145300
H	-2.34885500	2.22145700	2.01675500
H	-1.35345200	3.52386200	1.35580600
H	-2.81410900	3.00967000	0.50497900
O	0.53676800	-1.29914000	0.87705100
Cd	1.88085700	0.04822400	-0.09561600

Molecule: Cd-LEU-031

N	0.10350600	1.59013300	1.82440300
H	-0.75385700	1.03169800	1.83962300
H	-0.16133400	2.57480000	1.74676300
H	0.57848000	1.45893200	2.72076400
C	1.01223600	1.17946000	0.70290000
H	1.88418200	1.82108000	0.78540700
C	1.39632100	-0.28502800	0.89427900
C	0.35156700	1.47994000	-0.65090200
H	1.94698800	-0.36250100	1.83652500
H	0.48476300	-0.87964100	1.01575900
C	2.23851500	-0.87916600	-0.23792600
O	-0.87619400	1.14520700	-0.83839700
H	1.67966700	-0.76664100	-1.17295900
C	3.58238800	-0.16792600	-0.39127500
C	2.44128100	-2.37199200	0.01187700
H	4.15751400	-0.23130100	0.53663100
H	4.17103600	-0.63540900	-1.18302200
H	3.46426100	0.88573800	-0.64547400
H	1.48579400	-2.89481400	0.09211100
H	3.01023800	-2.82741800	-0.80075000
H	2.99394800	-2.53552400	0.94103800
O	1.03410700	2.04977100	-1.50434200
Cd	-1.93882000	-0.56378100	-0.07739700

Molecule: Cu-LEU-01

N	-0.60808400	2.65147000	0.20950700
H	-0.37738700	2.82129000	-0.77359500
H	0.13364600	3.05716700	0.78528000
H	-1.48445600	3.13341400	0.42349400
C	-0.75706700	1.18217300	0.46638300
H	-0.93463600	1.06496800	1.53160100
C	-1.93160800	0.65756800	-0.36162900
C	0.55193300	0.51761500	0.10843800
H	-2.80891300	1.24171100	-0.06944400
H	-1.72749700	0.87309500	-1.41377900
C	-2.24280900	-0.83226400	-0.19441200
O	1.27516600	0.94786400	-0.81638600
H	-1.37397700	-1.40543700	-0.53268100
C	-2.53174300	-1.20824700	1.25795200
C	-3.42604500	-1.19015800	-1.09275500
H	-3.35193300	-0.60511600	1.65734700
H	-2.82414900	-2.25773500	1.32641500
H	-1.66283900	-1.06663200	1.90199000
H	-3.22566700	-0.93434000	-2.13529100
H	-3.64169200	-2.25884300	-1.04147300
H	-4.32401900	-0.65084600	-0.77884200
O	0.92832500	-0.50686900	0.77667500
Cu	2.62952500	-0.68486400	-0.10806000

Molecule: Cu-LEU-021

N	-0.66163500	-2.55419700	1.12296100
H	-0.72093400	-3.22979600	0.35550800
H	-1.57496800	-2.52745500	1.58206100
H	0.03564300	-2.87827500	1.79607300
C	-0.28492100	-1.21102900	0.58289200
H	-0.35665200	-0.50545200	1.40714100
C	1.13965700	-1.28432300	0.02835600
C	-1.30045900	-0.86085000	-0.50222900
H	1.75582100	-1.79673500	0.77327300
H	1.12183000	-1.91365500	-0.86493900
C	1.79328700	0.06264300	-0.29620500
O	-1.85229400	-1.76044600	-1.12998500
H	1.11126800	0.64310300	-0.92746000
C	2.09905800	0.87232700	0.96401100
C	3.07329400	-0.18349600	-1.09338100
H	2.75308800	0.30770800	1.63396100
H	2.60379100	1.80479700	0.70557000
H	1.19732300	1.13528800	1.52515300
H	2.86516400	-0.71938600	-2.02168100
H	3.56083300	0.75966000	-1.34660800
H	3.77996700	-0.78122700	-0.51059200
O	-1.52372900	0.38942500	-0.74623600
Cu	-0.88317400	1.86818400	0.17037600

Molecule: Cu-LEU-031

N	-0.62520800	1.29553300	1.84127300
H	-1.35245100	0.57580700	1.83303300
H	-1.07903700	2.21080200	1.78803500
H	-0.13506800	1.23393200	2.73757300
C	0.35046000	1.10199200	0.71619400
H	1.06461000	1.91350900	0.81034600
C	1.03872500	-0.24852500	0.87511700
C	-0.34723400	1.28471100	-0.63675700
H	1.57279100	-0.23684700	1.82988100
H	0.28310800	-1.03575000	0.94852200
C	2.01614000	-0.59806800	-0.25212000
O	-1.35861800	0.53281600	-0.93983100
H	1.46454700	-0.57834200	-1.19805800
C	3.17077800	0.39790400	-0.34835400
C	2.53687700	-2.01870200	-0.04667400
H	3.72478000	0.43805700	0.59362300
H	3.86651800	0.09711000	-1.13395500
H	2.82693500	1.40651100	-0.58174300
H	1.71809200	-2.74053100	-0.01331300
H	3.20820700	-2.30523200	-0.85824500
H	3.09342300	-2.09509100	0.89145200
O	0.07743600	2.13508000	-1.41081900
Cu	-2.00775900	-0.99350700	-0.12328600

Molecule: Fe-LEU-01

N	-0.43908300	2.64861600	0.09697400
H	-0.16909300	2.74829200	-0.88644600
H	0.29423600	3.06433300	0.67770800
H	-1.30400000	3.17591800	0.24413100
C	-0.66890400	1.21081900	0.44569000
H	-0.90595000	1.17126400	1.50542400
C	-1.81713800	0.66502100	-0.41147600
C	0.60685100	0.45551500	0.21450700
H	-2.68800800	1.28773900	-0.18961000
H	-1.55670400	0.81935400	-1.46115200
C	-2.17165300	-0.80627000	-0.17958900
O	1.40909400	0.75936700	-0.72308500
H	-1.29990000	-1.41622300	-0.43670000
C	-2.55357600	-1.09475100	1.27097600
C	-3.30801700	-1.18890300	-1.12611100
H	-3.38566300	-0.45796300	1.58390000
H	-2.86857600	-2.13408000	1.37979700
H	-1.72323000	-0.92977700	1.95910600
H	-3.04113500	-0.99679400	-2.16733400
H	-3.55126200	-2.24810700	-1.02737200
H	-4.20936400	-0.61359500	-0.89786600
O	0.94654400	-0.52909300	0.93783800
Fe	2.69660600	-0.74236400	-0.15247700

Molecule: Fe-LEU-027

N	0.69670700	2.53090100	1.09864500
H	0.70954800	3.22259000	0.34332000
H	1.62802100	2.51228400	1.52223500
H	0.02356300	2.83460000	1.80603200
C	0.31197500	1.18745400	0.56837900
H	0.36982800	0.48608100	1.39802900
C	-1.10305000	1.26295300	-0.01234600
C	1.33839900	0.80996000	-0.48640800
H	-1.71418400	1.81310800	0.70871400
H	-1.06166600	1.86211600	-0.92485000
C	-1.78398500	-0.07990000	-0.29595600
O	1.94652800	1.63575300	-1.12949800
H	-1.12213700	-0.69592200	-0.91672700
C	-2.10648500	-0.84532000	0.98668000
C	-3.06255700	0.17960900	-1.09256800
H	-2.75309600	-0.24972400	1.63656400
H	-2.62442000	-1.77740000	0.75560700
H	-1.21197700	-1.10052400	1.56073400
H	-2.84781500	0.68877700	-2.03409500
H	-3.57657100	-0.75598600	-1.31882300
H	-3.74781700	0.80890700	-0.51749200
O	1.51712000	-0.48698100	-0.68577400
Fe	0.91449200	-1.98630400	0.18483600

Molecule: Fe-LEU-031

N	0.65181500	-1.28465500	1.84738300
H	1.37939700	-0.56331400	1.85108500
H	1.10677900	-2.20066800	1.80497400
H	0.14417000	-1.22171700	2.73448600
C	-0.30889300	-1.09565600	0.71108800
H	-1.01659000	-1.91590500	0.77974200
C	-1.00883700	0.25148200	0.86470300
C	0.39239800	-1.24323400	-0.63729900
H	-1.53737800	0.22917300	1.82218800
H	-0.25944800	1.04439600	0.93621200
C	-1.99482800	0.59743500	-0.25589600
O	1.45903000	-0.47562000	-0.85816300
H	-1.43996600	0.63099500	-1.19969700
C	-3.11202500	-0.43543700	-0.39220100
C	-2.56779500	1.98971200	0.00012700
H	-3.66113800	-0.53665400	0.54770600
H	-3.81965500	-0.12444600	-1.16286700
H	-2.73276000	-1.41928300	-0.67159700
H	-1.77692100	2.73934000	0.07046700
H	-3.24264300	2.28329200	-0.80565300
H	-3.13371200	2.00780700	0.93550000
O	-0.01317800	-1.99662000	-1.48823400
Fe	2.13308600	1.05506500	-0.13639200

Molecule: Hg-LEU-02

N	2.67800600	-1.70178900	1.26383300
H	3.32382800	-2.07669100	0.56295300
H	2.21423000	-2.49446000	1.71333900
H	3.21234000	-1.19429600	1.97242500
C	1.68702000	-0.79571800	0.60161800
H	0.94368100	-0.53826500	1.35109000
C	2.42257700	0.44606200	0.08681200
C	1.05127700	-1.58757700	-0.54220100
H	3.10297100	0.77834600	0.87662900
H	3.03956200	0.14427600	-0.76309700
C	1.53032400	1.62913100	-0.30618400
O	1.71243400	-2.46024300	-1.10091700
H	0.73793800	1.27054100	-0.96945400
C	0.87949000	2.27881800	0.91467500
C	2.36302100	2.64819500	-1.08105700
H	1.64119600	2.65720800	1.60197900
H	0.24918000	3.11733500	0.61371600
H	0.24902500	1.57797500	1.47065900
H	2.78134700	2.20738100	-1.98832200
H	1.75574300	3.50778200	-1.37085700
H	3.19346600	3.01436600	-0.47086200
O	-0.14987800	-1.29893000	-0.93348900
Hg	-1.46616600	0.02876200	0.05982800

Molecule: Hg-LEU-011

N	-1.79814500	2.61846900	-0.14927600
H	-1.38107000	2.59395100	-1.08311300
H	-1.16886200	3.14183000	0.46332200
H	-2.68844600	3.11920400	-0.20559800
C	-2.02568000	1.22808800	0.36396200
H	-2.44234100	1.33441600	1.36136900
C	-3.00143800	0.52011400	-0.57704700
C	-0.69076000	0.49422900	0.48377600
H	-3.90014600	1.14259600	-0.63043300
H	-2.55837200	0.50158400	-1.57628300
C	-3.40384000	-0.90078800	-0.17024100
O	0.21572600	0.89891400	-0.34099000
H	-2.50216300	-1.51924200	-0.14750300
C	-4.05832000	-0.94939700	1.20945300
C	-4.33999500	-1.47552400	-1.23214700
H	-4.94489900	-0.30945400	1.23877000
H	-4.37390700	-1.96783900	1.44415000
H	-3.38070100	-0.62435400	2.00005700
H	-3.87461500	-1.46612600	-2.22006300
H	-4.60722800	-2.50664500	-0.99337300
H	-5.26400400	-0.89357200	-1.28967900
O	-0.56986600	-0.41012000	1.30417100
Hg	2.04533800	-0.22857900	-0.06860800

Molecule: Hg-LEU-021

N	2.67850800	-1.70155300	1.26363800
H	3.32415100	-2.07637100	0.56255500
H	2.21504200	-2.49427100	1.71338800
H	3.21297900	-1.19388900	1.97201000
C	1.68712400	-0.79570400	0.60172400
H	0.94384400	-0.53861000	1.35136800
C	2.42238300	0.44629900	0.08698500
C	1.05137400	-1.58759800	-0.54210600
H	3.10261300	0.77871900	0.87689100
H	3.03953900	0.14459800	-0.76283400
C	1.53008200	1.62924600	-0.30622100
O	1.71273500	-2.45996700	-1.10105100
H	0.73774100	1.27054800	-0.96949400
C	0.87917200	2.27909600	0.91449800
C	2.36280200	2.64826300	-1.08114300
H	1.64081500	2.65765600	1.60177800
H	0.24879900	3.11750700	0.61336900
H	0.24876500	1.57832200	1.47060600
H	2.78109200	2.20741100	-1.98840800
H	1.75556900	3.50788400	-1.37093800
H	3.19329000	3.01438800	-0.47097100
O	-0.14999800	-1.29940300	-0.93305400
Hg	-1.46616600	0.02867900	0.05982000

Molecule:Mn-LEU-11

N	-0.82314900	2.69667900	-0.05591400
H	-0.39811700	2.76567700	-0.98491500
H	-0.27762800	3.28147700	0.58040000
H	-1.77659600	3.06224000	-0.09567000
C	-0.81790700	1.26386100	0.38593000
H	-1.10396000	1.25397300	1.43303900
C	-1.80666100	0.48676500	-0.48736200
C	0.59834400	0.72646700	0.20770000
H	-2.76646800	1.01121000	-0.45595900
H	-1.44705500	0.52877400	-1.51889800
C	-2.02966700	-0.97241100	-0.07817700
O	1.28500500	1.19276500	-0.72837700
H	-1.05563200	-1.46410800	-0.00935100
C	-2.72308000	-1.07938900	1.27879400
C	-2.83903300	-1.68279600	-1.16173400
H	-3.68556400	-0.56010000	1.25775100
H	-2.91190100	-2.12397600	1.53354500
H	-2.12484200	-0.64588400	2.08186400
H	-2.31866300	-1.65788000	-2.12161900
H	-3.00957900	-2.72801600	-0.89643200
H	-3.81489800	-1.20756300	-1.29536300
O	0.97157200	-0.20621400	0.97902400
Mn	2.88433400	-0.82959800	-0.07972300

Molecule:Mn-LEU-022

N	2.20965500	1.19816700	1.27335500
H	2.80540500	1.63222000	0.56194000
H	2.77306700	0.50032400	1.76280800
H	1.90902700	1.90713000	1.94452500
C	1.03345800	0.56166900	0.59930400
H	0.55470000	-0.07479900	1.33957800
C	0.08710700	1.65648400	0.10339500
C	1.56786500	-0.28394700	-0.56608000
H	-0.02100800	2.40068700	0.89848700
H	0.56093900	2.15858000	-0.74371500
C	-1.30998500	1.16343200	-0.29213900
O	2.61773300	0.09153700	-1.11399800
H	-1.20069600	0.27475800	-0.92164200
C	-2.13829800	0.77996300	0.93356500
C	-2.02288600	2.24005100	-1.10642700
H	-2.31011000	1.65289900	1.56881600
H	-3.11015500	0.38287900	0.63495000
H	-1.64415700	0.02094100	1.54571600
H	-1.46781500	2.48290500	-2.01484200
H	-3.02298700	1.91221400	-1.39649200
H	-2.12725200	3.15839600	-0.52156600
O	0.87725300	-1.29016300	-0.92184200
Mn	-0.81720000	-2.15652800	0.18739900

Molecule:Mn-LEU-031

N	0.66579700	-1.27593900	1.80015200
H	1.39141600	-0.55874400	1.73709100
H	1.12078800	-2.19037100	1.75804500
H	0.20748800	-1.18605700	2.70966300
C	-0.33555500	-1.11405200	0.69418100
H	-1.06584700	-1.90530300	0.83332900
C	-0.97732300	0.26450800	0.83392200
C	0.35621100	-1.32536700	-0.66139700
H	-1.46127000	0.31081800	1.81431100
H	-0.18328500	1.01663700	0.84122000
C	-1.99782700	0.62224100	-0.24902500
O	1.52862200	-0.84293700	-0.80798900
H	-1.52302600	0.48943900	-1.22592400
C	-3.23294300	-0.27388200	-0.18856500
C	-2.38372200	2.09357300	-0.11170200
H	-3.72604400	-0.18088100	0.78312600
H	-3.95201500	0.01401300	-0.95798700
H	-2.98514700	-1.32479300	-0.34367600
H	-1.50861800	2.74078300	-0.20455100
H	-3.10124700	2.38057800	-0.88264600
H	-2.84248900	2.28604600	0.86210900
O	-0.26985100	-1.94803700	-1.52901600
Mn	2.25302000	1.11060300	-0.14134500

Molecule:Ni-LEU-01

N	-0.47363900	2.66024900	0.07606600
H	-0.17646600	2.74333500	-0.90044200
H	0.24164000	3.09190400	0.66663300
H	-1.34657800	3.18117000	0.19175500
C	-0.69227700	1.22446600	0.44010600
H	-0.94306100	1.19874300	1.49665500
C	-1.82780100	0.66408100	-0.42353700
C	0.59566400	0.47581600	0.22511800
H	-2.70344000	1.29105900	-0.23333000
H	-1.54916300	0.79472000	-1.47210300
C	-2.19182600	-0.80107000	-0.16681500
O	1.41422800	0.82830100	-0.67986100
H	-1.31175900	-1.41650400	-0.37519100
C	-2.62649800	-1.04899200	1.27651200
C	-3.29229100	-1.21448700	-1.14242400
H	-3.47468300	-0.41012100	1.53790100
H	-2.93664900	-2.08740800	1.40681600
H	-1.82428200	-0.85359900	1.98954200
H	-2.98184800	-1.06438300	-2.17850500
H	-3.54402100	-2.26861800	-1.01328900
H	-4.19986300	-0.62852100	-0.97388800
O	0.88003000	-0.54378900	0.92269300
Ni	2.56863400	-0.72387500	-0.13826500

Molecule:Ni-LEU-021

N	-0.76610600	2.52270600	1.13108100
H	-1.10118400	3.12164800	0.37129000
H	0.04692100	2.97800400	1.55282900
H	-1.50115900	2.44714700	1.83782400
C	-0.41561500	1.16821700	0.59877000
H	0.02464000	0.61218400	1.42254400
C	-1.69288900	0.49746700	0.08539600
C	0.60342400	1.37440500	-0.51810600
H	-2.44964400	0.59022500	0.87016400
H	-2.04622800	1.06767100	-0.77727800
C	-1.55470700	-0.98143600	-0.28865800
O	0.57243900	2.38954400	-1.19512500
H	-0.69723500	-1.09480600	-0.95956300
C	-1.33657300	-1.86500800	0.93897200
C	-2.80299900	-1.42535600	-1.04845300
H	-2.19712100	-1.80706400	1.61125300
H	-1.20922500	-2.90780800	0.64410200
H	-0.45427000	-1.57660000	1.51927400
H	-2.93400100	-0.84587200	-1.96436600
H	-2.73742100	-2.48064600	-1.31965100
H	-3.69694100	-1.29211800	-0.43290600
O	1.47599400	0.42659800	-0.73758900
Ni	1.89729600	-1.12892100	0.16289600

Molecule:Ni-LEU-031

N	-0.49512700	1.49701100	1.77841300
H	-1.19456100	0.75917700	1.89933400
H	-0.98507500	2.38459700	1.64240300
H	0.04869800	1.56094400	2.64321900
C	0.41805800	1.18966100	0.62966800
H	1.13148500	2.00662700	0.58049800
C	1.11377900	-0.14371800	0.90013500
C	-0.36592200	1.18653000	-0.67803700
H	1.69830800	-0.02542200	1.81700900
H	0.34840200	-0.89950400	1.10429900
C	2.02598400	-0.63910600	-0.22516800
O	-1.54609000	0.59897000	-0.67398300
H	1.41901900	-0.76443700	-1.12785100
C	3.15328800	0.34515800	-0.53355200
C	2.58880600	-2.00630500	0.15654500
H	3.75200200	0.53663500	0.36114900
H	3.81604800	-0.06391400	-1.29848600
H	2.77772600	1.30104500	-0.90053700
H	1.78954800	-2.71920100	0.37042600
H	3.19978100	-2.41303500	-0.65120700
H	3.21816800	-1.92831100	1.04728400
O	0.08394800	1.71785100	-1.67265000
Ni	-2.12358900	-1.01222000	-0.09503200

Molecule:Zn-LEU-02

N	-1.05175100	2.36180300	1.23388500
H	-1.51180200	2.92479100	0.51259900
H	-0.28119800	2.91381300	1.61637400
H	-1.71777600	2.16880100	1.98501900
C	-0.55327600	1.08675500	0.62675700
H	0.06610100	0.60582000	1.37867300
C	-1.76255700	0.23011600	0.24728800
C	0.29449000	1.45930300	-0.59403100
H	-2.38930800	0.12518000	1.13850700
H	-2.34252700	0.78614500	-0.49419400
C	-1.43473900	-1.16362800	-0.29868600
O	-0.03803300	2.44362800	-1.25924700
H	-0.67995100	-1.05857400	-1.08253700
C	-0.88891600	-2.09207400	0.78554800
C	-2.68462600	-1.76984900	-0.93482100
H	-1.66649400	-2.32211000	1.51885100
H	-0.54353900	-3.03263700	0.35350600
H	-0.05589200	-1.64826600	1.33797300
H	-3.05774900	-1.14526200	-1.74890200
H	-2.47181800	-2.76210600	-1.33765000
H	-3.48349300	-1.87398300	-0.19520600
O	1.29721400	0.71019100	-0.88633200
Zn	1.98673300	-0.79828400	0.15173600

Molecule:Zn-LEU-03

N	0.56098900	-1.33912900	1.80308700
H	1.29663500	-0.62807700	1.79499800
H	1.00529500	-2.25838200	1.74613800
H	0.07350900	-1.27533000	2.70032800
C	-0.40967100	-1.12917500	0.67690100
H	-1.14437700	-1.92255500	0.77232000
C	-1.06171800	0.24026900	0.84041100
C	0.29959300	-1.32058100	-0.67029800
H	-1.56449200	0.25541800	1.81217000
H	-0.28365000	1.00736800	0.88382700
C	-2.06982600	0.60270300	-0.25444300
O	1.41798500	-0.71203100	-0.87252100
H	-1.58970200	0.45880800	-1.22777600
C	-3.31376100	-0.28145300	-0.19675500
C	-2.44298400	2.07847300	-0.13187900
H	-3.81380500	-0.17469200	0.76989200
H	-4.02331100	0.00587300	-0.97502500
H	-3.07531600	-1.33635700	-0.34003800
H	-1.56257700	2.71708000	-0.23230400
H	-3.15852900	2.36299700	-0.90552700
H	-2.89951800	2.28484300	0.84004700
O	-0.22996600	-2.05612200	-1.50242300
Zn	2.04329900	0.96269000	-0.09482400

Molecule:Zn-LEU-012

N	-0.76224900	2.69243400	0.11127100
H	-0.47207200	2.80479900	-0.86430800
H	-0.08044800	3.17145900	0.70421000
H	-1.67117200	3.14518100	0.23258000
C	-0.85509600	1.23638300	0.45517200
H	-1.05296600	1.17726500	1.52063500
C	-1.98288800	0.61444600	-0.36908200
C	0.48742500	0.59504400	0.14546700
H	-2.90467000	1.14437200	-0.11157500
H	-1.77506500	0.80942700	-1.42445500
C	-2.18687600	-0.88913600	-0.16053000
O	1.10533400	0.91664400	-0.89297000
H	-1.25260500	-1.40046400	-0.41314800
C	-2.54602000	-1.23109000	1.28432800
C	-3.26570500	-1.38527800	-1.12114500
H	-3.45281100	-0.70209100	1.59062400
H	-2.73258600	-2.30188000	1.38725700
H	-1.74874500	-0.96760400	1.98055700
H	-3.00427500	-1.16652700	-2.15870100
H	-3.40301500	-2.46400800	-1.02647700
H	-4.22462600	-0.90495700	-0.90799500
O	0.93078500	-0.28185300	0.95253000
Zn	2.63056000	-0.66375200	-0.10566100

Molecule:Cd-MET-01

N	-0.35963900	3.12069500	-0.00963400
H	-0.11810400	3.08098900	-1.00358200
H	0.43720000	3.52313900	0.48896200
H	-1.16401400	3.74138000	0.10865200
C	-0.68284600	1.75161000	0.50406500
H	-0.88524500	1.85217600	1.56747200
C	-1.91834300	1.23080600	-0.23504900
C	0.52854200	0.85333800	0.30752100
H	-2.69962600	1.98690300	-0.12619600
H	-1.68516200	1.14501600	-1.29869000
C	-2.41172800	-0.10062000	0.30988200
O	1.34099700	1.12602100	-0.61844900
H	-1.68884600	-0.89327500	0.12165000
H	-2.57936100	-0.03564500	1.38590300
S	-3.97953200	-0.54990900	-0.50350500
C	-4.24917900	-2.16058700	0.28116400
H	-4.32626500	-2.05518700	1.36328100
H	-5.18785800	-2.55744000	-0.10360600
H	-3.44298500	-2.85148500	0.03489900
O	0.65377900	-0.14416200	1.06275700
Cd	2.62444500	-0.77677200	-0.10365000

Molecule:Cd-MET-02

N	-3.20958000	-2.03574600	-0.16852200
H	-3.59156000	-1.82301000	-1.09549400
H	-3.98350500	-2.36454600	0.41172400
H	-2.51697400	-2.78312400	-0.24297500
C	-2.61968900	-0.78323200	0.39920100
H	-2.44254100	-0.96050400	1.45662700
C	-1.30695200	-0.50073200	-0.33335800
C	-3.64394400	0.35747000	0.22056700
H	-0.64389500	-1.35809500	-0.19122900
H	-1.51240000	-0.40853500	-1.40246500
C	-0.63866700	0.76161700	0.18379300
O	-4.42593000	0.26051400	-0.75639900
H	-1.25931700	1.64106100	0.01965700
H	-0.39232800	0.69322500	1.24107100
S	0.89102100	1.10034600	-0.75024000
C	1.47909300	2.58784100	0.10504800
H	1.52460000	2.41422900	1.17667500
H	2.46889000	2.81278500	-0.28650000
H	0.79291900	3.40005700	-0.12638700
O	-3.57606500	1.28832400	1.05525500
Cd	2.58674500	-0.65723700	0.13292600

Molecule:Cd-MET-031

N	-0.70522700	3.08110300	-0.97175300
H	-0.47951600	3.71353700	-0.19763000
H	-1.69776700	3.19507000	-1.18819200
H	-0.15934300	3.34600400	-1.79460800
C	-0.40449200	1.67614600	-0.55651700
H	-0.84024800	1.02022300	-1.30755500
C	1.11395200	1.50414200	-0.50157600
C	-1.06536300	1.44022800	0.80705500
H	1.50082500	1.71066700	-1.50222100
H	1.52393600	2.25774500	0.17482300
C	1.55241200	0.11878200	-0.05152000
O	-1.16564200	2.40079700	1.57808700
H	1.27308800	-0.06233500	0.98546500
H	1.10004700	-0.65864700	-0.67526600
S	3.36399000	-0.01182500	-0.18375000
C	3.59942300	-1.60561300	0.64285500
H	3.06077400	-2.39718000	0.12178500
H	4.66579000	-1.82718500	0.61827800
H	3.27032800	-1.55584700	1.68061200
O	-1.44563800	0.25201800	1.09450200
Cd	-1.45813600	-1.46135800	-0.22074400

Molecule:Cu-MET-01

N	0.35425300	3.13465400	0.18514600
H	0.54193100	3.22529900	-0.81765000
H	1.18331700	3.45564900	0.69019800
H	-0.42980800	3.74322400	0.43185200
C	0.04003400	1.71128600	0.52043200
H	0.00677400	1.63485700	1.60394700
C	-1.31429600	1.34455900	-0.09151100
C	1.17034300	0.84726200	-0.02590800
H	-2.04649000	2.05368600	0.30172500
H	-1.26373300	1.48379200	-1.17369400
C	-1.74281900	-0.07676700	0.24207700
O	1.80949400	1.19372600	-1.01576500
H	-1.10581400	-0.80528600	-0.25784100
H	-1.68732600	-0.25165100	1.31752400
S	-3.45927000	-0.34113400	-0.30787500
C	-3.62729400	-2.09116800	0.12881400
H	-3.45996700	-2.24059000	1.19550800
H	-4.64597000	-2.39044100	-0.11514600
H	-2.93030500	-2.70353200	-0.44310900
O	1.34628300	-0.25173300	0.62959800
Cu	2.63117000	-1.43573800	-0.02267000

Molecule:Cu-MET-02

N	2.62156100	-1.79963400	-0.56281300
H	2.84507800	-2.02134000	0.41240000
H	3.48684900	-1.88994800	-1.09857000
H	1.94096700	-2.46973600	-0.92597800
C	2.10879200	-0.39531000	-0.63085600
H	2.10472500	-0.10681700	-1.67890600
C	0.68980600	-0.37187500	-0.05656000
C	3.06608900	0.51236800	0.16996500
H	0.12917900	-1.19128200	-0.51703200
H	0.73672700	-0.55712300	1.01918400
C	-0.01403200	0.94366500	-0.35257500
O	3.76953500	-0.04968500	1.04369800
H	0.55051500	1.80169900	0.00961300
H	-0.21335100	1.06700900	-1.41577300
S	-1.61521700	1.00679400	0.51850900
C	-2.60605800	2.03128600	-0.58466100
H	-2.61277900	1.60849700	-1.58653600
H	-3.61119900	2.07865700	-0.17151800
H	-2.15892000	3.02542700	-0.59211500
O	3.02296800	1.73179800	-0.10886800
Cu	-2.39699500	-1.19427700	0.11856100

Molecule:Cu-MET-011

N	0.58180300	2.90467900	0.06677300
H	0.82206400	2.88007300	-0.92859200
H	1.41597600	3.18722900	0.58737000
H	-0.14116500	3.61514600	0.20633600
C	0.08325600	1.56994600	0.52361700
H	-0.07326000	1.63261100	1.59791100
C	-1.23026400	1.25546800	-0.20035500
C	1.14771900	0.54047600	0.23690700
H	-1.90158800	2.09675000	-0.01526300
H	-1.03774500	1.21041200	-1.27462400
C	-1.87607300	-0.03332800	0.28520800
O	1.96024000	0.68631900	-0.71373500
H	-1.26653400	-0.89965500	0.02998200
H	-2.00788800	-0.00943400	1.36794400
S	-3.51108100	-0.22141800	-0.49678200
C	-4.03635600	-1.75417400	0.31213500
H	-4.08090300	-1.62364800	1.39323300
H	-5.03366300	-1.98795300	-0.05893100
H	-3.36370000	-2.57579600	0.06673400
O	1.21817100	-0.51308600	0.94711400
Cu	2.71779700	-1.21282700	-0.14838300

Molecule:Cu-MET-032

N	1.88251800	-2.33951500	-0.92664100
H	2.01014400	-2.92254600	-0.09445900
H	2.81122600	-2.10227800	-1.28232800
H	1.38683900	-2.87976600	-1.63937800
C	1.11128300	-1.11090100	-0.56456900
H	1.10638700	-0.46094300	-1.43812400
C	-0.31654100	-1.52632900	-0.18746900
C	1.85605000	-0.44058400	0.59109500
H	-0.66063400	-2.23240300	-0.94662400
H	-0.29109400	-2.05417000	0.76831900
C	-1.27914600	-0.35132800	-0.13271400
O	2.52408300	-1.13011500	1.35631800
H	-0.96900100	0.37654200	0.62091500
H	-1.31167800	0.14894100	-1.10256700
S	-2.95885800	-0.91624600	0.27958600
C	-3.82603500	0.66388600	0.09957600
H	-3.73759400	1.03761100	-0.92048200
H	-4.87730900	0.48529200	0.32234000
H	-3.43544600	1.40255800	0.79949600
O	1.73984600	0.83770700	0.73379600
Cu	0.78438500	2.04029300	-0.29760100

Molecule:Fe-MET-011

N	0.67632900	2.87529200	0.08786800
H	0.88272200	2.87988200	-0.91539200
H	1.53712500	3.11357600	0.58712700
H	-0.01905400	3.60157900	0.27969700
C	0.15938600	1.54173800	0.51868200
H	0.01696900	1.58285800	1.59622400
C	-1.17241000	1.27534200	-0.19014300
C	1.19696700	0.48893700	0.20842700
H	-1.83455500	2.10992300	0.04615000
H	-1.00262900	1.27103900	-1.26859600
C	-1.80721500	-0.03161200	0.26101800
O	2.08697800	0.67797000	-0.66070600
H	-1.19815600	-0.89884400	-0.00666700
H	-1.96500200	-0.05331300	1.34137200
S	-3.41209200	-0.24492000	-0.52978800
C	-4.03039800	-1.67483700	0.35265000
H	-4.11990200	-1.44024700	1.41437200
H	-5.00633800	-1.91764900	-0.06244200
H	-3.34444800	-2.51269900	0.21821200
O	1.15783900	-0.61581000	0.83703800
Fe	2.84138100	-1.30919700	-0.14165400

Molecule:Fe-MET-023

N	-2.83533900	-1.89249400	-0.10660800
H	-3.28895500	-1.63697000	-0.99096800
H	-3.57144300	-2.18691900	0.53758100
H	-2.19675700	-2.67700300	-0.25144400
C	-2.13256900	-0.67553100	0.41255600
H	-1.97013900	-0.82284500	1.47704700
C	-0.79874900	-0.53865000	-0.32334600
C	-3.05563800	0.54151800	0.18289700
H	-0.24021900	-1.46846300	-0.18884500
H	-0.99179700	-0.41552500	-1.39163700
C	0.02053500	0.63025200	0.20011000
O	-3.90218200	0.43710500	-0.73900000
H	-0.47693800	1.58360400	0.03279800
H	0.24627000	0.53109600	1.26106200
S	1.59371000	0.73355300	-0.71065700
C	2.35910200	2.16153100	0.09237400
H	2.38649900	2.01450900	1.16991600
H	3.36724800	2.25404000	-0.30670900
H	1.77822000	3.04704800	-0.16036900
O	-2.85083300	1.53044100	0.92168900
Fe	2.88361800	-1.04488700	0.23381800

Molecule:Fe-MET-32

N	2.04976300	-2.25621100	-0.82780800
H	2.15067900	-2.82369400	0.01923700
H	2.98681000	-1.97500200	-1.12583600
H	1.63375800	-2.83116800	-1.56461600
C	1.19389900	-1.06547200	-0.53985900
H	1.22801400	-0.42204900	-1.41693800
C	-0.23297200	-1.55947700	-0.28198000
C	1.80216300	-0.34157900	0.66783400
H	-0.54932700	-2.11227400	-1.16919700
H	-0.22626200	-2.24984700	0.56334400
C	-1.18605600	-0.40885300	-0.01440500
O	2.39655900	-0.98826800	1.51937300
H	-1.00391900	0.06925400	0.94942500
H	-1.08355900	0.36738500	-0.79199200
S	-2.90391000	-0.91628600	-0.05739200
C	-3.68559900	0.59231200	0.51538700
H	-3.43927300	1.40876500	-0.16580100
H	-4.76036100	0.42544700	0.52466100
H	-3.32889000	0.82983600	1.51851800
O	1.63772400	0.94296900	0.73243700
Fe	0.72629500	2.18570000	-0.41247000

Molecule:Hg-MET-02

N	3.25443600	-2.02571500	-0.41185600
H	3.54248800	-2.16967200	0.56194100
H	4.04475300	-2.29278700	-1.00134500
H	2.46056500	-2.62754500	-0.63870500
C	2.93280200	-0.57564100	-0.58979700
H	2.92500900	-0.37319500	-1.65779400
C	1.55728700	-0.30570400	0.02365000
C	4.03958800	0.24906100	0.10498400
H	0.85114100	-1.05191600	-0.35553700
H	1.61995700	-0.42637700	1.10775100
C	1.05010100	1.07671000	-0.34571500
O	4.68666800	-0.33716800	1.00662500
H	1.74449100	1.86075100	-0.04361900
H	0.84574800	1.16509200	-1.41067500
S	-0.48661100	1.46921500	0.55029200
C	-1.19865500	2.76130300	-0.49408000
H	-1.26699400	2.42355600	-1.52347800
H	-2.18130800	2.99420400	-0.09042700
H	-0.54201700	3.62614200	-0.40660900
O	4.15931600	1.43518000	-0.27463200
Hg	-1.87617100	-0.50592700	0.01858300

Molecule:Hg-MET-03

N	0.58607300	3.34683600	-1.02210000
H	1.01055200	3.91325000	-0.28166100
H	-0.33058100	3.74757600	-1.23199900
H	1.16716100	3.40236700	-1.86190600
C	0.46112500	1.93002600	-0.55873400
H	-0.10275900	1.39156600	-1.31724100
C	1.86744600	1.34387000	-0.41185800
C	-0.30822200	1.95037700	0.76513100
H	2.37179900	1.47035500	-1.37259100
H	2.41516600	1.92753100	0.33166600
C	1.86135400	-0.12532100	-0.02458100
O	-0.19102700	2.92910200	1.50054800
H	1.46973900	-0.26341100	0.98228700
H	1.24027900	-0.69976200	-0.72035600
S	3.54807200	-0.80592900	-0.07833000
C	3.18446800	-2.46885900	0.54145700
H	2.45979300	-2.96796400	-0.10211800
H	4.11692500	-3.03193000	0.52852400
H	2.80580400	-2.42828800	1.56245700
O	-1.03823500	0.93506300	1.09387100
Hg	-1.40073100	-0.79610200	-0.13416200

Molecule:Hg-MET-011

N	-1.52727700	3.37512600	0.14104000
H	-1.31828200	3.48468900	-0.85555400
H	-0.84386700	3.92798300	0.66338500
H	-2.46118300	3.74717100	0.32715900
C	-1.45397500	1.93016500	0.52083800
H	-1.56047100	1.87481200	1.60072900
C	-2.58625800	1.17495200	-0.18193100
C	-0.08208300	1.40888900	0.09226500
H	-3.50742200	1.73648200	-0.01057200
H	-2.39552200	1.17316400	-1.25770400
C	-2.75922600	-0.24375700	0.33840800
O	0.51847000	1.94677400	-0.83300100
H	-1.86226100	-0.83825800	0.17279600
H	-2.97238200	-0.23031700	1.40830100
S	-4.15187300	-1.04132800	-0.52565300
C	-4.16278400	-2.62459300	0.35369700
H	-4.37847600	-2.47928200	1.41216000
H	-4.94968700	-3.23415500	-0.08911300
H	-3.20884700	-3.13936000	0.23970300
O	0.30132900	0.37451100	0.76068800
Hg	2.07858600	-0.51789700	-0.02936600

Molecule:Mn-MET-11

N	0.40391000	3.05590400	0.13621800
H	0.67248200	3.06539100	-0.85235600
H	1.19944800	3.39906500	0.67823500
H	-0.38458100	3.69047000	0.27921500
C	0.04947400	1.65639900	0.53423700
H	-0.03717800	1.64183400	1.61714700
C	-1.27896000	1.28462700	-0.12621700
C	1.18929600	0.74430300	0.07882300
H	-2.01618100	2.03632300	0.16610800
H	-1.15960200	1.34125800	-1.21095300
C	-1.76490600	-0.09765200	0.28179300
O	1.80708700	1.04998300	-0.95699000
H	-1.08918200	-0.87242900	-0.07905200
H	-1.82956500	-0.17505200	1.36813600
S	-3.41831000	-0.39867300	-0.42310000
C	-3.71416000	-2.05408100	0.24966400
H	-3.70873100	-2.03416800	1.33947300
H	-4.69721000	-2.37430400	-0.09396100
H	-2.96583400	-2.76005500	-0.11029800
O	1.40097800	-0.28802800	0.79052800
Mn	3.01331000	-1.49072500	-0.08254900

Molecule:Mn-MET-21

N	-2.49762200	-1.87627600	0.48145300
H	-2.75864500	-2.03825100	-0.49686500
H	-3.33511400	-2.02283900	1.04796400
H	-1.78501400	-2.54861600	0.77134800
C	-2.01648000	-0.46399900	0.61003900
H	-1.99859400	-0.22601500	1.67046800
C	-0.61431400	-0.36625500	0.00643700
C	-3.01892700	0.44722500	-0.13068900
H	-0.00828800	-1.18993700	0.39889000
H	-0.68444500	-0.49701500	-1.07637000
C	0.05656800	0.95380400	0.35210700
O	-3.64477000	-0.07915600	-1.08430000
H	-0.56976800	1.80343800	0.09163400
H	0.29292200	1.00029500	1.41400900
S	1.62004800	1.12029400	-0.57551100
C	2.57334400	2.19196700	0.53203800
H	2.66710200	1.73871000	1.51613800
H	3.55885600	2.31566300	0.08645600
H	2.08214200	3.16059600	0.60307500
O	-3.08931600	1.63213100	0.26478200
Mn	2.64371900	-1.41148200	-0.07408800

Molecule:Mn-MET-31

N	2.65665400	-2.11896400	-0.28035300
H	2.83234100	-2.15249700	0.72870100
H	3.52692000	-1.84770800	-0.74230600
H	2.39460300	-3.05207100	-0.60618300
C	1.57171700	-1.12300500	-0.55158600
H	1.56781700	-0.93301300	-1.62091000
C	0.24180500	-1.72896400	-0.09791700
C	1.91122500	0.16917300	0.20865200
H	0.03415400	-2.58830800	-0.73995800
H	0.35137600	-2.09536800	0.92535700
C	-0.90335100	-0.73198100	-0.16246200
O	2.44014100	0.06115700	1.32469300
H	-0.78830000	0.02125200	0.61810500
H	-0.93160500	-0.23110400	-1.13138300
S	-2.50098600	-1.55505000	0.11739100
C	-3.54202800	-0.07333500	0.17341500
H	-3.48210600	0.47545100	-0.76657800
H	-4.56957900	-0.39932600	0.32911200
H	-3.24531900	0.57573500	0.99781400
O	1.60420700	1.25143500	-0.38416000
Mn	-0.17208400	2.49473700	-0.11409700

Molecule:Ni-MET-01

N	0.40391000	3.05590400	0.13621800
H	0.67248200	3.06539100	-0.85235600
H	1.19944800	3.39906500	0.67823500
H	-0.38458100	3.69047000	0.27921500
C	0.04947400	1.65639900	0.53423700
H	-0.03717800	1.64183400	1.61714700
C	-1.27896000	1.28462700	-0.12621700
C	1.18929600	0.74430300	0.07882300
H	-2.01618100	2.03632300	0.16610800
H	-1.15960200	1.34125800	-1.21095300
C	-1.76490600	-0.09765200	0.28179300
O	1.80708700	1.04998300	-0.95699000
H	-1.08918200	-0.87242900	-0.07905200
H	-1.82956500	-0.17505200	1.36813600
S	-3.41831000	-0.39867300	-0.42310000
C	-3.71416000	-2.05408100	0.24966400
H	-3.70873100	-2.03416800	1.33947300
H	-4.69721000	-2.37430400	-0.09396100
H	-2.96583400	-2.76005500	-0.11029800
O	1.40097800	-0.28802800	0.79052800
Mn	3.01331000	-1.49072500	-0.08254900

Molecule:Ni-MET-32

N	0.66241900	2.85129800	0.04763400
H	0.89160900	2.81178400	-0.95005600
H	1.50788700	3.11906100	0.55830800
H	-0.04172600	3.58102600	0.18597700
C	0.13157500	1.53718900	0.52480300
H	-0.02045700	1.61803300	1.59836300
C	-1.19042400	1.24596900	-0.19282100
C	1.16308200	0.47303000	0.25873800
H	-1.84388800	2.09894000	0.00201300
H	-1.00483300	1.20350400	-1.26825800
C	-1.85696200	-0.03424500	0.28838300
O	1.96660000	0.56712800	-0.71923700
H	-1.27014900	-0.91089100	0.01460300
H	-1.97483600	-0.02042100	1.37283100
S	-3.50313600	-0.17261500	-0.47923400
C	-4.04461300	-1.72545700	0.27840200
H	-4.07359200	-1.63658400	1.36425100
H	-5.05053000	-1.92683900	-0.08850200
H	-3.39013200	-2.54875700	-0.00738100
O	1.23135500	-0.55713800	0.99315100
Ni	2.74586800	-1.20160500	-0.16372100

Molecule:Zn-MET-01

N	0.49419600	2.91376800	0.04165700
H	0.74988100	2.86889400	-0.94856600
H	1.32079000	3.20958200	0.56557800
H	-0.23435800	3.62193900	0.15906800
C	0.00491100	1.58080000	0.51542400
H	-0.17590500	1.66890100	1.58421100
C	-1.29376400	1.24163700	-0.22434300
C	1.09122300	0.55013700	0.27849000
H	-1.97342100	2.08393800	-0.07555500
H	-1.08340200	1.16901200	-1.29394600
C	-1.94420200	-0.03688500	0.28190000
O	1.98146700	0.77122400	-0.59091800
H	-1.33583100	-0.90693900	0.04088100
H	-2.07408800	0.00373300	1.36447200
S	-3.58058400	-0.24708500	-0.49283500
C	-4.05378200	-1.81020400	0.29045500
H	-4.08817500	-1.70328100	1.37459200
H	-5.04802200	-2.06789100	-0.07275100
H	-3.35964700	-2.60618600	0.02115900
O	1.05855700	-0.52566900	0.93343700
Zn	2.79952100	-1.16340300	-0.15723600

Molecule:Zn-MET-23

N	3.00319800	-0.86434600	-1.31903400
H	2.86083600	-1.68505400	-0.72045600
H	4.00738600	-0.67970600	-1.35511200
H	2.67383000	-1.06054300	-2.26643100
C	2.27508700	0.28903200	-0.70005000
H	2.65142100	1.19804600	-1.16046500
C	0.78240400	0.10800500	-0.97774500
C	2.57707200	0.28459400	0.81373700
H	0.63177100	0.07110200	-2.05896700
H	0.47833500	-0.86130600	-0.56895800
C	-0.05980700	1.21674300	-0.36829100
O	2.78368900	-0.84041400	1.33302200
H	0.04710800	1.26074100	0.71215600
H	0.18855700	2.18902000	-0.79291400
S	-1.82751700	0.91769700	-0.72489800
C	-2.62009300	1.97820600	0.50993100
H	-2.38970000	3.01256600	0.26047800
H	-3.69245900	1.81093500	0.43338900
H	-2.26738600	1.73447000	1.50800200
O	2.54870900	1.39642900	1.38631700
Zn	-1.91196600	-1.44435300	0.31402300

Molecule:Zn-MET-32

N	1.84758000	-2.37458400	-0.95346300
H	1.99211600	-2.94955500	-0.11781400
H	2.76938900	-2.14130700	-1.32784100
H	1.33623300	-2.91579900	-1.65376300
C	1.09520000	-1.13992100	-0.57008900
H	1.13700500	-0.45688800	-1.41640400
C	-0.35056500	-1.53263000	-0.25282600
C	1.81023600	-0.53450600	0.64228600
H	-0.69996100	-2.18244300	-1.05852500
H	-0.36172300	-2.11738500	0.67010200
C	-1.28165400	-0.33639400	-0.13769800
O	2.44239200	-1.28727200	1.38805500
H	-0.97079700	0.32980500	0.66768700
H	-1.28858500	0.22823000	-1.07247300
S	-2.97548400	-0.91091300	0.20899300
C	-3.82726800	0.68668100	0.19809400
H	-3.74257900	1.16353500	-0.77828800
H	-4.87873200	0.49393400	0.40877700
H	-3.42670100	1.34691500	0.96719900
O	1.68815200	0.72507200	0.84252400
Zn	0.83629900	2.06786300	-0.30271800

Molecule: Cd-PRO-02

N	-2.68906400	0.41667500	0.68304900
H	-2.88686900	1.36200000	0.33417200
H	-2.88150200	0.39122700	1.68337000
C	-3.50012900	-0.63564900	-0.04258200
C	-1.22473200	0.14794000	0.42714900
H	-3.82250900	-0.19516300	-0.98239500
H	-4.36582900	-0.88918100	0.56097700
C	-2.51128400	-1.76792700	-0.26163500
C	-1.20923700	-1.02882900	-0.56018200
H	-0.77066500	-0.13014500	1.37516900
C	-0.55207400	1.40815100	-0.10972300
H	-2.82292000	-2.41710900	-1.07726400
H	-2.41283400	-2.36743300	0.64483000
H	-1.21163800	-0.65497100	-1.58526000
H	-0.32003200	-1.64094200	-0.41728800
O	-1.25368000	2.39104500	-0.37852100
O	0.71913700	1.37502700	-0.27415600
Cd	2.05373600	-0.31761900	0.06636700

Molecule: Cd-PRO-011

N	2.46287500	-1.03421500	0.55079700
H	1.78346600	-1.72474900	0.21874800
H	2.78311100	-1.31793900	1.47615800
C	3.63176800	-0.90659300	-0.40346000
C	1.77252000	0.31259300	0.64120500
H	3.29909000	-1.29101800	-1.36404600
H	4.45286300	-1.51003100	-0.03021900
C	3.89607700	0.58780300	-0.45348800
C	2.49386000	1.19184400	-0.39156400
H	1.92369900	0.68888000	1.64910800
C	0.28979900	0.16500600	0.37557600
H	4.43256400	0.86498200	-1.35854000
H	4.48562100	0.89765900	0.41078400
H	2.00160900	1.10694600	-1.36142100
H	2.48217500	2.23365500	-0.08359200
O	-0.14347000	-0.92719200	-0.08262300
O	-0.44868000	1.16010700	0.59724500
Cd	-2.34690100	-0.05575200	-0.12789900

Molecule: Cd-PRO-032

N	1.58945300	-0.33049700	1.21892600
H	0.73288000	-0.11860100	1.73520300
H	2.38261100	-0.23148600	1.85088700
C	1.53586600	-1.73084000	0.65083900
C	1.73578700	0.64925900	0.06682300
H	0.48444700	-2.00642800	0.58159400
H	2.04182400	-2.40496700	1.33493900
C	2.18075200	-1.58990700	-0.71434900
C	1.68934900	-0.22536800	-1.19364400
H	2.70551700	1.12383100	0.17620100
C	0.64872100	1.71739300	0.10274800
H	1.88817600	-2.40015100	-1.37881200
H	3.26788600	-1.59200500	-0.62064800
H	0.66331500	-0.30302400	-1.56179300
H	2.30144500	0.20446200	-1.98218600
O	-0.49392900	1.38741400	0.60235700
O	0.91684100	2.83131400	-0.34686500
Cd	-1.61917500	-0.34648300	-0.08721500

Molecule: Cu-PRO-01

N	1.87598800	-1.07393400	0.47127500
H	1.25844500	-1.77156300	0.04905700
H	2.14380600	-1.41372200	1.39495500
C	3.10543700	-0.83964500	-0.38740200
C	1.15025200	0.24638500	0.61400600
H	2.85174500	-1.16349200	-1.39308300
H	3.91821900	-1.44361400	0.00250100
C	3.32047700	0.66128400	-0.31208100
C	1.89808900	1.21692500	-0.31219800
H	1.23898000	0.55625100	1.65278600
C	-0.30464100	0.10087600	0.28815700
H	3.90969500	1.01885400	-1.15356900
H	3.83390100	0.92656700	0.61345900
H	1.47172600	1.17854700	-1.31546400
H	1.82574200	2.23385000	0.06292800
O	-0.79023700	-0.97946400	-0.16122000
O	-1.08346100	1.08576000	0.46057400
Cu	-2.60732000	-0.06101500	-0.17069700

Molecule: Cu-PRO-021

N	-2.20691000	0.57088100	0.63241900
H	-2.27230000	1.54068600	0.30348100
H	-2.48989000	0.53499800	1.61082600
C	-3.05729700	-0.36268400	-0.19428300
C	-0.76660700	0.13343900	0.49707600
H	-3.14920600	0.08286500	-1.18194700
H	-4.03684600	-0.44650400	0.26564700
C	-2.24155700	-1.64148800	-0.22681400
C	-0.80837200	-1.13150000	-0.38017900
H	-0.39466800	-0.08907400	1.49455000
C	0.05167100	1.27243400	-0.09080700
H	-2.54571900	-2.28773200	-1.04747000
H	-2.35972200	-2.18733800	0.71056100
H	-0.61019800	-0.86937400	-1.41986300
H	-0.05800700	-1.84916000	-0.05568400
O	-0.49300400	2.33566100	-0.38148300
O	1.31300500	1.05226100	-0.26482800
Cu	2.33578900	-0.52241700	0.08391400

Molecule: Cu-PRO-032

N	1.35706000	0.00178200	1.21833000
H	0.49025300	-0.18156300	1.72796500
H	2.03793300	0.39005600	1.86955500
C	1.88354900	-1.27655000	0.59622100
C	1.09434900	0.99853000	0.10299500
H	1.03639100	-1.95277500	0.50554500
H	2.62893900	-1.70554400	1.25806300
C	2.39363600	-0.83236600	-0.76122900
C	1.35940300	0.20668400	-1.18714800
H	1.79802400	1.81572000	0.22382900
C	-0.32765200	1.52983100	0.14002800
H	2.46035700	-1.66885300	-1.45360700
H	3.38023800	-0.37571100	-0.66718000
H	0.43924100	-0.28899000	-1.50950000
H	1.69766900	0.86812000	-1.98014200
O	-1.21322300	0.70868000	0.62769000
O	-0.58213300	2.63716300	-0.30771900
Cu	-1.70777000	-0.94608400	-0.15199300

Molecule: Fe-PRO-01

N	1.83503100	-1.12017100	0.34379700
H	1.31853100	-1.76644900	-0.25671400
H	1.97972800	-1.58407000	1.24093400
C	3.17101900	-0.75642000	-0.30299700
C	1.06998800	0.16114200	0.54905300
H	3.12118100	-1.10480800	-1.33005700
H	3.95824600	-1.27842600	0.23012700
C	3.23646800	0.75992200	-0.21613600
C	1.77843100	1.19108900	-0.34172200
H	1.17264700	0.43977400	1.59716800
C	-0.38308400	0.02834800	0.26187400
H	3.86069300	1.17123200	-1.00611000
H	3.63879500	1.07169900	0.74829100
H	1.42828800	1.09530800	-1.37061900
H	1.58407600	2.19974400	0.01104900
O	-0.94059200	-1.01375900	-0.19979600
O	-1.15349000	1.02294200	0.47832200
Fe	-2.74583400	-0.03002900	-0.16151100

Molecule: Fe-PRO-022

N	-2.12262900	0.63280400	0.62463000
H	-2.21335900	1.57701200	0.23886800
H	-2.33669400	0.67923200	1.62066900
C	-3.05486700	-0.34493900	-0.06973700
C	-0.71017300	0.14263100	0.43428500
H	-3.31043500	0.09199300	-1.03094600
H	-3.94759100	-0.45895000	0.53612600
C	-2.21555400	-1.60190200	-0.22317900
C	-0.82469700	-1.05184600	-0.52410700
H	-0.33273500	-0.16984400	1.40599400
C	0.16861800	1.24517700	-0.10260700
H	-2.59537300	-2.23635700	-1.02082000
H	-2.20854500	-2.17251400	0.70648100
H	-0.76340200	-0.70778000	-1.55728500
H	-0.01979600	-1.76325000	-0.34484900
O	-0.23871200	2.34575800	-0.39542000
O	1.44297700	0.90692200	-0.26645700
Fe	2.41431800	-0.60097500	0.12617100

Molecule: Fe-PRO-031

N	1.33068500	0.12697800	1.21075800
H	0.53087400	-0.04597500	1.82133300
H	2.05521700	0.58366600	1.76621200
C	1.86086700	-1.16956000	0.63579300
C	0.97036700	1.03728800	0.04939300
H	1.01512600	-1.85110100	0.56599600
H	2.60691400	-1.57160900	1.31368200
C	2.36648200	-0.76458500	-0.73291100
C	1.29280300	0.21069300	-1.20929800
H	1.60421800	1.91515600	0.11839300
C	-0.47396000	1.48764200	0.09384100
H	2.46676500	-1.62469000	-1.39091100
H	3.33375200	-0.26654000	-0.65254200
H	0.41859400	-0.34888600	-1.54860500
H	1.61105400	0.86303900	-2.01784700
O	-1.35225600	0.56469700	0.52242100
O	-0.82751200	2.58463100	-0.25294600
Fe	-1.67763500	-1.09790000	-0.13952800

Molecule: Hg-PRO-01

N	3.35768700	-1.03265900	0.36000600
H	2.87593700	-1.79276400	-0.13010200
H	3.72674800	-1.40108700	1.23607900
C	4.47353000	-0.44651100	-0.48331000
C	2.35634300	0.06306000	0.64560600
H	4.26684200	-0.72084900	-1.51438100
H	5.41503800	-0.88361500	-0.16700300
C	4.34920300	1.05106600	-0.26204800
C	2.83977100	1.26451100	-0.17883300
H	2.39477600	0.27435100	1.71042700
C	0.95768900	-0.39699700	0.26201800
H	4.80800400	1.60969000	-1.07525100
H	4.82910700	1.33922400	0.67447000
H	2.39618800	1.23295600	-1.17547000
H	2.55563100	2.19774600	0.29946000
O	0.77555000	-1.44473400	-0.34958300
O	0.06182200	0.45717900	0.62713600
Hg	-1.91662800	0.05078300	-0.05624100

Molecule: Hg-PRO-012

N	2.99677700	-1.01918500	0.58828300
H	2.31366200	-1.71792600	0.28521600
H	3.31708400	-1.27113300	1.52297600
C	4.16390100	-0.94472800	-0.37642600
C	2.32971700	0.34146600	0.63148600
H	3.82546000	-1.37725000	-1.31403100
H	4.98383900	-1.53102200	0.02533100
C	4.43475100	0.54343500	-0.50554100
C	3.03670700	1.15804600	-0.46018000
H	2.51395800	0.76167600	1.61580500
C	0.83626700	0.22461500	0.41135700
H	4.96231200	0.76944400	-1.42982100
H	5.03506800	0.89529500	0.33475900
H	2.52915300	1.02034400	-1.41600500
H	3.03223900	2.21530500	-0.20999600
O	0.37519000	-0.85813100	-0.05050900
O	0.12778600	1.22443400	0.66933800
Hg	-1.82902600	-0.04372300	-0.08358800

Molecule: Hg-PRO-021

N	-3.12659500	0.38945300	0.69570700
H	-3.35980500	1.33160800	0.36211400
H	-3.30190200	0.34957100	1.69898900
C	-3.92485900	-0.67569500	-0.02769200
C	-1.66219200	0.15335000	0.41742300
H	-4.26990000	-0.23402600	-0.95878200
H	-4.77542300	-0.95286200	0.58675900
C	-2.91403500	-1.78362900	-0.27043100
C	-1.63303800	-1.01418800	-0.58250600
H	-1.18798600	-0.12462300	1.35563400
C	-1.02799000	1.43046600	-0.11313200
H	-3.22311000	-2.43241300	-1.08721800
H	-2.78908600	-2.38803600	0.62936100
H	-1.65872800	-0.63138800	-1.60379200
H	-0.72582600	-1.60250900	-0.45503400
O	-1.73721100	2.40073500	-0.37602700
O	0.25496800	1.45976900	-0.29361100
Hg	1.57510700	-0.19484200	0.04271400

Molecule: Hg-PRO-032

N	1.86250600	-0.44268000	1.21988800
H	1.01905100	-0.18379100	1.73279200
H	2.65124200	-0.38927500	1.86372100
C	1.73226900	-1.84198100	0.65662100
C	2.10742200	0.50903200	0.05443900
H	0.66773100	-2.04421400	0.55256200
H	2.17582000	-2.54252800	1.35706100
C	2.40812300	-1.75138700	-0.69696900
C	1.98471100	-0.37089400	-1.19340300
H	3.12527200	0.86745100	0.16621700
C	1.18979400	1.72585400	0.06518900
H	2.08539500	-2.55434000	-1.35603700
H	3.49272500	-1.80275600	-0.58840100
H	0.94890100	-0.39782800	-1.54087000
H	2.60704500	0.01652400	-1.99607400
O	-0.07319700	1.59368900	0.33483200
O	1.68099700	2.82240400	-0.18586400
Hg	-1.26508800	-0.16028700	-0.04046500

Molecule: Fe-PRO-01

N	1.83503100	-1.12017100	0.34379700
H	1.31853100	-1.76644900	-0.25671400
H	1.97972800	-1.58407000	1.24093400
C	3.17101900	-0.75642000	-0.30299700
C	1.06998800	0.16114200	0.54905300
H	3.12118100	-1.10480800	-1.33005700
H	3.95824600	-1.27842600	0.23012700
C	3.23646800	0.75992200	-0.21613600
C	1.77843100	1.19108900	-0.34172200
H	1.17264700	0.43977400	1.59716800
C	-0.38308400	0.02834800	0.26187400
H	3.86069300	1.17123200	-1.00611000
H	3.63879500	1.07169900	0.74829100
H	1.42828800	1.09530800	-1.37061900
H	1.58407600	2.19974400	0.01104900
O	-0.94059200	-1.01375900	-0.19979600
O	-1.15349000	1.02294200	0.47832200
Fe	-2.74583400	-0.03002900	-0.16151100

Molecule: Fe-PRO-022

N	-2.12262900	0.63280400	0.62463000
H	-2.21335900	1.57701200	0.23886800
H	-2.33669400	0.67923200	1.62066900
C	-3.05486700	-0.34493900	-0.06973700
C	-0.71017300	0.14263100	0.43428500
H	-3.31043500	0.09199300	-1.03094600
H	-3.94759100	-0.45895000	0.53612600
C	-2.21555400	-1.60190200	-0.22317900
C	-0.82469700	-1.05184600	-0.52410700
H	-0.33273500	-0.16984400	1.40599400
C	0.16861800	1.24517700	-0.10260700
H	-2.59537300	-2.23635000	-1.02082000
H	-2.20854500	-2.17251400	0.70648100
H	-0.76340200	-0.70778000	-1.55728500
H	-0.01979600	-1.76325000	-0.34484900
O	-0.23871200	2.34575800	-0.39542000
O	1.44297700	0.90692200	-0.26645700
Fe	2.41431800	-0.60097500	0.12617100

Molecule: Hg-PRO-01

N	3.35768700	-1.03265900	0.36000600
H	2.87593700	-1.79276400	-0.13010200
H	3.72674800	-1.40108700	1.23607900
C	4.47353000	-0.44651100	-0.48331000
C	2.35634300	0.06306000	0.64560600
H	4.26684200	-0.72084900	-1.51438100
H	5.41503800	-0.88361500	-0.16700300
C	4.34920300	1.05106600	-0.26204800
C	2.83977100	1.26451100	-0.17883300
H	2.39477600	0.27435100	1.71042700
C	0.95768900	-0.39699700	0.26201800
H	4.80800400	1.60969000	-1.07525100
H	4.82910700	1.33922400	0.67447000
H	2.39618800	1.23295600	-1.17547000
H	2.55563100	2.19774600	0.29946000
O	0.77555000	-1.44473400	-0.34958300
O	0.06182200	0.45717900	0.62713600
Hg	-1.91662800	0.05078300	-0.05624100

Molecule: Hg-PRO-012

N	2.99677700	-1.01918500	0.58828300
H	2.31366200	-1.71792600	0.28521600
H	3.31708400	-1.27113300	1.52297600
C	4.16390100	-0.94472800	-0.37642600
C	2.32971700	0.34146600	0.63148600
H	3.82546000	-1.37725000	-1.31403100
H	4.98383900	-1.53102200	0.02533100
C	4.43475100	0.54343500	-0.50554100
C	3.03670700	1.15804600	-0.46018000
H	2.51395800	0.76167600	1.61580500
C	0.83626700	0.22461500	0.41135700
H	4.96231200	0.76944400	-1.42982100
H	5.03506800	0.89529500	0.33475900
H	2.52915300	1.02034400	-1.41600500
H	3.03223900	2.21530500	-0.20999600
O	0.37519000	-0.85813100	-0.05050900
O	0.12778600	1.22443400	0.66933800
Hg	-1.82902600	-0.04372300	-0.08358800

Molecule: Hg-PRO-021

N	-3.12659500	0.38945300	0.69570700
H	-3.35980500	1.33160800	0.36211400
H	-3.30190200	0.34957100	1.69898900
C	-3.92485900	-0.67569500	-0.02769200
C	-1.66219200	0.15335000	0.41742300
H	-4.26990000	-0.23402600	-0.95878200
H	-4.77542300	-0.95286200	0.58675900
C	-2.91403500	-1.78362900	-0.27043100
C	-1.63303800	-1.01418800	-0.58250600
H	-1.18798600	-0.12462300	1.35563400
C	-1.02799000	1.43046600	-0.11313200
H	-3.22311000	-2.43241300	-1.08721800
H	-2.78908600	-2.38803600	0.62936100
H	-1.65872800	-0.63138800	-1.60379200
H	-0.72582600	-1.60250900	-0.45503400
O	-1.73721100	2.40073500	-0.37602700
O	0.25496800	1.45976900	-0.29361100
Hg	1.57510700	-0.19484200	0.04271400

Molecule: Hg-PRO-032

N	1.86250600	-0.44268000	1.21988800
H	1.01905100	-0.18379100	1.73279200
H	2.65124200	-0.38927500	1.86372100
C	1.73226900	-1.84198100	0.65662100
C	2.10742200	0.50903200	0.05443900
H	0.66773100	-2.04421400	0.55256200
H	2.17582000	-2.54252800	1.35706100
C	2.40812300	-1.75138700	-0.69696900
C	1.98471100	-0.37089400	-1.19340300
H	3.12527200	0.86745100	0.16621700
C	1.18979400	1.72585400	0.06518900
H	2.08539500	-2.55434000	-1.35603700
H	3.49272500	-1.80275600	-0.58840100
H	0.94890100	-0.39782800	-1.54087000
H	2.60704500	0.01652400	-1.99607400
O	-0.07319700	1.59368900	0.33483200
O	1.68099700	2.82240400	-0.18586400
Hg	-1.26508800	-0.16028700	-0.04046500

Molecule: Mn-PRO-012

N	-2.12702700	1.02960400	0.38320600
H	-1.61166200	1.77837300	-0.09405500
H	-2.48513200	1.39273200	1.26534100
C	-3.25468200	0.48901500	-0.46766600
C	-1.14543600	-0.09143500	0.64261100
H	-3.03175700	0.75568800	-1.49764900
H	-4.18302300	0.95920000	-0.15910900
C	-3.18454700	-1.01223800	-0.24779800
C	-1.68215100	-1.27884300	-0.16958500
H	-1.16212600	-0.30306700	1.70834100
C	0.25346800	0.34331700	0.22654700
H	-3.66842800	-1.55483900	-1.05748300
H	-3.67057100	-1.28162500	0.69146700
H	-1.24394200	-1.27176300	-1.16930100
H	-1.43298500	-2.22107900	0.31089500
O	0.40283900	1.42513000	-0.36499800
O	1.16584100	-0.49346700	0.51863400
Mn	3.15637800	-0.14452200	-0.15258500

Molecule: Mn-PRO-013

N	1.89157300	-1.02844800	0.56703100
H	1.19202800	-1.74782600	0.35461600
H	2.36635800	-1.26949200	1.43561400
C	2.89106800	-0.87854200	-0.55326000
C	1.16527700	0.29144500	0.72210200
H	2.38346300	-1.17094500	-1.46965100
H	3.73123200	-1.54060700	-0.36870600
C	3.21308200	0.60369600	-0.53550200
C	1.85295400	1.25003600	-0.26880400
H	1.29210900	0.61755100	1.75084000
C	-0.31110000	0.09809200	0.42993100
H	3.65304800	0.92778900	-1.47640300
H	3.91268400	0.82726100	0.27163300
H	1.27449600	1.30434700	-1.19186400
H	1.92657500	2.25016500	0.14929400
O	-0.71263700	-1.03004200	0.03975200
O	-1.06321700	1.10066500	0.55448300
Mn	-2.94535400	-0.07009800	-0.27781100

Molecule: Mn-PRO-021

N	-2.17015500	0.50571600	0.60871400
H	-2.26138400	1.46502500	0.25119300
H	-2.47463900	0.48117600	1.58036300
C	-2.95276500	-0.48679700	-0.21378700
C	-0.70417000	0.14525500	0.51314700
H	-3.05625200	-0.06416800	-1.21046500
H	-3.93303700	-0.62170100	0.23270600
C	-2.06485100	-1.71744200	-0.21600000
C	-0.66163300	-1.12649000	-0.35049800
H	-0.34816000	-0.04783800	1.52211000
C	0.06659200	1.32882900	-0.07130500
H	-2.31848200	-2.39139600	-1.03170900
H	-2.16713400	-2.25685600	0.72721800
H	-0.45815100	-0.86465300	-1.38945600
H	0.11896300	-1.80199900	-0.00529500
O	-0.57005100	2.34578500	-0.39649900
O	1.32305000	1.18618800	-0.18967600
Mn	2.55865300	-0.58214100	0.07129600

Molecule: Mn-PRO-032

N	1.11056100	-0.11529100	1.22829500
H	0.17513800	-0.24136400	1.62363500
H	1.74060000	0.18464900	1.97016500
C	1.60340100	-1.39539400	0.58821000
C	1.03324400	0.95569600	0.15671800
H	0.72427100	-2.00245600	0.37418000
H	2.24625400	-1.91530100	1.29120700
C	2.27545000	-0.92063800	-0.68589000
C	1.35181400	0.20530900	-1.14350300
H	1.79560100	1.69368000	0.38466300
C	-0.34282000	1.61807800	0.13256500
H	2.36165400	-1.72351800	-1.41492700
H	3.27331100	-0.53453800	-0.46970700
H	0.43058100	-0.22086900	-1.55243300
H	1.79185700	0.87170700	-1.88028600
O	-1.32729300	0.92256400	0.56051300
O	-0.41631300	2.76418800	-0.32555400
Mn	-1.75563600	-1.10309100	-0.20371300

Molecule: Ni-PRO-01

N	1.83463500	-1.07075600	0.48381200
H	1.20852500	-1.77321300	0.08319700
H	2.12029100	-1.39763900	1.40703300
C	3.04772300	-0.85068800	-0.40036800
C	1.11606500	0.25423700	0.62364100
H	2.76721300	-1.16697200	-1.40143500
H	3.85994800	-1.46770400	-0.03025300
C	3.28363700	0.64648500	-0.32397500
C	1.86864100	1.22103700	-0.30445700
H	1.20311500	0.56552100	1.66217800
C	-0.33743400	0.11473300	0.29538300
H	3.86538600	0.99871600	-1.17285100
H	3.81336000	0.90199500	0.59502000
H	1.43174300	1.19548400	-1.30337500
H	1.81386100	2.23600300	0.07864500
O	-0.82459800	-0.97208600	-0.14430300
O	-1.11796900	1.10214600	0.44803300
Ni	-2.61632600	-0.06972200	-0.18128700

Molecule: Ni-PRO-02

N	-2.14288900	0.62874500	0.66122400
H	-2.19756900	1.58137700	0.28617800
H	-2.32081900	0.67223800	1.66406200
C	-3.13303800	-0.29760400	-0.01611300
C	-0.75261100	0.09986700	0.40968500
H	-3.41175100	0.17062900	-0.95623000
H	-4.00591000	-0.39686800	0.62073500
C	-2.34320800	-1.57733400	-0.23284600
C	-0.94460800	-1.06675400	-0.57220200
H	-0.35579400	-0.25329300	1.35902200
C	0.12805700	1.21345700	-0.12346200
H	-2.77642400	-2.17944100	-1.02850400
H	-2.32160700	-2.17029200	0.68277600
H	-0.90934400	-0.70005300	-1.59890800
H	-0.16170600	-1.81101400	-0.43918100
O	-0.33838200	2.30428300	-0.41079400
O	1.39739300	0.93852200	-0.28192700
Ni	2.40219700	-0.55309800	0.12617400

Molecule: Ni-PRO-032

N	1.31134200	-0.03098600	1.23146600
H	0.46591500	-0.17273600	1.78625700
H	2.04444500	0.30834000	1.85394000
C	1.74405700	-1.32652700	0.58078500
C	1.08702600	0.99191000	0.13047200
H	0.84320100	-1.91660700	0.42015300
H	2.40824400	-1.85173200	1.25992800
C	2.36843600	-0.87119500	-0.72188600
C	1.43903200	0.25423500	-1.17495400
H	1.76732300	1.81563700	0.31746900
C	-0.32959100	1.52844700	0.11549300
H	2.42297900	-1.68310300	-1.44368100
H	3.37576600	-0.49075200	-0.54513000
H	0.53837300	-0.16599500	-1.62866100
H	1.89426100	0.93950200	-1.88472000
O	-1.28935700	0.68987600	0.45964500
O	-0.55432000	2.67587200	-0.21127400
Ni	-1.71586600	-0.96260200	-0.15436600

Molecule: Zn-PRO-01

N	2.23180900	-1.02608900	0.41990800
H	1.73323800	-1.79230400	-0.04646200
H	2.55813800	-1.35680100	1.32699700
C	3.39072300	-0.51834800	-0.40747400
C	1.25132300	0.10980700	0.60967800
H	3.18864100	-0.79326800	-1.43960900
H	4.30066800	-1.00292400	-0.06791000
C	3.33999500	0.98569100	-0.20593600
C	1.83992800	1.27991100	-0.19261200
H	1.21578300	0.33970800	1.67122600
C	-0.12751100	-0.33009400	0.14375600
H	3.86631200	1.51116500	-1.00007600
H	3.79221700	1.25536100	0.75005500
H	1.44324200	1.27081400	-1.20905700
H	1.58876700	2.23043600	0.26998900
O	-0.27706100	-1.43094700	-0.39404000
O	-1.04179700	0.54486100	0.35760800
Zn	-2.89751900	0.11491100	-0.08625100

Molecule: Zn-PRO-02

N	-2.37024100	0.68907100	0.13073800
H	-2.24740500	1.52574100	-0.45073900
H	-3.01218100	0.91352100	0.88984700
C	-2.88503200	-0.49262700	-0.65193500
C	-1.01232900	0.31832300	0.68994400
H	-2.53617500	-0.37514600	-1.67524200
H	-3.97026700	-0.48734100	-0.62814200
C	-2.22401200	-1.67267900	0.03476100
C	-0.81130500	-1.15867200	0.31232000
H	-1.05959300	0.44634400	1.76926800
C	0.03363800	1.28820500	0.14990000
H	-2.23359800	-2.56024400	-0.59434300
H	-2.74110300	-1.90210300	0.96830500
H	-0.19981400	-1.22764800	-0.59099300
H	-0.30778000	-1.69671500	1.11080500
O	-0.32947200	2.26770300	-0.50355700
O	1.25507500	1.03021600	0.46729700
Zn	2.29630100	-0.51795200	-0.15445900

Molecule: Zn-PRO-32

N	1.26726300	0.10051400	1.22140000
H	0.40667900	-0.01017200	1.75999800
H	1.98084000	0.49563400	1.83259400
C	1.72303400	-1.23379600	0.67257900
C	1.03333900	1.04355200	0.04916300
H	0.84001700	-1.86978300	0.61032700
H	2.43635900	-1.67118000	1.36415000
C	2.27659500	-0.89015200	-0.69706100
C	1.33009900	0.20044900	-1.20132000
H	1.74829100	1.85297700	0.15053800
C	-0.38015700	1.61356500	0.08238100
H	2.29380300	-1.76261500	-1.34703000
H	3.29256100	-0.50273600	-0.60788900
H	0.41493400	-0.24780800	-1.59833500
H	1.76487800	0.81777300	-1.98310300
O	-1.32606500	0.81806900	0.45993200
O	-0.55427800	2.78442300	-0.24621700
Zn	-1.49679700	-1.03424400	-0.12917400

Molecule: Cd-THR-012

N	2.28117100	1.69160600	0.16154700
H	1.55738200	2.41415200	0.13396400
H	3.13897300	2.05482400	-0.25733900
H	2.46721100	1.46711800	1.14384800
C	1.80283000	0.46452200	-0.54773300
H	1.93307000	0.62953600	-1.61618900
C	2.61872800	-0.74832700	-0.11167800
C	0.30913400	0.29769600	-0.28857600
H	2.17337600	-1.61172200	-0.60964200
C	4.08603800	-0.65195600	-0.48500800
O	2.42685000	-0.84639100	1.30422200
O	-0.32554000	1.23466200	0.25510500
H	4.57703300	0.19328300	0.00107500
H	4.59837100	-1.56421800	-0.17599700
H	4.19475800	-0.54900700	-1.56567300
H	3.05156500	-1.49443800	1.65429500
O	-0.23257100	-0.77603500	-0.67047800
Cd	-2.32313000	-0.13438100	0.03433300

Molecule: Cd-THR-021

N	2.56384800	0.46455800	0.95084900
H	2.72252100	1.46283400	1.12483400
H	3.46257200	-0.01697300	0.89731200
H	2.03776000	0.07982900	1.73992800
C	1.77965100	0.34092300	-0.31846100
H	2.48031600	0.53306500	-1.13137600
C	1.24291200	-1.06589800	-0.51804500
C	0.78007000	1.52434800	-0.34394200
H	0.76530800	-1.07706600	-1.49785300
C	2.31650800	-2.13415400	-0.45378000
O	0.23479500	-1.32042700	0.49632800
O	1.20984200	2.60045100	0.08721700
H	2.75202600	-2.20468600	0.54459500
H	1.88381100	-3.10333200	-0.70404500
H	3.10884200	-1.91586200	-1.17040800
H	0.02742900	-2.26623600	0.50085200
O	-0.37580900	1.33704200	-0.84779700
Cd	-1.71777100	-0.15981900	0.10332500

Molecule: Cu-THR-02

N	-2.46248500	-0.94336900	-0.02151400
H	-2.39144300	-1.96605600	0.00955100
H	-3.24333800	-0.66308000	-0.61568500
H	-2.64350500	-0.61680300	0.93190300
C	-1.16060800	-0.39503600	-0.51918300
H	-1.18666800	-0.44444200	-1.60773200
C	-1.00900500	1.06071100	-0.09862700
C	-0.07137300	-1.38759400	-0.07077700
H	-0.11237000	1.44657700	-0.59332200
C	-2.18277300	1.92590800	-0.52611300
O	-0.83098700	1.05350100	1.32006100
O	-0.42671200	-2.49227900	0.33346100
H	-3.10738700	1.62836800	-0.02855300
H	-1.97725900	2.96490600	-0.26629800
H	-2.32673600	1.86621900	-1.60600300
H	-0.86211600	1.96600600	1.63570900
O	1.17257100	-1.09225300	-0.23352200
Cu	2.14868200	0.46366200	-0.06140600

Molecule: Cu-THR-012

N	1.60113900	1.72217500	0.02150600
H	0.88506600	2.43644900	-0.13170300
H	2.47759100	2.05464100	-0.38664100
H	1.73347100	1.61338800	1.03162700
C	1.19786800	0.41692900	-0.58167200
H	1.30329600	0.49907400	-1.66210900
C	2.08766600	-0.71404100	-0.06274600
C	-0.25813800	0.16540600	-0.27896800
H	1.68205700	-1.63656100	-0.48523200
C	3.54069000	-0.56705300	-0.47242700
O	1.91689600	-0.70637400	1.35622000
O	-0.93939300	0.97762700	0.41254400
H	3.98753200	0.34689300	-0.07709600
H	4.10788600	-1.41524800	-0.08688900
H	3.62853600	-0.56335500	-1.55965600
H	2.56378900	-1.30821200	1.74690800
O	-0.83553500	-0.85802200	-0.74714700
Cu	-2.55591200	-0.17915800	0.05731700

Molecule: Cu-THR-021

N	2.17795400	0.58372000	0.88914900
H	2.34975400	1.58453600	1.02608000
H	3.07756200	0.10876100	0.79303100
H	1.70970700	0.21881000	1.72357800
C	1.32583700	0.40288900	-0.32931100
H	1.96048100	0.64469400	-1.18148900
C	0.87008400	-1.03174200	-0.51412100
C	0.25228400	1.51641600	-0.28931700
H	0.39184900	-1.07647800	-1.49248700
C	1.98071500	-2.05567700	-0.43586000
O	-0.14565200	-1.31662600	0.49585600
O	0.65864500	2.65934400	-0.09801400
H	2.41872800	-2.10284400	0.56192800
H	1.58498800	-3.04059900	-0.68433700
H	2.76027100	-1.80711800	-1.15657900
H	-0.32298000	-2.27072100	0.50854900
O	-0.98125300	1.22182200	-0.50074000
Cu	-1.86218900	-0.33974900	0.13491500

Molecule: Fe-THR-01

N	1.52797000	1.70975100	0.13653800
H	0.82070200	2.43979400	0.01847400
H	2.41448200	2.06653800	-0.22844000
H	1.63991700	1.53214700	1.14023900
C	1.14259100	0.45057100	-0.56837200
H	1.24690200	0.61665800	-1.64057700
C	2.03470000	-0.71498200	-0.13546000
C	-0.30753400	0.17506400	-0.29015200
H	1.69195900	-1.58531400	-0.70097300
C	3.50469400	-0.46774500	-0.41867700
O	1.76629000	-0.88895900	1.25593800
O	-1.01599700	0.98655600	0.38063400
H	3.89176200	0.37060500	0.16306100
H	4.07475700	-1.35699500	-0.14795000
H	3.66172500	-0.26798600	-1.47938200
H	2.40926400	-1.51050600	1.62142100
O	-0.90218200	-0.85496700	-0.73801900
Fe	-2.67611000	-0.18737300	0.06099700

Molecule: Fe-THR-02

N	2.16244700	0.63931000	0.86142500
H	2.34374800	1.64067900	0.98151600
H	3.06196000	0.16269500	0.76219500
H	1.70512400	0.28941500	1.70910100
C	1.30146100	0.42927300	-0.34511100
H	1.91270500	0.69153600	-1.20890600
C	0.87891400	-1.01757400	-0.53003100
C	0.19183600	1.49949100	-0.28520300
H	0.44214900	-1.08942900	-1.52523200
C	1.98415800	-2.03374600	-0.36725800
O	-0.19234900	-1.29913300	0.43386700
O	0.50301900	2.65509800	-0.09481500
H	2.36737500	-2.05564800	0.65317000
H	1.60274000	-3.02401300	-0.61569500
H	2.79795200	-1.79652500	-1.05280500
H	-0.30095600	-2.25817900	0.55737600
O	-1.05275200	1.12282000	-0.47421000
Fe	-1.97198000	-0.38964000	0.15216100

Molecule: Hg-THR-01

N	3.15119400	1.61392300	0.01744600
H	2.61230100	2.47745800	-0.07820700
H	4.06099100	1.73235100	-0.43118300
H	3.29913100	1.44836200	1.01735500
C	2.40135100	0.46496700	-0.57666300
H	2.48751600	0.53577100	-1.65867200
C	2.99077800	-0.85897000	-0.09446200
C	0.92711200	0.61447800	-0.18955500
H	2.37965400	-1.64474300	-0.54337100
C	4.44157600	-1.06284000	-0.49149100
O	2.81830800	-0.85655900	1.32669800
O	0.55878200	1.56810100	0.48987600
H	5.09734100	-0.31051100	-0.04955100
H	4.77331600	-2.04287100	-0.14450700
H	4.54719500	-1.03117400	-1.57670700
H	3.32166700	-1.59295400	1.69668700
O	0.18339500	-0.32708700	-0.65590700
Hg	-1.84607800	-0.11113300	0.00592200

Molecule: Hg-THR-02

N	3.22018000	0.44931700	0.68230500
H	3.38946700	1.45097600	0.82414000
H	4.09508900	-0.01421400	0.43360700
H	2.88432500	0.05375400	1.56468800
C	2.19047100	0.30179500	-0.39333000
H	2.71080800	0.45195200	-1.34029700
C	1.59666000	-1.09719600	-0.41382500
C	1.22072000	1.50059700	-0.26642700
H	0.96820800	-1.14680800	-1.30382500
C	2.62811500	-2.20445600	-0.46893600
O	0.75370500	-1.22374400	0.75807100
O	1.68031300	2.54048400	0.20577600
H	3.25934900	-2.22374300	0.42057300
H	2.12035300	-3.16643700	-0.54786600
H	3.25949500	-2.07847400	-1.34960800
H	0.50385800	-2.15332500	0.85962400
O	0.03357300	1.39244200	-0.75034800
Hg	-1.39110900	-0.08746000	0.04012400

Molecule: Mn-THR-11

N	2.03120600	1.60479500	0.05387500
H	1.45250800	2.44832300	0.03177800
H	2.91593500	1.77586100	-0.42532000
H	2.22724500	1.39086700	1.03605700
C	1.27619200	0.47154900	-0.56462200
H	1.37572600	0.56073700	-1.64470800
C	1.85589700	-0.86144400	-0.10416500
C	-0.20670800	0.62372000	-0.19929400
H	1.23217500	-1.63627700	-0.55421500
C	3.30125700	-1.07754800	-0.51417500
O	1.69826600	-0.87665300	1.32071800
O	-0.56713700	1.62742600	0.43474700
H	3.96563900	-0.32757200	-0.08097300
H	3.63224500	-2.05947500	-0.17110500
H	3.39577500	-1.04655600	-1.60059100
H	2.20339400	-1.61940000	1.67481600
O	-0.95153100	-0.32058500	-0.60825400
Mn	-3.01662800	-0.36397000	0.01892000

Molecule: Mn-THR-012

N	2.02811400	1.60090300	0.08330700
H	1.37839000	2.38436300	0.21317900
H	2.83275200	1.89280200	-0.47149700
H	2.35513600	1.31406000	1.01067600
C	1.28002300	0.47515100	-0.55832600
H	1.38444000	0.57909300	-1.63657800
C	1.85634700	-0.86348800	-0.11333500
C	-0.20692200	0.62882700	-0.20528900
H	1.23576500	-1.63433100	-0.57404900
C	3.30409400	-1.06791800	-0.51969200
O	1.69489000	-0.89477500	1.31103600
O	-0.57258000	1.64910300	0.40014300
H	3.96008900	-0.31440000	-0.07969900
H	3.64149600	-2.04936000	-0.18139100
H	3.40202800	-1.02931300	-1.60553600
H	2.20934800	-1.63376300	1.65949300
O	-0.94778500	-0.32457400	-0.59649300
Mn	-3.01574800	-0.36755700	0.02178500

Molecule: Mn-THR-21

N	-2.26672100	0.64827600	-0.74782700
H	-2.25629600	1.65955700	-0.92911800
H	-3.21704800	0.33715800	-0.54392900
H	-1.94179500	0.17006800	-1.59173400
C	-1.33068300	0.39912000	0.39317800
H	-1.89079400	0.60194400	1.30628100
C	-0.86889700	-1.04756500	0.43997300
C	-0.22090300	1.47612200	0.31597000
H	-0.28496400	-1.15171000	1.35547100
C	-2.00642400	-2.05005000	0.44599600
O	0.00158600	-1.26266900	-0.69560900
O	-0.52632300	2.54493000	-0.24015200
H	-2.59021700	-2.00684100	-0.47549400
H	-1.60347100	-3.05908300	0.54427800
H	-2.66828200	-1.86193700	1.29219900
H	0.10976700	-2.21441200	-0.82706400
O	0.88897700	1.20610800	0.86357400
Mn	2.23430700	-0.38341600	-0.15557300

Molecule: Ni-THR-01

N	1.60662800	1.71989900	0.14807300
H	0.92465200	2.47213600	0.02494100
H	2.51246800	2.04867900	-0.19368600
H	1.69130800	1.52644400	1.15099900
C	1.18603300	0.48354200	-0.57616600
H	1.32977300	0.65182900	-1.64246100
C	2.01672600	-0.71995300	-0.12498400
C	-0.28206600	0.24354100	-0.33099800
H	1.63114000	-1.57664700	-0.68258900
C	3.50004200	-0.56424200	-0.39629000
O	1.73217900	-0.86940100	1.26756000
O	-0.98315500	1.04991900	0.35062400
H	3.92955400	0.26611600	0.16724100
H	4.01455300	-1.47818000	-0.09677900
H	3.67815800	-0.40317200	-1.46023300
H	2.34297300	-1.51934500	1.63835700
O	-0.84360000	-0.78693800	-0.80977600
Ni	-2.53817000	-0.20832600	0.07718100

Molecule: Ni-THR-02

N	-2.07510400	0.55744700	-0.97025700
H	-2.26876300	1.55024400	-1.13209900
H	-2.96813300	0.06246100	-0.91873500
H	-1.55237400	0.19525600	-1.77299100
C	-1.28992500	0.40993100	0.29682600
H	-1.97231700	0.65449600	1.10949800
C	-0.82919500	-1.01717900	0.52792200
C	-0.22954600	1.53804700	0.29692400
H	-0.33881500	-1.03083300	1.50182000
C	-1.93527900	-2.04815400	0.49396400
O	0.18020000	-1.33926300	-0.48522400
O	-0.65176900	2.68253300	0.17880700
H	-2.39209500	-2.12581900	-0.49344800
H	-1.52980200	-3.02334400	0.76427400
H	-2.70139100	-1.78130600	1.22196200
H	0.31448400	-2.30080000	-0.51815500
O	1.01644900	1.24603000	0.44503300
Ni	1.83141300	-0.36117000	-0.13475200

Molecule: Zn-THR-01

N	1.64247100	1.71234700	0.14694300
H	0.91093200	2.41607400	0.02809100
H	2.53013400	2.09968500	-0.17928500
H	1.72458200	1.51058800	1.14780700
C	1.29698000	0.45935400	-0.59380700
H	1.48109000	0.63823100	-1.65120500
C	2.16743100	-0.69900300	-0.10806500
C	-0.18108300	0.15132100	-0.40830300
H	1.85013600	-1.57523900	-0.67764700
C	3.65325500	-0.47218600	-0.31438200
O	1.83690200	-0.86779700	1.27456400
O	-0.88168700	0.94439100	0.29614100
H	4.01658800	0.38444200	0.25619600
H	4.20063000	-1.35536200	0.01808800
H	3.87069100	-0.31201300	-1.37120800
H	2.46096300	-1.49334500	1.66425400
O	-0.65932400	-0.86761400	-0.94537900
Zn	-2.61765500	-0.15360800	0.10936800

Molecule: Zn-THR-02

N	-1.12195000	0.41683900	-1.64371900
H	-0.26724700	-0.10680900	-1.85110200
H	-1.09671200	1.29534300	-2.16665000
H	-1.91867900	-0.12858200	-1.98065400
C	-1.22600900	0.68424500	-0.17195500
H	-2.15933200	1.21412800	-0.01199800
C	-1.27978000	-0.64142800	0.59907800
C	-0.07451000	1.62886200	0.24541800
H	-1.05286500	-0.42131300	1.64264300
C	-2.62309800	-1.33204000	0.49706700
O	-0.22999100	-1.49752300	0.06199400
O	1.12901100	1.24487100	0.00248700
H	-2.90086800	-1.54356700	-0.53669300
H	-2.59734800	-2.27302000	1.04762300
H	-3.38866300	-0.69599700	0.94266600
H	-0.23794500	-2.34300400	0.53312700
O	-0.37040900	2.69825900	0.77460300
Zn	1.68216000	-0.65059100	0.00522500

Molecule: Cd-VAL-01

N	2.31665900	1.94383400	-0.21928400
H	2.01893300	2.12386500	0.74239600
H	1.88080800	2.65114300	-0.81469400
H	3.33151500	2.05604600	-0.27664000
C	1.92029800	0.56359700	-0.64972800
H	2.14943900	0.49164800	-1.70970700
C	2.74266500	-0.48671900	0.12841200
C	0.42379900	0.39842100	-0.44462400
H	3.78036200	-0.15054500	0.03991400
C	2.37968400	-0.55950900	1.61095300
C	2.64150900	-1.85882300	-0.53153100
O	-0.15897300	1.11697600	0.41200300
H	1.36054600	-0.92636900	1.75046700
H	3.05437000	-1.25308200	2.11383400
H	2.46673000	0.40417100	2.11622300
H	2.89269700	-1.81071700	-1.59229300
H	3.33683000	-2.54524800	-0.04668900
H	1.63789000	-2.27414300	-0.43475500
O	-0.16964000	-0.49486400	-1.10506000
Cd	-2.12803200	-0.11859000	0.09396900

Molecule: Cd-VAL-02

N	2.40601500	-0.95515600	1.40078300
H	3.05720700	-1.34242900	0.71121800
H	2.01988100	-1.73845300	1.93088100
H	2.91339800	-0.34436700	2.04421500
C	1.31785500	-0.20904600	0.69044300
H	0.50638600	-0.08465100	1.40361700
C	1.83411900	1.18045100	0.26915900
C	0.85912600	-1.09155900	-0.47746500
H	2.21878400	1.62644800	1.19207700
C	2.97185600	1.12017100	-0.74834400
C	0.69693700	2.06374400	-0.23194800
O	1.68201500	-1.86625100	-0.98001300
H	2.63523800	0.69033000	-1.69352100
H	3.33212000	2.13014300	-0.94740500
H	3.82213000	0.53591400	-0.39153100
H	-0.12729400	2.10039500	0.48696000
H	1.05914500	3.08220100	-0.37682700
H	0.30753500	1.70879000	-1.18741100
O	-0.34507600	-0.97035600	-0.89573800
Cd	-1.98669800	0.05483500	0.10452500

Molecule: Cd-VAL-031

N	0.89223900	0.19323000	2.01888400
H	0.00751600	-0.27691900	1.82412700
H	0.66850700	1.08011300	2.47386700
H	1.42400300	-0.38153300	2.67676100
C	1.69150000	0.42570900	0.76929600
H	2.55630800	1.00362900	1.08691700
C	2.18121900	-0.91578800	0.18960700
C	0.90038000	1.29070200	-0.21744700
H	2.58460200	-1.46462900	1.04606700
C	1.07248400	-1.75462300	-0.44283100
C	3.32717200	-0.68930600	-0.79427600
O	-0.37570300	1.36792900	-0.08610200
H	0.66066600	-1.25627400	-1.32441300
H	1.47406200	-2.71661600	-0.76329800
H	0.25284900	-1.96179800	0.25132400
H	4.11735600	-0.08173200	-0.34987000
H	3.75628600	-1.64972000	-1.08259000
H	2.97987500	-0.18919000	-1.69875600
O	1.54612900	1.90448000	-1.06936800
Cd	-1.89849300	-0.20369500	-0.12613900

Molecule: Cu-VAL-02

N	-1.97689700	-1.06254700	-1.30347500
H	-2.59933000	-1.41477600	-0.57108000
H	-1.59404400	-1.87122300	-1.79782000
H	-2.52136100	-0.50538100	-1.96609200
C	-0.88032600	-0.24280400	-0.69837900
H	-0.12946300	-0.10598300	-1.47458800
C	-1.42644800	1.13706500	-0.27546300
C	-0.29165200	-1.05541500	0.45596800
H	-1.85524900	1.55545000	-1.19122800
C	-2.52378200	1.05782300	0.78407300
C	-0.30050700	2.06047500	0.17763800
O	-1.00344600	-1.84780100	1.06612600
H	-2.14429800	0.63606200	1.71647700
H	-2.89026800	2.06307500	0.99527100
H	-3.38039200	0.46285200	0.46227100
H	0.51749300	2.07991900	-0.54992700
H	-0.67734300	3.07793900	0.28422300
H	0.10409900	1.75015600	1.14272100
O	0.94930800	-0.85784900	0.76691200
Cu	2.20613200	0.12455400	-0.18114600

Molecule: Cu-VAL-011

N	1.56597900	2.02371300	-0.30870200
H	1.43997800	2.20397200	0.69052600
H	0.93019900	2.64315500	-0.81667900
H	2.52757000	2.26748800	-0.55817300
C	1.30798900	0.58618800	-0.64338200
H	1.44292700	0.49227500	-1.71963300
C	2.31649700	-0.33043700	0.08276000
C	-0.12993300	0.27226400	-0.32476200
H	3.28571200	0.15677300	-0.05771500
C	2.04016800	-0.45531500	1.58020200
C	2.38224400	-1.69965900	-0.58825300
O	-0.83784800	1.05747900	0.37303200
H	1.09792700	-0.97699800	1.76373500
H	2.83734700	-1.03342800	2.04811700
H	1.99732500	0.51184300	2.08529600
H	2.57929400	-1.60891700	-1.65760900
H	3.18764900	-2.28229900	-0.13978200
H	1.45243100	-2.25335600	-0.45489700
O	-0.66448300	-0.79289700	-0.75504700
Cu	-2.38701200	-0.22901300	0.11705000

Molecule: Cu-VAL-031

N	0.87343600	0.95416200	1.86608500
H	0.05306100	0.37887000	2.06826000
H	0.61125400	1.93020800	2.02034400
H	1.61315300	0.70886900	2.52826100
C	1.35262200	0.74607000	0.46033100
H	2.17021300	1.44650900	0.30978200
C	1.87671000	-0.69446300	0.28938600
C	0.23998900	1.09517500	-0.52289200
H	2.55915400	-0.84621400	1.13157600
C	0.78119600	-1.75620500	0.36141900
C	2.68939500	-0.82639300	-0.99566000
O	-0.96995100	1.05703600	-0.03960200
H	0.09109600	-1.67231200	-0.48425000
H	1.23233800	-2.74792900	0.31533900
H	0.20132100	-1.70618400	1.28525600
H	3.46790800	-0.06407700	-1.05354400
H	3.16781000	-1.80605100	-1.02313100
H	2.05584500	-0.73721400	-1.87860000
O	0.51383400	1.38743300	-1.67608400
Cu	-2.11475000	-0.43118800	-0.07282400

Molecule: Fe-VAL-01

N	1.49695700	2.01969900	-0.33864500
H	1.38810600	2.21692900	0.66014200
H	0.85332500	2.62977500	-0.84978300
H	2.45455800	2.26278600	-0.60638400
C	1.24773500	0.57675600	-0.65067500
H	1.36904900	0.46379700	-1.72704000
C	2.26644400	-0.33057100	0.07958200
C	-0.17696200	0.25723100	-0.31594500
H	3.22897600	0.16228900	-0.08342100
C	2.00952400	-0.43233900	1.58158100
C	2.32606700	-1.70641700	-0.57623300
O	-0.88157800	0.99374200	0.43899200
H	1.07899000	-0.96692200	1.78697900
H	2.82270900	-0.98855700	2.04791400
H	1.95740700	0.54263100	2.07016300
H	2.52181100	-1.62779300	-1.64651900
H	3.13089800	-2.28472200	-0.12158400
H	1.39571300	-2.25814400	-0.43400000
O	-0.76157600	-0.77201000	-0.78494300
Fe	-2.52199400	-0.24045300	0.12814600

Molecule: Fe-VAL-02

N	2.01286100	-1.05966800	1.26839600
H	2.65932100	-1.34946800	0.52954600
H	1.65590700	-1.90807600	1.71504700
H	2.53224600	-0.52143200	1.96630700
C	0.89145700	-0.23986400	0.71079600
H	0.17125300	-0.10378500	1.51463800
C	1.41742000	1.13751600	0.24953100
C	0.26057100	-1.05614700	-0.40399600
H	1.97806600	1.51431200	1.11033100
C	2.35845100	1.05454700	-0.94996000
C	0.26957300	2.10362800	-0.01790400
O	0.87274100	-1.89213000	-1.02818200
H	1.82874800	0.72349500	-1.84523700
H	2.76796800	2.04483300	-1.15114000
H	3.20075000	0.38216900	-0.78135200
H	-0.41476800	2.15523500	0.83262600
H	0.66446200	3.10470300	-0.19221900
H	-0.30036500	1.81199700	-0.90310200
O	-1.00761200	-0.78505400	-0.67628900
Fe	-2.34382600	0.11473400	0.17041300

Molecule: Fe-VAL-031

N	0.34214700	-0.11451800	2.01560200
H	-0.41522500	-0.74366000	1.73445000
H	-0.07398800	0.66649600	2.52955100
H	0.95835300	-0.62524600	2.65354500
C	1.13562300	0.37191800	0.84176100
H	1.92297000	1.00087100	1.25029300
C	1.77670400	-0.81713400	0.09547800
C	0.29693600	1.26179000	-0.06141000
H	2.32282600	-1.36064600	0.87255600
C	0.76283000	-1.76828700	-0.54030100
C	2.79278900	-0.31728800	-0.92799800
O	-1.01302500	1.01852400	-0.08874100
H	0.16354000	-1.26172800	-1.30417500
H	1.29380200	-2.58516100	-1.03004000
H	0.08709300	-2.22042300	0.18767900
H	3.49035000	0.39229500	-0.48065000
H	3.36452700	-1.16248700	-1.31133400
H	2.30415700	0.16917200	-1.77332800
O	0.78759800	2.13992000	-0.72546700
Fe	-2.17689700	-0.35082400	-0.28343400

Molecule: Hg-VAL-01

N	3.16376100	1.78347800	-0.39167400
H	3.08106700	2.03867600	0.59520000
H	2.73755900	2.53652000	-0.93585400
H	4.15447000	1.72557000	-0.63749800
C	2.48292300	0.47660300	-0.66276600
H	2.49290600	0.34205800	-1.74141600
C	3.26049200	-0.67182200	0.01026000
C	1.03558000	0.61089000	-0.18621300
H	4.30915800	-0.48332100	-0.24057700
C	3.11981100	-0.67990100	1.53147900
C	2.87909800	-2.02116000	-0.59219900
O	0.72140300	1.50543600	0.59426300
H	2.09177900	-0.89650600	1.83049800
H	3.75952700	-1.45662000	1.95183200
H	3.41444500	0.26763000	1.98690600
H	2.97489800	-2.01184500	-1.67925100
H	3.54178900	-2.79301300	-0.19853000
H	1.85476600	-2.29681200	-0.33968200
O	0.25083100	-0.28636900	-0.67574100
Hg	-1.76255000	-0.06871100	0.02748200

Molecule: Hg-VAL-02

N	2.80005500	-0.96426000	1.41264300
H	3.47534700	-1.33858300	0.73967300
H	2.40431100	-1.75582700	1.92382700
H	3.28668500	-0.35897400	2.07730300
C	1.72643800	-0.20953700	0.69127600
H	0.90839300	-0.07826500	1.39570700
C	2.25521800	1.17594500	0.27092000
C	1.27861700	-1.08713300	-0.47912700
H	2.62321500	1.62298100	1.19985800
C	3.41168200	1.10694500	-0.72497900
C	1.12917400	2.05800600	-0.25652600
O	2.09184300	-1.85355500	-0.99316700
H	3.09047300	0.67645900	-1.67519800
H	3.77887900	2.11518900	-0.91949500
H	4.25335700	0.52087600	-0.35141800
H	0.28350000	2.08540800	0.43704800
H	1.49266900	3.07826000	-0.38484500
H	0.76916600	1.70745100	-1.22487000
O	0.06903900	-0.98659800	-0.92890100
Hg	-1.52575300	0.03663400	0.06576300

Molecule: Hg-VAL-01

N	1.24026200	0.10368100	2.02027900
H	0.47323200	-0.54617000	1.82988400
H	0.83134300	0.95701600	2.40738500
H	1.84196200	-0.31930300	2.73010300
C	2.04352100	0.39734700	0.78706300
H	2.89897000	0.97188200	1.13604100
C	2.55464500	-0.91915900	0.16722600
C	1.28513300	1.30879400	-0.17693800
H	2.92772600	-1.50010100	1.01619100
C	1.46635100	-1.72882400	-0.53434900
C	3.73300300	-0.65862400	-0.76783000
O	-0.00476000	1.45519100	-0.06421400
H	1.11829100	-1.21615200	-1.43386100
H	1.86334000	-2.69926200	-0.83450800
H	0.60205800	-1.91951800	0.11016500
H	4.50493900	-0.06292700	-0.27772100
H	4.17541100	-1.60895400	-1.06905900
H	3.41559600	-0.13345300	-1.66921300
O	1.94305900	1.93992400	-0.99506800
Hg	-1.44171300	-0.12758700	-0.08080200

Molecule: Mn-VAL-11

N	1.74729200	1.94632600	-0.31142400
H	1.51345400	2.14710700	0.66373900
H	1.23884000	2.61786800	-0.89007200
H	2.74951500	2.08824400	-0.45167100
C	1.35566500	0.54067100	-0.66053500
H	1.50276000	0.43713300	-1.73274100
C	2.26294000	-0.45549000	0.08929000
C	-0.12240100	0.37594500	-0.32642800
H	3.27996200	-0.07126400	-0.03724700
C	1.95768500	-0.53561800	1.58458900
C	2.20799900	-1.83511900	-0.55944800
O	-0.63257900	1.14823100	0.51728200
H	0.96943700	-0.96615500	1.76090900
H	2.69317500	-1.17688700	2.07166500
H	1.99570600	0.43816600	2.07655200
H	2.43722400	-1.78113700	-1.62507400
H	2.94116900	-2.48950200	-0.08570400
H	1.22417000	-2.29122300	-0.44273900
O	-0.75687700	-0.55392300	-0.90527800
Mn	-2.78528500	-0.23493700	0.12846100

Molecule: Mn-VAL-21

N	1.91167000	-0.94160600	1.38476200
H	2.54334700	-1.33349400	0.67945900
H	1.54218600	-1.72157200	1.93085200
H	2.43363600	-0.32336600	2.00886100
C	0.80238100	-0.21079400	0.69072400
H	-0.00299300	-0.10236700	1.41308700
C	1.28788200	1.18820300	0.26663400
C	0.33882700	-1.09483300	-0.47619200
H	1.67055100	1.64417800	1.18564300
C	2.41835200	1.14932700	-0.75986000
C	0.13009600	2.04906100	-0.22875800
O	1.16769200	-1.87073300	-0.98006400
H	2.08083400	0.71509800	-1.70276300
H	2.76038000	2.16532900	-0.96083500
H	3.28142200	0.57864800	-0.41180500
H	-0.68821600	2.07907400	0.49746400
H	0.47416400	3.07243200	-0.38292300
H	-0.26300500	1.68223700	-1.17796800
O	-0.86363500	-0.95907200	-0.86438200
Mn	-2.46046700	0.09150800	0.20111500

Molecule: Mn-VAL-31

N	0.30717100	0.10489800	1.99341200
H	-0.55079500	-0.37894800	1.72424400
H	0.03024300	0.95869700	2.48069700
H	0.82601200	-0.48872100	2.64450800
C	1.14813100	0.42700900	0.79165800
H	1.97773900	1.02309400	1.16518800
C	1.71115000	-0.87510800	0.18875600
C	0.34126400	1.30460100	-0.17679100
H	2.17060400	-1.39869300	1.03327300
C	0.64529100	-1.78747800	-0.41458500
C	2.81360500	-0.57847300	-0.82396900
O	-0.93060600	1.30282200	-0.06281500
H	0.18907100	-1.32827900	-1.29642300
H	1.09403500	-2.73156200	-0.72388300
H	-0.15315600	-2.02383800	0.29568000
H	3.57518500	0.08022200	-0.40316600
H	3.29564200	-1.51103900	-1.12094100
H	2.41158400	-0.10565400	-1.72024300
O	0.98389000	1.97854600	-0.99151600
Mn	-2.29597100	-0.40095300	-0.27954300

Molecule: Ni-VAL-01

N	1.53586900	2.03222700	-0.27381000
H	1.37532100	2.19645400	0.72350300
H	0.92097400	2.66269200	-0.79432200
H	2.50655400	2.27597900	-0.48546700
C	1.28675600	0.60140400	-0.63925100
H	1.45335400	0.52086300	-1.71196200
C	2.27052300	-0.32917600	0.10773800
C	-0.15450200	0.27568900	-0.36191700
H	3.24177500	0.16463700	0.01333900
C	1.93633100	-0.48472800	1.59088900
C	2.36722500	-1.68471800	-0.58661900
O	-0.87568300	1.04081500	0.35229900
H	0.99647200	-1.02499000	1.72627000
H	2.72304300	-1.05952900	2.07991000
H	1.85824000	0.47265300	2.11008200
H	2.59773500	-1.57274100	-1.64716400
H	3.16203200	-2.27002300	-0.12297100
H	1.43772200	-2.24674900	-0.49258800
O	-0.67734000	-0.78528300	-0.82004400
Ni	-2.38707600	-0.23785400	0.12839100

Molecule: Ni-VAL-02

N	1.94701000	-1.13662000	1.29001800
H	2.56276300	-1.49972600	0.55781000
H	1.52407800	-1.93814600	1.76380400
H	2.50866500	-0.61827300	1.96994500
C	0.89588700	-0.25283600	0.69698700
H	0.15811300	-0.08041400	1.47745800
C	1.51525700	1.09854000	0.28423200
C	0.25436500	-1.02105900	-0.45501400
H	1.98840500	1.47079300	1.19784400
C	2.58505000	0.96913500	-0.79759500
C	0.43648600	2.09740100	-0.12475900
O	0.89796300	-1.84764700	-1.08083700
H	2.15758800	0.61059400	-1.73571300
H	3.02624200	1.94911300	-0.98288900
H	3.39745400	0.29835500	-0.51159600
H	-0.36090600	2.15311000	0.62238900
H	0.87541600	3.09034700	-0.22561700
H	-0.00853100	1.82835600	-1.08413700
O	-0.98829500	-0.74133400	-0.75355800
Ni	-2.31635600	0.14489200	0.17759400

Molecule: Ni-VAL-031

N	0.42323800	-0.06010700	2.02928200
H	-0.30091600	-0.74660400	1.80518100
H	-0.02652400	0.72979600	2.49803800
H	1.07240800	-0.49727300	2.68746600
C	1.17540900	0.39363700	0.81189300
H	1.95785600	1.04863800	1.18714400
C	1.83715600	-0.81146900	0.11346500
C	0.30146100	1.25018600	-0.10424100
H	2.33694200	-1.36098800	0.91738700
C	0.84496600	-1.74852000	-0.56887400
C	2.90895900	-0.34483600	-0.86765000
O	-0.99298100	1.08876600	-0.10165000
H	0.33407200	-1.24709600	-1.39711000
H	1.37048400	-2.61146200	-0.97830300
H	0.08410900	-2.13315400	0.11693100
H	3.62670700	0.32086500	-0.38612500
H	3.44996800	-1.21092600	-1.25048000
H	2.47017500	0.17991400	-1.71726400
O	0.84601500	2.09930400	-0.79265800
Ni	-2.16321100	-0.35676800	-0.24431900

Molecule: Zn-VAL-02

N	-2.08890400	-0.96178700	-1.30891200
H	-2.69664000	-1.31764200	-0.56521600
H	-1.75829500	-1.76636600	-1.84463000
H	-2.63047700	-0.35581800	-1.92883400
C	-0.93620000	-0.21933300	-0.70049300
H	-0.18882800	-0.10993900	-1.48260200
C	-1.40892600	1.17872700	-0.25096900
C	-0.38406000	-1.10451200	0.42391900
H	-1.86081000	1.61749400	-1.14641700
C	-2.46275300	1.14288200	0.85471800
C	-0.23364100	2.06317600	0.14959200
O	-1.15942000	-1.86898900	1.00491600
H	-2.05536700	0.72274200	1.77595100
H	-2.79329700	2.16024500	1.06778200
H	-3.34634500	0.56559400	0.57743700
H	0.54075200	2.07227800	-0.62335700
H	-0.57419900	3.08879200	0.29389700
H	0.21693400	1.72499300	1.08466800
O	0.86173900	-1.00576900	0.71787300
Zn	2.22346100	0.09875200	-0.15630700

Molecule: Zn-VAL-011

N	1.79957700	1.97002300	-0.28172700
H	1.62322400	2.15203100	0.70895200
H	1.25514000	2.64906000	-0.81790100
H	2.79220400	2.12316100	-0.47180500
C	1.41220800	0.56678100	-0.64165800
H	1.55042600	0.47601700	-1.71690700
C	2.33212500	-0.43621400	0.08656300
C	-0.06005900	0.38925800	-0.31511100
H	3.34091500	-0.02722600	-0.02456700
C	2.02014000	-0.55826700	1.57758200
C	2.31115100	-1.79732700	-0.60209700
O	-0.64299400	1.22426200	0.41095400
H	1.03253100	-0.99632600	1.73914000
H	2.75460800	-1.21138300	2.04995800
H	2.05595900	0.40255300	2.09508500
H	2.53997000	-1.70636400	-1.66528400
H	3.06071900	-2.44554800	-0.14613500
H	1.33962400	-2.28156100	-0.50011400
O	-0.65496000	-0.63200300	-0.78562100
Zn	-2.45507100	-0.22160100	0.10291100

Molecule: Zn-VAL-031

N	0.41887700	0.06911300	2.01657200
H	-0.38798400	-0.51079500	1.77771200
H	0.06296000	0.90361400	2.48690000
H	1.00753600	-0.44774600	2.67386400
C	1.21056700	0.44056600	0.79737500
H	2.01826200	1.07603800	1.15333500
C	1.82391300	-0.82333100	0.16353400
C	0.34829500	1.28558500	-0.14289300
H	2.34023200	-1.32547300	0.98772000
C	0.79158900	-1.78940800	-0.41444000
C	2.87081700	-0.45026200	-0.88330100
O	-0.93654500	1.20626300	-0.01924600
H	0.24531900	-1.33037500	-1.24414200
H	1.29449100	-2.67738400	-0.79874000
H	0.06739100	-2.12900700	0.33107600
H	3.59959100	0.25745400	-0.48472900
H	3.40426900	-1.34727900	-1.20011900
H	2.40891400	-0.00376700	-1.76453700
O	0.91792800	2.02993700	-0.93569400
Zn	-2.03717600	-0.36058600	-0.25054900

Molecule: Cd-ALA-01

N	-2.92715100	-1.24203600	-0.14883100
H	-2.57226000	-1.62096700	0.73327800
H	-2.61122700	-1.86012300	-0.89916400
H	-3.94892200	-1.25662200	-0.12408700
C	-2.42886100	0.15602700	-0.35310500
H	-2.74397000	0.45858600	-1.34890500
C	-3.02306100	1.07038400	0.70648100
C	-0.90857000	0.16064700	-0.29355100
H	-2.70070900	0.76713300	1.70379500
H	-2.69567400	2.09201300	0.52512200
H	-4.11174300	1.03838000	0.65797900
O	-0.33229300	-0.84478400	0.22563600
O	-0.29457000	1.16083000	-0.71907800
Cd	1.77192600	-0.03697600	0.07046600

Molecule: Cd-ALA-022

N	3.07510600	-0.59132900	-0.65718200
H	3.65393800	-0.23500500	0.10739800
H	3.25717900	-0.00695800	-1.47534300
H	3.35668500	-1.55124200	-0.86682500
C	1.63028400	-0.54144900	-0.26714500
H	1.05858400	-0.82671400	-1.14841600
C	1.38512500	-1.51080500	0.87731000
C	1.28490800	0.89977400	0.11567200
H	1.94880700	-1.21371700	1.76299000
H	0.32527800	-1.52450400	1.12587900
H	1.67767400	-2.52061400	0.58807900
O	2.20285900	1.70814600	0.29138100
O	0.04515400	1.19189600	0.25608000
Cd	-1.67895600	-0.08890400	-0.08808700

Molecule: Cd-ALA-031

N	-1.36836400	-1.55730600	-0.53514000
H	-0.78081200	-1.69209400	0.29217900
H	-0.75475900	-1.49637000	-1.35012300
H	-1.97020700	-2.37719000	-0.64213600
C	-2.19658800	-0.31584000	-0.37903500
H	-2.73177800	-0.18026600	-1.31438000
C	-3.15759800	-0.49944700	0.78510300
C	-1.29589300	0.89689400	-0.12598400
H	-2.60752500	-0.63379000	1.71786700
H	-3.78769000	0.38404700	0.87376700
H	-3.79673400	-1.36721500	0.61894500
O	-0.24260700	0.69631500	0.58653200
O	-1.68507300	1.99403800	-0.52475400
Cd	1.69437400	-0.07809300	0.02864900

Molecule: Cu-ALA-012

N	-2.41739200	-1.20442800	-0.02121400
H	-2.23872100	-1.42179400	0.96338200
H	-2.00939600	-1.95307000	-0.58615600
H	-3.42943600	-1.20075600	-0.16954100
C	-1.84597700	0.13079200	-0.38326400
H	-2.09359300	0.30469700	-1.43009800
C	-2.44312000	1.21242300	0.50384200
C	-0.34574200	0.06869300	-0.25809500
H	-2.17909700	1.04178800	1.54841000
H	-2.06253800	2.18317000	0.19452200
H	-3.52796100	1.21340500	0.40192200
O	0.23340800	-0.99354800	0.12907000
O	0.36303500	1.08376900	-0.50533000
Cu	1.98275800	-0.03205700	0.10556000

Molecule: Cu-ALA-022

N	-2.69884000	-0.41400700	0.50736600
H	-3.15036700	-0.09481800	-0.35390400
H	-2.92662600	0.26286200	1.23868900
H	-3.08877800	-1.32309600	0.76411900
C	-1.21804900	-0.51500500	0.30952600
H	-0.79057200	-0.77566600	1.27649900
C	-0.91511200	-1.58952100	-0.72333900
C	-0.71532000	0.86800800	-0.10021900
H	-1.28478100	-1.29910600	-1.70756300
H	0.15971800	-1.75526700	-0.78664400
H	-1.38220300	-2.52969300	-0.42918400
O	0.56247000	1.04808900	-0.17139300
O	-1.51547700	1.76445400	-0.35124300
Cu	1.93346300	-0.16097900	0.12799000

Molecule: Cu-ALA-032

N	-1.05921300	-1.56803200	-0.48442300
H	-0.56590700	-1.78618200	0.38514100
H	-0.37617300	-1.58288100	-1.24514500
H	-1.75157700	-2.30137200	-0.65580100
C	-1.73292200	-0.23199500	-0.38479800
H	-2.19928200	-0.04545700	-1.34843100
C	-2.76551700	-0.25996600	0.73147200
C	-0.70144600	0.86338100	-0.12719900
H	-2.28749900	-0.45435000	1.69275600
H	-3.27209800	0.70242000	0.77742900
H	-3.50681900	-1.03556100	0.53745900
O	0.31921000	0.51046700	0.59807000
O	-0.91174500	1.99968700	-0.52190000
Cu	1.97632500	-0.16655700	0.04556400

Molecule: Fe-ALA-01

N	-2.46801000	-1.12604800	-0.02387800
H	-2.29380600	-1.37994400	0.95340100
H	-2.16742500	-1.90337400	-0.61775600
H	-3.47764200	-1.00202700	-0.14196000
C	-1.77536000	0.14645500	-0.38243100
H	-2.00008700	0.35407400	-1.42866300
C	-2.25073100	1.28715600	0.51106500
C	-0.29231500	-0.02128000	-0.25617800
H	-2.02329200	1.07414200	1.55620800
H	-1.74761200	2.20545200	0.21673700
H	-3.32484300	1.41828200	0.39003400
O	0.27723400	-1.05354400	0.20252100
O	0.47434500	0.95282400	-0.55932100
Fe	2.08494500	-0.02125000	0.10995400

Molecule: Fe-ALA-023

N	2.67927400	-0.35705900	-0.48900300
H	3.11626400	-0.04507100	0.38319800
H	2.89946000	0.33524000	-1.20970700
H	3.09512900	-1.25160000	-0.75852100
C	1.20109100	-0.50383000	-0.31936700
H	0.79446100	-0.75555000	-1.29797000
C	0.89789700	-1.60178400	0.69186700
C	0.65724100	0.85668500	0.09213200
H	1.24265500	-1.31889500	1.68695200
H	-0.17269300	-1.80341500	0.73190900
H	1.39817900	-2.52065000	0.38581700
O	1.37033900	1.78017700	0.40929600
O	-0.66453200	0.98647200	0.07460000
Fe	-2.05046900	-0.18385600	-0.12144700

Molecule: Fe-ALA-031

N	-0.90405800	-1.56484500	-0.57768200
H	-0.31919900	-1.79469900	0.23042300
H	-0.30133000	-1.49340200	-1.40167000
H	-1.55722000	-2.33881100	-0.72708000
C	-1.67146400	-0.30104000	-0.34785200
H	-2.27232300	-0.13333500	-1.23862100
C	-2.54937000	-0.45233400	0.88534300
C	-0.75079400	0.89959100	-0.19715200
H	-1.94088400	-0.64492400	1.76995900
H	-3.12003000	0.46140900	1.03735700
H	-3.24391100	-1.27937600	0.73823600
O	0.48245200	0.65790100	0.22810000
O	-1.14979500	2.02283000	-0.38175100
Fe	2.08660800	-0.15946500	0.10855200

Molecule: Hg-ALA-01

N	-3.79000000	-0.93109500	-0.14533900
H	-3.65428700	-1.27968600	0.80723000
H	-3.58160000	-1.69854400	-0.78745900
H	-4.77022700	-0.66382400	-0.25963000
C	-2.89925100	0.24596700	-0.39546100
H	-3.01992900	0.51757800	-1.44113800
C	-3.29949500	1.39135600	0.52028700
C	-1.45749500	-0.18957600	-0.13963100
H	-3.15497900	1.11672600	1.56640800
H	-2.68991300	2.26427700	0.29763600
H	-4.34746000	1.64670600	0.36074700
O	-1.22336800	-1.23218300	0.46793300
O	-0.58425400	0.64803100	-0.58108500
Hg	1.40183500	0.00751500	0.01834500

Molecule: Hg-ALA-02

N	3.44748400	-0.59377100	-0.67224000
H	4.04173800	-0.23827000	0.08115100
H	3.61474100	-0.01162700	-1.49563700
H	3.72379700	-1.55454100	-0.88571000
C	2.01015000	-0.54610900	-0.25924100
H	1.42264700	-0.83398300	-1.12926300
C	1.77731900	-1.50865100	0.89490900
C	1.67881700	0.89405100	0.12000000
H	2.34794500	-1.20408800	1.77336700
H	0.71815000	-1.52132200	1.14817800
H	2.07103100	-2.51816900	0.60669700
O	2.58586800	1.70808300	0.27794300
O	0.43815500	1.22195400	0.29250000
Hg	-1.23827900	-0.05547100	-0.05613300

Molecule: Hg-ALA-031

N	-1.64865400	-1.58445300	-0.63080500
H	-0.98751400	-1.75393800	0.13392900
H	-1.10827100	-1.43746400	-1.48642900
H	-2.22084700	-2.42428800	-0.74855100
C	-2.52665800	-0.41029000	-0.33048200
H	-3.19250600	-0.31437100	-1.18347600
C	-3.31449900	-0.69677500	0.94133500
C	-1.78215900	0.92193400	-0.18439400
H	-2.64245500	-0.80007400	1.79460700
H	-4.00650100	0.12070800	1.13261000
H	-3.88679200	-1.61781800	0.82484800
O	-0.59675200	1.00529000	0.33680600
O	-2.43394800	1.92850400	-0.44926100
Hg	1.24463700	-0.03801400	0.02861200

Molecule: Mn-ALA-012

N	-2.47874500	-1.17725000	-0.09215800
H	-2.33268400	-1.44112700	0.88524900
H	-2.06231800	-1.90675900	-0.67359500
H	-3.48420200	-1.14503400	-0.27405100
C	-1.85508200	0.15586100	-0.37305600
H	-2.06485100	0.37998400	-1.41740100
C	-2.46490200	1.20807800	0.53672800
C	-0.34639100	0.04677500	-0.19968400
H	-2.25227000	0.98261000	1.58321400
H	-2.04262000	2.18088600	0.29442200
H	-3.54469200	1.25227700	0.39268700
O	0.14397100	-1.01357000	0.26616900
O	0.34547900	1.04490900	-0.53719200
Mn	2.36870000	-0.03108300	0.08955300

Molecule: Mn-ALA-021

N	2.59896500	-0.54665900	-0.58895400
H	3.11338700	-0.17340900	0.21259800
H	2.81666200	0.04705000	-1.39119200
H	2.92376900	-1.49596500	-0.78178700
C	1.12954600	-0.53441400	-0.29289700
H	0.62328900	-0.81053300	-1.21579700
C	0.83055000	-1.53648100	0.80885200
C	0.73260200	0.89491400	0.09634500
H	1.33062100	-1.25329900	1.73638300
H	-0.24241500	-1.57513500	0.98996600
H	1.16138100	-2.53357100	0.51590000
O	1.63215900	1.71056900	0.34988400
O	-0.51328600	1.14491900	0.14732000
Mn	-2.20106500	-0.16666200	-0.14379300

Molecule: Mn-ALA-32

N	-0.64014500	-1.53917200	-0.57477200
H	-0.04909800	-1.66771300	0.25131100
H	-0.02504200	-1.34013500	-1.36637900
H	-1.13567300	-2.41429400	-0.75489400
C	-1.60986300	-0.41922100	-0.35330200
H	-2.20519500	-0.35740300	-1.26219100
C	-2.49089000	-0.75950500	0.83647400
C	-0.88982500	0.92720100	-0.18258700
H	-1.89995300	-0.81252000	1.75274500
H	-3.25541400	0.00477500	0.95400900
H	-2.98372100	-1.71967000	0.67670500
O	0.37866200	0.95917400	-0.09103900
O	-1.62645500	1.92344100	-0.14779500
Mn	2.23843700	-0.09882500	0.15517000

Molecule: Ni-ALA-01

N	-2.40673100	-1.18998200	-0.09322500
H	-2.26594000	-1.44849400	0.88770300
H	-1.99381600	-1.92289300	-0.67529700
H	-3.41324300	-1.16120200	-0.27493600
C	-1.80441000	0.14781500	-0.38313400
H	-2.00911200	0.36782200	-1.42999800
C	-2.41543300	1.20644500	0.52253800
C	-0.31246500	0.06352600	-0.21022300
H	-2.19632000	0.99043200	1.56893300
H	-2.00360600	2.17993500	0.26577100
H	-3.49473700	1.23224700	0.37602900
O	0.24321800	-0.97402700	0.26532600
O	0.42396000	1.04629200	-0.52423900
Ni	2.00286800	-0.03545700	0.08680700

Molecule: Ni-ALA-21

N	2.67550800	-0.35032900	-0.62658900
H	3.19991600	-0.02783900	0.19108300
H	2.81076600	0.33726400	-1.37115500
H	3.06681000	-1.24420100	-0.93298000
C	1.22797900	-0.50779100	-0.28945600
H	0.72447800	-0.82623500	-1.20124200
C	1.07299500	-1.55101600	0.80682000
C	0.68794400	0.85379300	0.12489300
H	1.58376600	-1.23358000	1.71658400
H	0.01799900	-1.70238200	1.03256900
H	1.48886200	-2.50119100	0.47206700
O	1.44211300	1.77461600	0.39073400
O	-0.61297000	0.98109400	0.19434500
Ni	-2.00670700	-0.18446900	-0.14482000

Molecule: Ni-ALA-34

N	-0.79090200	-1.63154100	-0.40598300
H	-0.32569800	-1.81666800	0.48852600
H	-0.08850600	-1.62470200	-1.15192500
H	-1.42220500	-2.41732000	-0.58846300
C	-1.57845000	-0.36303500	-0.36753500
H	-2.03073500	-0.26442700	-1.35408100
C	-2.65724900	-0.47799300	0.69846700
C	-0.74596000	0.89828300	-0.18362000
H	-2.21362800	-0.60080100	1.68714000
H	-3.27392900	0.41693300	0.69183900
H	-3.28957700	-1.33855500	0.47876900
O	0.56651500	0.92046800	-0.06103000
O	-1.32182200	1.96939900	-0.17923500
Ni	1.93260700	-0.15700500	0.12958300

Molecule: Zn-ALA-01

N	-2.95284200	-0.79182600	-0.15499900
H	-2.93904800	-1.08472300	0.82520500
H	-2.76990600	-1.61895000	-0.72647700
H	-3.88651400	-0.43853800	-0.37550900
C	-1.92452000	0.26760100	-0.39783800
H	-1.96593000	0.50347300	-1.45943800
C	-2.24237900	1.49358900	0.44195800
C	-0.54647500	-0.30428900	-0.07254400
H	-2.20594400	1.25136200	1.50560800
H	-1.51207500	2.27236400	0.23428200
H	-3.23412100	1.87398700	0.19656100
O	-0.43813500	-1.40625300	0.46732900
O	0.40923600	0.48590700	-0.40596200
Zn	2.25649600	0.04683900	0.01881200

Molecule: Zn-ALA-02

N	-2.69225700	0.48496800	-0.56246400
H	-3.18990100	0.14326100	0.26366900
H	-2.91107100	-0.14820900	-1.33391500
H	-3.03836800	1.41873100	-0.79221500
C	-1.21910300	0.52160100	-0.29356000
H	-0.73673900	0.79535200	-1.23028200
C	-0.93291700	1.55494200	0.78477400
C	-0.77466900	-0.88756000	0.10623500
H	-1.41863000	1.28045900	1.72231600
H	0.13993200	1.62596300	0.95795800
H	-1.29107900	2.53543500	0.46980200
O	-1.62802200	-1.74501700	0.34142000
O	0.48981700	-1.10448700	0.18613000
Zn	1.93191400	0.15387900	-0.13083900

Molecule: Zn-ALA-03

N	-0.85847100	-1.57393600	-0.55585500
H	-0.26394700	-1.73211000	0.26177200
H	-0.25250200	-1.47272900	-1.37276100
H	-1.44652500	-2.40007000	-0.69013900
C	-1.71641900	-0.36007800	-0.35964700
H	-2.30094000	-0.24961200	-1.26897000
C	-2.62143200	-0.57893800	0.84358000
C	-0.88172900	0.90831500	-0.16833100
H	-2.02846300	-0.70881800	1.75054600
H	-3.26960400	0.28617400	0.96822100
H	-3.24322000	-1.46189900	0.69318500
O	0.30032100	0.81524900	0.32398400
O	-1.44227500	1.97780000	-0.40867100
Zn	1.97558700	-0.11345200	0.07776700

Molecule: Cd-ASP-01

N	-1.39007700	2.52559900	-0.09583400
H	-1.04251200	2.59623700	-1.05494900
H	-0.70411200	2.97219500	0.51554200
H	-2.27329500	3.03624200	-0.03171000
C	-1.59575000	1.09106100	0.29054900
H	-1.97964300	1.09673300	1.30846500
C	-2.60710500	0.44278500	-0.64604700
C	-0.25630000	0.35963800	0.28592200
H	-3.44555000	1.13292500	-0.77788900
H	-2.16039900	0.27043800	-1.62477100
C	-3.20483900	-0.86306500	-0.09690100
O	0.76765100	1.04857400	-0.05549400
O	-3.50616200	-0.89462000	1.12435100
O	-3.39779600	-1.78812700	-0.92798000
O	-0.23443600	-0.83735500	0.59643600
Cd	2.46429100	-0.31654700	-0.05340600

Molecule: Cd-ASN-011

N	1.48373500	2.58715000	0.02957000
H	1.09684300	2.67623600	0.97273600
H	0.85221800	3.07107900	-0.61286200
H	2.39596400	3.04942900	0.00145400
C	1.61593200	1.14437700	-0.34508500
H	1.98504800	1.11709900	-1.36692900
C	2.60799100	0.48118200	0.61637900
C	0.23996900	0.48638500	-0.29941300
H	3.55093200	1.02787200	0.55923800
H	2.22550000	0.54266400	1.63394000
C	2.85327100	-0.98231400	0.29675200
O	-0.64209300	1.04789300	0.42608600
O	2.38541800	-1.87784000	1.00562900
N	3.61146600	-1.22672300	-0.77818200
H	3.79235400	-2.17965900	-1.05561700
H	3.97934600	-0.47622100	-1.34101500
O	0.05078400	-0.55530800	-0.94916100
Cd	-2.37084300	-0.29265100	0.02035700

Molecule: Cd-ASN-022

N	3.31927900	-0.44939600	-0.24583800
H	3.29907600	-0.83828600	-1.19189100
H	3.73090100	-1.15988800	0.36249200
H	3.91503200	0.38128800	-0.24331900
C	1.93440500	-0.11511100	0.21445800
H	2.04036500	0.29748000	1.21623300
C	1.33278100	0.93176200	-0.74337500
C	1.13902000	-1.42955200	0.31378200
H	2.14870500	1.50580600	-1.18323200
H	0.79029300	0.44127900	-1.54985300
C	0.39899900	1.90860300	-0.06350700
O	1.68673300	-2.47039800	-0.06310000
O	-0.84282400	1.75126600	-0.03616000
N	0.95741200	2.97323700	0.49244300
H	0.38233000	3.64818400	0.97633500
H	1.95259200	3.12689000	0.43810200
O	-0.02465100	-1.34855400	0.83055000
Cd	-1.74128000	-0.33963200	-0.09853300

Molecule: Cd-ASN-031

N	4.07892800	0.50937700	-0.36609900
H	4.13427200	-0.13608000	-1.16105100
H	4.79988600	0.22818100	0.30118900
H	4.27450300	1.46379300	-0.67487100
C	2.72198000	0.38471400	0.24815800
H	2.77903600	0.80858400	1.24674600
C	1.72990200	1.16887300	-0.61980300
C	2.34991700	-1.11176300	0.33254900
H	2.03176900	2.21733000	-0.64516600
H	1.73678200	0.77288100	-1.63382600
C	0.31951100	1.07496800	-0.09695400
O	2.98882800	-1.89528500	-0.40858500
O	-0.50632000	0.37593500	-0.73167900
N	0.01766300	1.75122300	0.99586800
H	-0.91096300	1.68480100	1.39043700
H	0.71728900	2.29642600	1.47691500
O	1.41620700	-1.38773200	1.11875700
Cd	-2.54492300	-0.22925400	-0.07750800

Molecule: Cd-ASN-041

N	-3.04846000	-0.34722300	-1.09962400
H	-3.70419300	-0.38032900	-0.31297000
H	-3.08504400	-1.25671600	-1.56560900
H	-3.34852400	0.37315000	-1.76038600
C	-1.67021900	-0.07371600	-0.59129200
H	-0.99288100	-0.21088900	-1.43039200
C	-1.63715300	1.36687900	-0.06713200
C	-1.36131200	-1.10733400	0.50956500
H	-2.03937600	2.02319100	-0.84594300
H	-2.27229100	1.45671300	0.81245600
C	-0.28004200	1.92579000	0.29409500
O	-2.31818300	-1.62899100	1.09228400
O	-0.07698700	2.50370700	1.34397100
N	0.73947300	1.75143700	-0.63513500
H	1.52443700	2.38202100	-0.50843100
H	0.45294500	1.64195400	-1.60274200
O	-0.13630700	-1.35476000	0.76914800
Cd	1.65775000	-0.51433200	-0.14911100

Molecule: Cu-ASN-01

N	1.87997200	2.37907600	-0.13948500
H	1.68310800	2.66151400	0.82500200
H	1.43104300	3.05757100	-0.75921400
H	2.89028300	2.41566000	-0.29561500
C	1.35911100	1.00231600	-0.39256000
H	1.51700300	0.78963500	-1.44691600
C	2.12734800	0.01480600	0.49476300
C	-0.13378400	0.99222400	-0.08178900
H	3.19168600	0.12179100	0.27773400
H	1.95520600	0.26135400	1.54159400
C	1.71071600	-1.43051300	0.28069400
O	-0.64025100	1.86114600	0.62115500
O	1.01211400	-2.02418700	1.10709600
N	2.15724400	-2.00763400	-0.84092500
H	1.90860800	-2.96436100	-1.04303500
H	2.74710200	-1.50908300	-1.48806400
O	-0.74874800	-0.01692300	-0.59685100
Cu	-2.51551100	-0.32646400	-0.05539500

Molecule: Cu-ASN-02

N	2.47547600	1.44846100	0.42491800
H	2.94896800	1.50269700	-0.48106800
H	3.12145000	1.00266000	1.07990300
H	2.27706700	2.39788600	0.74934300
C	1.21557100	0.65468300	0.31596600
H	0.78486900	0.62471000	1.31576300
C	0.25032800	1.34397600	-0.67113900
C	1.59369700	-0.77854500	-0.07628700
H	0.61306300	2.34310700	-0.90261700
H	0.20129100	0.78825300	-1.60854400
C	-1.15343600	1.43671100	-0.13526600
O	2.77621600	-1.05893000	-0.26113600
O	-1.81058200	0.41300500	0.19856300
N	-1.70023600	2.62872900	-0.03150000
H	-2.64985800	2.72009700	0.30332500
H	-1.18317500	3.45863900	-0.27907100
O	0.63682300	-1.62790400	-0.18092700
Cu	-1.23437800	-1.41823100	0.08335800

Molecule: Cu-ASN-04

N	-2.83164400	0.35018900	-0.88046400
H	-3.37648000	0.56216800	-0.03975800
H	-3.19418900	-0.52425100	-1.26862700
H	-2.97948700	1.09515700	-1.56619100
C	-1.38398300	0.21930400	-0.55262000
H	-0.88172100	-0.04769700	-1.48079500
C	-0.88868300	1.56027200	-0.00506500
C	-1.20941100	-0.91562800	0.46159700
H	-1.08833800	2.33633900	-0.75081700
H	-1.42446000	1.81691500	0.90741700
C	0.58371400	1.63122700	0.29583200
O	-2.19276000	-1.41223100	0.99736000
O	1.03832000	2.14269100	1.28877200
N	1.44906200	1.05212500	-0.67070700
H	2.39739700	1.41621500	-0.58813100
H	1.10802400	1.11537000	-1.62921000
O	0.00368200	-1.26838500	0.72648300
Cu	1.57633000	-0.97427800	-0.27672600

Molecule: Cu-ASN-031

N	-3.44863500	-0.69647900	-0.39507900
H	-3.48023800	-0.29959800	-1.33726300
H	-4.18170500	-0.23673900	0.14842500
H	-3.65323600	-1.69703600	-0.44646600
C	-2.11442500	-0.43925500	0.22605200
H	-2.15456100	-0.85021200	1.23233200
C	-1.05301500	-1.16170700	-0.61168400
C	-1.86891300	1.08168800	0.31185900
H	-1.28509800	-2.22784100	-0.64221600
H	-1.05428100	-0.76985100	-1.62741900
C	0.32870800	-0.99115700	-0.04646800
O	-2.70606100	1.83059600	-0.24091800
O	1.14585900	-0.28517300	-0.70605900
N	0.63633200	-1.60812200	1.06990700
H	1.55220600	-1.49251800	1.48372600
H	-0.05678700	-2.15359200	1.56178700
O	-0.83332300	1.41120300	0.93413500
Cu	2.80668600	0.38859200	-0.14732800

Molecule: Fe-ASN-01

N	0.72963300	2.61487100	-0.03036200
H	0.46705300	2.74884000	0.95143500
H	0.00625300	3.04072900	-0.61711600
H	1.61068900	3.10987800	-0.19580800
C	0.90018700	1.16646300	-0.34922600
H	1.16397700	1.09491100	-1.40314500
C	2.01350400	0.58070600	0.52944900
C	-0.41746800	0.46891100	-0.16612100
H	2.91886400	1.15144200	0.31941800
H	1.74937900	0.70144500	1.57858700
C	2.25683500	-0.89706200	0.26911300
O	-1.37811700	0.98917600	0.47916700
O	1.81943800	-1.75583900	1.03829500
N	2.96378400	-1.18644400	-0.82732800
H	3.14133500	-2.15089600	-1.06587300
H	3.31488900	-0.46077100	-1.43217700
O	-0.63135600	-0.67155100	-0.67854700
Fe	-2.58555500	-0.60165300	-0.02085000

Molecule: Fe-ASN-02

N	-2.41348500	1.51720700	-0.44322300
H	-2.88076500	1.59105700	0.46528400
H	-3.07676600	1.09644100	-1.09827900
H	-2.18554100	2.46030900	-0.76908600
C	-1.17597800	0.68950600	-0.33812900
H	-0.76645700	0.61641000	-1.34487300
C	-0.17496800	1.39045800	0.60726200
C	-1.59380200	-0.71344200	0.09903800
H	-0.47724800	2.42388200	0.76129200
H	-0.16873700	0.90562500	1.58634400
C	1.23739100	1.35778300	0.09952200
O	-2.76068500	-0.97867500	0.30982700
O	1.76876400	0.28601500	-0.33928600
N	1.94940300	2.45150300	0.12949500
H	2.92272200	2.42891600	-0.14980300
H	1.55157200	3.31999600	0.45946800
O	-0.63991600	-1.60256100	0.21544700
Fe	1.21648700	-1.56183300	-0.07724300

Molecule: Fe-ASN-04

N	-2.91544500	0.42379100	-0.64308800
H	-3.36269000	0.53550700	0.27217700
H	-3.31998700	-0.40806500	-1.08232700
H	-3.14105700	1.23936700	-1.21852300
C	-1.44018000	0.27117100	-0.49744700
H	-1.04899800	0.08050700	-1.49769300
C	-0.86213300	1.55328500	0.10489600
C	-1.18870300	-0.96728600	0.34771100
H	-1.16918400	2.39335400	-0.52660000
H	-1.27435000	1.71793300	1.09965200
C	0.63775100	1.66301600	0.22979400
O	-2.07855200	-1.68653800	0.73526500
O	1.17139400	2.23010900	1.14857000
N	1.43963000	1.07392000	-0.79303100
H	2.37571900	1.47834300	-0.81018900
H	1.01353600	1.06672700	-1.72007100
O	0.07698200	-1.17946600	0.64643800
Fe	1.69302800	-1.10083100	-0.22367300

Molecule: Fe-ASN-031

N	-3.34780400	-0.68274400	-0.51755400
H	-3.24674500	-0.69655400	-1.53550200
H	-3.94326900	0.12124000	-0.27723600
H	-3.78644400	-1.55569600	-0.22068100
C	-2.03572700	-0.45205600	0.15390400
H	-2.14072500	-0.83119400	1.16993700
C	-0.92638200	-1.18906000	-0.59097600
C	-1.79143600	1.07157000	0.25298200
H	-1.13675500	-2.26096400	-0.60684300
H	-0.85710600	-0.82893100	-1.61560400
C	0.40777900	-1.00154300	0.06049200
O	-2.79443100	1.80892200	0.13156300
O	1.35106500	-0.48022000	-0.64772300
N	0.63817000	-1.44859700	1.25943000
H	1.54960900	-1.33004600	1.68659000
H	-0.10248000	-1.87702100	1.80065300
O	-0.60719700	1.40514000	0.48159400
Fe	2.88886300	0.45132100	-0.17601300

Molecule: Hg-ASN-01

N	2.88917100	2.37535600	-0.14530500
H	2.74089800	2.65164100	0.82935000
H	2.40272000	3.05338500	-0.73674400
H	3.89001700	2.41935500	-0.35305400
C	2.36657100	0.99561600	-0.38204000
H	2.49920300	0.78689200	-1.44044900
C	3.15858200	0.01024200	0.48237600
C	0.87223400	0.98410600	-0.05099700
H	4.21843300	0.13033200	0.24885900
H	3.00336400	0.24282400	1.53475100
C	2.76402500	-1.43996500	0.26039900
O	0.36725600	1.89354600	0.59753200
O	2.17103800	-2.08099800	1.13251600
N	3.12031200	-1.97141700	-0.91494400
H	2.86806100	-2.92476800	-1.12768000
H	3.59931200	-1.42604100	-1.61402600
O	0.28278900	-0.06271400	-0.51279600
Hg	-1.81031900	-0.11324800	-0.01894600

Molecule: Hg-ASN-02

N	-3.34635600	-0.54124200	-0.69763700
H	-3.87339400	-0.88227200	0.11062100
H	-3.43458100	-1.24235700	-1.43652200
H	-3.76593500	0.33240200	-1.02477100
C	-1.91301500	-0.34005300	-0.33535600
H	-1.39620100	-0.07741800	-1.25669500
C	-1.78489400	0.79577900	0.70750400
C	-1.34641200	-1.68310600	0.16068500
H	-2.77282400	1.11206600	1.03499900
H	-1.25300100	0.43033200	1.58663800
C	-0.99692300	1.98005300	0.19619300
O	-2.13933700	-2.59369400	0.41154200
O	0.20867700	1.89276300	-0.14537300
N	-1.62179700	3.14368000	0.13857200
H	-1.12345800	3.96666800	-0.17055200
H	-2.59132200	3.22591400	0.40289800
O	-0.07335700	-1.80247300	0.28544400
Hg	1.34084200	-0.11964000	-0.05150300

Molecule: Hg-ASN-04

N	-3.33267800	-0.49294600	-1.12631900
H	-3.99957400	-0.56794100	-0.35187100
H	-3.30531200	-1.40205400	-1.59411500
H	-3.66678200	0.20898300	-1.79060600
C	-1.98530500	-0.13002400	-0.59392000
H	-1.28314100	-0.21573700	-1.41994000
C	-2.06775300	1.30662800	-0.06115100
C	-1.63614100	-1.14546400	0.50827100
H	-2.51613000	1.93136600	-0.84008300
H	-2.71393000	1.33926000	0.81447500
C	-0.76309000	1.96913300	0.31183600
O	-2.56333700	-1.73620200	1.06536100
O	-0.60286000	2.53842600	1.37267700
N	0.26349100	1.90478600	-0.62642800
H	0.99786700	2.59211900	-0.49047800
H	-0.00964300	1.77287300	-1.59475200
O	-0.40360800	-1.32675300	0.82171500
Hg	1.31566400	-0.29183900	-0.09414500

Molecule: Hg-ASN-031

N	4.59493900	0.60138700	-0.36443200
H	4.65528100	0.08645700	-1.24718800
H	5.31580000	0.22402900	0.25340800
H	4.79277200	1.59060200	-0.53138700
C	3.24261500	0.40064800	0.23932400
H	3.28017500	0.81328800	1.24411000
C	2.21888700	1.15448100	-0.61849400
C	2.93583400	-1.11017100	0.31300300
H	2.48856800	2.21142400	-0.65437900
H	2.21840000	0.75466200	-1.63092100
C	0.82269900	1.03843100	-0.06694200
O	3.68306000	-1.87679200	-0.33693200
O	0.00941200	0.31112700	-0.70586500
N	0.51913200	1.71449000	1.01569900
H	-0.40534600	1.64270300	1.42213700
H	1.21536400	2.27869600	1.48115500
O	1.94133000	-1.41262500	1.01033300
Hg	-1.99687700	-0.13608800	-0.04796800

Molecule: Mn-ASN-11

N	1.80211300	2.39294500	-0.06203800
H	1.52942300	2.65378200	0.88940500
H	1.38350300	3.07466200	-0.69804900
H	2.81939800	2.45238600	-0.14498000
C	1.31975800	1.01232300	-0.37474700
H	1.54546700	0.83034400	-1.42261500
C	2.05964600	0.02340600	0.53335700
C	-0.19325100	0.95543700	-0.15367100
H	3.13295700	0.14831400	0.37653600
H	1.82864500	0.24703800	1.57413400
C	1.68539400	-1.42474800	0.27514000
O	-0.74070000	1.84714100	0.51189700
O	0.99982600	-2.06386400	1.07900900
N	2.16344800	-1.96016500	-0.85452100
H	1.93697600	-2.91483400	-1.08914600
H	2.73234600	-1.42322700	-1.48967900
O	-0.75583600	-0.06339800	-0.66567700
Mn	-2.79693000	-0.37021800	-0.02648000

Molecule: Mn-ASN-21

N	3.06058900	0.03490900	-0.00352000
H	3.16646700	-0.37955600	-0.93318800
H	3.51703100	-0.59180800	0.66248000
H	3.53558700	0.94000500	0.01878900
C	1.60867500	0.17862500	0.32870300
H	1.55224600	0.53257500	1.35497100
C	1.00099600	1.21194900	-0.63589400
C	0.95368400	-1.20882300	0.22068500
H	1.66659600	2.07579800	-0.67076900
H	0.93235500	0.78474600	-1.63575500
C	-0.37604100	1.67627900	-0.22466600
O	1.54437700	-2.08608400	-0.41924800
O	-1.40954600	1.11150600	-0.64870500
N	-0.44193800	2.71742500	0.58926700
H	-1.34236700	3.04837800	0.90628100
H	0.39218500	3.18825400	0.90553300
O	-0.16650600	-1.33098600	0.81695900
Mn	-2.02484600	-0.86273600	-0.03334400

Molecule: Mn-ASN-031

N	-3.43284300	-0.56974000	-0.19732900
H	-3.55535200	0.03056700	-1.01979700
H	-4.10793500	-0.26341700	0.50584700
H	-3.63300600	-1.54249700	-0.43856900
C	-2.03638000	-0.39171000	0.30764700
H	-2.00721500	-0.77577600	1.32333500
C	-1.09579100	-1.19228500	-0.60282300
C	-1.70172100	1.11613200	0.30095100
H	-1.38508000	-2.24366800	-0.56786500
H	-1.18393700	-0.83244200	-1.62698100
C	0.34441800	-1.05115200	-0.18248400
O	-2.36309400	1.83172100	-0.48897500
O	1.09010700	-0.24601600	-0.78006700
N	0.76095900	-1.80809400	0.82155700
H	1.71090200	-1.72373700	1.15570400
H	0.13346700	-2.44706300	1.28499100
O	-0.77755300	1.46872900	1.06683000
Mn	3.04290000	0.44486000	-0.09233300

Molecule: Mn-ASN-43

N	-2.59284600	-0.01944300	-1.19471500
H	-3.26615300	-0.01901300	-0.42101200
H	-2.71768000	-0.89229700	-1.71170600
H	-2.79458400	0.76196900	-1.82236800
C	-1.21247100	0.06798000	-0.62719100
H	-0.51523000	-0.18486900	-1.42321400
C	-0.99020800	1.50529800	-0.14283300
C	-1.11360800	-0.95531500	0.51978100
H	-1.16367000	2.18051700	-0.98120000
H	-1.70556100	1.73189900	0.64736600
C	0.40045600	1.71985800	0.41722900
O	-2.15373100	-1.21096100	1.14635600
O	0.65234900	1.51857800	1.60843000
N	1.32635300	2.13963600	-0.45708300
H	2.27600300	2.28292500	-0.14711400
H	1.09545100	2.30900000	-1.42288400
O	0.03175600	-1.44625600	0.75548000
Mn	1.87635400	-1.11717200	-0.40957300

Molecule: Ni-ASN-01

N	-0.88832500	2.63208700	0.02210100
H	-0.56947000	2.75499800	-0.94385400
H	-0.22764500	3.11323600	0.63844700
H	-1.80420600	3.07921000	0.11783600
C	-0.98549400	1.18257500	0.36775200
H	-1.28199100	1.11660900	1.41193400
C	-2.03040700	0.51966800	-0.54141900
C	0.37477400	0.54987900	0.21149400
H	-2.98390500	1.01925000	-0.36512200
H	-1.74157900	0.65623600	-1.58231700
C	-2.16856100	-0.97196900	-0.28597500
O	1.26299800	1.06399800	-0.52793700
O	-1.65147900	-1.79581700	-1.04441700
N	-2.87983700	-1.31601600	0.79249200
H	-2.97928400	-2.29060200	1.03439200
H	-3.28378900	-0.61811900	1.39679700
O	0.63040000	-0.53849000	0.81223500
Ni	2.43470700	-0.55577700	0.00569900

Molecule: Ni-ASN-02

N	-2.27957700	1.66900800	-0.46048900
H	-2.73751700	1.81895100	0.44238600
H	-2.97755700	1.27369700	-1.09482300
H	-1.97320700	2.57286900	-0.82885600
C	-1.12016100	0.74102700	-0.32109500
H	-0.68927900	0.64222000	-1.31681400
C	-0.09263800	1.34272500	0.66195300
C	-1.65451600	-0.63394300	0.09555100
H	-0.35864100	2.37458300	0.88158200
H	-0.10256600	0.79674300	1.60709500
C	1.31346200	1.29018400	0.13684200
O	-2.85490400	-0.78557100	0.28916500
O	1.84759300	0.20275700	-0.22652300
N	2.00138200	2.40529700	0.07103400
H	2.96199700	2.38806100	-0.24604500
H	1.58644800	3.28550400	0.33805400
O	-0.78628600	-1.57948300	0.21328100
Ni	1.06819900	-1.52908400	-0.09654600

Molecule: Ni-ASN-041

N	-2.83936500	0.42684600	-0.83144600
H	-3.35852700	0.57354000	0.03923000
H	-3.22957100	-0.40234800	-1.28672800
H	-2.98617400	1.23341100	-1.44419000
C	-1.38630900	0.23807400	-0.54732200
H	-0.91798500	-0.00077800	-1.49952700
C	-0.85255400	1.54729500	0.04082700
C	-1.23388200	-0.95177100	0.40646100
H	-1.08268700	2.35461000	-0.66264800
H	-1.35137900	1.77035300	0.98288900
C	0.62833900	1.63442900	0.28179700
O	-2.20187800	-1.35884600	1.02957200
O	1.11654800	2.20573600	1.22153600
N	1.47387200	0.99303800	-0.67825500
H	2.41802300	1.37866200	-0.63392200
H	1.11413500	1.03752100	-1.63295300
O	-0.05105800	-1.47653200	0.49587000
Ni	1.61107700	-0.98768500	-0.22716600

Molecule: Zn-ASN-011

N	1.10774500	2.61547400	0.02270200
H	0.70139100	2.71685000	0.95711600
H	0.53887300	3.16099700	-0.62978400
H	2.05271800	3.00800500	0.03152600
C	1.14159000	1.17530300	-0.37454100
H	1.49402300	1.13101600	-1.40102100
C	2.09883300	0.43480100	0.57032200
C	-0.26241200	0.60361300	-0.29336700
H	3.09224700	0.86859800	0.45029800
H	1.76929400	0.57744800	1.59868700
C	2.14140600	-1.05935000	0.30321200
O	-1.08590100	1.09645200	0.52662600
O	1.47271500	-1.84559900	0.98042500
N	2.93754500	-1.45311200	-0.69617500
H	2.98124700	-2.43013900	-0.94465300
H	3.47199900	-0.78689500	-1.23088300
O	-0.55060500	-0.38674400	-1.01254600
Zn	-2.46083300	-0.47405000	0.02310800

Molecule: Zn-ASN-021

N	2.34801100	1.58451700	0.49215300
H	2.85988000	1.67572000	-0.38931300
H	2.99542800	1.19236200	1.17879500
H	2.05995600	2.51506200	0.80409900
C	1.16356900	0.69143600	0.32213300
H	0.70605200	0.60130900	1.30598500
C	0.17664100	1.33422600	-0.68011100
C	1.66635600	-0.70342300	-0.09594800
H	0.52440100	2.32812200	-0.95341700
H	0.13167800	0.74334500	-1.59610700
C	-1.22957400	1.41512700	-0.14045100
O	2.87076300	-0.84928300	-0.31636400
O	-1.86181800	0.39479600	0.23645000
N	-1.79607700	2.60517600	-0.08550800
H	-2.74827500	2.69281000	0.24187000
H	-1.29593600	3.43263000	-0.37104700
O	0.79881900	-1.64157900	-0.17951800
Zn	-1.14069300	-1.47216300	0.08581100

Molecule: Zn-ASN-031

N	3.52952400	0.70684300	-0.31258500
H	3.65283100	0.15474100	-1.16628800
H	4.25253700	0.40758800	0.34482400
H	3.66617900	1.69871000	-0.51955700
C	2.17250500	0.43596100	0.25290000
H	2.15973400	0.84648200	1.25916000
C	1.13332900	1.13531000	-0.63153900
C	1.94207900	-1.08890400	0.30617400
H	1.36603100	2.20068900	-0.67667500
H	1.16578700	0.72336500	-1.63864100
C	-0.26432800	0.96788700	-0.09875300
O	2.71257300	-1.80422000	-0.37559900
O	-1.06205900	0.21821800	-0.71864500
N	-0.59308500	1.63967200	0.98634600
H	-1.51429800	1.53217700	1.38934500
H	0.07812500	2.23255600	1.45138500
O	0.97635100	-1.45505300	1.01332800
Zn	-2.87661500	-0.35316700	-0.11617400

Molecule: Cd-GLN-03

N	0.16857200	3.09435300	-0.96611400
H	0.40055300	3.67091200	-0.15070500
H	-0.72704500	3.42324000	-1.33286400
H	0.88238700	3.22037800	-1.68649500
C	0.08460000	1.66373400	-0.53519300
H	-0.41145100	1.11138600	-1.33018000
C	1.50522300	1.14132800	-0.31664900
C	-0.75221400	1.62125400	0.75010500
H	2.07364000	1.32962100	-1.22853100
H	1.97243200	1.71269900	0.48931200
C	1.54382700	-0.34489400	0.00298200
O	-0.72631100	2.61314300	1.48879100
H	1.02673200	-0.56236400	0.93652000
H	1.02768900	-0.90451400	-0.78693100
C	2.95252300	-0.89489800	0.06352200
O	3.85192600	-0.48826700	-0.68136100
N	3.15007000	-1.87429500	0.95601800
H	4.05132300	-2.32368800	1.01505100
H	2.40768700	-2.18404600	1.56195100
O	-1.40438800	0.55411400	1.01605100
Cd	-1.70225000	-1.19969000	-0.24568500

Molecule: Cd-GLN-04

N	-2.47122700	-2.03013000	-0.73713700
H	-2.99909000	-1.66537300	-1.53693700
H	-3.06467100	-2.71674800	-0.26842700
H	-1.62388400	-2.49887700	-1.06290700
C	-2.16230700	-0.88265900	0.17523200
H	-1.86574200	-1.30201400	1.13317300
C	-1.02295200	-0.07436100	-0.44211200
C	-3.45107200	-0.04704700	0.33217100
H	-0.20728900	-0.76337100	-0.67263100
H	-1.36242700	0.36152800	-1.38444800
C	-0.51792300	1.02749300	0.49302500
O	-4.24909000	-0.06900300	-0.63814600
H	-1.33101000	1.69689300	0.76401000
H	-0.11798700	0.58474300	1.40715300
C	0.58871700	1.81153200	-0.15280300
O	1.73897000	1.33280200	-0.34171000
N	0.32802400	3.04588300	-0.54486200
H	1.04560900	3.59138700	-1.00174800
H	-0.58649600	3.44928800	-0.41402200
O	-3.56839800	0.59662000	1.39908800
Cd	2.39868300	-0.70293400	0.12966300

Molecule: Cd-GLN-05

N	3.87750800	-1.91238400	0.30851100
H	4.17511200	-1.58786900	1.23570500
H	4.71033200	-2.22683800	-0.19213900
H	3.23723000	-2.70263400	0.40476900
C	3.23904100	-0.75493000	-0.39694400
H	3.23884300	-0.98146300	-1.45955200
C	1.81295600	-0.60287500	0.12747300
C	4.09791900	0.49744200	-0.11689900
H	1.28281800	-1.54340800	-0.03695800
H	1.84743700	-0.42100500	1.20410100
C	1.05690700	0.53496200	-0.56705400
O	4.67973200	0.53109000	0.99666800
H	1.55950500	1.48484300	-0.39508500
H	1.01180700	0.35449200	-1.64070400
C	-0.35095000	0.61328600	-0.04880900
O	-1.22347600	-0.16149400	-0.52233900
N	-0.59958600	1.48796300	0.90931700
H	-1.52042100	1.54139000	1.32419500
H	0.13082300	2.08906700	1.25879600
O	4.10508200	1.37762000	-1.00613800
Cd	-3.38010200	-0.18209700	0.00083200

Molecule: Cd-GLN-011

N	-0.80913200	3.07041200	-0.13140300
H	-0.56516100	3.00352300	-1.12342000
H	-0.09034600	3.63369400	0.32870300
H	-1.70706800	3.55187100	-0.04383800
C	-0.88786400	1.70194600	0.46911600
H	-1.03982300	1.82796200	1.53812700
C	-2.06565300	0.95853800	-0.16321000
C	0.43275700	0.98689100	0.21635900
H	-2.95079900	1.58291700	-0.03665300
H	-1.88393500	0.85308200	-1.23554700
C	-2.31549500	-0.40472000	0.46662400
O	1.12079600	1.29988900	-0.77746500
H	-1.47344800	-1.07595100	0.30754400
H	-2.43835200	-0.29574100	1.54864400
C	-3.58581400	-1.04581100	-0.05082000
O	-4.59543900	-0.38369400	-0.32052100
N	-3.55485600	-2.38019000	-0.16923400
H	-4.38580700	-2.87687100	-0.45357000
H	-2.72293300	-2.90423300	0.04856100
O	0.73821600	0.05394200	1.02069500
Cd	2.54644600	-0.68904100	-0.07883700

Molecule: Cd-GLN-022

N	-0.86260600	1.89025300	1.63443600
H	-1.42457700	1.03851200	1.69884200
H	-1.49883800	2.68042100	1.50613100
H	-0.37534500	2.01502600	2.52546600
C	0.13034200	1.81040500	0.51190200
H	0.63742200	2.77104500	0.49286600
C	1.12837600	0.69824100	0.80668900
C	-0.59951500	1.65372000	-0.83071400
H	1.68149900	0.97180800	1.70565400
H	0.59745900	-0.23152300	1.03222700
C	2.09566300	0.46985700	-0.34751600
O	-1.49898200	0.74585500	-0.94659800
H	1.56583700	0.15831300	-1.24779900
H	2.61814700	1.40081400	-0.58754800
C	3.15550900	-0.55091000	0.00364700
O	3.68424400	-0.58722300	1.12156500
N	3.49958500	-1.39578200	-0.97709700
H	4.24073000	-2.06334100	-0.82504600
H	3.06133300	-1.34920200	-1.88263900
O	-0.27976900	2.41658500	-1.74510700
Cd	-1.67226500	-1.16547500	0.05578200

Molecule: Cu-GLN-01

N	0.23213700	2.80089700	-0.00608000
H	0.39963700	2.77700400	-1.01607500
H	1.07525400	3.16597800	0.44410000
H	-0.53886000	3.44790600	0.17799800
C	-0.11830300	1.44008600	0.50830100
H	-0.25982300	1.53029600	1.58394400
C	-1.40833900	0.96738900	-0.16815400
C	1.04358200	0.51531400	0.24917700
H	-2.12833400	1.78241400	-0.08991900
H	-1.21431900	0.79903900	-1.22950200
C	-1.99221500	-0.28083600	0.47663000
O	1.93919700	0.80644300	-0.59611600
H	-1.32268500	-1.13379700	0.37679600
H	-2.13410100	-0.11208400	1.54872500
C	-3.35152600	-0.63155200	-0.09188400
O	-4.11748600	0.22409000	-0.55017100
N	-3.68128800	-1.92893300	-0.03489300
H	-4.59836700	-2.22514600	-0.33325200
H	-3.04459600	-2.61201200	0.34179800
O	1.12642700	-0.59139700	0.86035300
Cu	2.80296400	-1.00340800	-0.17499100

Molecule: Cu-GLN-05

N	-3.06027900	1.97152300	0.07146200
H	-3.38436300	1.79776500	1.02952500
H	-3.87153800	2.25657500	-0.47967200
H	-2.37742000	2.73067700	0.06265300
C	-2.48598100	0.68959100	-0.45063100
H	-2.46507600	0.76040500	-1.53497600
C	-1.07225000	0.53227200	0.10509600
C	-3.42477400	-0.45426300	-0.00993100
H	-0.49884100	1.42088100	-0.16261000
H	-1.11581000	0.47830100	1.19547900
C	-0.37317600	-0.70796700	-0.44976600
O	-4.06958800	-0.26268400	1.05197200
H	-0.87643500	-1.61917800	-0.13569700
H	-0.38304700	-0.68094800	-1.54309200
C	1.06771300	-0.77248000	-0.04458800
O	1.78776700	0.25115400	-0.24306300
N	1.53191500	-1.88547200	0.47308700
H	2.50818100	-1.96460700	0.72777700
H	0.92129400	-2.67591900	0.61821900
O	-3.43402000	-1.47600200	-0.73170500
Cu	3.64479800	0.45072500	0.03075300

Molecule: Cu-GLN-022

N	1.43122300	1.14337800	-1.79676700
H	1.83781500	0.20363800	-1.78102700
H	2.19202900	1.82322600	-1.72642300
H	0.97389800	1.26833400	-2.70346800
C	0.42898500	1.33667900	-0.69608900
H	0.07277300	2.35780400	-0.79907800
C	-0.72364600	0.35683600	-0.87076100
C	1.11291400	1.24355200	0.67446200
H	-1.14651200	0.51397500	-1.86373700
H	-0.36211000	-0.67400100	-0.83644100
C	-1.79276900	0.57655100	0.18818400
O	1.75467500	0.16537900	0.99361200
H	-1.39955900	0.39933400	1.19094400
H	-2.12979200	1.61716900	0.16882000
C	-3.01396200	-0.29492600	-0.01697400
O	-3.23799500	-0.88508700	-1.08004800
N	-3.84783800	-0.36809000	1.03019000
H	-4.71310200	-0.87900500	0.94394400
H	-3.65821100	0.13208000	1.88378800
O	1.02041100	2.19260700	1.44495100
Cu	1.82355900	-1.49258400	0.14991900

Molecule: Cu-GLN-031

N	-1.23671100	2.63410800	-0.79791100
H	-1.20378300	3.20342600	0.05278100
H	-2.20314100	2.62838600	-1.13164500
H	-0.64782100	3.06860300	-1.51214300
C	-0.75892500	1.24875800	-0.49693300
H	-0.93347700	0.64806600	-1.38856200
C	0.73555900	1.31119600	-0.17469200
C	-1.59805900	0.71891900	0.66757500
H	1.22828100	1.79425500	-1.01873400
H	0.88397500	1.94285700	0.70401600
C	1.35724400	-0.05849800	0.05331200
O	-2.03263800	1.50418400	1.50426700
H	0.98532900	-0.51396300	0.97089200
H	1.08333800	-0.72967200	-0.77136700
C	2.87080900	-0.02110600	0.07565200
O	3.52114500	0.74490900	-0.64426100
N	3.45586300	-0.90242500	0.89693900
H	4.46185100	-0.97484600	0.91751700
H	2.90971400	-1.51187000	1.48376200
O	-1.80366300	-0.55628200	0.73884700
Cu	-1.21437400	-1.87638300	-0.43241400

Molecule: Cu-GLN-041

N	-2.31739300	-1.77953900	-0.88229600
H	-2.86396100	-1.28337200	-1.59462700
H	-2.94467600	-2.43790900	-0.41722300
H	-1.56308000	-2.30655600	-1.32649300
C	-1.79476300	-0.76426600	0.08832800
H	-1.50406300	-1.29716800	0.98988700
C	-0.58553800	-0.08174200	-0.54532800
C	-2.93728400	0.22548900	0.40151000
H	0.14663500	-0.85820000	-0.78038800
H	-0.88347600	0.38509800	-1.48680900
C	0.04828600	0.96850300	0.37271900
O	-3.78960300	0.39444900	-0.50529700
H	-0.61491800	1.81800100	0.51433500
H	0.25872300	0.52877400	1.35044500
C	1.35715000	1.43364800	-0.18976100
O	2.36321400	0.66056500	-0.28670400
N	1.47303700	2.67474300	-0.59855100
H	2.34738900	3.00872100	-0.98229300
H	0.69054000	3.30898800	-0.53430100
O	-2.89050000	0.78803500	1.51843700
Cu	2.44308200	-1.12300000	0.27783100

Molecule: Fe-GLN-01

N	0.35655600	2.76880700	0.00415200
H	0.52787000	2.74705500	-1.00602900
H	1.20282600	3.11843300	0.46213400
H	-0.40339400	3.43053400	0.18507800
C	-0.02972000	1.41777400	0.51455300
H	-0.17878600	1.50747900	1.59056100
C	-1.32069700	0.95778900	-0.17450300
C	1.11163500	0.47543200	0.27502000
H	-2.00807400	1.80354500	-0.15168300
H	-1.10749300	0.73379700	-1.22124000
C	-1.96668100	-0.23265700	0.51785500
O	2.10976500	0.78896900	-0.44706300
H	-1.31214000	-1.10325000	0.51615100
H	-2.16538700	0.01213300	1.56581100
C	-3.29721700	-0.59640400	-0.10889300
O	-3.98770100	0.22945700	-0.71683800
N	-3.68736100	-1.86601900	0.06629800
H	-4.59301000	-2.16130400	-0.26607600
H	-3.10972700	-2.52446600	0.56343100
O	1.12819000	-0.69839600	0.75929700
Fe	2.90296100	-1.09905100	-0.21687100

Molecule: Fe-GLN-023

N	1.41504200	-1.28720300	1.74430900
H	1.88295700	-0.37666700	1.75367700
H	2.13281900	-2.01337000	1.67564400
H	0.93110000	-1.39900900	2.63965700
C	0.42728500	-1.39788900	0.62186000
H	0.06458700	-2.42177100	0.63716300
C	-0.72508700	-0.41883800	0.83028800
C	1.12433900	-1.20021800	-0.72202300
H	-1.15102700	-0.62090000	1.81520300
H	-0.35854300	0.60903900	0.84132000
C	-1.79701700	-0.57355700	-0.24328000
O	1.87059900	-0.10581100	-0.86482900
H	-1.37231400	-0.40164100	-1.23664900
H	-2.20379900	-1.58474100	-0.23456000
C	-2.89889800	0.44944800	-0.07665400
O	-2.64706000	1.63980900	0.15084700
N	-4.14923000	-0.00508900	-0.21653600
H	-4.92388500	0.63937600	-0.16544500
H	-4.33216000	-0.97825700	-0.40065700
O	0.99075900	-1.98455400	-1.62948000
Fe	1.92198000	1.54018300	-0.06666200

Molecule: Fe-GLN-031

N	-1.10504300	2.64980300	-0.82278900
H	-0.93987000	3.25063000	-0.00985700
H	-2.09643600	2.71438000	-1.06750500
H	-0.55369200	3.00698000	-1.60740600
C	-0.70953200	1.24164000	-0.52198100
H	-0.95175300	0.64702700	-1.40241100
C	0.79410500	1.19733800	-0.24476200
C	-1.52957700	0.76934500	0.66789400
H	1.29008900	1.62569100	-1.11625100
H	1.01363600	1.83402300	0.61523100
C	1.32796400	-0.21249100	-0.00413500
O	-1.97169200	1.52747400	1.49951000
H	0.93641300	-0.62831100	0.92228100
H	1.02817700	-0.87017800	-0.82635300
C	2.84273300	-0.20575900	0.02807600
O	3.50502000	0.19353700	-0.93681300
N	3.40924000	-0.65559600	1.15381700
H	4.41487200	-0.66525500	1.23819100
H	2.85061900	-0.95366200	1.93677000
O	-1.69987300	-0.54338400	0.76231700
Fe	-1.46704700	-1.92623900	-0.42886800

Molecule: Fe-GLN-041

N	-2.31288900	-1.79189000	-0.85598800
H	-2.84949300	-1.29293200	-1.57403400
H	-2.95753100	-2.42227100	-0.37560800
H	-1.57761900	-2.35013700	-1.29396700
C	-1.75859100	-0.77781600	0.09608500
H	-1.47382800	-1.30474800	1.00292200
C	-0.53863000	-0.12768600	-0.55175700
C	-2.87782900	0.24214500	0.40761700
H	0.19286400	-0.91480300	-0.75307700
H	-0.82061600	0.31096900	-1.51101800
C	0.07285600	0.95367100	0.34770000
O	-3.73474500	0.41577900	-0.49271600
H	-0.58843800	1.81322900	0.42699200
H	0.24547400	0.55459700	1.34926700
C	1.39568500	1.40202400	-0.17027300
O	2.42081700	0.61594700	-0.12132800
N	1.56544900	2.58715900	-0.67160000
H	2.47341000	2.87539700	-1.01950400
H	0.79056600	3.23721900	-0.71502400
O	-2.80524800	0.81699100	1.51627700
Fe	2.57652900	-1.19296800	0.27546400

Molecule: Fe-GLN-052

N	2.94595400	-1.98071700	0.07961200
H	3.25589700	-1.80466500	1.04215600
H	3.76591400	-2.26576300	-0.45871300
H	2.26433200	-2.74082900	0.06151100
C	2.38116500	-0.70057500	-0.45475500
H	2.38155900	-0.77149500	-1.53924200
C	0.95689600	-0.54112900	0.07449700
C	3.30856100	0.44597600	0.00481900
H	0.38712300	-1.43370600	-0.18872800
H	0.97998600	-0.46554900	1.16395900
C	0.27147100	0.69205000	-0.51770300
O	3.94293300	0.25069300	1.07174700
H	0.80141000	1.60551900	-0.25847400
H	0.24095100	0.61357900	-1.60807600
C	-1.14290500	0.81232800	-0.06436200
O	-1.92083300	-0.19422000	-0.26013800
N	-1.57259500	1.90468100	0.49289200
H	-2.54027700	1.99486300	0.78151300
H	-0.94125100	2.68165100	0.64132900
O	3.31761100	1.47261300	-0.70985900
Fe	-3.75299900	-0.51407400	0.04947100

Molecule: Hg-GLN-01

N	-1.35012800	3.00314900	-0.10254000
H	-1.12434900	2.93967400	-1.09886000
H	-0.59730300	3.52282600	0.35440200
H	-2.22130200	3.52895600	0.00056300
C	-1.50413800	1.63795900	0.49124000
H	-1.72991700	1.77733400	1.54659700
C	-2.65428900	0.91663200	-0.21437500
C	-0.18617600	0.88398500	0.37443700
H	-3.49324700	1.61196000	-0.26094000
H	-2.36048500	0.68885800	-1.24142400
C	-3.09231200	-0.34748000	0.51165200
O	0.70030800	1.34756100	-0.40606600
H	-2.28631600	-1.07692400	0.55547200
H	-3.35880100	-0.10557200	1.54542300
C	-4.31654700	-0.97691100	-0.11714800
O	-5.19237100	-0.30702700	-0.67783600
N	-4.40350100	-2.30892400	0.00238500
H	-5.21864300	-2.79088100	-0.34516300
H	-3.67578100	-2.83967500	0.45273200
O	-0.04731800	-0.16924900	1.02761200
Hg	2.16471700	-0.39714400	-0.08290300

Molecule: Hg-GLN-05

N	4.32804400	-2.01212400	-0.05773600
H	4.53150800	-1.96734600	0.94653600
H	5.20228200	-2.23861200	-0.53499800
H	3.65115300	-2.75396200	-0.24616600
C	3.82473200	-0.66884700	-0.48894800
H	3.91083300	-0.62609700	-1.57173500
C	2.36563000	-0.52977000	-0.05631700
C	4.72417800	0.40349200	0.16180900
H	1.81940500	-1.41476900	-0.38547300
H	2.31415400	-0.49281300	1.03448500
C	1.71548000	0.71655600	-0.65577500
O	5.27428200	0.08468400	1.24569800
H	2.25685700	1.61626500	-0.37324900
H	1.72414500	0.64971300	-1.74678700
C	0.27865700	0.85647600	-0.24300900
O	-0.48927000	-0.13381200	-0.43647800
N	-0.11021700	1.99830400	0.27777000
H	-1.07928600	2.13696200	0.53967000
H	0.54757600	2.75182900	0.41042500
O	4.79430100	1.50408800	-0.43022200
Hg	-2.60612500	-0.17339500	0.06310600

Molecule: Hg-GLN-023

N	-0.36313100	2.15391500	1.65578700
H	-1.06116600	1.40473500	1.64479400
H	-0.85495000	3.04512400	1.55469300
H	0.09625100	2.14558200	2.57043300
C	0.65100100	1.97692500	0.56510600
H	1.26244400	2.87515000	0.57453300
C	1.51409600	0.75829900	0.85952100
C	-0.05508800	1.94383000	-0.79770600
H	2.02663700	0.92673800	1.80687900
H	0.87887300	-0.12196600	1.00003700
C	2.52195900	0.47773500	-0.24733100
O	-1.00281000	1.08716600	-1.02493500
H	2.03351600	0.42393900	-1.22042600
H	3.25775100	1.28529200	-0.30026800
C	3.27952200	-0.80692600	0.00886700
O	3.61334500	-1.15297300	1.14858400
N	3.57469500	-1.53642000	-1.07513400
H	4.11255100	-2.38421000	-0.97472900
H	3.29172700	-1.24270500	-1.99606000
O	0.29200400	2.75447900	-1.65046300
Hg	-1.35267300	-0.75360900	0.01449100

Molecule: Hg-GLN-031

N	1.10583400	3.04051100	-1.10840400
H	1.54558800	3.58070600	-0.35635200
H	0.27474000	3.55026500	-1.41550700
H	1.75403400	2.97286400	-1.89650400
C	0.74855700	1.68114700	-0.59791100
H	0.09045300	1.22497800	-1.33501200
C	2.03807500	0.87537300	-0.44215100
C	0.01308600	1.86830400	0.73109600
H	2.51574500	0.83436900	-1.42203800
H	2.70664200	1.41358700	0.23458600
C	1.82056200	-0.54521500	0.07620000
O	0.30657800	2.83022600	1.43839400
H	1.41448000	-0.53333700	1.08589900
H	1.11241800	-1.07133300	-0.57058300
C	3.11585500	-1.32698900	0.03424900
O	3.70701600	-1.53480200	-1.03284300
N	3.57626000	-1.77361800	1.20895500
H	4.43927800	-2.29592000	1.24276100
H	3.08456200	-1.58557100	2.06717700
O	-0.86847800	0.99598200	1.09848300
Hg	-1.54112900	-0.63257300	-0.11474300

Molecule: Hg-GLN-041

N	-2.81810300	-2.00394500	-0.84640300
H	-3.47567900	-1.65323700	-1.55186400
H	-3.27003500	-2.78432300	-0.36635900
H	-1.96836100	-2.34783200	-1.29795900
C	-2.53523400	-0.88381000	0.10816900
H	-2.14964900	-1.32581200	1.02300000
C	-1.49696300	0.03641700	-0.52795800
C	-3.86730300	-0.15472700	0.38894000
H	-0.64429900	-0.57957700	-0.82471900
H	-1.91399000	0.48294400	-1.43313600
C	-1.03553700	1.13476300	0.43367800
O	-4.72593500	-0.19514900	-0.52766200
H	-1.86899700	1.77677500	0.70744900
H	-0.63014900	0.68528900	1.34260900
C	0.05292000	1.96651400	-0.17133800
O	1.23836500	1.54585100	-0.34157400
N	-0.23060800	3.19473600	-0.54818200
H	0.48308000	3.77502700	-0.96794800
H	-1.15976100	3.56859300	-0.42793800
O	-3.95461800	0.43306300	1.48978500
Hg	1.88461300	-0.45998100	0.09007000

Molecule: Mn-GLN-11

N	0.13114800	2.81677700	-0.16719700
H	0.33813800	2.72204100	-1.16494500
H	0.94123700	3.25625600	0.27526300
H	-0.67668600	3.43308000	-0.05285200
C	-0.14595900	1.47233500	0.43250700
H	-0.28048500	1.62449600	1.50107800
C	-1.42277500	0.91717300	-0.19458600
C	1.06565700	0.57785200	0.20271500
H	-2.19919400	1.67571500	-0.08596000
H	-1.25774500	0.76192500	-1.26377600
C	-1.89725000	-0.37716700	0.45860800
O	1.83803800	0.83640200	-0.75618200
H	-1.19510400	-1.18919100	0.28032100
H	-1.96973100	-0.23788600	1.54113400
C	-3.28120900	-0.75733000	-0.02145600
O	-4.20216500	0.06817200	-0.06946800
N	-3.45584900	-2.03876100	-0.36920700
H	-4.36973800	-2.35536900	-0.65704800
H	-2.69874200	-2.70111400	-0.32754900
O	1.22408300	-0.39556900	0.98605100
Mn	3.19402100	-1.10021200	-0.11363100

Molecule: Mn-GLN-023

N	1.48117000	-1.39177300	1.60706800
H	1.96567700	-0.48993300	1.58832200
H	2.18833500	-2.12580300	1.53163800
H	1.00421800	-1.49356000	2.50562900
C	0.50647800	-1.46897400	0.47015300
H	0.16469100	-2.49895900	0.41870400
C	-0.66471800	-0.53525300	0.76270400
C	1.24075300	-1.12509700	-0.83436200
H	-1.13166300	-0.86721200	1.69049600
H	-0.29925300	0.48139000	0.93147600
C	-1.69753800	-0.53013800	-0.35605500
O	2.12370800	-0.20391200	-0.77053800
H	-1.27926600	-0.11812800	-1.27394200
H	-2.00976500	-1.55500700	-0.57888700
C	-2.94928300	0.22876300	0.02929400
O	-3.41783900	0.18527400	1.17370700
N	-3.53403000	0.92660700	-0.95347000
H	-4.40474500	1.40462600	-0.77665100
H	-3.14312000	0.94851300	-1.88114200
O	0.93036900	-1.75196300	-1.85319400
Mn	1.82443400	1.77277000	0.09755900

Molecule: Mn-GLN-031

N	-1.36262800	2.47676800	-0.94724200
H	-1.37910400	3.09647100	-0.13110600
H	-2.31804800	2.41211500	-1.30382900
H	-0.77072500	2.88525200	-1.67332100
C	-0.85296200	1.13374400	-0.52562000
H	-1.04444700	0.44252400	-1.34432900
C	0.64665600	1.24929600	-0.26190400
C	-1.64153500	0.70870700	0.72331100
H	1.10852300	1.67840300	-1.15191600
H	0.81225300	1.94275300	0.56610900
C	1.30185400	-0.09284000	0.03841600
O	-2.02688300	1.60450200	1.49158700
H	0.93548200	-0.51438200	0.97303000
H	1.05587600	-0.80091600	-0.76194400
C	2.81192100	0.00456100	0.06056000
O	3.43601300	0.63723900	-0.80015000
N	3.42854200	-0.66068300	1.04556100
H	4.43644800	-0.66437700	1.09095000
H	2.90459200	-1.15491000	1.74915900
O	-1.82344000	-0.53657700	0.89812900
Mn	-1.21933500	-2.14790600	-0.46504500

Molecule: Mn-GLN-43

N	-2.15147300	-1.20287500	-1.30510600
H	-2.84608800	-0.58184800	-1.73456200
H	-2.65303000	-2.00363900	-0.91727300
H	-1.49756800	-1.53899300	-2.01409600
C	-1.46188900	-0.43025800	-0.22406100
H	-0.96987600	-1.14763200	0.42986900
C	-0.43029200	0.49039500	-0.86825300
C	-2.53746000	0.35328600	0.55640000
H	0.25426400	-0.13748900	-1.44268800
H	-0.92901800	1.16184100	-1.56846300
C	0.36136000	1.30306700	0.16991600
O	-3.55577700	0.69413700	-0.09593100
H	-0.02086500	2.31729500	0.25603600
H	0.27137700	0.83825400	1.15399700
C	1.83148400	1.29996900	-0.14033100
O	2.46343000	0.22240900	-0.24515300
N	2.44341900	2.46520300	-0.28897900
H	3.43650900	2.49482200	-0.47099900
H	1.93295900	3.33053300	-0.21006500
O	-2.28306100	0.60404900	1.75608300
Mn	1.65607000	-1.75331800	0.37579300

Molecule: Mn-GLN-51

N	-3.05981600	-1.92768700	-0.20459200
H	-3.49955300	-1.62506700	-1.08079900
H	-3.80140000	-2.26506200	0.41121500
H	-2.41136600	-2.69639100	-0.38538600
C	-2.36969300	-0.73712200	0.38976200
H	-2.20335500	-0.95006900	1.44252400
C	-1.04106500	-0.54295800	-0.33696000
C	-3.31134900	0.47533700	0.23953300
H	-0.44442700	-1.44521600	-0.19591300
H	-1.23034500	-0.43213400	-1.40751000
C	-0.26561500	0.66509300	0.17890300
O	-4.08199600	0.46205200	-0.75334600
H	-0.75443800	1.59689400	-0.09831400
H	-0.21979400	0.63537100	1.27230100
C	1.16550700	0.67548400	-0.28125400
O	1.84158600	-0.38098400	-0.27171100
N	1.68276500	1.83693400	-0.65326800
H	2.65579200	1.89256100	-0.92032900
H	1.12422400	2.67636400	-0.65948200
O	-3.20385000	1.37282200	1.10582900
Mn	3.95645600	-0.46392400	0.23362500

Molecule: Ni-GLN-13

N	0.26339900	2.78789300	-0.18647800
H	0.43659600	2.68693600	-1.19103000
H	1.10252400	3.18840000	0.24186000
H	-0.50766900	3.44897800	-0.05932300
C	-0.09155200	1.47105800	0.43039900
H	-0.21186800	1.63565100	1.49891500
C	-1.39528700	0.96436600	-0.18872500
C	1.05201300	0.51410200	0.21861400
H	-2.14532800	1.73837800	-0.02433100
H	-1.25762900	0.85144300	-1.26631700
C	-1.87802000	-0.34648800	0.42256100
O	1.81346900	0.61080600	-0.79188200
H	-1.21041100	-1.16946200	0.17057700
H	-1.89897800	-0.26222300	1.51283200
C	-3.29430700	-0.65926100	-0.01404100
O	-4.17809200	0.20635100	-0.01624400
N	-3.53610200	-1.92631900	-0.37137500
H	-4.47209400	-2.19933000	-0.63164800
H	-2.80623300	-2.61979900	-0.37084200
O	1.26138400	-0.42700700	1.04090700
Ni	2.79817300	-1.00406600	-0.10895800

Molecule: Ni-GLN-023

N	1.35058300	1.73123000	-1.46223100
H	1.74303700	0.83142500	-1.75451500
H	2.12718200	2.35625500	-1.23141200
H	0.84530100	2.12988000	-2.25812700
C	0.41690100	1.56640800	-0.30077000
H	0.08099500	2.56360000	-0.03001700
C	-0.76706700	0.70548300	-0.73288300
C	1.16648700	1.00252600	0.90605400
H	-1.26943200	1.22743100	-1.54769400
H	-0.40680200	-0.24548600	-1.13658400
C	-1.74317900	0.45902400	0.40692700
O	1.97394500	-0.01655300	0.69858100
H	-1.27461100	-0.10257900	1.21616800
H	-2.06791300	1.41270400	0.83415300
C	-2.99080200	-0.26986700	-0.04605200
O	-3.39975500	-0.22137900	-1.21174400
N	-3.63658100	-0.95009600	0.91113900
H	-4.50683700	-1.41260600	0.69629500
H	-3.28856700	-0.97730700	1.85593800
O	1.01564100	1.49323900	2.00641000
Ni	1.81453400	-1.57411300	-0.21900700

Molecule: Ni-GLN-033

N	-0.98286600	2.71830900	-0.79810700
H	-0.85319100	3.29656800	0.03698400
H	-1.96142900	2.79646700	-1.08477800
H	-0.39315900	3.08617700	-1.54842200
C	-0.61742700	1.29947700	-0.50005500
H	-0.85885400	0.71865100	-1.38910800
C	0.88029600	1.23541100	-0.20369800
C	-1.46817600	0.84233200	0.68193300
H	1.39913500	1.66203400	-1.06242900
H	1.09622700	1.86323400	0.66389700
C	1.38465000	-0.18097600	0.03459600
O	-1.81242600	1.64396000	1.53496000
H	0.98432600	-0.59482800	0.95920000
H	1.05623200	-0.83331400	-0.78311500
C	2.89816400	-0.24871600	0.04175000
O	3.58259000	0.38982600	-0.76630400
N	3.44216600	-1.06643100	0.95164600
H	4.44291000	-1.19378100	0.97367700
H	2.87078000	-1.56597700	1.61340400
O	-1.77970800	-0.42469500	0.75402300
Ni	-1.54952700	-1.83415200	-0.42723800

Molecule: Ni-GLN-043

N	-2.32677400	-1.96517900	-0.58105100
H	-2.77412600	-1.60692700	-1.43103600
H	-3.02990800	-2.50862800	-0.07706000
H	-1.55031700	-2.58167700	-0.82778400
C	-1.87715300	-0.79457100	0.23808700
H	-1.66367700	-1.16381500	1.23805200
C	-0.61980100	-0.21094100	-0.40310100
C	-3.03394900	0.22606000	0.28119400
H	0.08726200	-1.02733200	-0.57245000
H	-0.87135700	0.21625700	-1.37633000
C	0.02816000	0.85710600	0.48800100
O	-3.85463200	0.18051800	-0.66896700
H	-0.66851000	1.66821500	0.68170400
H	0.31965400	0.41370500	1.44251100
C	1.26186400	1.41166600	-0.14419500
O	2.34933400	0.74309200	-0.24933400
N	1.24444700	2.62950200	-0.61697800
H	2.06825300	3.01821600	-1.05887300
H	0.40933800	3.19375800	-0.54757100
O	-3.02980200	1.02586100	1.24384500
Ni	2.74913400	-1.02870400	0.19824200

Molecule: Ni-GLN-053

N	-3.06089700	1.94959900	0.28636000
H	-3.37945900	1.65566600	1.21631700
H	-3.87753900	2.28170000	-0.22955800
H	-2.39556000	2.71981600	0.37396500
C	-2.45446300	0.75851700	-0.39027200
H	-2.41011600	0.97687800	-1.45377300
C	-1.05257300	0.54865300	0.17814900
C	-3.37628000	-0.45275800	-0.13208000
H	-0.47050100	1.45485100	0.00213500
H	-1.12214900	0.39651400	1.25778000
C	-0.34414400	-0.64707600	-0.46614100
O	-4.00258200	-0.45041700	0.95700300
H	-0.86791900	-1.57441700	-0.24623500
H	-0.30744700	-0.51923700	-1.54933800
C	1.06869400	-0.74929000	0.01511800
O	1.87611500	0.16575500	-0.34226100
N	1.41491900	-1.75779300	0.77588700
H	2.36286500	-1.84439400	1.12077700
H	0.73533600	-2.45879400	1.03404600
O	-3.38517600	-1.33957400	-1.01490800
Ni	3.72500200	0.42194300	-0.03532700

Molecule: Zn-GLN-01

N	0.18312500	2.67950400	-0.42937500
H	0.57439000	2.32382700	-1.30583200
H	0.91925300	3.20317500	0.04932500
H	-0.58302300	3.32132700	-0.64694200
C	-0.30528200	1.54128600	0.41460300
H	-0.59085600	1.95879600	1.37658300
C	-1.50943500	0.90709000	-0.27873200
C	0.83091800	0.54096700	0.62552500
H	-2.24952200	1.69270800	-0.43903500
H	-1.20149800	0.53448700	-1.25869500
C	-2.13805100	-0.22080100	0.53423800
O	1.67554700	0.47672800	-0.34278800
H	-1.44195600	-1.04792700	0.65914000
H	-2.39868900	0.14268300	1.53234300
C	-3.42022000	-0.71093600	-0.10199100
O	-4.29104900	0.07206300	-0.50207200
N	-3.56209000	-2.04004600	-0.18842400
H	-4.41372100	-2.42813700	-0.56572900
H	-2.84718100	-2.66466100	0.14713700
O	0.84340400	-0.14062700	1.64934700
Zn	3.04382500	-0.90411400	-0.29404900

Molecule: Zn-GLN-04

N	-2.31316600	-1.81574800	-0.88269900
H	-2.85574900	-1.32031400	-1.59874900
H	-2.93981300	-2.48257200	-0.42902700
H	-1.54827900	-2.33240600	-1.32057800
C	-1.81259400	-0.79920500	0.09826400
H	-1.53090200	-1.33174800	1.00289900
C	-0.60200200	-0.10245300	-0.51723900
C	-2.96929500	0.17896100	0.39639100
H	0.14518000	-0.86787500	-0.74356700
H	-0.89552300	0.36321600	-1.46107400
C	0.00943000	0.95251900	0.40812300
O	-3.80422500	0.34884800	-0.52666700
H	-0.69422500	1.75947400	0.59555200
H	0.27545400	0.49874700	1.36568300
C	1.27098700	1.49894900	-0.19043400
O	2.30116200	0.78730800	-0.35487300
N	1.28818100	2.76296800	-0.56461200
H	2.12226800	3.16035400	-0.97472700
H	0.47445700	3.34562400	-0.44225700
O	-2.95210300	0.73234600	1.51874500
Zn	2.49614000	-1.09145600	0.26229200

Molecule: Zn-GLN-022

N	-1.43516600	1.42281600	1.60653100
H	-1.77313500	0.46599800	1.74379200
H	-2.24706500	2.02745900	1.46143000
H	-0.96577000	1.71899300	2.46605000
C	-0.48604800	1.50856700	0.44663800
H	-0.20380400	2.55433100	0.36495300
C	0.74024000	0.65490800	0.74759000
C	-1.20876300	1.12781400	-0.85735800
H	1.21749000	1.05921000	1.64087200
H	0.43874500	-0.36899800	0.98702500
C	1.73897800	0.63439800	-0.40506200
O	-1.85883600	0.01459300	-0.90712100
H	1.28520800	0.22563900	-1.30774000
H	2.06638700	1.65251500	-0.63138800
C	2.97204400	-0.15814900	-0.02924900
O	3.57600900	0.05077300	1.03050300
N	3.37019900	-1.08882700	-0.90591300
H	4.20382600	-1.62597300	-0.71880100
H	2.87303700	-1.24352700	-1.76752400
O	-1.12995200	1.90746700	-1.80458000
Zn	-1.58922100	-1.57304800	0.16304100

Molecule: Zn-GLN-031

N	-1.14756000	2.67578000	-0.83820000
H	-1.07867200	3.25871300	0.00109300
H	-2.11928200	2.70074100	-1.15402500
H	-0.55508100	3.07172200	-1.57116900
C	-0.72323900	1.27822900	-0.50958600
H	-0.95190500	0.66223800	-1.37764100
C	0.77955500	1.28849600	-0.23059300
C	-1.54716300	0.81552100	0.69720200
H	1.27122800	1.72719100	-1.09925800
H	0.97561300	1.93999500	0.62460200
C	1.36129800	-0.09384400	0.02822800
O	-1.93592600	1.66251800	1.50479700
H	0.97079600	-0.52188200	0.95042200
H	1.08361500	-0.77524700	-0.78535500
C	2.87527000	-0.07647400	0.06896000
O	3.54431100	0.63863100	-0.68611600
N	3.44172300	-0.90928600	0.95190700
H	4.44697900	-0.98260800	0.99566000
H	2.88281800	-1.47343500	1.57115200
O	-1.76906200	-0.44289900	0.82308400
Zn	-1.27247200	-1.87034800	-0.41369400

Molecule: Zn-GLN-051

N	3.11977600	-1.95208300	0.29116400
H	3.49247200	-1.64824700	1.19760900
H	3.90087300	-2.31799500	-0.25580200
H	2.43771900	-2.70058300	0.42675600
C	2.51627100	-0.75387000	-0.37671900
H	2.42945500	-0.98110100	-1.43585300
C	1.13916900	-0.50905300	0.23624500
C	3.47374100	0.43717500	-0.16542300
H	0.52863500	-1.39623400	0.06049200
H	1.24332200	-0.38192000	1.31649400
C	0.44077300	0.71679500	-0.35990100
O	4.15741300	0.42037500	0.88860500
H	0.94157100	1.63522100	-0.06251200
H	0.45618700	0.65793400	-1.45016000
C	-1.00220100	0.75697400	0.04787200
O	-1.75528000	-0.17403600	-0.34721800
N	-1.42697400	1.75443700	0.79562800
H	-2.39726100	1.80396900	1.07690500
H	-0.79390900	2.48242000	1.09095000
O	3.45612800	1.32409300	-1.04813800
Zn	-3.67870900	-0.40738400	-0.06036200

Molecule: Cd-GLY-01

N	3.46250700	-0.57662500	-0.00014200
H	3.27486700	-1.15065800	-0.82510200
C	2.61104100	0.64194800	0.00022500
H	3.27426500	-1.15161200	0.82403300
H	4.45207900	-0.31920400	0.00034600
C	1.14231800	0.28873500	0.00003800
H	2.84831700	1.22769200	0.88508000
H	2.84838000	1.22827800	-0.88422400
O	0.78310300	-0.91182300	0.00007100
O	0.33072100	1.26001300	-0.00018200
Cd	-1.50762900	-0.08682800	0.00000300

Molecule: Cd-GLY-021

N	3.13475800	-1.08570700	-0.08208500
H	3.72482700	-0.49851600	0.51232200
C	1.69921000	-0.78355000	0.15809200
H	3.38866400	-0.88996900	-1.05260700
H	3.33672100	-2.06790000	0.11391500
C	1.43749800	0.71035000	0.03945200
H	1.10413100	-1.34076100	-0.56134100
H	1.44531600	-1.11844900	1.16210500
O	2.39516500	1.47911700	-0.08216500
O	0.21437300	1.09593800	0.10198600
Cd	-1.55499000	-0.13845200	-0.01965900

Molecule: Cu-GLY-02

N	-2.76590500	-0.91193700	0.05007400
H	-3.25678400	-0.40443300	-0.68995100
C	-1.29366600	-0.79084600	-0.11936400
H	-3.06158300	-0.50810800	0.94189000
H	-3.05444000	-1.89203200	0.02009700
C	-0.87458500	0.66400900	-0.05182800
H	-0.80660700	-1.35748500	0.67174400
H	-1.01960200	-1.21803500	-1.08107800
O	-1.70493000	1.53990300	0.16189800
O	0.38318000	0.89963400	-0.25401400
Cu	1.86702900	-0.24109000	0.05347800

Molecule: Cu-GLY-011

N	-3.03203500	-0.41872600	-0.00110100
H	-2.90277900	-1.01455900	0.82013800
C	-2.05752400	0.70500700	0.00204300
H	-2.90825200	-1.00503800	-0.82999000
H	-3.98936700	-0.06000200	0.00392300
C	-0.64909900	0.17816400	-0.00018800
H	-2.22718000	1.31725500	-0.88068300
H	-2.22564400	1.31089200	0.88952100
O	-0.39741900	-1.03631000	0.00047500
O	0.29418200	1.05239800	-0.00179600
Cu	1.81183100	-0.10500700	0.00014600

Molecule: Fe-GLY-01

N	2.77628600	-0.60158800	0.14687000
H	2.68565800	-1.22541500	-0.65983300
C	2.00353900	0.65546300	-0.04460800
H	2.44772400	-1.10984300	0.97370900
H	3.76778100	-0.38587700	0.27930000
C	0.54555600	0.35552300	-0.12751400
H	2.18458800	1.31401900	0.80217900
H	2.33978500	1.14922700	-0.95539600
O	0.12257000	-0.83472000	-0.25801000
O	-0.34534000	1.25421200	0.00666500
Fe	-1.78353700	-0.19049400	0.06059400

Molecule: Fe-GLY-02

N	-2.67645100	-0.92577000	-0.09714500
H	-2.91230600	-0.59302100	-1.03586200
C	-1.22911200	-0.76701800	0.17580600
H	-3.23944800	-0.39941900	0.57584500
H	-2.93447000	-1.91399300	-0.03199600
C	-0.80301100	0.67312000	0.05150400
H	-1.02092800	-1.11620400	1.18612000
H	-0.66954900	-1.38080500	-0.52884100
O	-1.57899200	1.58819200	-0.08596300
O	0.51374600	0.87254400	0.12674200
Fe	1.93179100	-0.27841000	-0.04520500

Molecule: Hg-GLY-011

N	-4.11292400	-0.31289500	-0.01207100
H	-4.15666100	-0.72865700	0.92118800
C	-3.04422500	0.71797900	-0.08223300
H	-3.93035400	-1.06937600	-0.67637000
H	-5.02307200	0.10076500	-0.22449600
C	-1.67776700	0.07385300	0.05170600
H	-3.10732900	1.21158300	-1.04961100
H	-3.21422100	1.45032800	0.70133900
O	-1.55152800	-1.14678800	0.04845400
O	-0.72463100	0.94122000	0.12690000
Hg	1.18454200	-0.02351000	-0.01009000

Molecule: Hg-GLY-021

N	-3.50514300	-1.09758000	-0.07440900
H	-3.78305200	-0.88349200	-1.03489800
C	-2.07042300	-0.78478300	0.14297100
H	-4.09320400	-0.53867100	0.54886500
H	-3.68570200	-2.08809100	0.10159100
C	-1.83075800	0.70893800	0.03430500
H	-1.79183600	-1.12582900	1.13845500
H	-1.48000000	-1.32642700	-0.59206800
O	-2.78320400	1.47567600	-0.07091000
O	-0.60819200	1.14302800	0.08859300
Hg	1.12385100	-0.08561200	-0.01057700

Molecule: Mn-GLY-01

N	-3.09489800	-0.35730300	-0.01718000
H	-2.95002300	-1.03516800	0.73439200
C	-2.06397100	0.71552800	0.03876300
H	-3.03374300	-0.86701700	-0.90127400
H	-4.03360900	0.03693500	0.06793300
C	-0.66760800	0.12336500	-0.00470000
H	-2.22343500	1.38483700	-0.80252000
H	-2.19996600	1.27205200	0.96331500
O	-0.52646700	-1.11120800	0.00519100
O	0.27778000	0.97636500	-0.03685300
Mn	2.17936100	-0.08980500	0.00429300

Molecule: Mn-GLY-02

N	-2.63210800	-1.02757500	0.01968900
H	-3.09679700	-0.58357200	-0.77521000
C	-1.16390700	-0.79482200	-0.04105800
H	-3.02702800	-0.61483700	0.86756200
H	-2.84697200	-2.02619200	0.00937000
C	-0.85410800	0.69690300	-0.01314000
H	-0.70310700	-1.29326000	0.80875600
H	-0.78705800	-1.24039000	-0.95890500
O	-1.79420400	1.50221400	0.03238100
O	0.38038300	1.01170000	-0.04295300
Mn	2.09217500	-0.26290100	0.01281500

Molecule: Ni-GLY-01

N	2.87205100	-0.55423300	0.00022800
H	2.69503200	-1.13050700	-0.82658300
C	2.01742100	0.65903400	-0.00021600
H	2.69545000	-1.12961800	0.82774700
H	3.85903800	-0.28473100	-0.00015400
C	0.56381000	0.30616000	-0.00008700
H	2.24380200	1.25108500	0.88433600
H	2.24375600	1.25036700	-0.88527200
O	0.16180700	-0.89580200	-0.00025100
O	-0.30298000	1.23264000	0.00013600
Ni	-1.72140800	-0.16295800	0.00003800

Molecule: Ni-GLY-02

N	2.78522700	-0.88133200	0.00005600
H	3.17615900	-0.41850800	0.82444900
C	1.30293900	-0.79977700	-0.00011700
H	3.17633000	-0.41818000	-0.82406900
H	3.08826800	-1.85786700	-0.00010200
C	0.85458800	0.64445700	-0.00004800
H	0.92740500	-1.30932400	-0.88535900
H	0.92715800	-1.30948900	0.88492600
O	1.66519700	1.55507900	0.00014900
O	-0.43545400	0.86171200	-0.00024500
Ni	-1.91339300	-0.24713300	0.00005400

Molecule: Zn-GLY-01

N	2.74136700	-0.96338400	-0.00021500
H	2.39037000	-1.45671900	-0.82366200
C	2.28288800	0.45271700	0.00023000
H	2.39096600	-1.45668900	0.82348500
H	3.76225700	-1.01319400	-0.00054200
C	0.77130000	0.57041000	0.00009500
H	2.68342800	0.94126600	0.88496400
H	2.68363200	0.94199100	-0.88400100
O	0.11775500	-0.53647900	0.00015600
O	0.27726100	1.69917400	-0.00021200
Zn	-1.81951600	-0.22177600	-0.00000800

Molecule: Zn-GLY-02

N	2.71030000	-0.99943700	-0.11836700
H	3.30259900	-0.32228500	0.36880500
C	1.28156700	-0.77003000	0.22195000
H	2.86795200	-0.87471800	-1.12070300
H	2.99939100	-1.94510600	0.13943100
C	0.91917100	0.69597300	0.05551300
H	0.66785000	-1.40192100	-0.41508300
H	1.12759700	-1.05931100	1.26000600
O	1.81402700	1.52838900	-0.09577800
O	-0.33140000	0.99024400	0.12505100
Zn	-1.83343100	-0.23684400	-0.04342800

Molecule: Cd-ILE-01

N	1.67143900	2.49411600	-0.23471700
H	1.46704900	2.63535200	0.75717200
H	1.03350900	3.09096000	-0.76526800
H	2.63096100	2.79698900	-0.41620100
C	1.50582800	1.05457700	-0.61912400
H	1.70163400	1.00185900	-1.68754800
C	2.52791800	0.18044000	0.13900700
C	0.06358800	0.64652800	-0.36319500
H	3.46910500	0.73888200	0.09579700
C	2.14975800	-0.02466200	1.60625100
C	2.75277400	-1.14454500	-0.59996900
O	-0.66020700	1.35159200	0.37837100
H	1.26313300	-0.65714600	1.69547000
H	2.96375600	-0.51216900	2.14053600
H	1.94744000	0.91729700	2.12000600
H	2.92820100	-0.92572200	-1.65657600
H	1.84281800	-1.74568500	-0.54992900
C	3.92859700	-1.94734300	-0.05287900
H	3.75118600	-2.28654800	0.96869600
H	4.10217200	-2.83283400	-0.66690600
H	4.84608100	-1.35335100	-0.05521000
O	-0.33610400	-0.42641300	-0.90336400
Cd	-2.40098800	-0.38162700	0.06671600

Molecule: Cd-ILE-02

N	2.08112300	-1.62875300	1.38960300
H	2.72224200	-2.04404100	0.70826100
H	1.55987400	-2.39516200	1.82010600
H	2.61962400	-1.15246900	2.11615600
C	1.15015200	-0.68035000	0.69815900
H	0.35780100	-0.45231800	1.40818500
C	1.89244300	0.62127900	0.33204300
C	0.57201800	-1.41592100	-0.51580100
H	2.41077100	0.92333500	1.24889100
C	2.93286700	0.42404400	-0.77054100
C	0.89052600	1.73110900	-0.00939700
O	1.24146900	-2.31921800	-1.02911400
H	2.45394400	0.20935900	-1.72810900
H	3.52899300	1.32781900	-0.88766200
H	3.62389300	-0.39003600	-0.54617600
H	0.11443300	1.74890200	0.76534400
H	0.39098800	1.48849100	-0.95041800
C	1.52234500	3.11474600	-0.11036300
H	2.22839100	3.17883000	-0.93919700
H	0.75437200	3.87278600	-0.27455600
H	2.05586400	3.37209200	0.80793100
O	-0.56457600	-1.03170000	-0.96523700
Cd	-2.05346500	0.11982500	0.10279700

Molecule: Cu-ILE-01

N	0.76420200	2.47535800	-0.28020200
H	0.52741700	2.61018400	0.70609700
H	0.05413200	2.95931300	-0.83519600
H	1.66911500	2.91777400	-0.45586300
C	0.82969900	1.02204100	-0.63616400
H	1.01444600	0.97467700	-1.70752000
C	1.98440600	0.32932400	0.12113400
C	-0.51518100	0.40720700	-0.35260400
H	2.82557300	1.02659600	0.04800900
C	1.66289200	0.10047200	1.59785400
C	2.39302500	-0.95873300	-0.60467700
O	-1.36530700	0.99836200	0.37392500
H	0.88748800	-0.66014200	1.71728800
H	2.55134800	-0.24433100	2.12426600
H	1.32623600	1.01022600	2.09817400
H	2.54122800	-0.72545000	-1.66213900
H	1.57405300	-1.67959800	-0.55339000
C	3.66639700	-1.58565000	-0.04769700
H	3.53121000	-1.94836200	0.97201500
H	3.97005700	-2.43597100	-0.66095800
H	4.48871000	-0.86579900	-0.04305900
O	-0.81673700	-0.73242900	-0.81969300
Cu	-2.58556900	-0.63041600	0.11423300

Molecule: Cu-ILE-02

N	-1.46952500	-1.85983600	-1.28406500
H	-2.01992300	-2.31803000	-0.55265000
H	-0.87098700	-2.57202600	-1.70771000
H	-2.10355700	-1.49816400	-1.99999400
C	-0.64817100	-0.75544900	-0.69741500
H	0.05547500	-0.44458600	-1.46847300
C	-1.54984200	0.43798900	-0.32424300
C	0.12395200	-1.34637100	0.48469000
H	-2.09051400	0.67862000	-1.24628100
C	-2.57135000	0.10029600	0.76122700
C	-0.69952600	1.65902700	0.04227200
O	-0.35527100	-2.28331000	1.11623900
H	-2.08329300	-0.09988100	1.71676400
H	-3.25288800	0.93833000	0.90008800
H	-3.18381600	-0.76547400	0.50245600
H	0.10078600	1.76472300	-0.70098300
H	-0.21479200	1.48431600	1.00667200
C	-1.49312400	2.95897500	0.09828600
H	-2.25113400	2.93597600	0.88236900
H	-0.83082000	3.80157900	0.30370200
H	-1.99606000	3.15101300	-0.85246800
O	1.26415800	-0.81508500	0.79330200
Cu	2.23398200	0.42837300	-0.18138900

Molecule: Fe-ILE-01

N	0.66302600	2.45979600	-0.15905200
H	0.44985800	2.53657700	0.83998500
H	-0.06949300	2.95790400	-0.67218200
H	1.55426600	2.93160800	-0.33032100
C	0.76257700	1.02907200	-0.58930100
H	0.97500600	1.03272000	-1.65683300
C	1.90155700	0.31195700	0.17837900
C	-0.57627500	0.39454300	-0.36615200
H	2.68288100	1.07112800	0.28417200
C	1.46662100	-0.13863600	1.57186400
C	2.48128300	-0.82227800	-0.67247200
O	-1.45150300	0.95847900	0.35772300
H	0.75992500	-0.97022600	1.51217800
H	2.32903800	-0.47393900	2.14449800
H	0.99723800	0.66948300	2.13593500
H	2.74285900	-0.41298100	-1.65161700
H	1.71373300	-1.58261400	-0.84117000
C	3.71684200	-1.45880000	-0.04570000
H	3.47572600	-2.00097300	0.86940400
H	4.17385700	-2.16646200	-0.73912100
H	4.46409100	-0.69991800	0.20008700
O	-0.90125800	-0.73729100	-0.85375900
Fe	-2.71475800	-0.68367400	0.09719000

Molecule: Fe-ILE-022

N	1.39540000	-1.95779800	1.25593300
H	1.94391200	-2.40910900	0.51887300
H	0.77689400	-2.66823600	1.65485400
H	2.03330500	-1.63500200	1.98710600
C	0.61026600	-0.80634100	0.71187000
H	-0.01833000	-0.44315900	1.52417500
C	1.56209400	0.31752600	0.24386500
C	-0.27085600	-1.35657500	-0.39702300
H	2.31531400	0.38910000	1.03495600
C	2.26612200	-0.01550200	-1.07004100
C	0.82859300	1.66162300	0.19832900
O	-0.03004000	-2.39384700	-0.97017800
H	1.56917500	0.02412700	-1.91018200
H	3.05960000	0.70573100	-1.25864500
H	2.72812500	-1.00377100	-1.05656800
H	0.31870300	1.80734400	1.15481600
H	0.05296800	1.62458500	-0.57563300
C	1.74915900	2.84730700	-0.06444800
H	2.19202600	2.80515500	-1.05987700
H	1.19378000	3.78339400	0.01147800
H	2.56129800	2.87886700	0.66616000
O	-1.31661700	-0.61141700	-0.73586900
Fe	-2.31513800	0.61536400	0.17038500

Molecule:Hg-ILE-01

N	2.68103600	2.37203200	-0.46260000
H	2.59656000	2.65381400	0.51699600
H	2.14786700	3.04346100	-1.01936000
H	3.66407600	2.43083600	-0.73797700
C	2.16730900	0.98016800	-0.66826200
H	2.18655500	0.80292900	-1.74041600
C	3.08229300	-0.03897800	0.03986500
C	0.71756800	0.94475100	-0.18143900
H	4.10013300	0.24791900	-0.24717700
C	2.96923400	0.02391500	1.56262100
C	2.83921000	-1.45164000	-0.50721500
O	0.29278100	1.79449100	0.59464700
H	1.97848500	-0.29447600	1.89537000
H	3.70493700	-0.63643300	2.01973100
H	3.15355500	1.02540800	1.95537900
H	2.78608700	-1.39895500	-1.59755000
H	1.86743800	-1.81224600	-0.16339400
C	3.92482700	-2.44562800	-0.10775400
H	3.95671900	-2.60300500	0.97123400
H	3.74333100	-3.41538800	-0.57490200
H	4.91119800	-2.10038000	-0.42727900
O	0.06162500	-0.05402600	-0.66617600
Hg	-1.95752600	-0.20683700	0.02666100

Molecule: Hg-ILE-02

N	-2.72369600	-1.41921000	-1.36861800
H	-3.38134000	-1.76498200	-0.66469000
H	-2.33868800	-2.23452600	-1.84970800
H	-3.22852200	-0.84538800	-2.04662200
C	-1.63455500	-0.62548800	-0.71029800
H	-0.89792900	-0.41119700	-1.48085600
C	-2.23192200	0.69479000	-0.16467900
C	-1.02463000	-1.54067600	0.35181800
H	-2.97018600	0.99624300	-0.91530500
C	-2.95316800	0.51013900	1.16955000
C	-1.18789500	1.81631100	-0.10964500
O	-1.70634900	-2.44971800	0.82129900
H	-2.24354400	0.27941900	1.96728700
H	-3.47644000	1.42535900	1.44326200
H	-3.69554600	-0.28836100	1.13114800
H	-0.59477500	1.78680800	-1.02880900
H	-0.49900100	1.63204900	0.72193600
C	-1.80171900	3.20450800	0.03725100
H	-2.33334800	3.31741200	0.98286100
H	-1.02675100	3.97176600	0.00013100
H	-2.50820100	3.40236900	-0.77281600
O	0.18853500	-1.34045300	0.75403700
Hg	1.56757500	0.05789200	-0.04942700

Molecule: Hg-ILE-011

N	2.68096900	2.37230300	-0.46205100
H	2.59656800	2.65385100	0.51762600
H	2.14770800	3.04383700	-1.01859700
H	3.66398000	2.43120900	-0.73750600
C	2.16729800	0.98045800	-0.66799600
H	2.18673200	0.80336800	-1.74017100
C	3.08215200	-0.03878500	0.04017900
C	0.71748800	0.94496000	-0.18142400
H	4.10012100	0.24882800	-0.24567600
C	2.96763600	0.02282200	1.56288300
C	2.84023600	-1.45111300	-0.50827700
O	0.29251600	1.79467300	0.59458700
H	1.97698500	-0.29708400	1.89448000
H	3.70376700	-0.63691100	2.02018400
H	3.15028000	1.02427300	1.95652300
H	2.78917800	-1.39769100	-1.59868100
H	1.86784700	-1.81200300	-0.16650700
C	3.92520100	-2.44527100	-0.10747400
H	3.95509900	-2.60342600	0.97145500
H	3.74469700	-3.41470700	-0.57567700
H	4.91212900	-2.09966600	-0.42489000
O	0.06169500	-0.05389800	-0.66622700
Hg	-1.95744500	-0.20693300	0.02659500

Molecule: Mn-ILE-11

N	1.09623700	2.51023200	-0.15227100
H	0.70264800	2.62870100	0.78463100
H	0.59512600	3.14932300	-0.77219300
H	2.08367400	2.77334400	-0.14104800
C	0.92421900	1.09014100	-0.60245700
H	1.15008200	1.07371100	-1.66563900
C	1.90708600	0.17102200	0.15606500
C	-0.53365000	0.70525300	-0.37145300
H	2.85164400	0.72403200	0.19718600
C	1.45582300	-0.11750100	1.58846500
C	2.17227200	-1.10701100	-0.64789200
O	-1.20906900	1.37915700	0.43327900
H	0.57414100	-0.76297400	1.59961800
H	2.24696300	-0.62341200	2.13972200
H	1.21261800	0.79497400	2.13602800
H	2.44109800	-0.82036300	-1.66805600
H	1.25174600	-1.69039700	-0.71754400
C	3.28431200	-1.96966200	-0.05949900
H	3.00553200	-2.38969500	0.90788000
H	3.51361100	-2.80307600	-0.72599400
H	4.20057300	-1.38969400	0.07768500
O	-0.95730900	-0.32357600	-0.98161300
Mn	-2.85729800	-0.77256800	0.11683700

Molecule: Mn-ILE-23

N	1.58462900	-1.63779100	1.38766100
H	2.22147900	-2.05199600	0.70220900
H	1.06564200	-2.40589200	1.81713800
H	2.12603600	-1.16386500	2.11316800
C	0.64977700	-0.68810500	0.70036200
H	-0.12969600	-0.44987000	1.42146200
C	1.40281800	0.59894500	0.30643200
C	0.03748100	-1.42694900	-0.49644700
H	1.98869200	0.87727200	1.18957400
C	2.36323200	0.38858100	-0.86458700
C	0.41641700	1.74231300	0.04333300
O	0.66235700	-2.38655700	-0.97519700
H	1.81532300	0.21129900	-1.79251200
H	2.98208700	1.27374000	-1.00495300
H	3.03726000	-0.45422300	-0.70373100
H	-0.29979800	1.78043200	0.87188700
H	-0.16176500	1.52003700	-0.85727000
C	1.08479600	3.10417100	-0.10385700
H	1.70277300	3.15802100	-1.00102000
H	0.33283700	3.89203200	-0.17520900
H	1.72126500	3.32522500	0.75661700
O	-1.06590200	-0.97819900	-0.94080600
Mn	-2.47973300	0.26226500	0.19062500

Molecule: Ni-ILE-01

N	0.72576500	2.48370500	-0.30743800
H	0.42798400	2.61690300	0.66281000
H	0.06061300	2.97800400	-0.90742600
H	1.64614700	2.91504900	-0.42236000
C	0.79438500	1.03200000	-0.66827900
H	0.98370100	0.98379400	-1.73796400
C	1.94564500	0.33300900	0.09211400
C	-0.54410600	0.40769200	-0.38490100
H	2.80697400	0.99498300	-0.04695400
C	1.66813800	0.19181100	1.58774500
C	2.27961300	-1.00737900	-0.57471200
O	-1.33080800	0.90333600	0.48072100
H	0.87082100	-0.53163200	1.77295900
H	2.56276600	-0.15924500	2.09923800
H	1.38439000	1.13741100	2.05205100
H	2.39882200	-0.83999100	-1.64812100
H	1.43793400	-1.69331700	-0.45410400
C	3.54596500	-1.65278000	-0.02235100
H	3.43014800	-1.94925700	1.02095400
H	3.79480400	-2.54958700	-0.59245100
H	4.39632400	-0.96940600	-0.08607700
O	-0.90111800	-0.65570000	-0.97503900
Ni	-2.55586500	-0.64738700	0.15058400

Molecule: Ni-ILE-022

N	1.70423400	-1.78096200	1.23882600
H	2.21612100	-2.22026500	0.46917600
H	1.19842700	-2.52223100	1.72892500
H	2.37359600	-1.36185900	1.88785700
C	0.75879000	-0.74449900	0.71459300
H	0.12384200	-0.45595800	1.55002400
C	1.55156000	0.48741500	0.21896300
C	-0.08861500	-1.42141900	-0.36089000
H	2.27503700	0.69307300	1.01508400
C	2.31473200	0.21500800	-1.07631200
C	0.64538300	1.71938200	0.10637000
O	0.33321400	-2.40782500	-0.94324300
H	1.62585200	0.06377000	-1.91027900
H	2.95429400	1.06260300	-1.31833700
H	2.95995600	-0.66221500	-1.00540600
H	0.00797800	1.76867600	0.99736100
H	-0.01508700	1.59890300	-0.75892900
C	1.40810100	3.03250300	-0.02608000
H	1.99316600	3.07117200	-0.94570200
H	0.71347300	3.87399300	-0.04200700
H	2.08939500	3.17515000	0.81586300
O	-1.25757300	-0.90676000	-0.64150500
Ni	-2.30680400	0.39886700	0.14509700

Molecule: Zn-ILE-01

N	1.43307000	2.37618300	-0.60729800
H	1.24399500	2.72444300	0.33592400
H	0.88950900	2.94544400	-1.25903300
H	2.42562700	2.49877400	-0.81903200
C	1.04176300	0.93269000	-0.71067400
H	0.97909000	0.70454600	-1.77128200
C	2.12429200	0.04745800	-0.06402900
C	-0.34782900	0.78568000	-0.08743300
H	3.05727200	0.34313500	-0.55658600
C	2.27172900	0.29269000	1.43691000
C	1.88076800	-1.43030600	-0.39353600
O	-0.74073300	1.60834000	0.74320600
H	1.38319500	-0.03816200	1.97868900
H	3.12674500	-0.26082000	1.82329000
H	2.44174900	1.34522200	1.67131800
H	1.62175000	-1.51634700	-1.45187200
H	1.01570200	-1.78651700	0.17008900
C	3.08528000	-2.31915800	-0.10245200
H	3.33449400	-2.33318300	0.95949700
H	2.88117600	-3.34783900	-0.40530900
H	3.96660800	-1.97699300	-0.65053200
O	-0.99478300	-0.23644300	-0.51408200
Zn	-2.82834300	-0.55881600	0.06400700

Molecule: Zn-ILE-02

N	1.58486000	-1.76692000	1.32908400
H	2.20349400	-2.18491000	0.62984600
H	1.02463700	-2.52173700	1.73028000
H	2.15109600	-1.35086200	2.07172200
C	0.70494000	-0.73395400	0.69663000
H	-0.03125600	-0.45666500	1.44940600
C	1.53863400	0.50682500	0.31514300
C	-0.00575100	-1.38786300	-0.49141000
H	2.10291900	0.76117600	1.21906600
C	2.53262600	0.22918700	-0.81247100
C	0.62548200	1.69700100	-0.00287300
O	0.53482900	-2.34009800	-1.05839300
H	2.01587800	0.04320000	-1.75616200
H	3.18825900	1.08753700	-0.95117900
H	3.17219500	-0.62918000	-0.60032700
H	-0.14126100	1.76949500	0.77743000
H	0.10079600	1.50564100	-0.94215600
C	1.36672300	3.02506800	-0.10007500
H	2.07578500	3.03228500	-0.92892600
H	0.66303300	3.84327800	-0.26328100
H	1.91976500	3.23616900	0.81830600
O	-1.12953800	-0.88503700	-0.86217100
Zn	-2.24525400	0.33388400	0.17257500
